# Identification of long-chain alkane-degrading (LadA) monooxygenases in *Aspergillus flavus via in silico* analysis

**DOI:** 10.3389/fmicb.2022.898456

**Published:** 2022-08-30

**Authors:** Madushika Perera, Sulochana Wijesundera, C. Dilrukshi Wijayarathna, Gamini Seneviratne, Sharmila Jayasena

**Affiliations:** ^1^Department of Biochemistry and Molecular Biology, Faculty of Medicine, University of Colombo, Colombo, Sri Lanka; ^2^Department of Chemistry, Faculty of Science, University of Colombo, Colombo, Sri Lanka; ^3^National Institute of Fundamental Studies, Kandy, Sri Lanka

**Keywords:** *Aspergillus flavus*, LadA, fungal long-chain alkane monooxygenases, flavin-dependent monooxygenases, alkanes, hydrocarbon biodegradation, bioremediation

## Abstract

Efficient degradation of alkanes in crude oil by the isolated *Aspergillus flavus* MM1 alluded to the presence of highly active alkane-degrading enzymes in this fungus. A long-chain alkane-degrading, LadA-like enzyme family in *A. flavus* was identified, and possible substrate-binding modes were analyzed using a computational approach. By analyzing publicly available protein databases, we identified six uncharacterized proteins in *A. flavus* NRRL 3357, of which five were identified as class LadAα and one as class LadAβ, which are eukaryotic homologs of bacterial long-chain alkane monooxygenase (LadA). Computational models of *A. flavus* LadAα homologs (*Af1-Af5*) showed overall structural similarity to the bacterial LadA and the unique sequence and structural elements that bind the cofactor Flavin mononucleotide (FMN). A receptor-cofactor-substrate docking protocol was established and validated to demonstrate the substrate binding in the *A. flavus* LadAα homologs. The modeled *Af1*, *Af3, Af4*, and *Af5* captured long-chain *n*-alkanes inside the active pocket, above the bound FMN. Isoalloxazine ring of reduced FMN formed a π–alkyl interaction with the terminal carbon atom of captured alkanes, C_16_–C_30_, in *Af*3–*Af*5 and C_16_–C_24_ in *Af*1. Our results confirmed the ability of identified *A. flavus* LadAα monooxygenases to bind long-chain alkanes inside the active pocket. Hence *A*. *flavus* LadAα monooxygenases potentially initiate the degradation of long-chain alkanes by oxidizing bound long-chain alkanes into their corresponding alcohol.

## Introduction

Enzymes that catalyze the first step of long-chain *n*-alkane (C_16_–C_30_) oxidation, LadA and AlmA, have been recorded only in bacteria to date (Feng et al., 2007; Li et al., 2008; Wang and Shao, 2011). While LadA oxidizes long-chain alkanes from C_15_ to C_36_, AlmA is reported to be involved in the metabolism of even longer-chain alkanes (Feng et al., 2007; Wentzel et al., 2007).

Degradation of long-chain alkanes have been demonstrated in fungi such as *Aspergillus, Penicillium, Fusarium*, and *Rhizopus* (Yamada-Onodera et al., 2002; Mittal and Singh, 2009; Abd et al., 2016; El-Hanafy et al., 2017; Al-Hawash et al., 2018; Barnes et al., 2018; Wemedo et al., 2018). The filamentous fungus, *Aspergillus flavus* MM1, has been shown to rapidly degrade *n*-alkanes ranging from C_12_ to C_30_ in crude oil (Perera et al., 2021).

However, fungal alkane hydroxylases reported so far have only been shown to degrade short (C_1_–C_4_) and medium (C_5_–C_15_) chain alkanes (Sanglard and Loper, 1989; Scheller et al., 1996; Zimmer et al., 1996; Iida et al., 2000; Vatsyayan et al., 2008). Eukaryotic cytochrome P450 (CYP52) has been involved in the degradation of < C_18_ *n*-alkanes in *Candida maltosa*, *Candida tropicalis, Yarrowia lipolytica*, and a filamentous fungus *Aspergillus terreus* (Sanglard and Loper, 1989; Scheller et al., 1996; Zimmer et al., 1996; Iida et al., 2000; Vatsyayan et al., 2008).

A bacterial LadA, a flavin-binding monooxygenase, was the first structurally and functionally characterized enzyme responsible for long-chain alkane metabolism. Initially described in the thermophile *Geobacillus thermodenitrificans* NG80-2 (Feng et al., 2007; Li et al., 2008), LadA homologs have later been shown in other *Geobacillus* spp. and utilized a broad range of *n*-alkanes (C_10_–C_30_) (Tourova et al., 2016). *G. thermoleovorans* B23, an extreme thermophile, was shown to possess a cluster of two types of chromosomal *lad* genes, *ladA* and *ladB*. The genes *ladA*α*_*B*23_, ladA*β*_*B*23_*, and *ladB_B23_* encode enzymes that oxidize *n*-alkanes from C_13_ to C_23_ and from C_26_ to C_30_ effectively (> 60%) at 70°C (Kato et al., 2001; Boonmak et al., 2014). In contrast, a moderate halophile, *Amycolicicoccus subflavus* DQS3-9A1T, isolated from oil “mud” (45°C) also encodes a LadA homolog induced by C_16_–C_36_ alkanes (Nie et al., 2013). Homologs of *ladA*s have also been detected in psychrophile genomes like *Octadecabacter arcticus*, *O*. *antarcticus*, and *Terroglobus saanensis in silico* (Bowman and Deming, 2014).

Enzymes and mechanisms involved in the oxidation of long-chain *n*-alkanes in fungi have not been previously reported. Unraveling the underlying fungal metabolism and characterization of enzymes involved in the breaking down of these highly resistant hydrocarbons are therefore an important area that needs exploration. Highly efficient degradation of crude oil by *Aspergillus flavus* MM1 observed in our previous work (Perera et al., 2019, 2021) alluded to the presence of highly catalytic long-chain alkane-degrading enzymes in these fungi. In this study, we report eukaryotic homologs of the bacterial LadA for the first time. In the current study, LadA-like flavin-dependent long-chain alkane monooxygenases (LadA) were identified in *Aspergillus flavus* through *in silico* protein sequence analyses, including protein phylogeny. Good quality 3D models of LadA homologs were predicted using comparative modeling. Subsequent docking simulations were carried out for the structure-based functional annotation of the protein. Herein, we report the presence of long-chain alkane hydroxylase systems in the filamentous fungi for the first time.

## Materials and methods

### *In silico* identification and analysis of long-chain alkane monooxygenase-like flavin mononucleotide-dependent monooxygenases in bacteria and fungi

The crystal structure and amino acid sequence of the LadA from *Geobacillus thermodenitrificans* NG80-2 (Li et al., 2008) (PDB ID: 3B9O) were retrieved from the RCSB Protein Data Bank^1^ ^2^ (Berman et al., 2000, 2002). The crystal structure, 3B9O, represents the homodimer of *G. thermodenitrificans* LadA bound to two FMN molecules.

Similarity searches were conducted by HMMER (web version 2.41.1) (Potter et al., 2018) using profile hidden Markov models (HMM) and the position-specific iterative BLAST (PSI-BLAST) (Altschul et al., 1997). Two hundred and sixty protein sequences with percentage amino acid identities ranging from 99 to 20% at *E*-scores < 10^–10^ and bit score > 50 carrying bacterial luciferase family domain (PF00296) were selected, including organisms as phylogenetically diverse as possible (Supplementary Figures 1, 2B). Bacterial and fungal sequences sharing > 30% sequence identity over their entire length with *E*-scores < 10^–10^ and > 50-bit score are considered homologous (Pearson, 2013).

### Phylogenetic tree construction

A representative sample (*n* = 63) of the 260 derived protein sequences including *Aspergillus flavus* NRRL3357 (taxonomy ID: 332952), a close relative to *Aspergillus flavus* MM1 (data not shown) sequences, the structurally and functionally characterized LadAs (Li et al., 2008; Nie et al., 2013), LadB_B23_ (Boonmak et al., 2014), and alkanesulfonate monooxygenase (SsuD) from *Escherichia coli* were selected (Eichhorn et al., 2002). The remaining sequences were selected preferentially from organisms in which the degradation of long-chain *n*-alkanes have being demonstrated so far (Kummer et al., 1999; Pepi et al., 2000; Yamada-Onodera et al., 2002; Saul et al., 2005; Hasanuzzaman et al., 2007; Zanaroli et al., 2010; Hidayat and Tachibana, 2012; Liu et al., 2014; Shiri et al., 2014; Zheng et al., 2016; Brzeszcz and Kaszycki, 2018; Li et al., 2019, 2020; Daccò et al., 2020; Santoyo et al., 2021).

The phylogenetic tree was constructed by the maximum likelihood (ML) method using MEGA X: Molecular Evolutionary Genetics Analysis across Computing Platforms (Kumar et al., 2018), using the LG substitution matrix (Le and Gascuel, 2008) as the optimal model. Structurally and functionally characterized *Vibrio harveyi* luciferase (LuxA) (Fisher et al., 1996) and F420-dependent secondary alcohol dehydrogenase (Adf) enzymes from *Methanoculleus thermophilus* (Aufhammer et al., 2004), distant family members of bacterial luciferase family, were used as outgroups, as LadA has been identified as a luciferase family enzyme. Bootstrapping analysis was carried out to evaluate the tree topology of the data by performing 1,000 resampling events. The tree graphic was generated using Interactive Tree of Life (iTOL) v6 (Letunic and Bork, 2021).

### Multiple sequence alignment of *Aspergillus flavus* LadAα homologs and functionally characterized bacterial long-chain alkane monooxygenases

Functionally and structurally characterized LadA protein sequence from *G. thermodenitrificans* NG80-2 (Li et al., 2008) and other functionally characterized LadA homologs from *G. thermoleovorans* B23 (LadAα_B23_ and LadAβ_B23_) (Boonmak et al., 2014) and *Amycolicicoccus subflavus* (AS9A_3890) (Nie et al., 2013) were aligned along with identified *A. flavus* LadAα homologs, using T-COFFEE with homology extension (PSI-Coffee) (Notredame et al., 2000; Robert and Gouet, 2014). Conservations within the alignment were analyzed for structural or functional significance.

### Secondary structure determination of *Aspergillus flavus* LadAα homologs

The secondary structures of identified *A. flavus* LadAα homologs were predicted from primary amino acid sequences using the PSIPRED 4.0 algorithm (McGuffin et al., 2000).

### 3D structure prediction of *Aspergillus flavus* LadAα homologs and analysis

Prediction of 3D structures of the five identified *A. flavus* LadAα homologs was carried out using SWISS-MODEL automated modeling server^3^ (Benkert et al., 2011; Bertoni et al., 2017; Waterhouse et al., 2018). To achieve an unbiased 3D structure prediction, the homologous sequences were not manually edited. Further, the models were predicted using Automated Modeling mode in SWISS-MODEL (Waterhouse et al., 2018) with no post-refinements to the models obtained.

The validity of the predicted models was evaluated using PROCHECK (Laskowski et al., 1993), VERIFY3D (Einsenberg et al., 1997), PROSA (Wiederstein and Sippl, 2007), and ERRAT (Colovos and Yeates, 1993). Protein surface representation was predicted using UCSF Chimera (Pettersen et al., 2004) v1.1.

### Molecular docking simulations

#### Preparation of protein structures for docking

In the present study, the monomeric behavior of the proteins was considered in docking simulations in order to analyze the binding modes of the ligands and the active pocket residues. Therefore, chain A of LadA (3B9O_A) was isolated from 3B9O using UCSF Chimera 1.12. The bound cofactor and crystalline water molecules were deleted from chain A and saved in mol2/pdb format as the template for analysis (3B9O_A). Then, this partially prepared template and five built models of *A. flavus* LadAα homologs were prepared for docking by the addition of polar H atoms and the charges for each atom into the atomic coordinates of the molecule using AutoDock Tools (ADT) (Sanner, 1999; Morris et al., 2009).

#### Preparation of ligands for docking

##### Flavin mononucleotide coenzyme preparation

Ideal coordinates of FMN were retrieved from RCSB Protein Data Bank, geometrically optimized with ORCA 4.2.1 (Neese, 2012) and saved in mol2/pdb format as the final structure of FMN for analysis (FMNopt). The optimized structure was prepared for docking as described above and defining the rotatable bonds.

##### Alkane preparation

The 3D structures of the alkane molecules (C_16_-C_30_) were retrieved from RCSB Protein Data Bank and Zinc database (Irwin and Shoichet, 2005).^4^ Retrieved molecules were geometrically optimized with ORCA 4.2.1 (Neese, 2012) and were saved in mol2 format. Geometrically optimized ligand structures were prepared for docking similarly to FMN.

#### Docking simulations

AutoDock Vina (Trott and Olson, 2009) was used to dock the optimized ligands with protein models (flexible ligand-rigid receptor method).

Validation of the docking protocol (re-docking) was performed in order to evaluate the “receptor-cofactor-substrate docking” approach being used in this study, using AutoDock Vina and UCSF Chimera (Pettersen et al., 2004) v1.1. For this purpose, prepared 3B9O_A and optimized FMN cofactor (as mentioned above) were docked (blind docking) in AutoDock Vina. The resultant docked conformations were visualized, and the receptor-cofactor complex was generated (3B9O_A: FMNopt). The search space was decreased to the recognized active pocket in the 3B9O_A: FMNopt complex. The final docking simulations were performed with the alkane substrate. The dimensions of the grid box of the active pocket for alkane molecules were specified as 25 × 25 × 25 Å^3^ with a grid spacing of 1.000 Å. The receptor-cofactor-substrate complex was generated. Binding conformations and active pocket residues were visualized and analyzed by UCSF Chimera and BIOVIA Discovery Studio Visualizer (Biovia, 2015). The docked conformation and active pocket residues obtained were compared with the crystal structure (3B9O) and pre-recognized active pocket residues (Li et al., 2008) to determine the accuracy of the re-docking analysis.

Similarly, the receptor-cofactor-substrate docking approach adopted here was continued for docking simulations of the modeled *A. flavus* LadAα homologs with FMN and alkane molecules. Docked conformations and free energy of bound ligands were predicted accordingly.

## Results

### Phylogenetic relationship of LadA homologs

**FIGURE 1:**
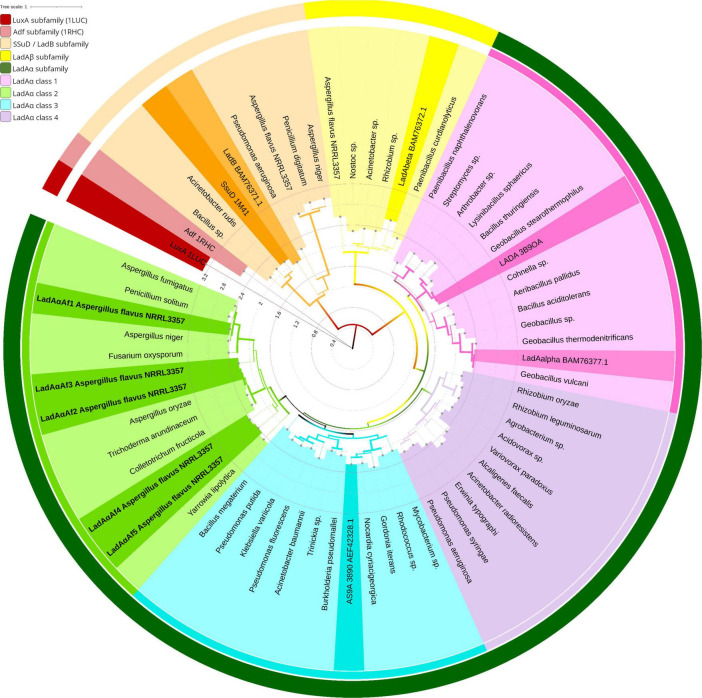
Protein phylogeny of the LadA and LadB like FMN-dependent monooxygenases in bacteria and fungi. The evolutionary relationship was inferred by Maximum Likelihood method based on the LG substitution matrix (Le and Gascuel, 2008). The tree with highest log likelihood (−22645.26) is shown. The percentage of trees in which the associated taxa clustered together (1,000 bootstrap replications) is represented by the width of the branch. The tree is drawn to scale, with branch lengths measured in the number of substitutions per site (Internal tree scale). Leaves are colored according to their affiliation to clusters. Outer circle shows LadAα subfamily (dark green), LadAβ subfamily (yellow), LadB/SsuD family (orange), Adf (light maroon), and LuxA (maroon). The inner circle shows four classes of LadAα subfamily: LadAα Class 3 (light green), LadAα Class 2 (blue), LadAα Class 4 (purple), LadAα Class 1 (pink). The structurally and functionally characterized LadAs, LadB_B23_, and alkanesulfonate monooxygenase (SsuD) from *Escherichia coli* (Fisher et al., 1996; Eichhorn et al., 2002; Aufhammer et al., 2004; Li et al., 2008; Boonmak et al., 2014) are highlighted in each cluster. The tree was inferred by MEGA X (Kumar et al., 2018) and visualized using iTOL (Letunic and Bork, 2021) v6. GenBank accession numbers correspond to all leaf labels are shown in Supplementary Figure 2A.

Bacterial and fungal LadA homologs formed two major clades in the phylogenetic tree (Figure 1). They were designated as class LadAα and LadAβ and with respect to the relationship with functionally characterized LadA homologs (LadAα_B23_ and LadAβ_B23_) from *Geobacillus thermoleovorans* B23 identified in each class (Boonmak et al., 2014).

Class LadAα and LadAβ proteins shared > 30% (*E*-scores < 10^–10^ and bit score of > 50) sequence identity. Six sequences, which have been annotated as “uncharacterized protein” were detected in *Aspergillus flavus* NRRL 3357 proteome. Five of them having 45–50% sequence identity (with > 97% query cover) were grouped under Class LadAα with *G. thermodenitrificans* LadA (Li et al., 2008) and the functionally characterized *Amycolicicoccus subflavus* homolog (YP_004495128) (Nie et al., 2013). One (35% sequence identity) was grouped under Class LadAβ.

Within the LadAα proteins, four distinct groups were evident (LadAα1-4) (Figure 1). The *G. thermodenitrificans* LadA and LadAα_B23_ fell within LadAα1, while *Amycolicicoccus subflavus* homolog fell into the second group LadAα2. The five *A. flavus* LadAα homologs fell within LadAα3 group. LadAα4 could be distinguished as a more distant branch (branch length) of bacterial LadAα-type proteins. The five *Aspergillus flavus* NRRL 3357 protein sequences were designated LadAα*_*Af*1_*—LadAα*_*Af*5_*; *Af1* (XP_041143749.1), *Af2* (XP_041147879.1), *Af3* (XP_041147424.1), *Af4* (XP_041140192.1), and *Af5* (XP_041142110.1) were selected for further analysis.

### Amino acid residue conservation in *Aspergillus flavus* LadAα homologs

**FIGURE 2:**
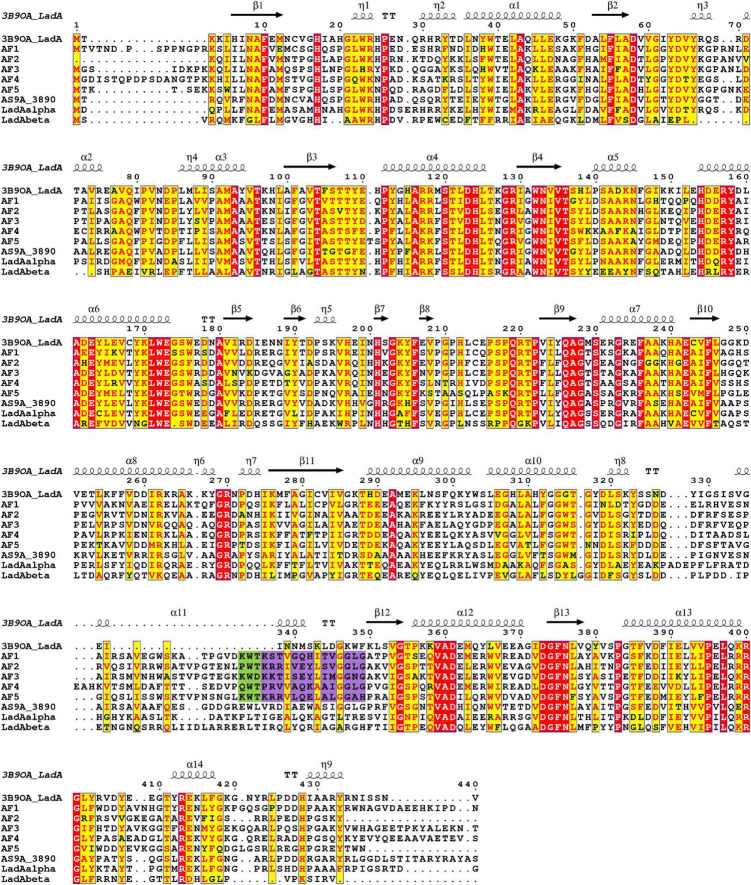
Multiple sequence alignment of putative homologs of *Aspergillus flavus* NRRL 3357 (*Af1*, *Af2*, *Af3*, *Af4*, and *Af5*) comparison to *G. thermodenitrificans* NG80-2 LadA (Li et al., 2008) (3B9O_A), functional homologs from *G. thermoleovorans* B23 (Boonmak et al., 2014) (LadAα_B23_, LadAβ_B23_), and *Amycolicicoccus subflavus* (Nie et al., 2013) (AS9A_3890). Identical residues are indicated in red, and the most conserved residues are highlighted in yellow. The secondary structure of LadA (3B9O_A) is indicated above the sequence alignment. The purple highlight indicates the additional region in *A. flavus* homologs compared to LadA (3B9O_A). Conserved substrate-binding residues upstream of the additional region in *A. flavus* homologs are highlighted in green. Alignment graphic was generated using ENDscript server (Robert and Gouet, 2014).

Comparison of amino acid sequences of the identified *A. flavus* LadAα homologs (*Af1*-*Af5*), and four functionally characterized bacterial LadA homologs from *Geobacillus* spp. and *Amycolicicoccus subflavus* (Li et al., 2008; Nie et al., 2013; Boonmak et al., 2014) showed that there are several highly conserved residues among these enzymes, even though the overall amino acid sequence identity was low (Figure 2). Fifty-eight residues were conserved among *A. flavus* LadAα homologs identified in this study. Some of these residues were in areas involved in maintaining the integrity of the quaternary structure of enzymes and others in the putative active site regions.

The amino acid positions in the alignment (Figure 2) are presented as concurrently numbered columns from the beginning of the alignment with respect to the *G. thermodenitrificans* LadA (3B9O_A). These numbers are used to reference the amino acid positions of the identified *A. flavus* LadAα homologs throughout this manuscript and may differ from the actual residue number (Supplementary Figures 6–11).

### Dimerization

The *A. flavus* LadAα homologs (*Af1*-*Af5*) demonstrated a potential for dimerization, which has been reported to be required for catalytic activity in luciferase family proteins (Friedland and Hastings, 1967; Fisher et al., 1996).

Among the critical H-bond-forming amino acid pairs, required for dimerization, the Arg117 and Asp58 pair (Li et al., 2008) was conserved in all except *Af4*, where Arg117 was replaced by synonymous amino acid lysine. In the other critical H-bond-forming amino acid pairs, Arg118 and Glu110 pair (Li et al., 2008) was conserved in *Af1*, 2, and 4, while in *Af3*, Glu110 was replaced by aspartate, and in *Af5*, Arg118 was replaced by lysine. Both these replacements are conservative substitutions and are not expected to affect the H-bonding pattern (Figure 2). Additionally, N-terminal two-hairpin loop formation between residues 175 and 222 (insertion segment 5 in *G*. *thermodenitrificans* LadA), which plays an important role in the dimer formation, was observed in 3D models of all five *A. flavus* homologs.

### Stabilization of flavin in the active pocket

A solvent-inaccessible cavity located in front of the flavin C4a is important to stabilize and sequester the isoalloxazine ring of the flavin during the oxygenation reaction in the absence of a non-prolyl *cis*-peptide bond (Fisher et al., 1996; Shima et al., 2000; Aufhammer et al., 2004; Alfieri et al., 2007; Li et al., 2008). In *G*. *thermodenitrificans* LadA, it is achieved through the isolation effect from the cavity lined by Met12, Ala57, Val59, His138, the 4′ OH group of the FMN ribityl chain, and the terminal carbon of the alkane chain (Li et al., 2008). Ala57 and Val59 are conserved in all five *A. flavus* LadAα homologs. Met12 is conserved in *Af2*-*Af5*. However, His138 is replaced by tyrosine in *Af1*, 2, and 3 and by tryptophan in *Af4* and phenylalanine in *Af5*. A similar replacement of His138 by tyrosine was also observed in the *Amycolicicoccus subflavus* DQS3-9A1T homolog, as well as in the *Geobacillus* LadA homologs, LadAα_B23_ and LadAβ_B23_ (Li et al., 2008; Nie et al., 2013; Boonmak et al., 2014).

### Cofactor preference

Ser230, which interacts with the phosphate group of FMN, was conserved in all the *A. flavus* LadAα homologs, similarly in *G*. *thermodenitrificans* LadA as well as in other SsuD and LuxA subfamilies (Aufhammer et al., 2004; Li et al., 2008; Liberles et al., 2012; Ahmed et al., 2015; Mascotti et al., 2016; Chenprakhon et al., 2018).

In addition, a search of the CDD: NCBI’s conserved domain database (Marchler-Bauer et al., 2015) identified all the *A. flavus* homologs to contain FMN-binding domain in their amino acid sequences. Therefore, it can be deduced that *A. flavus* homologs of LadAα use FMN as a cofactor.

### Secondary structure determination of *Aspergillus flavus* LadAα homologs

Secondary structural elements obtained by PSIPRED 4.0 showed that *A. flavus* LadAα homologs consisted of eight main α-helices and β-sheets which form the TIM-barrel core (β/α)_8_) of each protein and additional α-helices and β-sheets in regions corresponding to the insertion segments in *G. thermodentrificans* LadA (Li et al., 2008; Supplementary Figure 3).

### 3D structure prediction and analysis of LadAα homologs in *Aspergillus flavus*

As the five identified *A. flavus* LadAα homologs showed 45–50% sequence identity to the *G. thermodentrificans* LadA, it was selected as a template for comparative/homology modeling. Even though the resultant models possessed a TIM-barrel core and additional regions, especially the two-hairpin loop and the large bulge similarly to 3B9O_A, the local model quality of the models was low (data not shown). This may reflect the lack of eukaryotic LadA structural data and the fact that sequence identities were only marginal for homology modeling (Sali and Blundell, 1993; Baker and Sali, 2001).

In order to obtain 3D models of high local model quality which guarantee that important functional sites of a protein have been modeled correctly (Bordoli et al., 2009), SWISS-MODEL automated modeling mode was selected where multiple templates are considered for modeling the correct conformation of the target protein (Waterhouse et al., 2018; Supplementary File 1A). Further, the local model quality of the obtained 3D models of *A. flavus* LadAα homologs (Supplementary Figure 4A) was shown to be highly reliable through multiple approaches (different structure validation tools PROCHECK, VERIFY3D, PROSA, and ERRAT) (Supplementary Figure 4B).

The resultant statistical scores of the Ramachandran plot obtained through PROCHECK (Laskowski et al., 1993) showed > 90% residues of the predicted structures of the five *A. flavus* LadAα homologs (*Af1*–*Af5*) were in the most favored region. VERIFY3D analysis (Jed et al., 1992; Einsenberg et al., 1997) indicated that more than 90% of the residues in each of the five predicted structures (*Af1*-*Af5*) had an average 3D–1D score ≥ 0.2, ensuring that each residue position in their three-dimensional environment is reliable in terms of sequence-structure compatibility. The Z-scores predicted by ProSA (Wiederstein and Sippl, 2007) for the five models (*Af1*-*Af5*) (i.e., −9.01, −9.39, −9.2, −9.37, and −9.03, respectively) are in the range of native proteins of similar size. Overall, the residue energies are largely negative (energy plot), predicting that the local model quality is reliable. The ERRAT (Colovos and Yeates, 1993) scores for the 3D models of *Af1*-*Af5* were in the range of 84–91, which reflects a high quality for non-bonded atomic interactions.

Further, the expected topology and the sequence of secondary structure elements predicted by PSIPRED were at the expected positions in the predicted 3D models. Each of these consisted of TIM-barrel (β/α)_8_ fold as the protein core with additional insertion regions, especially the N-terminal two-hairpin loop formed between residues 175 and 222 and the large bulge from residues 289 to 354 (Figure 2) at the C-terminal end of the TIM-barrel, which are important for dimerization and the catalytic mechanism. However, it is important to note that the large bulge has an additional region (an α-helix according to PSIPRED 4.0) in *A. flavus* LadAα homologs extending the bulge than observed in *G*. *thermodenitrificans* LadA (Figure 2).

**FIGURE 3:**
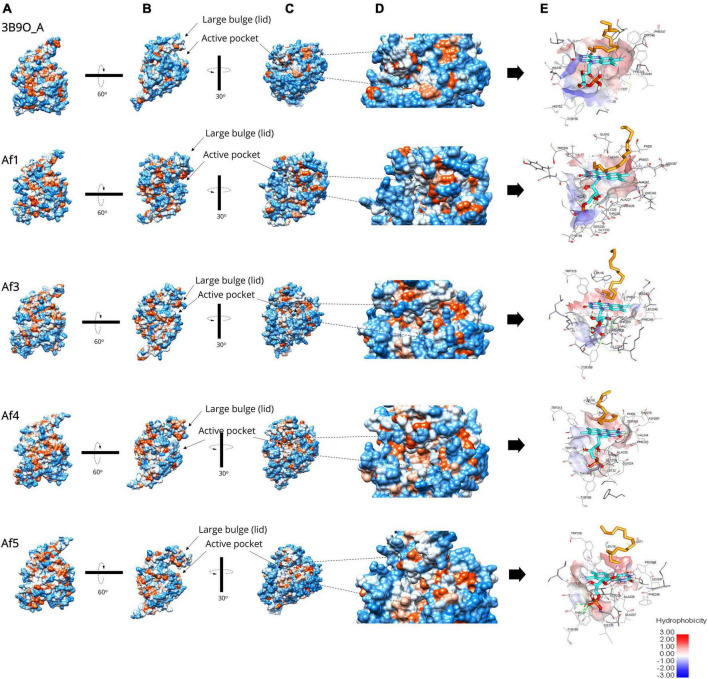
Surface representation of the predicted *A. flavus* LadAα homologs *Af1*, *Af2*, *Af4*, and *Af5* enzymes and the active pocket compared to the *G. thermodenitrificans* LadA (3B9O chain A) crystal structure (Li et al., 2008) analyzed by UCSF Chimera (Pettersen et al., 2004) v1.1. and BIOVIA Discovery Studio Visualizer (Biovia, 2015). **(A)** The five structures aligned in the same view; hydrophobicity is depicted by color range from red (+) to blue (−). **(B)** Illustrates the large bulge (lid) gating the underlying active pocket. **(C)** A detailed view of the active pocket inside the enzyme. **(D)** The enlarged view of the active pocket inside the enzyme. **(E)** Illustration of the inner surface of the active pocket, alkane (orange), FMN (cyan), active site amino acids (gray).

The root mean squared deviation (RMSD) of each 3D model of *A. flavus* LadAα homologs (*Af1*-*Af5*) with *G*. *thermodenitrificans* LadA (3B9O_A) crystal structure was 1.547 Å, 1.220 Å, 1.526 Å, 1.552 Å, and 1.511 Å for *Af1*–*Af5*, respectively (Figure 3C). This deviation was acceptable where ∼1.0 Å RMSD is generally observed in the core Cα atoms of proteins sharing 50% sequence identity (Rost, 1999; Addou et al., 2009).

Further, the surface hydrophobicity of the identified enzymes and their active pockets were similar compared to the *G. thermodenitrificans* LadA (3B9O chain A) crystal structure (Li et al., 2008; Figure 3), which confirmed the ability to capture and bind alkane molecule inside the active pocket of identified *A*. *flavus* LadA homologs, that is, *Af1*, *Af3*, *Af4*, and *Af5*.

### Molecular docking simulations

#### Validation of adapted receptor-cofactor-substrate docking approach (re-docking analysis)

Re-docking analysis was successful with the adapted receptor-cofactor-substrate docking approach described in methods in which 3B9O_A was docked with FMNopt and hexadecane. Similar conformation of the FMNopt (−7.7 Kcal/mol) (Supplementary Figures 5A,B) and hexadecane (C_16_) (−4.5 Kcal/mol), as well as the active site residues (Supplementary Figure 5C) described in *G. thermodentrificans* LadA (Li et al., 2008) (3B9O), was achieved through blind docking. Although the ribityl side chain and phosphate moieties of FMN in docked complex showed a minor conformational difference, the binding residues were consistent with the crystal structure. This difference in conformation can be attributed to the rigid receptor state used in the docking approach.

Despite the slight difference in the placement of ribityl side chain and phosphate moieties of FMN in our docked complex, hexadecane positioning at the *si*-face of FMN and the active site residues in the binding pocket around the bound FMN and alkane (hexadecane) were met with the above-described typical binding pose of FMN (Li et al., 2008; Kawabata, 2010; Chenprakhon et al., 2018; Supplementary Figure 5C).

Active pocket residues of the re-docked pose of 3B9O_A with FMN and hexadecane were Ile18, Phe55, Ala57, Asp58, Val59, Thr104, Asn133, Val135, Thr136, Ser137, His138, His154, Tyr158, Ala227, Gly228, Met229, Ser230, Phe245, Leu246, Gly247, His311, Tyr312, Gly315, Lys347, Trp348, and Phe349 within 5 Å (Supplementary Figure 5C). Phe10, Gly233, and Asn376 were also in the active pocket as viewed by UCSF Chimera (Pettersen et al., 2004) v1.12 and BIOVIA Discovery Studio Visualizer (Biovia, 2015).

Some of the active pocket residues were hydrogen bonded while others formed hydrophobic interactions with the docked cofactor and alkane molecule. Most importantly, the alkane molecule (> C_16_) docked in the cavity that formed above the FMN *si*-face with the terminal carbon atom of the alkane forming a π–alkyl interaction with the π-electron cloud of the isoalloxazine ring of reduced FMN visualized by BIOVIA Discovery Studio Visualizer. This π–alkyl interaction with the terminal carbon atom of the alkane suggested terminal hydroxylation of the long-chain alkanes (Li et al., 2008). Therefore, the adapted receptor-cofactor-substrate docking approach in this study can be concluded as successful.

#### Cofactor and substrate docking simulations of 3D models of *Aspergillus flavus* LadAα homologs

Modeled 3D structures of *A. flavus* LadAα homologs were docked with FMN cofactor and then with the alkane molecules (C_16_–C_30_). In blind docking, FMN was found to enter the binding pocket only in *Af1*, *Af3*, *Af4*, and *Af5*. Therefore, the docking of alkane molecules was continued only with *Af1*, *Af3*, *Af4*, and *Af5*. Additionally, a similar procedure was carried out for 3B9O_A with FMNopt (generated through re-docking) and the rest of the alkanes (C_17_–C_30_) in order to validate the results obtained for *A. flavus* LadAα homologs by comparing binding free energies, binding conformation, and common active pocket residues.

When the FMN cofactor and the alkane substrates were docked into the four *A. flavus* LadAα homologs, after decreasing the search space (grid box in AutodockTools), binding energies of FMN and *Af1*, *Af3*, *Af4*, and *Af5* corresponding to the best conformation compared to 3B9O_A (−7.7 Kcal/mol) were −8.9 Kcal/mol, −6.8 Kcal/mol, −7.7 Kcal/mol, and −7.5 Kcal/mol, respectively.

**FIGURE 4:**
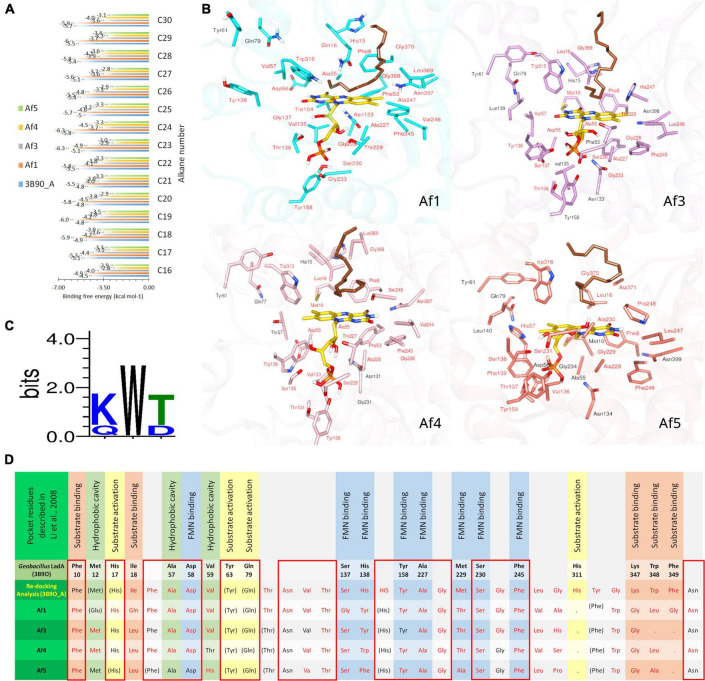
Docking simulations of *A. flavus* LadAα homologs with FMNopt and hexadecane. **(A)** Binding free energy of *n*-alkanes (C_16_–C_30_) into 3B9O A: FMNopt, *Af1*: FMNopt, *Af3*: FMNopt, *Af4*: FMNopt, and *Af5*: FMNopt complexes. **(B)** Active pocket residues of each of the four hexadecane (dark brown) (C_16_)-bound *A. flavus* LadAα homologs: FMN (gold) complexes. **(C)** Conservation of substrate-binding residues, (K/Q) W (T/D) in *A. flavus* LadAα homologs *Af1*, *Af3*, *Af4*, and *Af5*. **(D)** Comparison of active pocket residues of *A. flavus* LadAα homologs with FMNopt and hexadecane: *Af1*, *Af3*, *Af4*, and *Af5* and re-docking analysis of 3B9O_A visualized by BIOVIA Discovery Studio Visualizer and UCSF Chimera; residues within 5 Å from the bound ligands inside the active pocket are labeled in red lettering, while others are in black. Active pocket residues of *G. thermodentrificans* LadA (Li et al., 2008) that were conserved but observed outside the active pocket in the *A*. *flavus* homologs are depicted in brackets (FMN binding 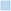, Substrate binding 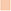, Hydrophobic cavity 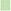, Substrate activation 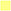, other 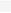, Conserved (100%) 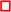.

FMN-bound *Af1*, *Af3*, *Af4*, *Af5*, and 3B9O_A enzyme complexes were docked with *n*-alkanes C_16_–C_30_. Binding free energies of *Af1*: FMNopt, *Af3*: FMNopt, *Af4*: FMNopt, *Af5*: FMNopt, and 3B9O_A with *n*-alkanes (C_16_–C_30_) are depicted in Figure 4A.

All *n*-alkanes (C_16_–C_30_) successfully docked inside the cavity above the FMN *si*-face in each protein model, coordinating in a similar manner (Figure 4B). However, *Af1*: FMNopt complex was able to bind *n*-alkanes from C_16_ to C_24_ while *Af3*: FMNopt, *Af4*: FMNopt, and *Af5*: FMNopt complexes bound to all *n*-alkanes from C_16_ to C_30_ chain length. The terminal carbon of each alkane molecule formed a π–alkyl interaction with the π-electron cloud of the isoalloxazine ring of reduced FMN according to the BIOVIA Discovery Studio visualization, except for the alkanes > C_24_ captured by *Af1*. Although *Af1*: FMNopt complex was able to accommodate C_25_–C_30_ alkanes inside the active pocket, they did not bind the terminal carbon atom of the alkane.

The pocket residues in all the docked complexes visualized by BIOVIA Discovery Studio Visualizer are tabulated and submitted as supplementary data (Supplementary Figures 6–10), and the pdb files of *A*. *flavus* LadAα homologs in complex with coenzyme FMN and traicontane (C_30_H_62_) are available in ModelArchive^5^ with the accession codes mentioned in Supplementary File 1B. Conservation of active pocket residues was observed between four (*Af1* and *Af3*-*Af5*) alkane-bound *A. flavus* LadAα: FMN complexes compared to binding with 3B9O_A (Figure 4D and Supplementary Figures 6–10).

Although, substrate binding residues Lys347, Trp348, Phe349 (KWF) shown in *G. thermodenitrificans* LadA appeared to be replaced in the *A. flavus* homologs in the docking simulations (Figure 4D), this is likely due to the unbiased modelling process that was adopted where no post refinements were made to the 3D models. In the multiple sequence alignment, a conserved (K/Q) W (T/D) region (Figure 4C and green highlight in Figure 2) could be observed in all *A. flavus* homologs, within the additional region (highlighted in purple Figure 2). Although this region appears within the large bulge in the modelled structures, this could be due to lack of a template having this region, for modelling. Therefore, we hypothesize that these conserved residues, are located at the surface, lining the active pocket in the actual protein, similar to *G. thermodenitrificans* LadA (Li et al., 2008) and binds the alkane molecule.

## Discussion

*Aspergillus flavus* MM1, previously isolated in our laboratory, was shown to degrade long-chain alkanes present in crude petroleum oil (C_12_–C_30_ in our sample) to an extent of 98 ± 2% within 7 days at 30°C under static conditions (Perera et al., 2021). This suggested that this fungus possesses multiple pathways to catalyze the oxidation of a broad range of alkanes. Although several fungi have been reported (El-Hanafy et al., 2017; Al-Hawash et al., 2018; Barnes et al., 2018; Wemedo et al., 2018; Perera et al., 2021) to degrade a broad range of alkanes at moderate temperatures, the only pathway that has so far been identified in fungi is the CYP52 system, which is known to catalyze the oxidation of short-to-medium-chain alkanes, but not long-chain alkanes (> C_16_) (Sanglard and Loper, 1989; Scheller et al., 1996; Zimmer et al., 1996; Iida et al., 2000; Vatsyayan et al., 2008).

To date, LadAs that catalyze the first step of the terminal oxidation of long-chain *n*-alkanes have been experimentally proved to be possessed mostly by extremophilic bacteria (Park and Park, 2018). LadA (3B9O_A) was first structurally and functionally characterized in the thermophilic bacterium, *Geobacillus thermodenitrificans* NG80-2 (Feng et al., 2007; Li et al., 2008). Genes homologous to LadA were subsequently identified and functionally characterized in several other and the halophiles, *Amycolicicoccus subflavus* and *Alcanivorax* sp. strain Est-02 (Nie et al., 2013; Boonmak et al., 2014).

In this study, we identified five novel fungal LadAα homologs (*Af1*–*Af5*) in *A. flavus* NRRL 3357. Protein phylogenetic analysis further identified the evolutionary relationship among bacterial LadAs and the novel fungal homologs (Figure 1). The bacterial and fungal LadA homologs identified in this study fall into two major classes: LadAα and LadAβ. The *A flavus* LadA homologs formed a distinct group among structurally and functionally characterized LadAα proteins. Therefore, our study suggests that the identified *A*. *flavus* sequences may be LadAα homologs despite the sequence divergence (Rost, 1999; Addou et al., 2009; Pearson, 2013).

Analysis of structural architecture by Li et al. (2008) showed that the LadA monooxygenases are derived from or belong to the luciferase family of enzymes. Accordingly, *A. flavus* LadAα homologs had in its protein core the TIM-barrel fold, which is distinctive for the luciferase family, as well as the additional insertion regions (IS 1–5) observed in the *G. thermodenitrificans* LadA subfamily (Wierenga, 2001; Li et al., 2008; Ellis, 2010).

*Aspergillus flavus* LadAα homologs have a unique sequence and structural elements that indicate its preference for FMN as a cofactor. Ser230, which is required for binding reduced FMN in other FMN-binding luciferases (LadA, SsuD, and LuxA subfamilies), is conserved in *A*. *flavus* homologs. The lid-gating mechanism of the large bulge and solvent-inaccessible cavity located in front of the bound FMN *si*-face sequester and protect the FMN intermediates formed through the reaction with molecular oxygen for later oxidation of the alkanes (Fisher et al., 1996; Eichhorn et al., 2002; Roper and Grogan, 2016). The structural elements observed in *A*. *flavus* homologs support this mechanism of FMN (Lin et al., 2004; Zhan et al., 2008; Campbell et al., 2009; Figure 4D).

The *in silico* docking simulations demonstrated that the *A. flavus* LadAα homologs bound long-chain alkanes in thermodynamically favorable manner, suggesting that functional similarity may be preserved to *Geobacillus* LadA.

Out of the five *A*. *flavus* LadAα sequence homologs identified, only *Af1*, *Af3*, *Af4*, and *Af5* were able to capture FMN inside their binding pockets in the blind docking experiments. All of these FMN-bound LadAα homologs, except *Af1*, were able to bind *n*-alkanes from C_16_ to C_30_ with their terminal carbon atoms, suggesting terminal oxidation of the alkanes, similar to bacterial LadA (3B9O_A) (Li et al., 2008). Long-chain alkanes bound to bacterial LadA were found to be oxidized into its corresponding primary alcohol *via* a terminal oxidation pathway in which the terminal carbon atom of the bound alkane forms the π–alkyl interaction with the reduced FMN (Li et al., 2008).

However, it was observed that although the FMN-bound *Af1* accommodated C_25_–C_30_ alkanes, there was no bond formation with the terminal carbon atom. Therefore, it is hypothesized that *Af1* may degrade these alkanes through the subterminal oxidation pathway. In this pathway, a subterminal carbon atom of the alkane forms the π–alkyl interaction with the reduced FMN, and the alkane is oxidized to the corresponding secondary alcohol, which is further oxidized into the corresponding ester by Baeyer–Villiger monooxygenase (Minerdi et al., 2012). This hypothesis is further supported by the reports on the presence of Baeyer–Villiger monooxygenase in *A. flavus*, which is an essential enzyme in organisms that undergo subterminal oxidation pathway (Ferroni et al., 2016; Fürst et al., 2019).

In summary, a family of distant functional homologs of the bacterial long-chain alkane-degrading monooxygenases (LadAα homologs) was identified in the proteome of *A. flavus* using computational approaches. Four out of the five identified protein sequences were shown to functionally bind long-chain alkanes up to C_30_ through molecular docking simulations. We plan to further investigate the alkane inducibility of these LadAα homologs and functionally characterize them using a heterologous expression system.

## Data availability statement

The datasets presented in this study can be found in online repositories. The names of the repository/repositories and accession number(s) can be found in the article/Supplementary material.

## Author contributions

SJ and MP conceptualized the study and contributed to the study design and data interpretation. MP additionally carried out all the experimental work and wrote the manuscript. All authors contributed to the manuscript revisions and approved the final version.

## Footnotes

1
www.rcsb.org

2
www.wwpdb.org

3
https://swissmodel.expasy.org

4
http://zinc.docking.org

5
http://modelarchive.org
