# Supplementary material for: Identification of long-chain alkane-degrading (LadA) monooxygenases in Aspergillus flavus via in silico analysis
Source: Front Microbiol. 2022 Aug 30;13:898456. doi: 10.3389/fmicb.2022.898456 (PMC9468676; doi:10.3389/fmicb.2022.898456)
Supplement: Supplementary file 1 [file Data_Sheet_1.pdf]

Supplementary File 1. Accession codes of protein models and docked complexes deposited in ModelArchive (modelarchive.org); **(A)** Pdb files of predicted protein models. **(B)** Pdb files of LadAα homologs in *A. flavus* in complex with coenzyme FMN and Triacontane ($C_{30}H_{62}$)

The models are available in ModelArchive (modelarchive.org) with the following accession codes,

A

Model / Complex (.pdb)	Accession code
LadAαAf1	ma-o4ags
LadAαAf2	ma-vlx9d
LadAαAf3	ma-xuduh
LadAαAf4	ma-m3hh3
LadAαAf5	ma-daifv

B

Model / Complex (.pdb)	Accession code
LadAαAf1 + FMN + C30	ma-vvsjt
LadAαAf3 + FMN + C30	ma-ywcfg
LadAαAf4 + FMN + C30	ma-ts22w
LadAαAf5 + FMN + C30	ma-kd0rq
