# Supplementary material for: Identification of long-chain alkane-degrading (LadA) monooxygenases in Aspergillus flavus via in silico analysis
Source: Front Microbiol. 2022 Aug 30;13:898456. doi: 10.3389/fmicb.2022.898456 (PMC9468676; doi:10.3389/fmicb.2022.898456)
Supplement: Supplementary file 2 [file Image_1.pdf]

Supplementary Figure 1. Two hundred and sixty amino acid sequences with percentage identities ranging from 99% to 20% at E-scores <10–10 and bit score >50 carrying bacterial luciferase family domain (PF00296) aligned by MUSCLE.

```
                                                                         1         10         20         30         40
LADA_Geobacillus_thermodenitrificans_WP_011888513   .................MTKKIHINAFEMNCV.GHIAHGLWRHPEN.QRHR.YTDLNYWTELA
Geobacillus_sp._LEMMY01_WP_079935807.1:1-440        .................MTKKIHINAFEMNCV.GHIAHGLWRHPEN.QRHR.YTDLNYWTELA
Geobacillus_stearothermophilus_ABV66256.1:1-440     .................MTKEIHINAFEKNCV.GHIAHGLWRHPEN.QRHR.YTDLNYWTELA
Geobacillus_sp._B4113_201601_WP_061912600.1:1-440   .................MVKKIYINAFEMNCV.GHIAHGLWRHPEN.QRHR.YTDLNYWVELA
Geobacillus_sp._B4113_201601_5WP_061912600.1:1-44   .................MVKKIYINAFEMNCV.GHIAHGLWRHPEN.QRHR.YTDLNYWVELA
Bacillus_thuringiensis_6WP_029441578.1:1-435        .................MKKKIYLNAFEMNCV.GHISHGLWAHPDN.QRHR.YTDLNYWTELA
Lysinibacillus_sphaericus_WP_103976887.1:2-422      ...................KKIYWNAFEMNCV.GHINHGLWKIPGN.KRIN.YKNLNYWTELA
Lysinibacillus_sphaericus_WP_069508309.1:2-422      ...................KKIFWNAFEMNCV.GHINHGLWKIPGN.KRIN.YKNLNYWTELA
Rummeliibacillus_sp._SL167_10_WP_146551437.1:2-42   ...................KRIYWNAFEMNCV.GHINHGLWKIPGN.KRAH.YKNLEYWTDLA
Paenibacillus_sp._DXL2_WP_110843609.1:1-459         .................MAKKIHLNAFEMNCV.GHLAHGLWRHPDN.NRHR.YTDLDYWTELA
Paenibacillus_naphthalenovorans_WP_074730028.1:1-   .................MPKKMHLNAFEMNCV.GHLAHGLWRHPDN.TRHR.YTDITYWTELA
Cohnella_sp._HS21_WP_130606586.1:1-453              .................MGKKILLNAFDMNTA.MHGAHGLWKHPAN.QRHR.YKDLNYWVELA
Streptosporangium_subroseum_15_WP_089205784.1:1-4   .................MTDIIHFNLFEMNCV.GHIQHGLWTHPDN.NRHR.YTDLEYWTELA
Arthrobacter_sp._Hz2_WP_120147654.1:1-455           .................MTRQIAFNLFEMNCV.GHISHGLWVHPEN.NRHR.FNDLDFWVETA
Nonomuraea_solani_WP_103963668.1:1-453              .................MSGIIHFNLFEMNCV.GHIQHGLWTHPAN.NRHR.YTDLDYWTDLA
Arthrobacter_ruber_WP_105030338.1:1-455             .................MTRQIAFNLFEMNCV.GHISHGLWVHPEN.NRHR.FNDLDFWTETA
Bacillus_aciditolerans_20_WP_121446947.1:1-456      .................MTKQIRLNVFDMTSA.VHNSHGLWKHPNS.ERHTRYKDLDYWIELA
WP_137163763.1:1-458                                .................MPRPIRFNAFSMNAA.SHQSPGLWRHPRN.TSVA.FNRLGYWTDLA
Aeribacillus_pallidus_WP_130157503.1:4-457          ...................KRIYLNSFDMNCV.AHQSPGLWVHPED.QSYR.YKDIEYWVELA
Bacillus_megaterium_24WP_098602848.1:4-460          ...................KRIYLNAFDMNCV.AHQSPGLWLHPED.QSHR.YNDIEYWIELA
Streptomyces_sp._150FB_WP_078877664.1:5-455         ...................KKLHVNLFEMNCV.GHISHGLWVHPDN.NRHR.FNDIDFWLELA
Arthrobacter_sp._DWC3_WP_104051071.1:1-454          .................MTRQIAFNLFEMNCV.GHISHGLWVHPEN.NRHR.FNDLDFWVETA
Cohnella_thermotolerans_WP_027093898.1:1-456        .................MAKQIRLNAFEMTSA.MLNSHGLWKHPDNVRTRR.YKDLDYYTELA
Paenibacillus_bovis_28WP_144921429.1:1-452         ................MTKKRIYLNAFEMNCA.GHQSPGLWTHPDD.RSHQ.YKDSEYWIELA
Bacillus_aciditolerans_WP_121447760.1:1-452         ................MTKKRMYLNAFDMNCV.GHQSPGLWTHPED.QSHR.YKDSEYWIELA
Leifsonia_sp._ALI-44-B_30_WP_077053645.1:1-455      .................MTDNIAFNLFEMNCV.GHISHGLWRHPDN.NRHR.FNDLDFWVEEA
Parageobacillus_thermoglucosidasius_WP_064552269.   .................MAKQIILNAFEMASA.MHNSHGLWKHPES.KRQRGYKDIDYWIKMA
Geobacillus_sp._Y4.1MC1_WP_013399886.1:1-459        .................MAKQIILNAFEMASA.MHNSHGLWKHPES.KRQRGYKDIEYWIKMA
Bacillus_methanolicus_35WP_004434428.1:1-462        .................MGKQIILNAFEMTSA.MHNSHGLWKHPES.KRQRRYKDLDYWIEMA
Terribacillus_halophilus_WP_077309754.1:1-448       ................MTKKRIYLNAFDMNCV.GHQSPGLWTHPED.QAHR.YKDLDYWTELA
Paraburkholderia_kururiensis_WP_017777532.1:1-453   .................MTRQIRLNAFDMNCV.GHQSPGLWAHPRD.ASWR.YRELGYWTELA
Gracilibacillus_massiliensis_WP_058308419.1:1-456   .................MTKEIILNAFEMTSA.MHNSHGLWKHPKS.ERHRQYKDLDYWIDLA
Streptomyces_yanglinensis_WP_103888551.1:5-452      ...................KKLHVNLFEMNCV.GHISHGLWVHPDN.NRHR.FNDIEFWLELA
Trinickia_sp._7GSK02_40_WP_136896361.1:1-452        .................MTRQIRLNAFDMNCV.GHQSPGLWAHPRD.SSWR.YRELGYWTDLA
Cryobacterium_sp._NEAU-85_WP_123045138.1:1-448      .................MSQILHFNGFEMNTP.SHINHGLWTHPDN.RRHE.YTDLRYWTDTA
Alicyclobacillus_acidoterrestris_WP_021298468.1:4   ...................RRMYLNAFDMNCI.GHQSPGLWTHPED.QAYR.YTDMEYWVELA
Oceanobacillus_senegalensis_WP_085994229.1:1-452    ................MTKQRIYLNAFEINSA.GHQSPGLWTHPDD.QSHR.YKDMDYWTELA
Bacillus_sinesaloumensis_WP_077620983.1:1-449       ............................MNSS.MHNSHGFWKHPKN.QRHRRYKDLDYWVELA
Halopenitus_persicus_WP_021074147.1:6-457           .....................IHLNGFTMNCV.GHQSPGLWKHPDD.NSDA.YTDLEYWTDLA
Microbacteriaceae_bacterium                         .................MARELHFNGFEMNTP.SHINHGLWRHPGN.NRHR.YTDLSYWTETA
Lysinibacillus_sinduriensis_WP_036201100.1:1-458    .................MAKQLILNLFDMAGA.MHNSHGLWKHPQN.KRHLVYNDLNYWIELG
Bacillus_endophyticus_WP_113749053.1:5-457          ....................RIYLNAFDMNCV.VHQSPGLWTHPDD.QSHR.YNDINYWIELA
Pseudomonas_monteilii_WP_119371117.1:1-455          .................MPREIRLNAFEMNCV.GHQSPGLWRHPKD.RAWQ.YKDLDYWTDLA
Bacillus_endophyticus_50_WP_061801645.1:5-457       ....................RIYLNAFDMNCV.VHQSPGLWTHPDD.QSHR.YNDINYWIELA
Planococcus_maitriensis_WP_112233285.1:1-457        ................MTKKRIYLNAFEMVCAGGHQSPGLWLHEDD.RSHT.YKDSEYWIELA
Oceanobacillus_profundus_WP_118889880.1:1-447       ................MTKKRIFLNAFDMNTP.GHQSPGLWTHPDD.QSHR.YKDSAYWTELA
Burkholderia_pseudomallei_WP_141404579.1:1-452      .................MTRQIRFNAFDMNCV.GHQSPGLWAHPRD.ESWR.YRELSYWTGLA
Terribacillus_saccharophilus_WP_095261886.1:1-455   .................MGKQIILNAFDMNSA.MHNSHGLWKHPES.KRHRQYKDLNYWIDLA
Sediminibacillus_albus_WP_093212831.1:4-453         ...................KRIYLNAFDMNCA.GHQSPGLWTHPED.QSHK.YKDSDYWIELA
Sciscionella_marina_WP_020496929.1:1-448            .................MARILHFNGFEMNTP.SHINHGLWVHPGN.RRHE.YTDIGYWLDTA
Mycetocola_saprophilus_WP_043321267.1:1-452         .................MSKQLHFNAFEMNTV.SHINHGLWVHPDN.RRHE.YTDIEYWVETA
Lysinibacillus_macroides_WP_053993569.1:4-460       ...................KRIYLNAFDMNCV.AHQSPGLWRHPDD.QAWR.YKDIEYWVELA
Pseudomonas_putida_WP_086978777.1:1-458             .................MPREIRLNAFEMNCV.GHQSPGLWRHPKD.RAWQ.YKDLDYWTDLA
Sediminibacillus_halophilus_60_WP_074598704.1:4-4   ...................KRIYLNAFDMNCA.GHQSPGLWTHPDD.QSHK.YKDSEYWIELA
Rhizobium_oryzae_WP_085420425.1:1-450               .................MAREIWFNAFDMNCV.GHIQQGLWTHPRD.QSHR.YAELRYWTDYA
Pseudomonas_extremorientalis_WP_071491956.1:1-455   .................MSREIRLNAFDMNCV.GHQSPGLWAHPRD.RSWQ.YKDLEYWTDLA
Pseudomonas_fluorescens_WP_016977452.1:1-454        .................MSREIRLNAFDMNCV.GHQSPGLWAHPRD.RSWQ.YKDLEYWTDLA
Neorhizobium_sp._T25_27_WP_105419890.1:1-450        .................MTREIRFNAFDMNCV.GHIQQGLWAHPRD.QSHR.YGQLRYWTDYA
Pseudomonas_extremaustralis_WP_078833627.1:1-457    .................MAREIRLNAFDMNCV.GHQSPGLWAHPRD.RSWQ.YKDLEYWTDLA
Fictibacillus_aquaticus_WP_094253544.1:1-455        .................MTKQIILNAFEMTSA.MHNSHGLWKHPKS.KRHRGYKDLNYWIEMA
Pseudomonas_extremaustralis_KAA8559760.1:1-456      .................MSREIRLNAFDMNCV.GHQSPGLWAHPRD.RSWQ.YKDLEYWTDLA
Mycobacterium_sp._ACS1612_WP_067812332.1:1-454      .................MTRQIHLNAFDMNCV.THIVAGTWRHPES.QAAR.YKDLEYWTDLA
Bacillus_megaterium_WP_116075167.1:1-455            .................MEKHIYLNAFDMNCV.VHQTPGLWTYPSD.QAAE.YKTLDYWVKLA
Pseudomonas_frederiksbergensis_70_WP_123409596.1:   .................MAREIRLNAFDMNCV.GHQSPGLWAHPRD.RSWQ.YKDLEYWTDLA
Pseudomonas_citronellolis_SFC61163.1:1-452          .................MSKPLHVNLFEMNCV.SHIVHGLWAHPEN.QRHR.FNDIDYWTELA
Herbaspirillum_chlorophenolicum_WP_050468599.1:4-   ...................KQIRLNAFNMNCV.NHINHGLWTHPRD.RSQD.YTDIAYWTHLA
Arthrobacter_crystallopoietes_WP_074700854.1:1-45   .................MSKEIHFNGFEMNTV.SHINHGLWVHPDN.RRHE.YTDIEYWTETA
Terribacillus_goriensis_WP_038558686.1:1-438        .................MTKQIILQAFDMTAA.NHNAHGLWKHPEN.RRHREYKSLEYWMELA
Bacillus_megaterium_WP_025749753.1:1-455            .................MGKHIYLNAFDMNCV.VHQTPGLWTYPSD.QASE.YKTLDYWVELA
Neorhizobium_sp._T7_12_WP_105403044.1:1-451         .................MTREIRFNAFDMNCV.GHIQQGLWAHPRD.QSHR.YAQLRYWTAYA
Rummeliibacillus_sp._SL167_WP_146553619.1:4-459     ...................KRIYLNAFDMNCV.GHQSPGLWRHPKD.RSST.YNTIEYWVNLA
Burkholderia_sp._PAMC                               ...................KKILLNAFDMNCV.GHINHGLWTHPRD.RSTD.YRKLSYWTDLA
Pseudomonas_koreensis_WP_064585463.1:4-449          ...................KKILLNAFNMNCI.GHINHGLWTHPRD.TSTR.YNTLEYWTELA
Pseudomonas_fluorescens_80_WP_064116961.1:6-451     ...................KKILLNAFNMNCI.GHINHGLWTHPRD.TSTR.YNTIEYWTELA
Pseudomonas_rhodesiae_WP_040265276.1:4-450          ...................KKILLNAFNMNCI.GHINHGLWTHPRD.TSTQ.YKTLAYWTDLA
Collimonas_sp._OK412_WP_092393355.1:4-454           ...................KQIRLNAFNMNCV.GHINHGLWTHPRD.RSSD.YTSIAYWTEQA
Pseudomonas_marginalis_WP_058415544.1:1-456         .................MSREIRLNAFDMNCV.GHQSPGLWAHPRD.RSWQ.YKDLEYWTDLA
Pseudomonas_antarctica_WP_064452947.1:1-458         .................MSREIRLNAFDMNCV.GHQSPGLWAHPRD.RSWQ.YKDLEYWTDLA
Pseudomonas_protegens_WP_041115159.1:9-454          ...................KKILLNAFNMNCV.GHINHGLWTHPRD.NSTQ.YKTLEYWTELA
Pseudomonas_synxantha_WP_057024776.1:4-449          ...................KKILLNAFNMNCI.GHINHGLWTHPRD.NSTQ.YKTLEYWTELA
Rhizobium_leguminosarum_WP_128409501.1:2-451        ...................KTIQIYAFDMNCV.GHINHGLWTHPRD.RSAH.YTDLDYWTELA
Pseudomonas_chlororaphis_WP_038359779.1:6-451       ...................KKILLNAFNMNCI.GHINHGLWTHPRD.TSTR.YNTLEYWTELA
Pseudomonas_syringae_90_WP_024642725.1:1-456        .................MSRQIRLNAFDMNCV.GHQSPGLWAHPRD.RSWQ.YKDLEYWTDLA
Collimonas_fungivorans_WP_061540754.1:4-453         ...................KQIRLNAFNMNCV.GHINHGLWTHPRD.RSSD.YTSIEYWTEQA
Rhizobium_leguminosarum_WP_116409027.1:2-451        ...................KTMQIYAFDMNCV.GHINHGLWTHPRD.QSMR.YTDLDYWTEFA
Microvirga_ossetica_WP_099511173.1:1-454            .................MTKKINIYAFDMNCV.GHINHGLWTHPRD.LSSS.YTSLDYWVNLA
Pseudomonas_caspiana_WP_087268376.1:4-451           ...................KKILLNAFNMNCI.GHINHGLWTHPRD.NSTQ.YNTLEYWTELA
Pseudomonas_lundensis_WP_048375063.1:4-451          ...................KKILLNAFNMNCI.GHINHGLWTHPRD.NSTA.FNTLEYWTELA
Caballeronia_mineralivorans_WP_047894899.1:4-456    ...................KKILLNAFNMNCV.GHINHGLWTHPRD.RSQD.YRKLSYWTDLA
Pseudomonas_paralactis_WP_057702387.1:4-450         ...................KKILLNAFNMNCI.GHINHGLWTHPRD.NSTE.YKTLQYWTELA
Pseudomonas_taetrolens_WP_048381143.1:4-453         ...................KKILFNAFNMNCI.GHINHGLWTHPRD.TSTR.FNTLEYWTELA
Pseudomonas_psychrophila_WP_123750761.1:4-450       ...................KKILLNAFNMNCI.GHINHGLWTHPRD.TSTR.YNTLEYWTELA
Collimonas_arenae_100_WP_061532973.1:4-454          ...................KQIRLNAFNMNCV.GHINHGLWTHPRD.RSTD.YTSLEYWTDQA
Pseudomonas_viridiflava_WP_122210720.1:4-451        ...................KKILLNAFNMNCI.GHINHGLWTHPRD.TSTQ.FNRLEYWTDLA
Kocuria_varians_WP_068470402.1:1-454                .................MSKRISVNAFDMTCV.DHQSFGLWRHPRS.RATE.YNTLEYWTNLA
Pseudomonas_wadenswilerensis_WP_115084816.1:4-455   ...................KKILLNAFNMNCV.GHINHGLWTHPRD.NSSQ.YKTLEYWTELA
Pseudomonas_syringae_WP_011266344.1:4-452           ...................KKILLNAFNMNCI.GHINHGLWTHPRD.TSTQ.FNSLEYWTELA
Pseudomonas_sp._286_105WP_122415793.1:4-453         ...................KKILLNAFNMNCI.GHINHGLWTHPRD.TSTQ.FNSLEYWTDLA
Geobacillus_vulcani_WP_031406843.1:1-461            .................MDQPLLFNAFEMASA.MHNAHGLWKHPDS.ERHRRYKELHYWIEMA
Geobacillus_icigianus_WP_033018365.1:1-461          .................MDQPLLFNAFEMASA.MHNAHGLWKHPDS.ERHRRYKELHYWIEMA
Pseudomonas_floridensis_WP_083182568.1:4-453        ...................KKILLNAFNMNCI.GHINHGLWTHPRD.TSTQ.FNSLEYWTDLA
Pseudomonas_asturiensis_WP_073167523.1:4-451        ...................KKILLNAFNMNCI.GHINHGLWTHPRD.TSTQ.FNSLEYWTDLA
Geobacillus_vulcani_110_WP_031406843.1:1-461        .................MDQPLLFNAFEMASA.MHNAHGLWKHPDS.ERHRRYKELHYWIEMA
```

```
Pseudomonas_syringae_WP_024682626.1:4-452                ...................KKILLNAFNMNCI.GHINHGLWTHPRD.TSTQ.FNSLEYWTDLA
LadAalpha_Geobacillus_thermoleovorans_BAM76377.1         ..................................HNAHGLWKHPDS.ERHRRYKELHYWIEMA
Tsukamurella_pseudospumae_WP_068746786.1:1-457           .................MTRQIRFNAFDMNCV.AHQSPGLWRHPED.QSHR.YKELSYWTDLA
Kocuria_sp._Marseille-P3598_WP_085529073.1:5-456         ...................KRIAINAFDMTCV.GHQSFDLWRHPRS.RATE.YNTLEYWTDLA
Microvirga_aerophila_WP_114188789.1:1-456                .................MKKQIRLNAFDMNCV.GHIQHGLWTHPRD.RSTA.YNTIEYWQHLA
Burkholderia_ubonensis_WP_071760483.1:5-455              ...................KKILLNAFNMNCV.GHINHGLWTHPRD.RSAH.YTDLDYWADLA
Achromobacter_marplatensis_WP_006226823.1:4-450          ...................KQILLNAFNMNCV.GHINHGLWTHPRD.TSTQ.YKTLDYWTDLA
Acinetobacter_baumannii_WP_070165746.1:1-443             ............................MNCI.AHQSPGLWRHPQD.RSVE.YKDLEYWTDLA
Labedella_phragmitis_WP_128496094.1:1-453                .................MPKQIRFNAFDMNCV.AHQASGMWRHPDD.RSSE.YTDLAYWTSLA
Nocardia_cyriacigeorgica_120_WP_130918184.1:1-453        .................MSRRIRFNAFDMNCV.AHQSPGLWRHPED.QSHR.YKDLSYWTELA
Nocardia_cyriacigeorgica_WP_130918184.1:1-453            .................MSRRIRFNAFDMNCV.AHQSPGLWRHPED.QSHR.YKDLSYWTELA
Mycobacterium_sp._1164985.4_WP_067298521.1:1-453         .................MTRVIRFNAFDMNCV.AHQSPGLWKHPDD.QSWR.YKDLEYWTELA
Rhodococcus_sp._1R11_WP_135042739.1:1-449                .................MPREIRFNAFDMNCV.AHQSPGLWRHPLD.QSWR.YKELGYWTDLA
Erwinia_amylovora_WP_004157587.1:5-459                   ...................REIRLNAFDMNCV.GHQSPGLWAHPRD.RSWQ.YKDLSYWTDLA
Ralstonia_mannitolilytica_WP_102079134.1:4-451           ...................KRILLNAFNMNCV.GHINHGLWTHPRD.RSAD.YNTLEYWTDQA
Leifsonia_flava_WP_135120808.1:15-465                    ...................RQIRFNAFDMNCV.AHQSSGMWRHPQD.QSYR.YKEIGYWTELA
Cryobacterium_roopkundense_WP_084141147.1:1-453          .................MPRQIRFNAFDMNCV.AHQSAGLWRHPDD.QGWR.YKDLRYWTDLA
Cupriavidus_pauculus_WP_101681197.1:1-455                .................MPREIRLNAFDMHCV.GHIQQGMWTHPRD.QSHR.YAELQYWIDYA
Rhizobium_sp._BK333_130_WP_133704837.1:2-454             ...................KPMQIYAFDMNCV.GHINHGLWTHPRD.TSLS.YTDLDYWVDFA
Caballeronia_mineralivorans_WP_047846789.1:1-454         .................MSKTIHFNAFEMNCV.GHQSPGLWSHPRD.RSSQ.YKDLEYWTDLA
Nocardia_altamirensis_WP_069163049.1:1-454               .................MTRRIRFNAFDMNCV.AHQSPGLWRHPDD.QSHR.YKELSYWTDLA
Cupriavidus_sp._P-10_WP_116322758.1:1-452                .................MKKEIRINAFDMNTG.VHQSQGLWRHPRD.RSSE.YTTLRYWQDLA
Serratia_sp._S1B_WP_116726564.1:1-452                    .................MSKRISFNAFEMNCI.AHQSPGLWRHPQD.RSTE.YKDLEYWTDLA
Pseudomonas_putida_WP_043864435.1:1-452                  .................MPKQIRLNAFAMNTV.GHLSPGLWRHPRD.QATR.YTDIHYWTELA
Pseudomonas_aeruginosa_WP_023084248.1:4-453              ...................KQILLNAFSMNCV.GHINHGLWTHPRD.RSSD.YRKLRHWTELA
Pseudomonas_mucidolens_WP_084381795.1:9-455              ...................KKILLNAFNMNCI.GHINHGLWTHPRD.TSTQ.YKNLEYWVELA
Pseudomonas_poae_WP_060549803.1:4-450                    ...................KKILLNAFNMNCI.GHINHGLWTHPRD.TSTH.YKTIEYWTDLA
Pseudomonas_chlororaphis_WP_123573412.1:9-454            ...................KKILLNAFNMNCI.GHINHGLWTHPRD.TSTQ.YKTIEYWTELA
Janthinobacterium_lividum_140_WP_072454679.1:1-44        .................MAKTIRFNAFQMNSV.GHQSPGLWTHARD.RSHR.YTDPAHWTELA
Haladaptatus_cibarius_WP_049970513.1:4-446               ...................ENIHFNLFTMNAV.EHVTAGNWRTPGD.QSHR.YTDMDYWQEVA
Arthrobacter_luteolus_WP_066298473.1:1-443               ............................MTTP.THQSPGLWRHPEN.RADS.YNTLSYWTGLA
Geobacillus_sp._ZGt-1_WP_047758532.1:1-436               ...........................................PDS.ERHRRYKELHYWIEMA
Lysinibacillus_composti_WP_124766485.1:4-458             ...................RRIYLNAFDMNTP.GHQSPGLWAHPDD.RSHR.YKDSEYWIELA
Microcella_putealis_WP_130486201.1:20-474                ...................RRIRFNAFDMNCV.AHQSSGLWRHPSD.QSHR.YTELSYWTELA
Pseudomonas_nitroreducens_WP_037016976.1:1-453           .................MSKQIRLNAFAMNCV.GHLSPGLWRHPRDQQAAR.YTDIHYWIELA
Acinetobacter_wuhouensis_WP_130168784.1:1-453            .................MTKKISFNAFEMNCI.AHQSPGLWRHPAD.RSTE.YKDIEYWTDLA
Bradyrhizobium_sp._YR681_WP_008144663.1:1-454            .................MAKQIRLNAFAMNCV.AHQSPGLWTHPRD.RTAE.YNRLPYWIDLA
Schumannella_sp._10F1D-1_WP_141164655.1:10-460           ...................RQIRFNAFDMNCV.AHQSSGLWRHPDD.RSDT.YNTLEYWTELA
Pseudomonas_coronafaciens_150_WP_122355187.1:4-45        ...................KKILFNAFNMNCI.GHINHGLWTHPRD.TSTQ.FNSLEYWTDLA
Pseudomonas_syringae_WP_003430238.1:4-452                ...................KKILLNAFNMNCI.GHINHGLWTHPRD.TSTQ.FNSLEYWTDLA
unclassified_Rhodococcus_WP_094682261.1:1-449            .................MSREIRFNAFDMNCV.AHQSPGLWRHPLD.QSWR.YKELGYWTDLA
Hafnia_alvei_WP_046449330.1:6-449                        ...................KQILMNAFNMNCV.GHIHHGMWTHPED.RSTD.FNSLNYWLDLA
AS9A_3890_Hoyosella_subflava_WP_013808677.1:1-453        .................MTRQVRFNAFDMNCV.AHQSPGLWRHPAD.QSQR.YTEIEYWTGLA
Acinetobacter_baumannii_WP_000995600.1:1-443             ............................MNCI.AHQSPGLWRHPQD.RSVE.YKDLEYWTDLA
Agrobacterium_sp._B133/95_WP_065696417.1:2-454           ...................KPMQIYAFDMNCV.GHINHGLWTHPRD.LSLN.YTDLDYWVDFA
Microbacterium_sp._KROCY2_WP_024289149.1:1-453           .................MAKRISLNAFDMTCV.DHQSFGLWRHPRS.RATE.YNTIEYWTNLA
Variovorax_paradoxus_WP_012747040.1:6-452                ...................KEILLNAFNMNCV.GHINHGLWTHPRD.RSLE.YNTLEYWTSIA
Methylobacterium_radiotolerans_WP_076730229.1:5-4        ..................TKQILLNAFTMTCA.GHINHGLWTHPRD.RSAE.YNTLSYWTEQA
Streptomyces_sp._BK215_WP_132915265.1:7-452              ...................RPLVFNAFTMNCV.SFINHGLWRHPDT.LQTR.YADLDTWVELA
Geobacillus_thermodenitrificans_160_WP_008880416.        .................MTQPLLFNAFDMTSA.MHNAHGLWKHPES.ERHRRYNDLHYWIEMA
Pseudomonas_aeruginosa_WP_132556951.1:356-785            ........................................WTHPRD.RSSD.YRKLRHWTELA
Penicillium_solitum_OQE02104.1:14-471                    ...................KSLILNAFVEMCS.GHQSPGLWRHPED.ESHR.FNDVDHWVELA
Penicillium_vulpinum_OQE05502.1:12-469                   ...................KSLILNAFVEMCS.GHQSPGLWRHPED.ESYR.FNDVDHWVELA
Clavibacter_michiganensis_WP_045526194.1:1-454           .................MASRIILNAFDMSCV.THQAPGLWRHPAN.QAHR.YNSLDYWVDLA
Glutamicibacter_creatinolyticus_WP_054821484.1:6-        ...................REILFNAFDMNCV.AHQSPGLWRHPQD.QAAS.YNTLGYWTHLA
Campylobacter_sp._P0078_WP_086237522.1:4-454             ...................KQIHFNAFEMNCI.THLSPGLWRYPGD.EALR.YKDIEYWQNIA
Pseudomonas_furukawaii_WP_003454569.1:1-447              .................MAKQLLLNAFNMNCV.GHINHGLWTHPRD.RSVD.FNRLDYWTDLA
Leucobacter_triazinivorans_WP_130111548.1:1-442          ............................MNCV.AHQSSGLWRHPDD.QAWR.YTDIEYWTNLA
Aspergillus_brasiliensis_CBS_101740_OJJ68286.1:12        ...................KQLILNAFVEMCS.GHQSPGLWRHPED.ESHR.FNDIDHWTELA
Aspergillus_udagawae_170_GAO89440.1:15-472               ...................KSLILNAFVEMCS.GHQSPGLWRHPED.ESHR.FNDVDHWIELA
Aspergillus_flavus_RMZ37351.1:15-472                     ...................KSLILNAFVEMCS.GHQSPGLWRHPED.ESHR.FNDIDHWIELA
Aspergillus_flavus_RAQ54081.1:15-472                     ...................KSLILNAFVEMCS.GHQSPGLWRHPED.ESHR.FNDIDHWIELA
Penicillium_arizonense_XP_022490779.1:12-469             ...................KSLILNAFVEMCS.GHQSPGLWRHPED.ESHR.FNDLEHWTELA
Salinibacterium_sp._CGMCC                                .................VTRQIRFNAFDMNCV.AHQSSGLWRHPQD.QSRR.YNELTYWTELA
Aspergillus_fumigatus_OXN06785.1:14-471                  ...................KSLILNAFVEMCS.GHQSPGLWRHPED.ESHR.FNDVDHWIELA
Aspergillus_lentulus_GAQ10210.1:7-464                    ...................KSLILNAFVEMCS.GHQSPGLWRHPED.ESHR.FNDVDHWIELA
Aspergillus_flavus_RAQ66103.1:15-472                     ...................KSLILNAFVEMCS.GHQSPGLWRHPED.ESHR.FNDIDHWIEIA
Aspergillus_fumigatus_OXN22894.1:14-471                  ...................KSLILNAFVEMCS.GHQSPGLWRHPED.ESHR.FNDVDHWIELA
Aspergillus_nidulans                                     ...................KSLILNAFVEMCS.GHQSPGLWVHPED.ESHR.FNDIDHWIELA
Penicillium_nalgiovense_OQE79848.1:14-471                ...................KSLILNAFVEMCS.GHQSPGLWRHPED.ESHR.FNDVDHWVELA
Aspergillus_wentii                                       ...................KQIILNAFVMNTP.GHLSPGLWRHPRN.KTSQ.YKKLSFWTDLA
Gordonia_iterans_WP_105943624.1:1-453                    .................MTRQIHLNAFDMNCV.AHQSPGLWRHPDD.RSWD.YNTLDYWVHLA
Azotobacter_beijerinckii_WP_090735436.1:4-454            ...................KQILLNAFNMNCV.GHINHGLWTHPRD.RSTE.FNTLEYWTELA
Staphylococcus_sp._NAM3COL9_WP_057513692.1:4-453         ...................KQIHFNGFVQNSP.SPHSSGLWKHDKD.QGAH.HNQLEYWINIA
Acidovorax_sp._56_WP_099656603.1:11-450                  ...................KPIVLNAFNMNCV.GHINHGLWTHPRD.RSTE.YNTLPYWTDLA
Paenibacillus_sp._BIHB4019_WP_099518466.1:1-456          .................MKKKIHLGVFEVNAV.NHLTQGIWTHPEQ.NRIH.YNSMDYWIDIA
Penicillium_antarcticum_OQD90033.1:5-465                 ...................KQLIINAFAMQSP.SHLNPGLFRYPTD.QGPD.YKNIKHWIALA
Aspergillus_oryzae_RIB40_190_XP_001823703.1:9-463        ...................KQIILNAFVMNTP.GHLAPGLWKHPRN.KTDQ.YKKLSFWTELA
Fusarium_oxysporum_f._sp._pisi                           ...................KQLIINAFVESCS.GHQSPGLWRHPDD.KSWD.FNNISHWVKLA
Colletotrichum_fructicola_Nara_gc5_ELA35345.1:18-        ...................KQIILNAFVMNTP.GHLAPGLWKHPRN.KTDQ.YKKLSFWTELA
Micrococcus_luteus_WP_041104460.1:19-468                 ...................REILFNAFDMNCV.AHQSPGLWRHPDD.HARD.YNTLGYWTHLA
Aspergillus_awamori_GCB21923.1:12-469                    ...................KQLILNAFVEMCS.GHQSPGLWRHPED.ESHR.FNDIDHWIELA
Aspergillus_niger_CBS                                    ...................KQLILNAFVEMCS.GHQSPGLWRHPED.ESHR.FNDIGHWIELA
Aspergillus_lacticoffeatus_CBS_101883_XP_02545389        ...................KQLILNAFVEMCS.GHQSPGLWRHPED.ESHR.FNDIDHWIELA
Aspergillus_wentii_DTO_134E9_OJJ40237.1:14-471           ...................KSFILNAFVEMCS.GHQSPGLWRHPED.ESHL.FNDIDHWIELA
Aspergillus_indologenus_CBS_114.80_PYI31207.1:11-        ...................KSLILNAFVEMCS.GHQSPGLWRHPED.ESHR.FNDIEHWTELA
Aspergillus_fischeri_NRRL_181_XP_001264002.1:14-4        ...................KSLILNAFVEMCS.GHQSPGLWRHPED.ESHR.FNDVDHWIELA
Penicillium_coprophilum_200_OQE46388.1:11-466            ...................KQIILNAFVMNTP.GHLAPGLWKHPRN.KTDQ.YKSLSFWTDLA
Cupriavidus_taiwanensis_WP_116379876.1:8-454             ...................KQILLNAFNMNCV.GHINHGLWTHPRD.RSAE.YNTLEYWVDQA
Penicillium_digitatum                                    ...................KQLIINAFAMQSP.SHLNPGLFRHPTD.QGAD.YKNIKHWIALA
Aspergillus_niger_GAQ46262.1:12-469                      ...................KQLILNAFVEMCS.GHQSPGLWRHPED.ESHR.FNDIDHWIELA
Aspergillus_nomius_NRRL_13137_XP_015412385.1:15-4        ...................KSLILNAFVEMCS.GHQSPGLWRHPED.ESYR.FNDIDHWIELA
Aspergillus_aculeatus_ATCC_16872_XP_020055010.1:1        ...................KSLILNAFVEMCS.GHQSPGLWRHPED.ESHR.FNDIEHWTELA
Penicillium_brasilianum_OOQ84969.1:19-474                ...................KQFILNAFVMNTP.GHLAPGLWKHPRN.KTDQ.YKKLSFWTDLA
Penicillium_subrubescens_OKP15100.1:19-474               ...................KQIILNAFVMNTP.GHLAPGLWKHPGN.KTDQ.YKKLSFWTDLA
Trichoderma_arundinaceum_RFU80530.1:19-474               ...................KQIILNAFVMNTP.GHLSPGFWRHPRN.KTDQ.YTKLSFWTDLA
Aspergillus_thermomutatus_210_XP_026614594.1:15-4        ...................KPLILNAFVEMCS.GHQSPGLWRHPED.ESHR.FNDVDHWIELA
Serratia_liquefaciens_WP_044551273.1:4-449               .................IAKEVLLNAFNMNCV.GHIHHGMWTHPQD.RSTE.FNQLGYWTELA
Micrococcus_luteus_WP_065572847.1:19-468                 ...................REILFNAFDMNCV.AHQSPGLWRHPDD.HARD.YNTLGYWTHLA
Halolamina_rubra_WP_049979911.1:1-449                    .................MSDRLHLNLFTMNAV.EHVSPGTWRRDGD.RSGE.YTDREYWTDVA
Acinetobacter_pittii_WP_075382847.1:7-452                ...................KEILLNAFDMNCV.GHINHGLWTHPRD.ESHR.FNELSYWTEQA
Corynebacterium_variabile_WP_052302534.1:2-464           ..................TRIRFNAFDMNCV.VHQSPGLWRHPED.HSRD.YTDLRYWTDLA
Aspergillus_luchuensis_GAT21254.1:12-471                 ...................KQLILNAFVEMCS.GHQSPGLWRHPED.ESHR.FNDIDHWIELA
```

```
Aspergillus_luchuensis                                  ..................KQLILNAFVEMCS.GHQSPGLWRHPED.ESHR.FNDIDHWIELA
Aspergillus_bombycis_XP_022386026.1:15-472              ..................KSLILNAFVEMCS.GHQSPGLWRHPED.ESHR.FNDIDHWIELA
Agrobacterium_vitis_WP_070163524.1:1-454                ................MTRTIHFNAFDMNCV.GHQSPGLWAHPDD.RSYT.YKDLDYWQNLA
Pseudomonas_psychrotolerans_220_WP_074584491.1:5-       .................AKQVLLNAFNMNTV.GHINHGLWTHPRD.TSSR.YHTLGYWTELA
Micrococcus_yunnanensis_WP_135040276.1:19-468           ..................REILFNAFDMNCV.AHQSPGLWRHPDD.HARD.YNTLGYWTHLA
Acinetobacter_calcoaceticus_WP_004642549.1:7-452        ..................KEILLNAFDMNCV.GHINHGLWTHPRD.ESHR.FNELSYWTEQA
Penicillium_brasilianum_CEO59775.1:15-472               ..................KSLILNAFVEMCS.GHQSPGLWRHPDD.ESHR.FNDIEHWVELA
Aspergillus_cristatus_ODM22551.1:12-469                 ..................KSLILNAFVEMCS.GHQSPGLWQHPED.ESWR.FNDVDHWVELA
Pseudomonas_taiwanensis_WP_027907363.1:1-446            ................MPRPIRFNAFSMNAA.SHQSPGLWRHPRN.TSVA.FNRLDYWTDLA
Acidovorax_avenae                                       ..................RPIILNAFSMNTV.GHINHGLWTHPRD.RSGE.YHSLDYWTGLA
Staphylococcus_pseudintermedius_WP_101431388.1:4-       ..................KQIHFNGFVQNSP.APHAAGLWKHPKD.KGAT.HHNLKYWTNIA
Rhodococcus_triatomae_WP_081607437.1:2-451              ..................SRIHLHAFKIAGV.GHTAIGSWRNPYD.QQHR.YTDLNYWLDTA
Alcaligenes_sp._RS4_230_WP_128393375.1:4-450            ..................KKILLNAFNMNSI.GHIHHGMWTHPRD.QSDR.YNTIEYWTHIA
Gibbsiella_quercinecans_WP_121552947.1:7-454            ..................KEILLNAFNMNCV.GHIHHGMWTHPQD.RSVE.FNRLEYWLDLA
Acinetobacter_ursingii_WP_044435211.1:5-452             ................LPKKILLNAFDMNSV.GHINHGLWTHPRD.QSHR.FNELSYWTDLA
Erwinia_typographi_WP_034887712.1:4-447                 ..................KQILLNAFNMNCV.GHIHHGMWTHPQD.RSVD.FNSLSYWTGLA
Trichoderma_harzianum_KKP01913.1:14-470                 ..................KKLILNAFVESCS.GHQSPGLWRHPQD.RSAN.FNSIHHWVELA
Fusarium_graminearum_235_CEF79766.1:12-466              ..................KSLILNAFVEMCS.GHQSPGLWRHPDD.QSWR.FTDIEHWTDLA
Gordonia_alkanivorans_WP_006357654.1:10-461             .................TRKVRFNAFDMNCV.AHQSPGLWRHPSD.RSWN.YNTIDYWVDLA
Acinetobacter_radioresistens_WP_111281495.1:5-455       ................LPKQILLNAFDMNSA.GHINHGLWTHPRD.QSHR.FNELNYWTDLA
Mesorhizobium_plurifarium_WP_073985566.1:5-458          ..................KLIRLNGFKQSTV.SHAAVGAWRHPDN.QSHR.YRELDYWIKTA
Oceanobacillus_halophilus_WP_121203049.1:1-454          ................MAKQIHLNGFVQNSP.SPHSTGLWKHKKD.EGIK.HNQISYWTETA
Acinetobacter_tandoii_240_WP_100241535.1:7-457          ..................KQILLNAFDMNSV.GHINHGLWTHPRD.QSHR.FNELNYWTELA
Burkholderia_plantarii_WP_042624083.1:1-457             ................MPRQIRLNAFMMNCV.AHLAPGQWTAPDD.GSAD.YLDPAYWVGIA
Alcaligenes_faecalis_WP_094195208.1:6-457               ..................KHIHINAFNMNCV.GHIHHGLWTHPRD.QSTQ.YHTLKYWTDLA
Bradyrhizobium_arachidis_WP_092214516.1:1-453           ................MSKLIQVNGFAIFSP.VHLSPGLWRHPDD.RSLD.FSTLDYWTDVA
Cupriavidus_plantarum_WP_109583068.1:1-458              ................MSRQIRLNAFTMNCV.VHLAPGQWTAESD.RSTG.YLDTGHWVELA
Variovorax_sp._770b2_245_WP_093443018.1:5-461           ..................RPILLNAFDMNTV.GHISHGLWRHPRD.RSID.YKRMPYWLDLA
RZJ22128.1:4-447                                        ..................RQIKLGAFIPATA.QHAAG..WRHPES.RPQD.HLNLDYIIELA
LadB_Geobacillus_thermoleovorans_BAM76371.1             ..........MVEFITMAPTSGDSTFVGLGS.NSTNLNSWTGTDE.NPER.PPTVEYIEAIA
LadAbeta_Geobacillus_thermoleovorans_BAM76372.1         ...............MSVRQMKFGLFLMGVG.HHIAA..WRHPDV.RPEW.CEDFTFFRRIA
Af1_AFLA_024140_XP_002373925.1                          ....MTVTNDPSPPNGPRKSLILNAFVEMCS.GHQSPGLWRHPED.ESHR.FNDIDHWIELA
Af3_AFLA_58870_XP_002378631.1                           ..........MGSIDKPKKQLILNAFAMQSP.SHLNPGLHRYPKD.QGGA.YKSLQHWVTLA
Af4_AFLA_049780_XP_002383105.1                          MGDISTQPDPSDANGTPKKHILLNAFDMSTV.GHLSPGQWKNPAD.KSAT.KRSLTYWIELA
Af5_AFLA_126060_XP_002381799.1                          ...........MTKTSEKKSWILNAFAMFSP.GHLSPGLWKNPQD.RAGD.FLDLSYWIELA
Af2_QMW32876.1:9-463                                    ..................KQIILNAFVMNTP.GHLAPGLWKHPRN.KTDQ.YKKLSFWTELA
Yarrowia_lipolytica_CLIB122_YALI0C07546p_XP_50156       .................TKKIILNAFVMFTP.THLNFGLWTHPDHSKFGRDFAKRQPWQELA
Rhizobium_sp._CF122_WP007794079.1                       ...................QIKLGAFLYPTG.HHVAA..WRHPEA.QADA.GSNFAHYIELA
Acinetobacter_sp._NIPH_2100_LLM                         ................MSRKIRLGAFIPATG.QHVAA..WRHPES.QPTK.AVDFDFIKEQV
Nostoc_sp._PCC_7107_WP_015115963.1                      .................TRKFRLGAFIQATG.HHVSA..WRHPDA.QIDA.GFNFEHYKEIT
Aspergillus_flavus_NRRL3357_uncharacterized             ...............MPEKTLHLTAFMRPVS...LHTGAWRYPGA.YPDA.NFNLTHLKSFI
Paenibacillus_curdlanolyticus_WP006037998.1             ....................LRLGAFMMIPG.HHVAA..WRHPEA.EAKS.VLDFKFYKQLA
LadAbeta_Geobacillus_thermoleovorans_FMN-dependen       .................VRQMKFGLFLMGVG.HHIAA..WRHPDV.RPEW.CEDFTFFRRIA
SsuD_1M41                                               .....................SLNMFWFLPT...HGDGHYLGTEE..GSR.PVDHGYLQQIA
Paenibacillus_curdlanolyticus_WP006037998.1             ....................LRLGAFMMIPG.HHVAA..WRHPEA.EAKS.VLDFKFYKQLA
Bacillus                                                ...................................................Y...SYFRQVA
Pseudomonas                                             ..........................................................Q...
LadB_Geobacillus_thermoleovorans_FMN-dependent          ..............................................................
Pseudomonas_aeruginosa_PAO1_NP_251290.1                 .....................MITATPSSEV.............D.QPLGEAVDAGFVRRFA
Alkanesulfonate                                         ..............................................................
Alkanesulfonate                                         ..................MPVEFISLTF....PNASTEIKPIPNA......TIDPEYLERYA
Aspergillus_niger_ATCC13496_RDH19503.1                  ................MPTEFISLTF......PNASTEVAPIPNA......RIDPDYLVRYA
Penicillium_digitatum_Pd1_XP014534980.1                 ..................MAEFISVTFPNAS.TEINP.........IAKA.SIDPEYLVRYA
Acinetobacter_rudis_WP_016657872.1                      ...................................MNIFWFLPTH.GDGH.FLGTSEN.ARP
```

```
                                                    50        60        70         80        90        100
LADA_Geobacillus_thermodenitrificans_WP_011888513  QLLEKGKFDALFLADVVGIYDVYRQSRDT..AVREAV..QIPVNDPLMLISAMAYVTKHLAF
Geobacillus_sp._LEMMY01_WP_079935807.1:1-440       QLLEKGKFDALFLADVVGIYDVYRQSRDT..AVREAV..QIPVNDPLMLISAMAYVTKHLAF
Geobacillus_stearothermophilus_ABV66256.1:1-440    QLLEKGKFDALFLADVVGIYDVYRQSRDT..AVREAV..QIPVNDPLMLISAMAYVTKHLAF
Geobacillus_sp._B4113_201601_WP_061912600.1:1-440  QLLENGKFDALFLADVVGIYDVYKQSRDT..AVREAV..QIPVNDPFMLVSAMAYVTKHLAF
Geobacillus_sp._B4113_201601_5WP_061912600.1:1-44  QLLENGKFDALFLADVVGIYDVYKQSRDT..AVREAV..QIPVNDPFMLVSAMAYVTKHLAF
Bacillus_thuringiensis_6WP_029441578.1:1-435       QLLEKGMFDSLFLADVVGVYDTYQQNKDT..AVREAV..QIPANDPLMIISAMANVTKHLGF
Lysinibacillus_sphaericus_WP_103976887.1:2-422     KTLEKGKFDSLFLADVVGVYDVYKGGFDT..SVKEAV..QIPANDPLIPISAMAHVTTNLCF
Lysinibacillus_sphaericus_WP_069508309.1:2-422     KTLEKGKFDSLFLADVVGVYDVYKGGFDT..SVKEAV..QIPANDPLIPISAMAHVTTNLCF
Rummeliibacillus_sp._SL167_10_WP_146551437.1:2-42  KTLEKGRFDALFLADVVGLYDVYKGGLDT..SIKEAV..QIPANDPFIPISAMAHVTSNLSF
Paenibacillus_sp._DXL2_WP_110843609.1:1-459        QLLERGKFDALFLADVVGVYDVYKQGDET..ALRESV..QLPCNDPFFVVPPMALVTKHLGF
Paenibacillus_naphthalenovorans_WP_074730028.1:1-  QLLERGKFDALFLADVVGVYDVYKQGDET..ALRESV..QIPCNDPFFVVPPMALVTKHLGF
Cohnella_sp._HS21_WP_130606586.1:1-453             KLLERGKFDALFLADVLGFYDIFGGNRDA..ALRDAV..QAPVNDPSLIIPTMAYATENLSF
Streptosporangium_subroseum_15_WP_089205784.1:1-4  TLLERGLFDAVFLADVVGAYDRYRGGPET..AISEAV..QIPANDPLLVVPAMAAVTKHLGF
Arthrobacter_sp._Hz2_WP_120147654.1:1-455          KLLEAGLFDSVFLADVIGTYDGYRGGPDT..ALREAV..QIPSNDPLLIIPAMAAVTRHLGF
Nonomuraea_solani_WP_103963668.1:1-453             KLLERGLFDAVFLADVVGAYDRFRGGPET..AIREAV..QIPANDPLLVVPAMAAVTRHLGF
Arthrobacter_ruber_WP_105030338.1:1-455            KLLEAGLFDSVFLADVIGTYDGYRGGPGT..ALREAV..QIPSNDPLLVIPAMAAVTRHLGF
Bacillus_aciditolerans_20_WP_121446947.1:1-456     KLLERGKFDAVFFADVLGIYDVYKQSKHP..SIRDGV..QVPLNDPAFVVPAMASVTEHLSF
WP_137163763.1:1-458                               RLLERGLFDALFIADVLGIYDVYQGGPEA..ALRGGV..QVPVNDPLLLVPAMAGVTEHLGF
Aeribacillus_pallidus_WP_130157503.1:4-457         QLLEKGRFDGLFIADVIGIYDVYGNDKTT..AVREAT..QVPVNDPILLVSAMAHATEHLGF
Bacillus_megaterium_24WP_098602848.1:4-460        QILEKGRFDGLFIADVIGIYDVYGGDKTV..TVREAT..QVPVNDPMMLVSAMARETEHLGF
Streptomyces_sp._150FB_WP_078877664.1:5-455        QLLEYGTFDAVFLADVIGAYDGFRGGPAT..ALTEAV..QIPNNDPLLLIPAMAAVTKGLGF
Arthrobacter_sp._DWC3_WP_104051071.1:1-454         KLLEAGLFDSVFLADVIGTYDGYRGGPET..ALREAV..QIPSNDPLLVIPAMAAATRHLGF
Cohnella_thermotolerans_WP_027093898.1:1-456       RLLERGKFDAIFFADVIGVYDVYKGSRDP..AIREGV..QVPLNDPAFAIPIMAQATKHLSF
Paenibacillus_bovis_28WP_144921429.1:1-452         KILEKGRFDAVFLADVLGTYDVYNGSRDA..AVRQGA..QSPVNDPLLVVPLMSSVTKHLGF
Bacillus_aciditolerans_WP_121447760.1:1-452        KLLEKGRFDAIFLADVLGPYDVYQGSRDA..AIRQGT..QTPVNDPSLVVPLMSAVTKHLGF
Leifsonia_sp._ALI-44-B_30_WP_077053645.1:1-455     KLLEEGLFEAVFLADVIGTYDGYRDGPET..ALKEAV..QIPNNDPLLVIPAMAAATKNLGF
Parageobacillus_thermoglucosidasius_WP_064552269.  KLLERGKFDAVFFADVLGVYDTYRQSKAP..SIRDGL..QFPVNDAALIIPIMASVTKHLSF
Geobacillus_sp._Y4.1MC1_WP_013399886.1:1-459       KLLERGKFDAVFFADVLGVYDTYRQSKAP..SIRDGL..QFPVNDASLVIPVMASVTKHLSF
Bacillus_methanolicus_35WP_004434428.1:1-462       KLLERGKFDAVFFADVLGVYDTYKQSKEP..SIRDGM..QIPLIDAALVIPVMASVTKHLSF
Terribacillus_halophilus_WP_077309754.1:1-448      QILEKGQFDTLFLADVLGTYDVYQSSREG..AVRQAA..QVPVNDPLLVVPAMAQVTKHLGF
Paraburkholderia_kururiensis_WP_017777532.1:1-453  QLLERGLFDGLFIADVLGIYDVYQGSGEA..AIRQAT..QVPVNDPILLVSAMAHVTQHLGF
Gracilibacillus_massiliensis_WP_058308419.1:1-456  KLLERGKFDAVFFADVLGIYDVYQKSKAP..SIRDGL..QVPLNDPALLVSAMASVTKHLAF
Streptomyces_yanglinensis_WP_103888551.1:5-452     RLLEYGTFDAVFLADVIGTYDGFRGGPQT..ALTEAV..QIPNNDPLLVIPAMAAVTRGLGF
Trinickia_sp._7GSK02_40_WP_136896361.1:1-452       KLLERGKFDGLFIADVLGIYDVYQGSGEA..AIRQAA..QVPVNDPILLVPAMANVTEHLGF
Cryobacterium_sp._NEAU-85_WP_123045138.1:1-448     RLLERGLFDAIFLADVVGTYNVYGGSRDP..AIARGI..QSPNNDPFLLVPAMAAVTEHLGF
Alicyclobacillus_acidoterrestris_WP_021298468.1:4  RLLERGCFDAVFLADVLGVYDVYQGSRDA..AVRDAA..QVPVNDPMLVVPCMAQVTKHLGF
Oceanobacillus_senegalensis_WP_085994229.1:1-452   QVLERGRFDGVFLADVLGTYDVYQNSRDA..AVTQGA..QVPINDPTYVIPLMANVTKHLGF
Bacillus_sinesaloumensis_WP_077620983.1:1-449      KLLERGKFDAVFFADVLGIYDVYQKSKAP..SIRDGL..QVPLNDPALLISAMAHVTENLSF
Halopenitus_persicus_WP_021074147.1:6-457          QTLEEGKFDAVFIADVLGTYSVFDGSRDT..AVREAV..QTPVNDPLLPIPAMAQVTDHLGF
Microbacteriaceae_bacterium                        KLLERGLFDALFLADVVGTYTVYQGSRDP..AVERGI..QTPNNDPFLLVPAMAAVTENLGF
Lysinibacillus_sinduriensis_WP_036201100.1:1-458   KLLEKGKFDSIFFADVAGVYDVYQNSKDP..AVRDGV..QVPMNDPAFVVPAMAAVTEHLSF
Bacillus_endophyticus_WP_113749053.1:5-457         QLLEKGRFDGLFIADVIGIYDVYGADKTV..TVREAT..QVPVNDPMMLVSAMAHATEHLGF
Pseudomonas_monteilii_WP_119371117.1:1-455         KLLERGKFDGIFIADVIGIYDVLGGNGDA..AIRQAA..QVPVNDPLALITPMALVTEHLAF
Bacillus_endophyticus_50_WP_061801645.1:5-457      QLLEKGRFDGLFIADVIGIYDVYGADKTV..TVREAT..QVPVNDPMMLVSAMAHATEHLGF
Planococcus_maitriensis_WP_112233285.1:1-457       KLLEKGKFDAVFLADVLGTYDVYGGTRDA..ALRNAV..QTPVNDPLLVVPLMSSVTKHLSF
Oceanobacillus_profundus_WP_118889880.1:1-447      KTLENGRFDAVFFADVLGTYDVYGESRDA..AVRNGV..QSPVNDPFLIVPYMAAVTKHLGF
Burkholderia_pseudomallei_WP_141404579.1:1-452     RLLERGKFDGLFIADVLGIYDVYQGSGEA..AIRQGA..QVPVNDPILLVSAMAAATRHLGF
Terribacillus_saccharophilus_WP_095261886.1:1-455  KLLERGKFDAIFFADVLGIYDIYRNSKAP..SIRDGL..QVPLNDPALLISAMASVTENLGF
Sediminibacillus_albus_WP_093212831.1:4-453        QLLERGNFDAVFLADVLGTYDVYQGSRDA..AVRQGA..QSPVNDPLLVVPLMSAVTSHLGF
Sciscionella_marina_WP_020496929.1:1-448           RLLERGLFDALFLADVVGTYSVYEGSRDS..AIARGI..QSPNNDPFLVVPAMAAVTENLGF
Mycetocola_saprophilus_WP_043321267.1:1-452        QLLERGLFDAMFIADVVGTYNVYRGSRDA..AVERGI..QIPNNDPYLIVPAMAAATRDLGF
Lysinibacillus_macroides_WP_053993569.1:4-460      KTLEKGRFDGLFIADVIGIYDVYGNDKET..TIREAT..QVPVNDPILLVSAMSHATKHLGF
Pseudomonas_putida_WP_086978777.1:1-458            KLLERGKFDGIFIADVIGIYDVLGGNGDA..AIRQAA..QVPVNDPLAVITPMALVTEHLGF
Sediminibacillus_halophilus_60_WP_074598704.1:4-4  KLLERGNFDAVFLADVLGTYDVYEDSRDA..AVRQGA..QSPVNDPLLVVPLMSAVTKHLGF
Rhizobium_oryzae_WP_085420425.1:1-450              KRLEAGLFDGIFLADVVGINDVYGGGNAA..ALRGAV..QVPVNDPMMLVPAMAAVTEHLSF
Pseudomonas_extremorientalis_WP_071491956.1:1-455  RILERGKFDGLFIADVLGIYDVYNGNGDA..AIRQAT..QVPVNDPLSLIAPMALVTEHLGF
Pseudomonas_fluorescens_WP_016977452.1:1-454       KILERGKFDGLFIADVLGIYDVYNGNGDA..AIRQAA..QVPVNDPLSLIAPMALVTEHLGF
Neorhizobium_sp._T25_27_WP_105419890.1:1-450       KRLEAGLFDGIFLADVLGVNDVFGGSNAA..ALRGAV..QVPVNDPLLLVPAMAAVTEHLSF
Pseudomonas_extremaustralis_WP_078833627.1:1-457   KILERGKFDGLFIADVLGIYDVYNGNGDA..AIRQAT..QVPVNDPLSLIAPMALVTEHLGF
Fictibacillus_aquaticus_WP_094253544.1:1-455       RLLERGKFDAVFFADVLGVYDTYKQSKEP..SIHDGL..QIPVNDGAFVIPTMASVTEHLSF
Pseudomonas_extremaustralis_KAA8559760.1:1-456     KILERGKFDGLFIADVLGIYDVYNGNGDA..AIRQAT..QVPVNDPLSLIAPMALVTEHLGF
Mycobacterium_sp._ACS1612_WP_067812332.1:1-454     KLLERGLFDGLFIADVLGIYDVYGGGPEA..ALRAGT..QVPVNDPLLIVSAMAAATKHLGF
Bacillus_megaterium_WP_116075167.1:1-455           KLLEKGKFDGLFLADVLGIYDVYRQSNEG..ALKQAA..QLPVNEPTALISAMAYATKHLGF
Pseudomonas_frederiksbergensis_70_WP_123409596.1:  KILERGKFDGLFIADVLGIYDVYNGNGDA..AIRQAA..QVPVNDPLQLIPPMALVTEHLGF
Pseudomonas_citronellolis_SFC61163.1:1-452         QLLEHGTFDGVFLADVVGVYDRFRGGPET..ALREAV..QVPSNDPLLVIPAMAAVTRNLGF
Herbaspirillum_chlorophenolicum_WP_050468599.1:4-  KLLERGKFDGLFLADIVGVYDVYQQSVDI..TLRESI..QLPVNDPLLLISAMAVVTEHLGF
Arthrobacter_crystallopoietes_WP_074700854.1:1-45  QLLERGLFDAIFLADVVGTYDVYRGSRGP..AVERGL..QVPNNDPYLLVPAMAAVTRNLGF
Terribacillus_goriensis_WP_038558686.1:1-438       RLLEKGKFDAVFFADVLGVYDAYGGDEKA..ALRDGV..QVPLNDPAFAIPAMAAVTKHLSF
Bacillus_megaterium_WP_025749753.1:1-455           KLLEKGKFDGLFLADVLGIYDVYRQSNEG..ALKQAA..QLPVNEPTALISAMAYATKHLGF
Neorhizobium_sp._T7_12_WP_105403044.1:1-451        KRLEAGLFDGIFLADVLGVNDVFGGSNAA..ALRGAV..QVPVNDPLLLVPAMAAVTEHLSF
Rummeliibacillus_sp._SL167_WP_146553619.1:4-459    KLLEKGKIDGLFIADVIGLYDVYQNSREA..TVREGT..QVPVNDPAMLISAMAYVTEHLGF
Burkholderia_sp._PAMC                              KLLEKGLFDGLFIADIVGVYDVYQQSADL..TLKESV..QLPVNDPMMLVSAMAAVTRNLGF
Pseudomonas_koreensis_WP_064585463.1:4-449         QLLERGLFDGLFIADIVGVYDVYQNSVDV..TLKESI..QLPVNDPLLLVSAMAAVTKNLGF
Pseudomonas_fluorescens_80_WP_064116961.1:6-451    QLLERGLFDGLFIADIVGVYDVYQNSVDV..PLKESI..QLPVNDPLLLVSAMAAVTKNLGF
Pseudomonas_rhodesiae_WP_040265276.1:4-450         QTLERGLFDGLFIADIVGVYDVYQQSVEV..PLKESI..QLPVNDPLLLVSAMAAVTRHLGF
Collimonas_sp._OK412_WP_092393355.1:4-454          QLLERGKFDGLFLADIVGVYDVYQGSADL..TLRESI..QLPVNDPLLLVSAMAAVTRHLGF
Pseudomonas_marginalis_WP_058415544.1:1-456        KILERGKFDGLFIADVLGIYDVYNGNGDA..AIRQAT..QVPVNDPLSLIAPMAMVTQHLGF
Pseudomonas_antarctica_WP_064452947.1:1-458        KILERGKFDGLFIADVLGIYDVYNGNGDA..AIRQAA..QVPVNDPLSLIAPMALVTEHLGF
Pseudomonas_protegens_WP_041115159.1:9-454         QLLERGLFDGLFIADIVGVYDVYQNSVDV..PLKESI..QLPVNDPLLLVSAMAAVTKNLGF
Pseudomonas_synxantha_WP_057024776.1:4-449         QILERGLFDGLFIADIVGVYDVYQNSIDV..PLKESI..QLPVNDPLLLVSAMAAVTRNLGF
Rhizobium_leguminosarum_WP_128409501.1:2-451       KTAERGKLDGIFLADIVGVYDVYKGSPAP..VIETAA..QIPVNDPLIPISAMAYVTKHLGF
Pseudomonas_chlororaphis_WP_038359779.1:6-451      QLLERGLFDGLFIADIVGVYDVYQNSVDV..PLKESI..QLPVNDPLLLVSAMAAVTKNLGF
Pseudomonas_syringae_90_WP_024642725.1:1-456       KILERGKFDGLFIADVLGIYDVYRGNGEA..AIRQAT..QVPVNDPLQLIPPMALVTEHLGF
Collimonas_fungivorans_WP_061540754.1:4-453        RLLERGKFDGLFLADIVGVYDVYQGSADL..TLRESI..QLPVNDPLLLVSAMAAVTKNLGF
Rhizobium_leguminosarum_WP_116409027.1:2-451       RTAEQGKLDGIFLADIVGVYDVYKGSPAP..VIETAA..QIPVNDPLIPISAMAYVTRHLGF
Microvirga_ossetica_WP_099511173.1:1-454           KIAERGKLDGIFLADIVGVYDVYKGGPET..AIAAGA..QVPVNDPMLLVPAMAYATKHLGF
Pseudomonas_caspiana_WP_087268376.1:4-451          QLLERGLFDGLFIADIVGVYDVYQNSVDV..TLKESI..QLPVNDPLLLVSAMAAVTKNLGF
Pseudomonas_lundensis_WP_048375063.1:4-451         QLLERGLFDGLFIADIVGVYDVYQQSVDV..PLKESI..QLPVNDPLLLVSAMAAVTRHLGF
Caballeronia_mineralivorans_WP_047894899.1:4-456   KLLERGLFDGLFIADIVGAYDVYQQSVDV..TLKESV..QLPVNDPLMLVSAMAAVTQHLGF
Pseudomonas_paralactis_WP_057702387.1:4-450        QILERGLFDGLFIADIVGVYDVYQNSIDV..PLKESI..QLPVNDPLLLVSAMAAVTRHLGF
Pseudomonas_taetrolens_WP_048381143.1:4-453        QLLERGLFDGLFIADIVGVYDVYQQSVDV..PLKESI..QLPVNDPLLLVSAMAAVTKNLGF
Pseudomonas_psychrophila_WP_123750761.1:4-450      QLLERGLFDGLFIADIVGVYDVYQQSVDV..PLKESI..QLPVNDPLLLVSAMAAVTKNLGF
Collimonas_arenae_100_WP_061532973.1:4-454         KLLERGKFDGLFLADIVGVYDVYQGSADL..TLRESI..QLPVNDPLLLVSAMAGVTRHLGF
Pseudomonas_viridiflava_WP_122210720.1:4-451       KLLERGLFDGLFIADIVGVYDVYRNSVDV..TLKEAI..QLPVNDPLLLVSAMAGVTRHLGF
Kocuria_varians_WP_068470402.1:1-454               RLLEKGKFDALFLADVVGIYDIYRNSAAP..AVRDGA..QVPVNDPFMQVSAMAAVTEHLGF
Pseudomonas_wadenswilerensis_WP_115084816.1:4-455  QLLERGLFDGLFIADIVGVYDVYQQSVDV..PLKESI..QLPVNDPLLLVSAMAAVTRNLGF
Pseudomonas_syringae_WP_011266344.1:4-452          KLLERGLFDGLFIADIVGVYDVYQNSVDV..TLKEAI..QLPVNDPLLLVSAMAGVTRHLGF
Pseudomonas_sp._286_105WP_122415793.1:4-453        KLLERGLFDGLFIADIVGVYDIYQNSVDV..TLKEAI..QLPVNDPLLLVSAMAGVTKHLGF
Geobacillus_vulcani_WP_031406843.1:1-461           KRLEAGLFDAVFFADVLGVYDTYRQSKDP..SIRDGM..QFPLNDASLIIPVMASVTTHLSF
Geobacillus_icigianus_WP_033018365.1:1-461         KRLEAGLFDAVFFADVLGVYDTYRQSKDP..SIRDGM..QFPLNDASLIIPVMASVTTHLSF
Pseudomonas_floridensis_WP_083182568.1:4-453       KLLERGLFDGLFIADIVGVYDIYQNSVDV..TLKEAI..QLPVNDPLLLVSAMAGVTKHLGF
Pseudomonas_asturiensis_WP_073167523.1:4-451       KLLERGLFDGLFIADIVGVYDIYQNSVDV..TLKEAI..QLPVNDPLLLVSAMAGVTRHLGF
Geobacillus_vulcani_110_WP_031406843.1:1-461       KRLEAGLFDAVFFADVLGVYDTYRQSKDP..SIRDGM..QFPLNDASLIIPVMASVTTHLSF
```

```
Pseudomonas_syringae_WP_024682626.1:4-452            KLLERGLFDGLFIADIVGVYDVYQNSVDV..TLKEAI..QLPVNDPLLLVSAMAGVTRHLGF
LadAalpha_Geobacillus_thermoleovorans_BAM76377.1     KRLEAGLFDAVFFADVLGVYDTYRQSKDP..SIRDGM..QFPLNDASLIIPVMASVTTHLSF
Tsukamurella_pseudospumae_WP_068746786.1:1-457       KLLERGTFDGIFIADVLGIYDAYNGNGDA..AIKHAA..QVPVSDPILLASAIAGATEHLGI
Kocuria_sp._Marseille-P3598_WP_085529073.1:5-456     KTLEKGLFDGVFLADVVGIYDIYRNSAAP..SIRGGA..QVPVNDPFMQISAMAAVTEHLGF
Microvirga_aerophila_WP_114188789.1:1-456            RTAERGLFDGIFLADIVGVYDVYKGSPAP..SLENAV..QVPVNDPLLVVPVMAAVTKHIGF
Burkholderia_ubonensis_WP_071760483.1:5-455          KTLERGKFDGIFLADIVGVYDVFGGGPDA..ALRESV..QVPVNDPLLIVPAMAQVTRHLGF
Achromobacter_marplatensis_WP_006226823.1:4-450      RLLERGLFDGLFLADIVGVYDVYQDSADL..TLRESI..QLPVNDPLLTISAMAAATRHLGF
Acinetobacter_baumannii_WP_070165746.1:1-443         QILERGFFDGIFIADVLGIYDVYHQSAEH..ALTGAV..QVPVNDPLQIVPAMAAVTKHLGF
Labedella_phragmitis_WP_128496094.1:1-453            QLLERGGFDGIFIADVLGTYDVYGGSNEA..AIRNGA..QVPVNDPVLLVSAMAAVTEHLGF
Nocardia_cyriacigeorgica_120_WP_130918184.1:1-453    KLLERGRFDGIFIADVLGTYDVYGGDDTA..ALRQAA..QIPLADPLLLVSAMAAVTDHLGF
Nocardia_cyriacigeorgica_WP_130918184.1:1-453        KLLERGRFDGIFIADVLGTYDVYGGDDTA..ALRQAA..QIPLADPLLLVSAMAAVTDHLGF
Mycobacterium_sp._1164985.4_WP_067298521.1:1-453     TLLERGRFDGLFIADVLGTYDVYGASDEA..AIRQAA..QIPVNDPMLLVSAMALVTEHLGF
Rhodococcus_sp._1R11_WP_135042739.1:1-449            KLLERGLFDGLFVADVLGTYDVYGGNHDA..AIRHGA..QIPVSDPLLLVSAQAAVTEHLGF
Erwinia_amylovora_WP_004157587.1:5-459               RLLERGKFDGLFIADVLGVYDVLNGNNHA..AIRNGT..QVPVNDPLALITPMALVTEHLGF
Ralstonia_mannitolilytica_WP_102079134.1:4-451       RTLERGLFDGLFIADIVGVYDVYRGNVDV..TLQESI..QLPVNDPLLLVSAMAAVTQHLGF
Leifsonia_flava_WP_135120808.1:15-465                KLLERGTFDGIFIADVLGTYDVYAGSNEA..AIRHGA..QVPVADPLLLVSAMAAATEHLGF
Cryobacterium_roopkundense_WP_084141147.1:1-453      KLLEAGTFDGIFIADVLGTYDVYGGSNEA..AIRHGA..QVPVGDPILLVSAMASVTENLGF
Cupriavidus_pauculus_WP_101681197.1:1-455            KTLERGLFDGLFFADVVGVYDVYGTSPDA..ALRGAV..QVPVNDPTLVIPAMAAVTRHLGF
Rhizobium_sp._BK333_130_WP_133704837.1:2-454         LTAERGKLDGIFLADIVGVYDVYKGSPET..SIRTGA..QIPVNDPIIPLSAMAYVTKHLGF
Caballeronia_mineralivorans_WP_047846789.1:1-454     KILEKGFFDGIFIADVIGYYDVYKGSNYH..AIHQAA..QIPVNDPLQLAAPIAMVTEHLGI
Nocardia_altamirensis_WP_069163049.1:1-454           KLLEHGHFDGIFIADVLGTYDVYGGTDIA..ALRQGA..QIPVADPLLLVSAMAAVTEHLGF
Cupriavidus_sp._P-10_WP_116322758.1:1-452            KVLERGKFDGIFLADVTGVYDVYGSNCDA..ALRNGV..QVPNCDPFLLVPAMAAVTEHLGF
Serratia_sp._S1B_WP_116726564.1:1-452                KILERGYFDGLFIADVLGIYDVYHHSAEH..ALTGAV..QVPVNDPLQIVPAMAAVTHHLGF
Pseudomonas_putida_WP_043864435.1:1-452              RTLERGKIDGLFIADVLGAYDVYGGNADE..ALRTAA..QVPVNDPLQLVPAMAAVTEHLGF
Pseudomonas_aeruginosa_WP_023084248.1:4-453          RLLERGLFDGLFIADILGVYDVYQGGIEV..TAREAV..QLPVNDPLMLVSAMAGVTEHLGF
Pseudomonas_mucidolens_WP_084381795.1:9-455          QLLERGLFDGLFIADIVGVYDVYQNAVDV..PLKESI..QLPVNDPLLLVSAMAAATRNLGF
Pseudomonas_poae_WP_060549803.1:4-450                KTLERGLFDGLFIADIVGVYDVYQHCIDV..PLKESI..QLPVNDPLLLVSAMAAVTRNLGF
Pseudomonas_chlororaphis_WP_123573412.1:9-454        QLLERGLFDGLFIADIVGVYDVYQNSVDV..TLKESI..QLPVNDPLLLVSAMAAVTRNLGF
Janthinobacterium_lividum_140_WP_072454679.1:1-44    QLLEAGLFDAVFLADVLGVYDVYRGSLDA..ALEHGV..QVPLNDPLALVPIMAQATTHLGF
Haladaptatus_cibarius_WP_049970513.1:4-446           RLAERGKFDAVFFADVRGIYDVHGGNRDT..AIEKAI..QTPANDPELLIPAMASVTDDLGF
Arthrobacter_luteolus_WP_066298473.1:1-443           KTLERGTFDALFLADVLGIYDVYRNSSAP..ALLDAD..QVPLNDPFMQISAMAAVTRNLGF
Geobacillus_sp._ZGt-1_WP_047758532.1:1-436           KRLEAGLFDAVFFADVLGVYDTYRQSKDP..SIRDGM..QFPLNDASLIIPVMASVTTHLSF
Lysinibacillus_composti_WP_124766485.1:4-458         KLLEKWRFDGIFIADVLGTYDVYGGSRDT..AVRHGV..QSPVNDPFQIVPLMSAVTKNLGF
Microcella_putealis_WP_130486201.1:20-474            QLLERGTFDGIFIADVLGTYDVYGGTNEA..AIRHGA..QVPVNDPILLVSAMAAVTEHLGF
Pseudomonas_nitroreducens_WP_037016976.1:1-453       KTLERGRIDGLFLADVLGVYDVYHGNADA..ALSAGA..QVPANDPLQIVPAMAAATEHLGF
Acinetobacter_wuhouensis_WP_130168784.1:1-453        KILEKGLFDGIFIADVLGIYDVYHHSAEH..ALTGAV..QVPVNDPLQIVPAMAAVTKHLGF
Bradyrhizobium_sp._YR681_WP_008144663.1:1-454        RTLERGRFDGLFLADVLGVYDVYGNSPDA..ALRNAA..QTPSNEPLLLLSAMAAVTKNLGF
Schumannella_sp._10F1D-1_WP_141164655.1:10-460       KLLEKGTFDGIFIADVLGTYDVYGSSNEA..AIRNGA..QVPVNDPLLLVSAMALVTEHLGF
Pseudomonas_coronafaciens_150_WP_122355187.1:4-45    KLLERGLFDGLFIADIVGVYDVYQNSVDV..TLKEAI..QLPVNDPLLLVSAMAGVTTHLGF
Pseudomonas_syringae_WP_003430238.1:4-452            KLLERGLFDGLFIADIVGVYDVYQNSVDV..TLKEAI..QLPVNDPLLLVSAMAGVTRHLGF
unclassified_Rhodococcus_WP_094682261.1:1-449        KLLERGLFDGLFVADVLGTYDVYGGNHDA..AIRHGA..QIPVSDPLLLVSAQAAVTEHLGF
Hafnia_alvei_WP_046449330.1:6-449                    KLLERGLFDGLFIADIVGVYDVYQQGMGL..TARESI..QLPVNDPMLLVPAMASVTQHLGF
AS9A_3890_Hoyosella_subflava_WP_013808677.1:1-453    KLLERGVFDGLFIADVLGTYDVYGGTDEA..ALREGA..QIPVADPLLLVSAMAAVTQHLGF
Acinetobacter_baumannii_WP_000995600.1:1-443         QILERGFFDGIFIADVLGIYDVYHQSAEH..ALTGAV..QVPVNDPLQIVPAMAAVTKHLGF
Agrobacterium_sp._B133/95_WP_065696417.1:2-454       RTAERGKLDGIFLADIVGVYDVYKGSPET..SIRTGA..QIPVNDPIIPLSAMAYVTKHLGF
Microbacterium_sp._KROCY2_WP_024289149.1:1-453       QVLERGRFDALFLADVVGIYDIYRNSAAP..SIAGGA..QVPVNDPFMQISAMAAVTKNLGF
Variovorax_paradoxus_WP_012747040.1:6-452            RTLERGLFDGLFIADIVGVYDVYQHNVDV..TLRESI..QLPVNDPLMLVPAMAAATRHLGF
Methylobacterium_radiotolerans_WP_076730229.1:5-4    KLLERGLFDGLFIADIIGVYDVYGGGIDV..TAREAV..QLPVNDPTVMISAMAAVTEHLGF
Streptomyces_sp._BK215_WP_132915265.1:7-452          RMLERGRFDTLFFADVIGTYDVYRGGLDT..AVREAT..QFPNNDPAVLIPALAHATEHLGF
Geobacillus_thermodenitrificans_160_WP_008880416.    QRLEAGLFDAIFFADVLGVYDTYRQSKDP..SIRDGM..QFPLNDAALVIPAMASVTNHLSF
Pseudomonas_aeruginosa_WP_132556951.1:356-785        RLLERGLFDGLFIADILGVYDVYQGGIEV..TAREAV..QLPVNDPLMLVSAMAGVTEHLGF
Penicillium_solitum_OQE02104.1:14-471                QLLESAKFHGIFIADVLGGYDVYKGPRNLDPAVVSGA..QWPVNEPLAVVPAMAAATKNIGF
Penicillium_vulpinum_OQE05502.1:12-469               QLLESAKFHGIFIADVLGGYDVYKGPRNLDPAVISGA..QWPVNEPLAVVPAMAAATKNIGF
Clavibacter_michiganensis_WP_045526194.1:1-454       RLLERGTFDALFIADVVGVYDVYRHSAAP..ALEDSA..QIPVGDPVVQVSAMAHATRHLGF
Glutamicibacter_creatinolyticus_WP_054821484.1:6-    KVLEKGKFDGLFIADVLGTYDVYNGTNES..PLRTGA..QAPVNDPILLVSAMAAVTENLGF
Campylobacter_sp._P0078_WP_086237522.1:4-454         KIAEKGLFDAIFIADVLGVYDVYNSNNQA..ALRGAV..QTPVNDPIQLAAIGAAVTQNLGF
Pseudomonas_furukawaii_WP_003454569.1:1-447          RLLERGLFDGLFLADILGVYDVYQGGVEL..TLRESI..QLPVNDPLLLVSAMAGVTSHLGF
Leucobacter_triazinivorans_WP_130111548.1:1-442      RIAERGLFDSIFIADVLGTYDVFGGNDVA..AIRNGA..QVPVNDPVQLAAVMAAVTEHVGF
Aspergillus_brasiliensis_CBS_101740_OJJ68286.1:12    KLLESAKFHGIFIADVLGGYDVYKGPRNLEPAIVSGA..QWPVNEPLAVVPAMAAVTQNIGF
Aspergillus_udagawae_170_GAO89440.1:15-472           QLLESAKFHGIFIADVLGGYDVYKGPRNLAPAIASGA..QWPVNEPLAVISAMAAATRNIGF
Aspergillus_flavus_RMZ37351.1:15-472                 KLLESAKFHGIFIADVLGGYDVYKGPRNLEPAIISGA..QWPVNEPLAVVPAMAAATKNIGF
Aspergillus_flavus_RAQ54081.1:15-472                 KLLESAKFHGIFIADVLGGYDVYKGPRNLEPAIISGA..QWPVNEPLAVVPAMAAATKNIGF
Penicillium_arizonense_XP_022490779.1:12-469         QLLESAKFHGIFIADVLGGYDVYKGPRNLDPAAISGA..QWPVNEPLAVVPAMAAVTKNIGF
Salinibacterium_sp._CGMCC                            KLLERGRFDGIFIADVLGTYDVYGGSNEA..PIKHGA..QVPVADPLLVVPAMAYVTEHLGF
Aspergillus_fumigatus_OXN06785.1:14-471              QLLESAKFHGIFIADVLGGYDVYKGPRNLAPAIVSGA..QWPVNEPLAVVSAMAAATKNIGF
Aspergillus_lentulus_GAQ10210.1:7-464                QLLESAKFHGIFIADVLGGYDVYKGPRNLAPAIASGA..QWPVNEPLAVVSAMAAATKNIGF
Aspergillus_flavus_RAQ66103.1:15-472                 KLLESAKFHGIFIADVLGGYDVYKGPRNLEPAIISGA..QWPVNEPLAVVPAMAAATKNIGF
Aspergillus_fumigatus_OXN22894.1:14-471              QLLESAKFHGIFIADVLGGYDVYKGPRNLAPAIVSGA..QWPVNEPLAVVSAMAAATKNIGF
Aspergillus_nidulans                                 QLLESAKFHGIFIADVLGGYDVYKGPRNLEPAITSGA..QWPVNEPLAVVPAMAAATKNIGF
Penicillium_nalgiovense_OQE79848.1:14-471            QLLESAKFHGIFIADVLGGYDVYKGPRNLDPAVVSGA..QWPVNEPLAVVPAMAAATKNIGF
Aspergillus_wentii                                   QLLDKANFHAMFIADTLGPYDVYKGPANVVPALASGA..QFPVNDPLYLVPAMAAVTKNLVF
Gordonia_iterans_WP_105943624.1:1-453                QVLERGRFDGLFIADVLGTYDVYAGTDEA..AIRQGA..QIPVNDPLLLVSAMAHATEHLGF
Azotobacter_beijerinckii_WP_090735436.1:4-454        RLLERGLFDGLFIADIAGVYDVYRQSVDL..TLREAI..QLPVHDPLMLVSAMAGATRHLGF
Staphylococcus_sp._NAM3COL9_WP_057513692.1:4-453     QTLEKGKFDSIFIADVLGTYSVYNQSSDA..AIKHAV..QLPAHDPIPIISAMATVTKHIGF
Acidovorax_sp._56_WP_099656603.1:11-450              RTLERGLFDGLFIADIVGVYDVYQAGIDL..TLRESI..QLPVNDPVVLVSAMAAVTQHLGF
Paenibacillus_sp._BIHB4019_WP_099518466.1:1-456      RTLERGKFDFMFFADSYG.YP..KERTEL..AFREAS..GAVGNDPMLLIPALAQATEHLAF
Penicillium_antarcticum_OQD90033.1:5-465             KKLEDAKFHAIFFADVLGGYDVYRGPVSLDPTIPAGA..QFPINDPLYSVPAMAAATEHIGF
Aspergillus_oryzae_RIB40_190_XP_001823703.1:9-463    QLLDNAGFHAMFIADTLGPYDVYKGPANVVPTLASGA..QFPVNDPLYLVPAMAAVTKNLIF
Fusarium_oxysporum_f._sp._pisi                       QLLEKGKFHGIFIADVLGSYDVYKGPRNPDPAIISGA..QWPVNEPLATVPAMAAATKNIGF
Colletotrichum_fructicola_Nara_gc5_ELA35345.1:18-    QLLDDAGFHAMFIADTLGPYDVYKGPANVVPALAAGA..QFPVNDPLYLVPAMAAATKNLIF
Micrococcus_luteus_WP_041104460.1:19-468             QTLEKGLFDGLFIADVLGPYSVYGGTSEA..AIKTGA..QTPVNDPFLLVSAMAAVTEHLGF
Aspergillus_awamori_GCB21923.1:12-469                KLLESAKFHGIFIADVLGGYDVYKGPRNLEPAIISGA..QWPVNEPLAVVPAMAAATQNIGF
Aspergillus_niger_CBS                                KLLESAKFHGIFIADVLGGYDVYKGPRNLEPAIISGA..QWPVNEPLAVVPAMAAATQNIGF
Aspergillus_lacticoffeatus_CBS_101883_XP_02545389    KLLESAKFHGIFIADVLGGYDVYKGPRNLEPAIISGA..QWPVNEPLAVVPAMAAATQNIGF
Aspergillus_wentii_DTO_134E9_OJJ40237.1:14-471       KLLESAKFHGIFIADVLGGYDVYKGPRNLDPAIISGA..QWPVNEPLAVIPAMAAATQNIGF
Aspergillus_indologenus_CBS_114.80_PYI31207.1:11-    QLLEAAKFHGIFIADVLGGYDVYKGPRNLGPAIASGA..QWPVNEPLAVVPAMAAVTKSIGF
Aspergillus_fischeri_NRRL_181_XP_001264002.1:14-4    QLLESAKFHGIFIADVLGGYDVYKGPRNLAPAIASGA..QWPVNEPLAVVSAMASATKNIGF
Penicillium_coprophilum_200_OQE46388.1:11-466        QLLDKAGFHAMFLADTLGAYDVYKGPNNVVPALASGA..QFPVNDPLYLVPAMSAVTKNLIF
Cupriavidus_taiwanensis_WP_116379876.1:8-454         RTLERGLFDGLFIADIVGVYDVYRGNVDV..TLQESI..QLPVNDPLLLVPAMAAATRHLGF
Penicillium_digitatum                                KKLEDAKFHAIFFADVLGGYDVYRGPSNLSPTIPAAA..QFPINDPLYSVPAMAAATESIGF
Aspergillus_niger_GAQ46262.1:12-469                  KLLESAKFHGIFIADVLGGYDVYKGPRNLEPAIISGA..QWPVNEPLAVVPAMAAATQNIGF
Aspergillus_nomius_NRRL_13137_XP_015412385.1:15-4    KLLESAKFHGIFIADVLGGYDVYKGPKNLEPAIISGA..QWPVNEPLAVVPAMAAATKNIGF
Aspergillus_aculeatus_ATCC_16872_XP_020055010.1:1    QLLEAAKFHGIFIADVLGGYDVYKGPRNLGPAIASGA..QWPVNEPLAVVPAMAAVTKSIGF
Penicillium_brasilianum_OOQ84969.1:19-474            QLLDKANFHAMFIADTLGPYDVYKGPANVVPALASGA..QFPVNDPLYLVPAMAAVTKNLIF
Penicillium_subrubescens_OKP15100.1:19-474           QLLDKANFHAMFIADTLGPYDVYKGPANVVPALASGA..QFPVNDPLYLVPAMAAVTKNLIF
Trichoderma_arundinaceum_RFU80530.1:19-474           QLLDKADFHAMFIADTLGPYDVYKGPANVVPALASGA..QFPVNDPLYLVPAMAAVTKNLIF
Aspergillus_thermomutatus_210_XP_026614594.1:15-4    QLLESAKFHGIFIADVLGPRNLAP.......AIASGA..QWPVNEPLAVVSAMAAATRNIGF
Serratia_liquefaciens_WP_044551273.1:4-449           QLLERGLFDGLFIADILGVYDVYQQNIAL..TARESI..QLPVNDPLLLVSAMAAVTHHLGF
Micrococcus_luteus_WP_065572847.1:19-468             QTLEKGLFDGLFIADVLGPYSVYGGTSEA..AIRTGA..QTPVNDPFLLVSAMAAVTEHLGF
Halolamina_rubra_WP_049979911.1:1-449                RTAERGKFDAVFFADVRGIYDVYGGDRET..AIEKAV..QTPSNDPALVVPAMAEVTDDLGF
Acinetobacter_pittii_WP_075382847.1:7-452            KTLESGLFDGLFIADITGVYDVYQNSIDL..TLKESI..QLPSHDPSTLISAMAAVTQNLSF
Corynebacterium_variabile_WP_052302534.1:2-464       PLLETGYFDGLFLADVLGLYDLYGASPDA..ALRAAA..QVPVNDPLPLVSAMAAVTDNLGF
Aspergillus_luchuensis_GAT21254.1:12-471             KLLESAKFHGIFIADVLGGYDVYKGPRNLEPAIVSGA..QWPVNEPLAVVPAMAAATQNIGF
```

```
Aspergillus_luchuensis                                  KLLESAKFHGIFIADVLGGYDVYKGPRNLEPAIVSGA..QWPVNEPLAVVPAMAAATQNIGF
Aspergillus_bombycis_XP_022386026.1:15-472              KLLESAKFHGIFIADVLGGYDVYKGPRNLEPAIISGA..QWPVNEPLAVVPAMAAATQNIGF
Agrobacterium_vitis_WP_070163524.1:1-454                RTLERGIFDGIFIADVIGYYDVYKGSNFH..AIRQAA..QIPVNDPLQLAAPIALATEHLGI
Pseudomonas_psychrotolerans_220_WP_074584491.1:5-       QLLERGLFDGLFIADILGVYDVYGGNVDL..TLRESI..QLPVNDPFLLVSAMAASTRHLGF
Micrococcus_yunnanensis_WP_135040276.1:19-468           QTLEKGLFDGLFIADVLGPYSVYGGTSEA..AIKTGA..QTPVNDPFLLVSAMAAVTEHLGF
Acinetobacter_calcoaceticus_WP_004642549.1:7-452        KTLESGLFDGLFIADITGVYDVYQNGIDL..TLKESI..QLPSHDPSTLISAMAAVTQNLSF
Penicillium_brasilianum_CEO59775.1:15-472               QLLESAKFHGIFIADVLGGYDVYKGPRNLDPAIVSGA..QWPVNEPLAVVPAMAAATKNIGF
Aspergillus_cristatus_ODM22551.1:12-469                 KLLESAKFHGIFIADVLGGYDVYKGPRNLEPAIVSGA..QWPVNEPLAVVPAMAAATQNIGF
Pseudomonas_taiwanensis_WP_027907363.1:1-446            RLLERGLFDALFIADVLGIYDVYQGGPQA..ALRGGV..QVPVNDPLLLVPAMAGVTEHLGF
Acidovorax_avenae                                       RELERGLFDGLFLADIVGVYDTYGQSADV..TLRESV..QLPVNDPVLVVPAMAAATRHLSY
Staphylococcus_pseudintermedius_WP_101431388.1:4-       QTLERGLFDGIFIADVLGTYSVYNQSHDA..AVKHAV..QLPAHDPIPLVSAIAAVTEHIGI
Rhodococcus_triatomae_WP_081607437.1:2-451              RALERGMFDSLFLADFPGINDVYQGSNEA..SLREAI..AVPINDPSMAVAAMASVTRHLGF
Alcaligenes_sp._RS4_230_WP_128393375.1:4-450            RVLERGFFDGLFIADIIGYYDVFEGNVDL..TLKEAI..QLPVNDPWTLVSAMAAVTEHLGF
Gibbsiella_quercinecans_WP_121552947.1:7-454            RLLERGLFDGLFIADILGVYDVYQNGIDL..TAREAI..QLPVNDPLLLVSAMASVTQHLGF
Acinetobacter_ursingii_WP_044435211.1:5-452             KTLEAGLFDGLFIADITGVYDVYQNNIDL..TLRESI..QLPSHDPSTLVSAMASVTQHLGF
Erwinia_typographi_WP_034887712.1:4-447                 QLLERGLFDGIFIADILGVYDVYQQGINL..TASESI..QLPVNDPLMLVSAMAGVTQHLGF
Trichoderma_harzianum_KKP01913.1:14-470                 QLLESAHFHGIFIADVLGPYDVYKGPRNPDPAIISGA..QLPVNEPLVLVPAMAAATKNIGF
Fusarium_graminearum_235_CEF79766.1:12-466              KLLEEAKFHGIFIADVLGGYDVYKKSLDP..AIISGA..QWPVTEPLSVIPAMAAVTKNISF
Gordonia_alkanivorans_WP_006357654.1:10-461             RLLETGGFDGLFIADVLGTYDVLSGTDEA..AIRQGA..QIPVNDPLLLVSAMAHATKNLGF
Acinetobacter_radioresistens_WP_111281495.1:5-455       KTLEQGLFDGLFLADITGVYDVYQNGIEL..TLKESI..QLPSHDPSTLVSAMAAVTQNLGF
Mesorhizobium_plurifarium_WP_073985566.1:5-458          KTLEEGLFDGLFVADVLGVLDTHGGSIAG..TLKQGV..QTPSTDPLLAVSAMAAATRHLGF
Oceanobacillus_halophilus_WP_121203049.1:1-454          KVLERGMFDAVFIADVLGTYSVYGGNHES..AVRHAV..QLPAHDPLLPISAMAAVTEHIGF
Acinetobacter_tandoii_240_WP_100241535.1:7-457          KTLEQGLFDGLFIADITGVYDVYQNGIDL..TLKESI..QLPSHDPSTLVSAMAAVTQHLGF
Burkholderia_plantarii_WP_042624083.1:1-457             RTLEAGLFDGLFLGDVIGVYDVFGNAPDA..ALRTAA..QVPMNDPAVLIPLMAHATRHLGF
Alcaligenes_faecalis_WP_094195208.1:6-457               KTLEKGLFDGIFLADVVGYYDVYQQGVDL..TLREGI..QLPVNNPWLLVSAMAAVTQHIGF
Bradyrhizobium_arachidis_WP_092214516.1:1-453           KILEAGKFDSIFIADGIGIHDVYAGTADA..ALNTGA..QIPKLDPMLLVSAMAHVTEHLGL
Cupriavidus_plantarum_WP_109583068.1:1-458              KLLERGRFDSLFLGDITGIYDVAGGSAEM..ALRTAA..QVPMNDPAPLIPLMAHATEHLGF
Variovorax_sp._770b2_245_WP_093443018.1:5-461           RTLERGLFDGLFLADVSGVYDVWGGSPDA..ALRHAI..QVPINDPAVLVSAMASVTRHLGF
RZJ22128.1:4-447                                        KTAEKGLFDAYFLADGLSVR..WGSAIEG..ELGLGD..KGVGFEPVTLFAALSAVTKNLGF
LadB_Geobacillus_thermoleovorans_BAM76371.1             KAAEAHGFSTLLLPTGAGCL........................DSLAVAAYLAARTTKLHL
LadAbeta_Geobacillus_thermoleovorans_BAM76372.1         EIAEQGKLDMLFVSDGLAIE...........PLSHPA..EIVRLEPFTLLAALAAVTNRIGL
Af1_AFLA_024140_XP_002373925.1                          KLLESAKFHGIFIADVLGGYDVYKGPRNLEPAIISGA..QWPVNEPLAVVPAMAAATKNIGF
Af3_AFLA_58870_XP_002378631.1                           QKLEAAKFHAIFFADVLGGYDVYKGPANLDPTIPAGA..QFPINDPLYSVPAMAAATESIGF
Af4_AFLA_049780_XP_002383105.1                          KLLERGGINALFLADTYGGYDTYEGSLDE..CIRRAA..QWPVTDPTIPISAMAAVTKNLAF
Af5_AFLA_126060_XP_002381799.1                          KVLEKGKFHGLFLADHLGIYDVYKGPGNKEPALLSGA..QFPIGDPFLLISAMASVTTSLSF
Af2_QMW32876.1:9-463                                    QLLDNAGFHAMFIADTLGPYDVYKGPANVVPTLASGA..QFPVNDPLYLVPAMAAVTKNLIF
Yarrowia_lipolytica_CLIB122_YALI0C07546p_XP_50156       KTLEKGKFHAIFIADHLGFREVYGDGDPI..GLEKKI..QFPCHDPLFLVPIMASVTDHLNF
Rhizobium_sp._CF122_WP007794079.1                       QLAEAAKFDLIFMADGVG...TRGTDIEALSRTAVRYVAQF...EPITLLSALAAVTKNIGF
Acinetobacter_sp._NIPH_2100_LLM                         QLAEKGLFDAYFLADGLAVN..FGQAEKT....GYSD..KVAGFEPVTLFAALSTVTKHIGF
Nostoc_sp._PCC_7107_WP_015115963.1                      QIAERGLFDAVFLADSPG...VWGGSPET...QKRNG..KLVHFEPVTLFSALSAVTKNIGF
Aspergillus_flavus_NRRL3357_uncharacterized             KKLEDAKFDAFFMADHLAVLNMPVE......ALKRSH..TVTSFEPFTLLSALSVVTEKIGL
Paenibacillus_curdlanolyticus_WP006037998.1             LTAERGKFDMVFLADGYAVHERYPEALEQ..TVNVRP.......EPFTLLSALGAATTNIGL
LadAbeta_Geobacillus_thermoleovorans_FMN-dependen       EIAEQGKLDMLFVSDGLAIE...........PLSHPA..EIVRLEPFTLLAALAAVTNRIGL
SsuD_1M41                                               QAADRLGYTGVLIPT........................GRSCEDAWLVAASMIPVTQRLKF
Paenibacillus_curdlanolyticus_WP006037998.1             LTAERGKFDMVFLADGYAVHERYPEALEQ..TVNVRP.......EPFTLLSALGAATTNIGL
Bacillus                                                QAADRLGFEGVLIPT........................GKSCEDPWLLAASLIPETERLKF
Pseudomonas                                             ............AADSLGYHGV.........LIPTGR....SCEDSWVVASALAPLTERLRF
LadB_Geobacillus_thermoleovorans_FMN-dependent          ............................................DSLAVAAYLAARTTKLHL
Pseudomonas_aeruginosa_PAO1_NP_251290.1                 QAHEDAGFDRVLV....GYFS..........NAAEGA..........VVSSFVAAATRRLGV
Alkanesulfonate                                         ............................................DPFTVGSAIASVTKNLKI
Alkanesulfonate                                         RNLDDYAFNYTLVPY........................DSSYFDPWTIGATIASKTKNLKI
Aspergillus_niger_ATCC13496_RDH19503.1                  RSLDDYGFNYTLVPY........................DSSYFDPFTIGATIASVTKNLKI
Penicillium_digitatum_Pd1_XP014534980.1                 RNLDDYGYNYTLVPYGSALY........................DPFTIGATILAVTKNVKV
Acinetobacter_rudis_WP_016657872.1                      VSLQYLRQIAQA.ADYLGYY...........GVLIPT..GRSCEDSWVVASALAPLTEKLRF
```

```
                                                                   110        120        130        140        150
LADA_Geobacillus_thermodenitrificans_WP_011888513          AVTFSTTYE.HPYGHARRMSTLDHLTKGRIAWNVVTSHLPSADKNF...GIKKILEHDERYD
Geobacillus_sp._LEMMY01_WP_079935807.1:1-440               AVTFSTTYE.HPYGHARRMSTLDHLTKGRIAWNVVTSHLPSADKNF...GIKKILEHDERYD
Geobacillus_stearothermophilus_ABV66256.1:1-440            AVTFSTTYE.HPYGHARRMSTLDHLTKGRIAWNVVTSHLPSAHKNF...GIKKILEHDERYD
Geobacillus_sp._B4113_201601_WP_061912600.1:1-440          AVTFSTTYE.HPYGHARRMSTLDHLTKGRIAWNVVTSHLPSADKNF...GIKKILEHDERYD
Geobacillus_sp._B4113_201601_5WP_061912600.1:1-44          AVTFSTTYE.HPYGHARRMSTLDHLTKGRIAWNVVTSHLPSADKNF...GIKKILEHDERYD
Bacillus_thuringiensis_6WP_029441578.1:1-435               AVTFSTSYE.HPYGYARRMSTLDHLTKGRMAWNIVTSHLSSAEKNF...GIETKLNHDEKYD
Lysinibacillus_sphaericus_WP_103976887.1:2-422             AVTFSTSYE.PPYSFARRMSTLDHLTEGRMAWNIVTSHLDSADKNF...EIKHLLTHDEKYD
Lysinibacillus_sphaericus_WP_069508309.1:2-422             AVTFSTSYE.PPYSFARRMSTLDHLTEGRMAWNIVTSHLDSADKNF...EIKHLLTHDEKYD
Rummeliibacillus_sp._SL167_10_WP_146551437.1:2-42          AVTFSTSYE.HPYGFARRMSTLDHLTNGRIAWNIVTSHLESADKNF...EVKQYLDHDEKYD
Paenibacillus_sp._DXL2_WP_110843609.1:1-459                AVTFSTTYE.HPFAHARRMSTLDHLTKGRIAWNVVTSYLPSAARNF...GLDRMVRHDERYE
Paenibacillus_naphthalenovorans_WP_074730028.1:1-          AVTSSTTYE.HPFGHARRMSTLDHLTKGRIAWNVVTSYLPSAARNF...GLEHMVRHDERYE
Cohnella_sp._HS21_WP_130606586.1:1-453                     AVTIPTTYE.PPYAHARRMSTLDHLTNGRVGWNIVTSFLPSAALNF...GLDRMVTHDERYE
Streptosporangium_subroseum_15_WP_089205784.1:1-4          AVTFSTTYE.PPFGHARRMSTLDHLTKGRVAWNVVTSYLRSAARNF...GLDDEIEHDLRYE
Arthrobacter_sp._Hz2_WP_120147654.1:1-455                  AATFSTTYE.PPFAFARRASTLDHLTKGRFGWNIVTSYLPNAARNF...GLPDEVEHDQRYA
Nonomuraea_solani_WP_103963668.1:1-453                     AVTFSTTYE.PPFAHARRMSTLDHLTKGRVAWNVVTSYLRSAARNF...GLDDEIEHDRRYE
Arthrobacter_ruber_WP_105030338.1:1-455                    AATFSTTYE.PPFAFARRASTLDHLTKGRFGWNIVTSYLPNAARNF...GLADEVEHDQRYA
Bacillus_aciditolerans_20_WP_121446947.1:1-456             AVTVSTTYE.HPFGHARRFSTLDHLTKGRIAWNIVTSYLPNAARNF...GLPEMLKHDARYD
WP_137163763.1:1-458                                       GVTFSLTYE.HPYPFARRMSTLDHLSNGRVGWNIVTGYLDSAARNL...GLERQLGHDQRYD
Aeribacillus_pallidus_WP_130157503.1:4-457                 GITCSTTFE.HPYTFARRMSTLDHLTKGRVGWNIVTSYLESGTKNI...DIGDRFLHSERYN
Bacillus_megaterium_24WP_098602848.1:4-460                 GITCSTTFE.HPYTFARRMTTLDHLTKGRIAWNIVTSYLESGTKNI...EIGNKFQHDERYN
Streptomyces_sp._150FB_WP_078877664.1:5-455                AATFSTTYE.PPFAFARRMSTLDHLTKGRVAWNVVTSYLPNAARNF...GLSDEVDHDHRYE
Arthrobacter_sp._DWC3_WP_104051071.1:1-454                 AATFSTTYE.PPFAFARRASTLDHLTKGRFGWNIVTSYLPNAARNF...GLADEVEHDQRYA
Cohnella_thermotolerans_WP_027093898.1:1-456               ALTVSTTYE.HPYAHARRMSTLDHLTKGRLAWNVVTSYLPSAARNF...GLEEMIKHDDRYE
Paenibacillus_bovis_28WP_144921429.1:1-452                 GVTASVTHE.HPYTFARRMSTLDHLTKGRVGWNIVTSYLKSAAVNM...GLEDQINHDERYD
Bacillus_aciditolerans_WP_121447760.1:1-452                GVTASVTHE.HPYTFARRMSTLDHITKGRVGWNIVTSYLKSAAVNM...GLEDQIKHDERYD
Leifsonia_sp._ALI-44-B_30_WP_077053645.1:1-455             AATFSTTYE.PPFAFARRASTLDHLTKGRFGWNIVTSYLPNAARNF...GHDDEVAHGDRYA
Parageobacillus_thermoglucosidasius_WP_064552269.          ALTVSTTYE.HPFSTARRFSTLDHLTKGRIAWNVVTSYLPNAARNF...GLQEMIKHDQRYE
Geobacillus_sp._Y4.1MC1_WP_013399886.1:1-459               ALTVSTTYE.HPFNIARRFSTLDHLTKGRIAWNIVTSYLPNAARNF...GLPEMIKHDQRYD
Bacillus_methanolicus_35WP_004434428.1:1-462               AFTVSTTYE.PPFAHARRFSTLDHLTQGRIAWNVVTSYLPNAARNF...GLPEMIKHDRRYD
Terribacillus_halophilus_WP_077309754.1:1-448              GVTASVTYE.HPYSFARRVSTLDHLTNGRVGWNIVTSYLESAARNL...GLDTQIKHDERYN
Paraburkholderia_kururiensis_WP_017777532.1:1-453          GVTCSLSYE.HPYPFARRMSTLDHLTNGRVGWNIVTSYLDSAARNV...GLPSQANHDERYA
Gracilibacillus_massiliensis_WP_058308419.1:1-456          AVTVSTTYE.APFGNARRFSTLDHLTKGRVAWNVVTSYLPNAARNF...GLEDMIKHDERYD
Streptomyces_yanglinensis_WP_103888551.1:5-452             AATFSTTYE.PPFAFARRMSTLDHLTRGRVAWNVVTSYLPNAARNF...GLEDEVDHDHRYE
Trinickia_sp._7GSK02_40_WP_136896361.1:1-452               GVTCSLSYE.HPYPFARRMSTLDHLTNGRVGWNIVTSYLDSAARNV...GLPAQANHDERYA
Cryobacterium_sp._NEAU-85_WP_123045138.1:1-448             AVTFTTYE.PPFGNARRFSTLDHLTNGRVAWNVVTGYLPDAARNY...GLSAQVKHDDRYD
Alicyclobacillus_acidoterrestris_WP_021298468.1:4          GVTASVSYE.HPYTFARRMSTLDHLTKGRVGWNIVTSYLDSAARNI...GLHEQVSHDERYD
Oceanobacillus_senegalensis_WP_085994229.1:1-452           GVTASTSYE.HPYSFARRMSTLDHLTKGRVGWNVVTSFLNSAAINL...GLDKEVSHDKRYE
Bacillus_sinesaloumensis_WP_077620983.1:1-449              AITVSTTYE.APFGNARRFSTLDHLTKGRIAWNIVTSYLPNAARNF...GLQDMIKHDERYD
Halopenitus_persicus_WP_021074147.1:6-457                  AATHSVTYT.KPYMTAKRLSTLDHLTDGRIAWNIVTSYLEDAAVNL...GLEGRIEHDERYD
Microbacteriaceae_bacterium                                AVTFTTTYE.PPFPNARRFSTLDHLTRGRVAWNVVTGYLPDAAKNF...GLSEQVRHDDRYD
Lysinibacillus_sinduriensis_WP_036201100.1:1-458           IVTVSTSYE.HPFSNARRFSTLDHFTKGRVGWNIVTSYLPNAAQNY...GQKEMIKHDQRYE
Bacillus_endophyticus_WP_113749053.1:5-457                 GITCSTTFE.HPYTFARRMTTLDHLTNGRIAWNIVTSYLESGMKNI...DAGHKIQHEERYN
Pseudomonas_monteilii_WP_119371117.1:1-455                 GLTASLTFE.HPYPFARRLSTLDHLTKGRIGWNIVTSYLDSGARNL...GQKALSDHDARYD
Bacillus_endophyticus_50_WP_061801645.1:5-457              GITCSTTFE.HPYTFARRMTTLDHLTNGRIAWNIVTSYLESGMKNI...DTGHKIQHEERYN
Planococcus_maitriensis_WP_112233285.1:1-457               GVTASVSHE.HPYTFARRMSTLDHLTKGRIGWNVVTSYLKSAAVNI...GLDDQMSHDERYE
Oceanobacillus_profundus_WP_118889880.1:1-447              GITASVTHE.HPYTFARKISTLDHLTNGRIGWNIVTSYLKSAAVNM...GLEDQINHDERYN
Burkholderia_pseudomallei_WP_141404579.1:1-452             GITCSLSYE.HPYPFARRMSTLDHLTGGRVGWNVVTSYLDSAARNL...GLPRQANHDERYA
Terribacillus_saccharophilus_WP_095261886.1:1-455          AVTVSTTYE.PPFGNARRFSTLDHLTKGRVAWNVVTSYLPNAARNF...GLTDMIKHDERYE
Sediminibacillus_albus_WP_093212831.1:4-453                GVTASVSHE.HPYTFARRMSTLDHLTKGRVGWNIVTSYLKSAAVNM...GLEDQVGHDERYE
Sciscionella_marina_WP_020496929.1:1-448                   AVTFTTTYE.PPFGNARRFSTLDHLTRGRIAWNVVTGYLPDAARNY...GLGAQVGHDDRYE
Mycetocola_saprophilus_WP_043321267.1:1-452                AVTFATTYE.SPFANARRLSTLDHLTKGRLAWNVVTGYLPDAAKNF...GLSEQVTHDVRYE
Lysinibacillus_macroides_WP_053993569.1:4-460              GITCSTTFE.HPYPFARRISTLDHLTKGRVAWNIVTSYLESGTKNI...DIGDRFQHQERYH
Pseudomonas_putida_WP_086978777.1:1-458                    GLTASLTFE.HPYPFARRLSTLDHLTKGRIGWNIVTSYLDSGARNL...GQKALSDHDARYD
Sediminibacillus_halophilus_60_WP_074598704.1:4-4          GVTASVSHE.HPYTFARRMSTLDHLTKGRVGWNIVTSYLKSAAVNM...GLEDQVSHDERYD
Rhizobium_oryzae_WP_085420425.1:1-450                      GITANLTYE.PPFLFARRMSTLDHLTNGRIGWNIVTGYLDSAAKAI...GLDTQAAHDDRYD
Pseudomonas_extremorientalis_WP_071491956.1:1-455          GLTASLSFE.HPYPFARRLSTLDHLTKGRIGWNIVTSYLESGAKNL...GQKAQTEHDARYD
Pseudomonas_fluorescens_WP_016977452.1:1-454               GLTASLSFE.HPYPFARRLSTLDHLTKGRIGWNIVTSYLESGAKNL...GQKAQTEHDARYD
Neorhizobium_sp._T25_27_WP_105419890.1:1-450               GVTANLTHE.PPFVFARRMSTLDHLTGGRIGWNIVTGYLDSAAKAI...GLDGMAAHDDRYD
Pseudomonas_extremaustralis_WP_078833627.1:1-457           GLTASLSFE.HPYPFARRLSTLDHLTKGRIGWNIVTSYLESGAKNL...GQKAQTEHDARYD
Fictibacillus_aquaticus_WP_094253544.1:1-455               AITVSTTYE.QPFGNARRFSTLDHLTNGRIAWNVVTSYLPNAARNF...GLKDMVKHDKRYE
Pseudomonas_extremaustralis_KAA8559760.1:1-456             GLTASLSFE.HPYPFARRLSTLDHLTKGRIGWNIVTSYLESGAKNL...GQKAQTEHDARYD
Mycobacterium_sp._ACS1612_WP_067812332.1:1-454             GITAATSFE.HPYSFARRMSTLDHLTKGRVGWNIVTGYLESAARNH...GLDTITPHADRYD
Bacillus_megaterium_WP_116075167.1:1-455                   GVTVSTTYE.KPYAFARKFSTLDHLTNGRIAWNIVTSYLESGSINL...GLEEHFTHETRYD
Pseudomonas_frederiksbergensis_70_WP_123409596.1:          GLTASLSFE.HPYPFARRLSTLDHLTKGRAGWNIVTSYLESGAKNL...GQKNQTEHDARYD
Pseudomonas_citronellolis_SFC61163.1:1-452                 GATFSTTYE.PPFAFARRMSTLDHLTRGRVGWNIVTSYLPNAARNF...GLDDEVPHDRRYE
Herbaspirillum_chlorophenolicum_WP_050468599.1:4-          GITANLTYE.APFLFARRMSTLDHLTKGRVGWNIVTGYLDSAARAM...GLTEQLEHDQRYD
Arthrobacter_crystallopoietes_WP_074700854.1:1-45          AVTFSTTYE.PPFGNARRLSTLDHLTKGRVAWNVVTGYLPDAARNF...GLPAHIKHDDRYD
Terribacillus_goriensis_WP_038558686.1:1-438               AVTVSTSYE.QPFHNARKFSTLDHLTNGRVAWNVVTSYLPNAAENF...GLEKMVKHDERYK
Bacillus_megaterium_WP_025749753.1:1-455                   GVTVSTTYE.KPYAFARKFSTLDHLTNGRIAWNIVTSYLESGSINL...GLEEHFTHETRYD
Neorhizobium_sp._T7_12_WP_105403044.1:1-451                GVTANLTHE.PPFVFARRMSTLDHLTGGRIGWNIVTGYLDSAAKAI...GLDGMAAHDDRYD
Rummeliibacillus_sp._SL167_WP_146553619.1:4-459            GLTASTTFE.HPYTFARRISTLDHLTKGRIAWNIVTSYLESGTKNI...EIGDKFEHTKRYD
Burkholderia_sp._PAMC                                      GITVNLTYE.APYLLARRFSTLDHLSDGRIGWNIVTGYLDSAARAM...GLTGQIAHDERYD
Pseudomonas_koreensis_WP_064585463.1:4-449                 GLTANLTYE.PPYLFARRMSTLDHLSRGRVGWNIVTGYLDSAAKAM...GLSEQVEHDRRYD
Pseudomonas_fluorescens_80_WP_064116961.1:6-451            GLTANLTYE.PPYLFARRMSTLDHLSRGRVGWNIVTGYLDSAAKAM...GLSEQVEHDRRYD
Pseudomonas_rhodesiae_WP_040265276.1:4-450                 GLTANLTYE.PPYLFARRMSTLDHLSEGRVGWNIVTGYLDSAAKAM...GLTEQVEHDRRYD
Collimonas_sp._OK412_WP_092393355.1:4-454                  GVTVNLTYE.APYLFARRMSTLDHLTRGRIGWNIVTGYLDSAARAM...GLSEQPEHDARYE
Pseudomonas_marginalis_WP_058415544.1:1-456                GLTASLSFE.HPYPFARRLSTLDHLTKGRVGWNIVTSYLESGAKNL...GQKAQTEHDARYD
Pseudomonas_antarctica_WP_064452947.1:1-458                GLTASLSFE.HPYPFARRLSTLDHLTKGRVGWNIVTSYLESGAKNL...GQKAQTEHDARYD
Pseudomonas_protegens_WP_041115159.1:9-454                 GLTANLTYE.APYLFARRLSTLDHLSRGRVGWNIVTGYLDSAAQAM...GLKEQIEHDRRYD
Pseudomonas_synxantha_WP_057024776.1:4-449                 GLTANLTYE.PPYLFARRMSTLDHLSRGRVGWNIVTGYLDSAAKAM...GLNEQVEHDRRYD
Rhizobium_leguminosarum_WP_128409501.1:2-451               GVTVNTTYE.PPFLLARRMSTLDHLTKGRIGWNIVTGYLDSAARSM...GFDKLPEHDARYD
Pseudomonas_chlororaphis_WP_038359779.1:6-451              GLTANLTYE.PPYLFARRMSTLDHLSRGRVGWNIVTGYLDSAAKAM...GLSEQVEHDRRYD
Pseudomonas_syringae_90_WP_024642725.1:1-456               GVTASLSFE.HPYPFARRLSTLDHLTKGRAGWNIVTSYLESGAKNL...GQKTLTEHDARYD
Collimonas_fungivorans_WP_061540754.1:4-453                GVTVNLTYE.APYLFARRMSTLDHLTRGRIGWNIVTGYLDSAARAM...GLSQQPEHDARYE
Rhizobium_leguminosarum_WP_116409027.1:2-451               GVTVNTTYE.PPFLLARRMSTLDHLTKGRIGWNIVTGYLDSAARSM...GFDRLPEHDARYD
Microvirga_ossetica_WP_099511173.1:1-454                   GVTVNTTYE.QPYLHARRMSTLDHLTNGRIGWNIVTGYLDSAARGM...GLQEQLDHDHRYD
Pseudomonas_caspiana_WP_087268376.1:4-451                  GLTANLTYE.APYLFARRMSTLDHLSRGRVGWNIVTGYLDSAARAM...GLTEQVEHDRRYD
Pseudomonas_lundensis_WP_048375063.1:4-451                 GLTANLTYE.APYLFARRMSTLDHLSRGRVGWNIVTGYLDSAAKAM...GLKAQVEHDRRYD
Caballeronia_mineralivorans_WP_047894899.1:4-456           GVTVNLTYE.APYLLARRFSTLDHLTEGRIGWNIVTGYLDSAARAM...GLTEQIAHDERYD
Pseudomonas_paralactis_WP_057702387.1:4-450                GLTANLTYE.PPYLFARRMSTLDHLSRGRVGWNIVTGYLDSAAKAM...GLTEQVEHDRRYD
Pseudomonas_taetrolens_WP_048381143.1:4-453                GLTANLTYE.PPYLFARRMSTLDHLTRGRVGWNIVTGYLDSAAKAM...GLTEQVEHDRRYD
Pseudomonas_psychrophila_WP_123750761.1:4-450              GLTANLTYE.PPYLFARRMSTLDHLTRGRVGWNIVTGYLDSAAKAM...GLTEQVEHDRRYD
Collimonas_arenae_100_WP_061532973.1:4-454                 GVTVNLTYE.APYLFARRMSTLDHLTHGRIGWNIVTGYLDSAARAM...GLREQPEHDQRYE
Pseudomonas_viridiflava_WP_122210720.1:4-451               GLTANLTYD.APYLFARRMSTLDHLSRGRVGWNIVTSYLPSAARAM...GLSEQVEHDRRYD
Kocuria_varians_WP_068470402.1:1-454                       GVTSAVTYE.QPYTLARKFATLDHLTRGRVGFNVVTSYLPSAAENQ...GLPRQIEHDERYE
Pseudomonas_wadenswilerensis_WP_115084816.1:4-455          GLTANLTYE.PPYLFARRLSTLDHLSRGRVGWNIVTGYLDSAAKAM...GLDQQPEHDRRYD
Pseudomonas_syringae_WP_011266344.1:4-452                  GLTANLTYD.APYLFARRMSTLDHLSRGRVGWNIVTGYLDSAARAM...GLSEQNEHDRRYD
Pseudomonas_sp._286_105WP_122415793.1:4-453                GLTANLTYD.APYLFARRMSTLDHLSRGRVGWNIVTGYLDSAARAM...GLSEQVEHDRRYD
Geobacillus_vulcani_WP_031406843.1:1-461                   VLTASTTYE.HPFHIARRFSTLDHLTNGRIAWNIVTSYLPNAAKNF...GLERMIAHDQRYE
Geobacillus_icigianus_WP_033018365.1:1-461                 VLTASTTYE.HPFHIARRFSTLDHLTNGRIAWNIVTSYLPNAAKNF...GLERMIAHDQRYE
Pseudomonas_floridensis_WP_083182568.1:4-453               GLTANLTYD.APYLFARRMSTLDHLSRGRVGWNIVTGYLDSAARAM...GLREQVEHDRRYD
Pseudomonas_asturiensis_WP_073167523.1:4-451               GLTANLTYD.APYLFARRMSTLDHLSRGRVGWNIVTGYLDSAARAM...GLSEQVEHDRRYD
Geobacillus_vulcani_110_WP_031406843.1:1-461               VLTASTTYE.HPFHIARRFSTLDHLTNGRIAWNIVTSYLPNAAKNF...GLERMIAHDQRYE
```

```
Pseudomonas_syringae_WP_024682626.1:4-452                GLTANLTYD.APYLFARRMSTLDHLSRGRVGWNIVTGYLDSAARAM...GLSEQNEHDRRYD
LadAalpha_Geobacillus_thermoleovorans_BAM76377.1         VLTASTTYE.HPFHIARRFSTLDHLTNGRIAWNIVTSYLPNAAKNF...GLERMITHDQRYE
Tsukamurella_pseudospumae_WP_068746786.1:1-457           GITAGTGFE.HPFPFARRLTTLDHLTNGRIGWNVVTGYLPSAAKNM...GQLDQLQHDERYD
Kocuria_sp._Marseille-P3598_WP_085529073.1:5-456         GITSAVTYE.QPYTLARKYATLDHLTKGRVGFNVVTSYLPSAAENQ...GLPRQIEHDTRYE
Microvirga_aerophila_WP_114188789.1:1-456                GVTANLTYE.TPYLFARRMSTLDHLTRGRVGWNIVTGYLDSAARAM...GFKGQREHDDRYD
Burkholderia_ubonensis_WP_071760483.1:5-455              GVTANLTYE.PPYLFARRMSTLDHLTKGRVGWNIVTGYLDSAARGM...GLAQQIGHDDRYE
Achromobacter_marplatensis_WP_006226823.1:4-450          GVTVNLTYE.APYLLARRFSTLDHLTNGRVGWNIVTGYLESAARAM...GLSEQIAHDERYD
Acinetobacter_baumannii_WP_070165746.1:1-443             GVTTSISFE.HPYPFARRISTLDHLTQGRVGWNIVTSYLESGSKNL...GLKTQVNHDNRYD
Labedella_phragmitis_WP_128496094.1:1-453                GITAGTAYE.HPYPFARRMSTLDHLTKGRVGWNVVTGYLPSAARNM...GHEDQLQHDDRYD
Nocardia_cyriacigeorgica_120_WP_130918184.1:1-453        GITTGTGFE.HPYPFARRLSTLDHLTNGRLGWNVVTGYLPSAARNF...GADDQLDHDERYN
Nocardia_cyriacigeorgica_WP_130918184.1:1-453            GITTGTGFE.HPYPFARRLSTLDHLTNGRLGWNVVTGYLPSAARNF...GADDQLDHDERYN
Mycobacterium_sp._1164985.4_WP_067298521.1:1-453         GITTGTGFE.HPYPFARRMSTLDHLTKGRIGWNVVTGYLPAAARNM...GQTDQPAHDARYD
Rhodococcus_sp._1R11_WP_135042739.1:1-449                GITAGTGFE.HPYPFARRLSTLDHLTNGRIGWNVVTGYLPSAAQNM...GHDDQLDHDDRYD
Erwinia_amylovora_WP_004157587.1:5-459                   GLTASLSFE.HPYPFARRISTLDHLTKGRIGWNIVTSYLESGARNI...GQQAQTDHDARYD
Ralstonia_mannitolilytica_WP_102079134.1:4-451           GVTVNLTYE.PPYLLARRFSTLDHLTRGRIGWNIVTGYLDSAARAM...GLDKQFAHDERYD
Leifsonia_flava_WP_135120808.1:15-465                    GVTAGTAYE.HPYPFARRMSTLDHLTNGRVGWNVVTGYLPSAARNM...GHDDQLEHDDRYD
Cryobacterium_roopkundense_WP_084141147.1:1-453          GITAGTSYE.HPYPFARRMSTLDHLTNGRVGWNVVTGYLPSAARNM...GHDDQLEHDDRYD
Cupriavidus_pauculus_WP_101681197.1:1-455                GVTANLTYE.QPFLFARRMSTLDHLTGGRIGWNIVTGYLDSAARAI...GIDGQVAHDDRYD
Rhizobium_sp._BK333_130_WP_133704837.1:2-454             GVTVNTTYE.PPFLFARRMSTLDHLTKGRIGWNIVTGYLDSAARSM...GMERQPDHDARYD
Caballeronia_mineralivorans_WP_047846789.1:1-454         GITASTTFE.HPYTFARRLSTADHHTKGRVGWNIVTSYLESGAKNI...SEGGLRKHANRYE
Nocardia_altamirensis_WP_069163049.1:1-454               GITTGTGFE.HPFPFARRLSTLDHLTNGRIGWNVVTGYLPSAARNF...GAADQLDHDERYN
Cupriavidus_sp._P-10_WP_116322758.1:1-452                GVTGALSYE.PPYTFARKMTTLDHLTNGRVGWNIVTGYLNSAAKAS...GVTKQTDHDTRYD
Serratia_sp._S1B_WP_116726564.1:1-452                    GVTTSISFE.HPYPFARRLSTLDHLTKGRVGWNIVTSYLESGSKNL...GLKTQENHDDRYD
Pseudomonas_putida_WP_043864435.1:1-452                  GVTASVSFE.HPYPFARRMSTLDHLTRGRAGWNIVTSYLSSGARNL...GQREQLSHDQRYA
Pseudomonas_aeruginosa_WP_023084248.1:4-453              GVTANLTYE.APYPFARRLSTLDHLSDGRIGWNIVTGYLESAAKAM...GQSAQIEHDRRYD
Pseudomonas_mucidolens_WP_084381795.1:9-455              GLTANLSYE.PPYLFARRMSTLDHLSRGRVGWNIVTGYLDSAAKAM...GLREQVEHDRRYD
Pseudomonas_poae_WP_060549803.1:4-450                    GLTANLTYE.PPYLFARRMSTLDHLSRGRVGWNIVTGYLDSAAKAM...GLTEQVEHDRRYD
Pseudomonas_chlororaphis_WP_123573412.1:9-454            GLTANLTYE.PPYLFARRMSTLDHLSRGRVGWNIVTGYLDSAAKAM...GLSEQTEHDRRYD
Janthinobacterium_lividum_140_WP_072454679.1:1-44        GVTCALTYE.HPYTFARRISTLDHLTGGRIGWNIVTGYLDSAARNL...GLSQQLGHDERYD
Haladaptatus_cibarius_WP_049970513.1:4-446               AVTRSTTYV.HPYQLAREFSTLDHITDGRVAFNIVTSYLESAAENL...GLDERMEHDTRYD
Arthrobacter_luteolus_WP_066298473.1:1-443               AVTSALTYE.QPYALARRFSTLDHLTGGRIGWNVVTSYLESAARNL...GLERQLGHDERYD
Geobacillus_sp._ZGt-1_WP_047758532.1:1-436               VLTASTTYE.HPFHIARRFSTLDHLTNGRIAWNIVTSYLPNAAKNF...GLERMITHDQRYE
Lysinibacillus_composti_WP_124766485.1:4-458             GVTSSVTHE.HPFTFARKMSTLDHLTNGRVGWNIVTSYLKSAAVNM...GLEDQIKHDERYD
Microcella_putealis_WP_130486201.1:20-474                GVTAGTAYE.HPYPFARRMTTLDHLTKGRVGWNVVTGYLPSAARNM...GHDDQREHDDRYD
Pseudomonas_nitroreducens_WP_037016976.1:1-453           GITASVSFE.HPFPFARRMSTLDHLTRGRAGWNIVTSYLNSGARNL...GLKQQLNHQQRYA
Acinetobacter_wuhouensis_WP_130168784.1:1-453            GVTTSISFE.HPYPFARRISTLDHLTKGRVGWNIVTSYLESGSKNL...GLKTQLNHDDRYD
Bradyrhizobium_sp._YR681_WP_008144663.1:1-454            GVTSNLSFE.PPYPFARRMSTLDHLTEGRIGWNVVTGYLDSAARGA...GKDKQTGHDDRYD
Schumannella_sp._10F1D-1_WP_141164655.1:10-460           GVTAGTAYE.HPYPFARRLTTLDHLTKGRVGWNVVTGYLPSAARNM...GHDDQLEHDDRYD
Pseudomonas_coronafaciens_150_WP_122355187.1:4-45        GLTANLTYD.APYLFARRMSTLDHLSRGRVGWNIVTGYLDSAARAM...GLSEQNEHDRRYD
Pseudomonas_syringae_WP_003430238.1:4-452                GLTANLTYD.APYLFARRMSTLDHLSRGRVGWNIVTGYLDSAARAM...GLSEQNEHDRRYD
unclassified_Rhodococcus_WP_094682261.1:1-449            GITAGTGFE.HPYPFARRLSTLDHLTNGRIGWNVVTGYLPSAAQNM...GHDDQLKHDDRYD
Hafnia_alvei_WP_046449330.1:6-449                        GVTANLSYE.QPYLFARRMSTLDHLTQGRAGWNIVTGYLDSAARAM...GQTQQIPHDRRYD
AS9A_3890_Hoyosella_subflava_WP_013808677.1:1-453        GITTGTGFE.HPYPFARRLSTLDHLTKGRIGWNVVTGYLPSAARNF...GAADQLDHDERYD
Acinetobacter_baumannii_WP_000995600.1:1-443             GVTTSISFE.HPYPFARRISTLDHLTKGRVGWNIVTSYLESGSKNL...GLKTQVNHDNRYD
Agrobacterium_sp._B133/95_WP_065696417.1:2-454           GVTVNTTYE.PPFLFARGMSTLDHLTKGRIGWNIVTGYLDSAARSM...GMERQPDHDARYD
Microbacterium_sp._KROCY2_WP_024289149.1:1-453           GVTSAVTYE.QPYTLARKYATLDHLTRGRVGFNVVTSYLPSAAENQ...GLPTQIEHDERYE
Variovorax_paradoxus_WP_012747040.1:6-452                GVTVNLTYE.QPYLLARRFSTLDHLTRGRIGWNIVTGYLDSAARAM...GLAEQIAHDERYE
Methylobacterium_radiotolerans_WP_076730229.1:5-4        GVTINVHQE.APYTFARRLSTLDHLTGGRIGWNIVTGYLDSAHRAQ...SGGTLPAHDTRYD
Streptomyces_sp._BK215_WP_132915265.1:7-452              AFTGSILQE.HPFAFARKVSTLDHLTKGRVAWNIVTSFQSSAGRNF...GFDTQVPHDERYV
Geobacillus_thermodenitrificans_160_WP_008880416.        VLTASTTYE.HPFHIARRFSTLDHLTNGRIAWNIVTSYLPNAAKNF...GLERMIAHDQRYE
Pseudomonas_aeruginosa_WP_132556951.1:356-785            GVTANLTYE.APYPFARRLSTLDHLSDGRIGWNIVTGYLESAAKAM...GQSAQIEHDRRYD
Penicillium_solitum_OQE02104.1:14-471                    GVTVTTSYE.QPYHLARRLSTVDHLTKGRLGWNVVTGYLDSAARNM...GQAEQLHHDDRYL
Penicillium_vulpinum_OQE05502.1:12-469                   GVTVTTTYE.QPYHLARRLSTVDHLTKGRLGWNVVTGYLDSAARNM...GQAEQLHHDDRYL
Clavibacter_michiganensis_WP_045526194.1:1-454           GLTVASTYE.LPYALARRFSTLDHFTGGRIGWNVVTSYLDSAARNL...GLETQIGHDDRYG
Glutamicibacter_creatinolyticus_WP_054821484.1:6-        GITAGTAYE.HPYPFARRLATLDHLTGGRVGWNVVTGYLPSAARNM...GQEDQMEHDERYD
Campylobacter_sp._P0078_WP_086237522.1:4-454             GITAGVPFE.QPFPFARRLSTLDHLTKGRVGWNVVTGYLPSANANI...GQKE.LPHDERYT
Pseudomonas_furukawaii_WP_003454569.1:1-447              GVTANLSYE.APFTFARRMSTLDHLTGGRVGWNIVTGYLESAARAM...GHGEQETHDRRYD
Leucobacter_triazinivorans_WP_130111548.1:1-442          GITAGTAFE.HPVPFARRLSTLDHLTKGRVGWNVVTGYLPSAARNM...GQADQLEHDQRYD
Aspergillus_brasiliensis_CBS_101740_OJJ68286.1:12        GVTVTTTYE.QPYHLARRLSTIDHLTKGRIGWNIVTGYLDSAARNL...GHTQQPQHDDRYA
Aspergillus_udagawae_170_GAO89440.1:15-472               GVTVTTTYE.QPYHLARRLSTVDHLSKGRLGWNVVTGYLDSAARNL...GHAQQPQHDDRYA
Aspergillus_flavus_RMZ37351.1:15-472                     GVTVTTTYE.QPYHLARRLSTIDHLTKGRIGWNIVTGYLDSAARNL...GHTQQPQHDDRYA
Aspergillus_flavus_RAQ54081.1:15-472                     GVTVTTTYE.QPYHLARRLSTIDHLTKGRIGWNIVTGYLDSAARNL...GHTQQPQHDDRYA
Penicillium_arizonense_XP_022490779.1:12-469             GVTVTTTYE.QPYHLARRLSTVDHLTKGRLGWNVVTGYLDSAARNM...GQAEQPHHDDRYA
Salinibacterium_sp._CGMCC                                GVTAGTAYE.HPYPFARRMTTLDHLTNGRVGWNVVTGYLPSAARNM...GHDDQMEHDERYA
Aspergillus_fumigatus_OXN06785.1:14-471                  GVTVTTTYE.QPYHLARRLSTVDHLSKGRLGWNVVTGYLDSAARNL...GHAQQPQHDDRYA
Aspergillus_lentulus_GAQ10210.1:7-464                    GVTVTTTYE.QPYHLARRLSTVDHLSKGRLGWNVVTGYLDSAARNL...GHAQQPQHDDRYA
Aspergillus_flavus_RAQ66103.1:15-472                     GVTVTTTYE.QPYHLARRLSTIDHLTKGRIGWNIVTGYLDSAARNL...GHTQQPQHDDRYA
Aspergillus_fumigatus_OXN22894.1:14-471                  GVTVTTTYE.QPYHLARRLSTVDHLSKGRLGWNVVTGYLDSAARNL...GHAQQPLHDDRYA
Aspergillus_nidulans                                     GVTVTTTYE.QPYHLARRLSTVDHLTKGRLGWNVVTGYLDSAARNL...GHAEQPQHDDRYA
Penicillium_nalgiovense_OQE79848.1:14-471                GVTVTTSYE.QPYHLARRLSTVDHLTKGRLGWNVVTGYLDSAARNM...GQAEQLHHDDRYL
Aspergillus_wentii                                       GVTASVTYE.KPYALARRLSTVDHLSEGRLAWNIVTSYLDSAARNH...GLKEQIPHDERYA
Gordonia_iterans_WP_105943624.1:1-453                    GITTGTGFE.HPYPFARRLSTLDHLTRGRIGWNVVTGYLPSAARNL...GDDGLLDHDQRYD
Azotobacter_beijerinckii_WP_090735436.1:4-454            GVTASLSYE.PPYLLARRLSTLDHLSRGRVGWNIVTGYLESAARAM...GLEHQVEHDRRYD
Staphylococcus_sp._NAM3COL9_WP_057513692.1:4-453         APTISTTYA.QPYSLARQLSTLDHLTNGRLGWNVVTSYLESEAINL...GLKERLPKDLRYN
Acidovorax_sp._56_WP_099656603.1:11-450                  GVTVNPTTE.HPYLLARRFGTLDHLTTGRIGWNIVTGYLDSAARAI...GLPQQIGHDDRYD
Paenibacillus_sp._BIHB4019_WP_099518466.1:1-456          TMTTSTTYE.APYANARRLSTLDHITKGRIGWNVVTTSSKSAADLF...GRSEFLPHDERYD
Penicillium_antarcticum_OQD90033.1:5-465                 GVTASTTYD.APYALARRFSTVDHLSKGRVGWNIVTSYLDSAARNF...GLDTQVEHDERYR
Aspergillus_oryzae_RIB40_190_XP_001823703.1:9-463        GVTASVTYE.KPYALARRLSTVDHLSEGRLAWNIVTSYLDSAARNH...GLREQIPHDERYA
Fusarium_oxysporum_f._sp._pisi                           GVTVATTYE.QPYHLARRLSTVDHLTKGRLGWNVVTGYLDSAARNL...GHTEPSHDERYA
Colletotrichum_fructicola_Nara_gc5_ELA35345.1:18-        GVTASLTYE.KPYSLARRLSTVDHLSEGRLAWNIVTSYLDSAARNH...GLQEQIPHDERYA
Micrococcus_luteus_WP_041104460.1:19-468                 GVTAGTAYE.HPYPFARRLATLDHLTGGRVGWNVVTGYLPSAAQNM...GQDDQMEHDERYE
Aspergillus_awamori_GCB21923.1:12-469                    GVTVTTTYE.QPYHLARRLSTIDHLTKGRIGWNIVTGYLDSAARNL...GHTQQPQHDDRYA
Aspergillus_niger_CBS                                    GVTVTTTYE.QPYHLARRLSTIDHLTKGRIGWNIVTGYLDSAARNL...GHTQQPQHDDRYA
Aspergillus_lacticoffeatus_CBS_101883_XP_02545389        GVTVTTTYE.QPYHLARRLSTIDHLTKGRIGWNIVTGYLDSAARNL...GHTQQPQHDDRYA
Aspergillus_wentii_DTO_134E9_OJJ40237.1:14-471           GVTVTTTYE.QPFHLARRLSTIDHLTKGRVGWNVVTGYLDSAARNL...GHAQQPQHDDRYA
Aspergillus_indologenus_CBS_114.80_PYI31207.1:11-        GVTVATTYE.QPYHLARRLSTVDHLTKGRVGWNVVTGYLDSAARNL...GLTEQPQHDTRYE
Aspergillus_fischeri_NRRL_181_XP_001264002.1:14-4        GVTVTTTYE.QPYHLARRLSTVDHLSKGRY...VVTGYLDSAARNL...GHAQQPQHDDRYA
Penicillium_coprophilum_200_OQE46388.1:11-466            GVTASVTYE.KPYALARRLSTVDHLSQGRLAWNIVTSYLDSAARNH...GLNEQIPHDERYA
Cupriavidus_taiwanensis_WP_116379876.1:8-454             GVTVNLSYE.QPYLLARRFSTLDHLTRGRVGWNIVTGYLDSAARAM...GLEGQLAHDERYD
Penicillium_digitatum                                    GVTASTTYD.SPYALARRFSTVDHLSNGRIGWNIVTSYLDSAARNF...GLDTQVEHNERYR
Aspergillus_niger_GAQ46262.1:12-469                      GVTVTTTYE.QPYHLARRLSTIDHLTKGRIGWNIVTGYLDSAARNL...GHTQQPQHDDRYA
Aspergillus_nomius_NRRL_13137_XP_015412385.1:15-4        GVTVTTTYE.QPYHLARRLSTIDHLTKGRVGWNIVTGYLDSAARNL...GHTQQPQHDDRYA
Aspergillus_aculeatus_ATCC_16872_XP_020055010.1:1        GVTVATTYE.QPYHLARRLSTVDHLTKGRVGWNVVTGYLDSAARNL...GLTEQPQHDTRYA
Penicillium_brasilianum_OQQ84969.1:19-474                GVTASLTYE.KPYALARRLSTVDHLSEGRVAWNIVTSYLDSAARNH...GLKDQIPHDERYA
Penicillium_subrubescens_OKP15100.1:19-474               GVTASLTYE.KPYALARRLTTVDHLSEGRVAWNIVTSYLDSAARNH...GLKDQIPHDERYA
Trichoderma_arundinaceum_RFU80530.1:19-474               GVTASLTYE.KPYALARRLSTVDHLSDGRVAWNIVTSYLDSAARNH...GLKEQIPHDERYA
Aspergillus_thermomutatus_210_XP_026614594.1:15-4        GVTVTTTYE.QPYHLARRLSTVDHLSKGRLGWNVVTGYLDSAARNM...GHAQQPQHDDRYA
Serratia_liquefaciens_WP_044551273.1:4-449               GITANLSYE.APYPFARRLSTLDHLTQGRIGWNIVTGYLDSAARAM...GLNEQVHHDRRYD
Micrococcus_luteus_WP_065572847.1:19-468                 GVTAGTAYE.HPYPFARRLATLDHLTGGRVGWNVVTGYLPSAAQNM...GQDDQMEHDERYE
Halolamina_rubra_WP_049979911.1:1-449                    AITKSTTYN.HPYQLAREFSTLDHLTDGRVAFNVVTSYLESAADNL...GLDERMEKETRYE
Acinetobacter_pittii_WP_075382847.1:7-452                GVTVNLSYE.HPYQFARRFASLDHLTQGRIGWNIVTGYLDSAERLI...GQKGLKDHDARYE
Corynebacterium_variabile_WP_052302534.1:2-464           GLTTGTAFE.HPFPFARRISTLDHLTDGRLGWNVVTGYLPSAAENTQSRGQLDTFEHDTRYD
Aspergillus_luchuensis_GAT21254.1:12-471                 GVTVTTTYE.QPYHLARRLSTIDHLTKGRIGWNIVTGYLDSAARNL...GHTQQPQHDDRYA
```

```
Aspergillus_luchuensis                                  GVTVTTTYE.QPYHLARRLSTIDHLTKGRIGWNIVTGYLDSAARNL...GHTQQPQHDDRYA
Aspergillus_bombycis_XP_022386026.1:15-472              GVTVTTTYE.QPYHLARRLSTIDHLTKGRVGWNIVTGYLDSAARNL...GHTQQPQHDDRYE
Agrobacterium_vitis_WP_070163524.1:1-454                GITASTSFE.HPYTFARRLATADHHTKGRVGWNIVTSYLESGAKNV...GQSGLKRHDNRYE
Pseudomonas_psychrotolerans_220_WP_074584491.1:5-       GLTANLTYE.PPYPFARRLSTLDHLSEGRVGWNIVTGYLDSAARAM...GLDRQIAHDRRYD
Micrococcus_yunnanensis_WP_135040276.1:19-468           GVTAGTAYE.HPYPFARRLATLDHLTGGRVGWNVVTGYLPSAAQNM...GQDDQMEHDERYE
Acinetobacter_calcoaceticus_WP_004642549.1:7-452        GVTVNLSYE.HPYQFARRFASLDHLTQGRIGWNIVTGYLDSAERLI...GQKGLKDHDARYE
Penicillium_brasilianum_CEO59775.1:15-472               GVTVTTTYE.PPYHLARRLSTVDHLTKGRLGWNVVTGYLDSAARNL...GKAEQPHHDDRYA
Aspergillus_cristatus_ODM22551.1:12-469                 GVTVTTTYE.PPYHLARRLSTVDHLTKGRVGWNVVTGYLDSAARNM...GYAHQPNHDDRYA
Pseudomonas_taiwanensis_WP_027907363.1:1-446            GVTFSLTYE.PPYPFARRMSTLDHLSNGRVGWNIVTGYLDSAARNL...GLARQLGHDQRYD
Acidovorax_avenae                                       GVTVNLSYE.QPYLLARRFSTLDHLTRGRVGWNIVTGYLDSAARAM...GVAQQMPHDERYD
Staphylococcus_pseudintermedius_WP_101431388.1:4-       APTVSTTYA.QPYKLARQLSTLDHLTNGRLGWNVVTSYLESEAVNL...GLKERLPKALRYS
Rhodococcus_triatomae_WP_081607437.1:2-451              AITGSTTYE.QPYSFARRMTTLDHLTGGRVGWNIVTSATASAARNL...GLEQQVAHDERYA
Alcaligenes_sp._RS4_230_WP_128393375.1:4-450            GVTANIHTE.TPYSFARKVSSLDHLTRGRLGWNVVTGYLDSGARAM...GRDGLDEHDKRYD
Gibbsiella_quercinecans_WP_121552947.1:7-454            GLTANVSYE.SPYLFARRFSTLDHLSQGRVGWNIVTGYLDSSARAM...GQQQLVAHDRRYD
Acinetobacter_ursingii_WP_044435211.1:5-452             GITVNLSYE.PPYQFARRFASLDHLTQGRIGWNIVTGYLDSAERLI...GHSGLKDHDLRYE
Erwinia_typographi_WP_034887712.1:4-447                 GVTANLSYE.APYPFARRLSTLDHLTGGRIGWNIVTGYLDSAARAM...GQRQLLAHDRRYD
Trichoderma_harzianum_KKP01913.1:14-470                 GVTIATTYE.QPYHLARRLSTVDHLSGGRVGWNIVTGYLDSAARNL...GLTEQPPHDERYA
Fusarium_graminearum_235_CEF79766.1:12-466              GVTVSTTYE.QPFHLARRLSTLDHLTKGRVGWNIVTSYLDSAARNL...GREEQLSHDERYA
Gordonia_alkanivorans_WP_006357654.1:10-461             GVTTATGFE.HPYPFARRMSTLDHLTRGRVGWNVVTGYLPSGARNM...GDDDQLAHDDRYD
Acinetobacter_radioresistens_WP_111281495.1:5-455       GITVNLSYE.TPYQFARRFASLDHLTHGRIAWNIVTGYLDSAERLI...GQKGLKQHDLRYA
Mesorhizobium_plurifarium_WP_073985566.1:5-458          AITVSTTYE.QPYALARKLTTLDHLTGGRIGWNIVTSALESAARNL...GHNTQIPHDERYA
Oceanobacillus_halophilus_WP_121203049.1:1-454          APTISATYA.QPYSLARQLSTLDHLTEGRIAWNVVTSYLESEAINL...GLSGRLPKELRYD
Acinetobacter_tandoii_240_WP_100241535.1:7-457          GVTVNLSYE.NPYQFARRFASLDHLTQGRIGWNIVTGYLDSAERLI...GQQGLKEHDLRYA
Burkholderia_plantarii_WP_042624083.1:1-457             GLTTSVAYE.APYLLARRFTTLDHLSGGRIAWNIVTSYLQSGARSL...GDDALLAHDARYE
Alcaligenes_faecalis_WP_094195208.1:6-457               GLTASVAAH.HPYTFARDVSTLDQLSNGRIGWNIVTGYVDSGARGL...GQDGLDAHDSRYD
Bradyrhizobium_arachidis_WP_092214516.1:1-453           GVTAPVSYE.PPFTLARRFSTLDHLSKGRIAWNIVTGYSNTASRAV...GRDSIMAHDPRYD
Cupriavidus_plantarum_WP_109583068.1:1-458              GVTLSVSYE.HPYPLARRLTTLDHLSKGRVAWNIVTSYLDSGARSL...GLDALVEHDARYD
Variovorax_sp._770b2_245_WP_093443018.1:5-461           GITATISAE.SPHTFARRMSTLDHLSEGRIGWNIVTGFLDSSARAL...GQSAPLSHDARYD
RZJ22128.1:4-447                                        IATASTTYE.NPYLLARKFASLDHISKGRAAWNVVTTASPDTARNF...GLEQHPDPKTRYE
LadB_Geobacillus_thermoleovorans_BAM76371.1             LFAARPGAT.SPAIFAKQLATINCWTNGRALPNIVTGGSPEELA....AEGDFLDHDTRYE
LadAbeta_Geobacillus_thermoleovorans_BAM76372.1         AGTASTTYN.EPFHIARKFSSLDHISRGRAAWNIVTSYYEEEAYNF...SQTAHLEHRLRYE
Af1_AFLA_024140_XP_002373925.1                          GVTVTTTYE.QPYHLARRLSTIDHLTKGRIGWNIVTGYLDSAARNL...GHTQQPQHDDRYA
Af3_AFLA_58870_XP_002378631.1                           GVTASTTYD.APYALARRFSTVDHLTNGRVGWNIVTSYLDSAARNF...GLNTQVEHDERYR
Af4_AFLA_049780_XP_002383105.1                          GITASTSFE.PPFLLAKRFSTLDHLTNGRIGWNIVTSWKKAAFKAI...GLDTPIEHDERYR
Af5_AFLA_126060_XP_002381799.1                          GITASTTYETSPYALARKFSTLDHLTQGRVGWNIVTSFLDSAAKAY...GMDEQIPHDERYA
Af2_QMW32876.1:9-463                                    GVTASVTYE.KPYALARRLSTVDHLSEGRLAWNIVTSYLDSAARNH...GLKEQIPHDERYA
Yarrowia_lipolytica_CLIB122_YALI0C07546p_XP_50156       GITVSTSYT.PPFTLARQFSTLDHLTDGRIGWNIVTSNSDSASRNF...GLEHQVEHDDRYK
Rhizobium_sp._CF122_WP007794079.1                       VATASTSYN.EPYHIARKFASLDHISGGRAGWNLVTSSSEHEAHNF...GREEHYAHAERYE
Acinetobacter_sp._NIPH_2100_LLM                         IATASTTYE.EPYLLARKFASLDHLSHGRAGWNVVTSASEAAAANF...GYEQQIPHAERYE
Nostoc_sp._PCC_7107_WP_015115963.1                      IATASTTYE.EPYTLARKFASLDHLSNGRAGWNVVTTGNENAAANF...GLEHHPEHSQRYE
Aspergillus_flavus_NRRL3357_uncharacterized             AATASTTYD.EPYHIARRFASLDHLSSGRAAWNIVTTGNPESAKNF...GLDAHVEHADRYK
Paenibacillus_curdlanolyticus_WP006037998.1             AGTVSTTYN.EPFHVARKFASLDHLSEGRAAWNVVTSGSDAEARNF...NLEKHVQHDLRYE
LadAbeta_Geobacillus_thermoleovorans_FMN-dependen       AGTASTTYN.EPFHIARKFSSLDHISRGRAAWNIVTSYYEEEAYNF...SQTAHLEHRLRYE
SsuD_1M41                                               LVALRPSVT.SPTVAARQAATLDRLSNGRALFNLVTGSDPQELA....GDGVFLDHSERYE
Paenibacillus_curdlanolyticus_WP006037998.1             AGTVSTTYN.EPFHVARKFASLDHLSEGRAAWNVVTSGSDAEARNF...NLEKHVQHDLRYE
Bacillus                                                LVAVRPGIM.APSVAARMTSTLDRISNGRLLVNVVAGGDPAELA....GDGLFLSHDERYE
Pseudomonas                                             LVAIRPGIV.SPTVSARMAATLDRLSGGRLLINVVTGGDPDENR....GDGIHLGHAERYE
LadB_Geobacillus_thermoleovorans_FMN-dependent          LFAARPGAT.SPAIFAKQLATINCWTNGRALPNIVTGGSPEELA....AEGDFLDHDTRYE
Pseudomonas_aeruginosa_PAO1_NP_251290.1                 LLAYRPGVIAAPLA.ARQLATLDQFSEGRLALNVVSGGNDEDLA....RDGDYLDHDRRYA
Alkanesulfonate                                         VIALRPNTL.YPTVAAKALATLDQLSSGRAVVHFIAGGSDAEQA....REGDFLTKDERYA
Alkanesulfonate                                         IIALRPNTL.YPTVAAKALATLDQLSSGRVVVHFIAGGSDAEQA....KEGDFLTKEQRYA
Aspergillus_niger_ATCC13496_RDH19503.1                  IIALRPNTL.YPTVAAKALATLDQLSRGRAVVHLIAGGSDAEQA....KEGDFLTKSQRYG
Penicillium_digitatum_Pd1_XP014534980.1                 IIALRPNTL.YPTVAAKALATLDQLSRGRVVVHFIAGGSDAEQA....KEGDFLSKDERYG
Acinetobacter_rudis_WP_016657872.1                      LVAIRPGII.SPTVSARQAATLDRLSNGRLLINVVSGGDPDE..NY...GDGLFLDHTERYE
```

```
160       170       180       190       200       210
LADA_Geobacillus_thermodenitrificans_WP_011888513 LADEYLEVCYKLWEGSWEDNAVIRDIEN....NIYTDPSKVHEINHSGKYFEVPG.PHLCEP
Geobacillus_sp._LEMMY01_WP_079935807.1:1-440 LADEYLEVCYKLWEGSWEDNAVIRDIEN....NIYTDPRKVHEINHSGKYFEVPG.PHLCEP
Geobacillus_stearothermophilus_ABV66256.1:1-440 LADEYLEVCYKLWEGSWEDNAVIRDIEN....NIYTDPSKVHEINHSGKYFEVPG.PHLCEP
Geobacillus_sp._B4113_201601_WP_061912600.1:1-440 MADEYLEVCYKLWEGSWEDNAVIRDIDN....NIYTVPSKVHKINHSGKYFEVPG.PHLCEP
Geobacillus_sp._B4113_201601_5WP_061912600.1:1-44 MADEYLEVCYKLWEGSWEDNAVIRDIDN....NIYTVPSKVHKINHSGKYFEVPG.PHLCEP
Bacillus_thuringiensis_6WP_029441578.1:1-435 LADEFLEVCYKLWEISWEDDAVIRDREN....KIYTDPNKVHQINHIGKYFNVPG.PHLSEP
Lysinibacillus_sphaericus_WP_103976887.1:2-422 LADEYLDVCYKLWEESWEDDAVSIEKNM.....YYADPTKVHKINHKGKYFDVIG.PHLCEP
Lysinibacillus_sphaericus_WP_069508309.1:2-422 LADEYLNVCYKLWEESWEDDAVSIEKNM.....YYADPTKVHKINHKGKYFDVIG.PHLCEP
Rummeliibacillus_sp._SL167_10_WP_146551437.1:2-42 LAEEFLEVCYKLWELSWDNDAVSLENND.....CYASPSKVHKINHKGKYFNVPG.PHICEP
Paenibacillus_sp._DXL2_WP_110843609.1:1-459 IADEYLEVVYKLWESSWEDDAVVRDVET....QTYTDPDKVHTIDHEGRYFRVPG.PHLSEP
Paenibacillus_naphthalenovorans_WP_074730028.1:1- IAEEYMDVSYKLWEGSWEDDAVIRDVQK....QIYTDPDKVHFIHHEGKYFKVPG.PHLSQP
Cohnella_sp._HS21_WP_130606586.1:1-453 VADEYLDVVYKLWEASWEDDAVIRDAAR....GIYTDPSKVHEINHQGKYYNIPG.PHLSEP
Streptosporangium_subroseum_15_WP_089205784.1:1-4 IAEEYLEVLYKLWEGSWEDDAVVRDRER....HVYADPTKVHPIDHVGKHFRVAG.PHLSEP
Arthrobacter_sp._Hz2_WP_120147654.1:1-455 IADEYLDVLYKLWEGSWDDDAVIEDRER....RIYTDPSKVRYINHRGPHFSVAG.PHLSAP
Nonomuraea_solani_WP_103963668.1:1-453 IAEEYLEVLYKLWEGSWEDGAVLRDRDR....HVYTDPAKVHPIDHVGKHFRVAG.PHLSEP
Arthrobacter_ruber_WP_105030338.1:1-455 IADEYLEVLYKLWEGSWDDDAVIEDRDR....RIYTDPSKVRSIDHRGRHFSVAG.PHLSSP
Bacillus_aciditolerans_20_WP_121446947.1:1-456 LADEFLEVSYKLWEGSWEDNAVIADASK....EVLIDPLKVHEINHKGDYFSVEG.PHLSEP
WP_137163763.1:1-458 LAEEYLQVLYKLWEKSWDDDAVLLERDS....GRYIEPARVHPINHVGEHFQVPG.MHLCQP
Aeribacillus_pallidus_WP_130157503.1:4-457 IAEEYLEVCYKLWEGSWEDDAVIKDRDR....KIFTDPAKVHEINHHGKYFNVPG.IHLCEP
Bacillus_megaterium_24WP_098602848.1:4-460 IADEYLEVCYKLWEGSWEDGAVLKDKEK....RVFTDPTKVHEINHFGKYFKVPG.IHLCEP
Streptomyces_sp._150FB_WP_078877664.1:5-455 IADEYLEVLYKLWEGSWDDDAVIQDVDN....RVYTDPAKVRYIDHAGRYYKVAG.PHLSQP
Arthrobacter_sp._DWC3_WP_104051071.1:1-454 IADEYLDVLYKLWEGSWDDDAVVEDREK....RVYTDPSKVRYINHRGPHFSVAG.PHLSAP
Cohnella_thermotolerans_WP_027093898.1:1-456 LADEFMEVAYKLWEASWEKDAIVNDVSR....NMFADPSKVHEINHEGKFFKVPG.PHLSEP
Paenibacillus_bovis_28WP_144921429.1:1-452 IAAEYLEVCYKLWESSWEDDAVKVDKEN....RIYTDPDKVHDINHEGKYYKVPG.AHLCEP
Bacillus_aciditolerans_WP_121447760.1:1-452 IADEYLEVCYKLWEGSWEDDAVLLDKQN....RIFADPSKVHDINHEGKYYKVPG.AHLCEP
Leifsonia_sp._ALI-44-B_30_WP_077053645.1:1-455 IADEYLDVLYKLWEGSWDDDAVVQDREN....RIYTDPSKVRYIDHVGEHFRVKG.PHLSEP
Parageobacillus_thermoglucosidasius_WP_064552269. IADEFLEVAYKLWEGSWEDDAFMEDKQN....GILINPDKVHEINHVGKFFSVEG.PHLCEP
Geobacillus_sp._Y4.1MC1_WP_013399886.1:1-459 IADEFLEVTYKLWEGSWEDDAFIEDKQN....GILIDPDKVHEIHHAGKFFAVEG.PHLCEP
Bacillus_methanolicus_35WP_004434428.1:1-462 IADEYLEVCYKLWELSWEDGAVIEDVKN....GILVDPSKVHEINHSGEFFHVEG.PHLSEP
Terribacillus_halophilus_WP_077309754.1:1-448 IAEEYVEVCYKLWEGSWEDDAVVRDTER....KVYTDPEKVHDIHHNGKYYQVPG.AHLSEP
Paraburkholderia_kururiensis_WP_017777532.1:1-453 LADEYLEVCYKLWESSWDDDAVVRDAAR....HVFTEPSKVHPIAHRGRFFDVPG.IHLCEP
Gracilibacillus_massiliensis_WP_058308419.1:1-456 IADEYLDVSYKLWEQSWEDNAVVEDVEN....ETLVDPEKVHEINHKGHYFKVEG.PHLSEP
Streptomyces_yanglinensis_WP_103888551.1:5-452 IADEYMEVLYKLWEGSWEDDAVIQDVDN....RVYTDPSKVHYIDHAGRYYKVAG.PHLSQP
Trinickia_sp._7GSK02_40_WP_136896361.1:1-452 LADEYLEVCYKLWEASWEDDAVVRDAAR....QVFTEPSKVHPIGHQGRYFDVPG.IHLCEP
Cryobacterium_sp._NEAU-85_WP_123045138.1:1-448 LAEEFLEVSYKLWESSWEPDAVVRDRGR....KLYSEPSKVHEVNHEGRFFTVDG.PHLSEP
Alicyclobacillus_acidoterrestris_WP_021298468.1:4 RAEEFLEVCYKLWESSWEDDAVIRDKAS....GMYTNPRKVHDIEHRGKYSSVPG.IHLCEP
Oceanobacillus_senegalensis_WP_085994229.1:1-452 MAEEYMEVCYKLWEGSWENDAVVRDEER....KLYTDPAKVHDIGHHGKYYHVPG.AHLSEP
Bacillus_sinesaloumensis_WP_077620983.1:1-449 IADEFLDVSYKLWEQSWEDDAVVEDVKN....KTLVDPDKVHEINHEGLYFKVEG.PHLSEP
Halopenitus_persicus_WP_021074147.1:6-457 LAEEYIEVCYKLWEQSWESDAVVKDPET....ETFTDPEKVNEINHDGEYFTVPG.PHLTEP
Microbacteriaceae_bacterium VAEEFLEVSYKLWETSWEPDAVERNAET....GRYTDPSKVHEIGHEGRFFRVNG.PHLSEP
Lysinibacillus_sinduriensis_WP_036201100.1:1-458 IADEFLEVSYKLWESSWENNAFIGDTEI....GILFDPKKVHEINHNGTFFSVKG.PHLTYP
Bacillus_endophyticus_WP_113749053.1:5-457 IADEYVEVCYKLWEGSWEENAVIKDKEK....KVFTNPDKVHAINHHGKYFNVPG.IHLCEP
Pseudomonas_monteilii_WP_119371117.1:1-455 YADEYLEVLYKLFEGSWEDGAVVRDRET....GLFTDPRKVHEIRHHGKHFQVPG.IHLCEP
Bacillus_endophyticus_50_WP_061801645.1:5-457 IADEYVEVCYKLWEGSWEENAVIKDKEK....KVFSNPDKVHAINHHGKYFNVPG.IHLCEP
Planococcus_maitriensis_WP_112233285.1:1-457 FAAEYLDVCYKLWEGSWEDGAVVRDKQS....KVYTDPSKVHDIRHEGTYFKVPG.AHLSEP
Oceanobacillus_profundus_WP_118889880.1:1-447 IAEEYLEVCYKLWEESWEDDAVKLDKEN....RVFTDPSKVHDINHQGKYYRVPG.AHLCEP
Burkholderia_pseudomallei_WP_141404579.1:1-452 IADEYLEVCYKLWEGSWEDGAVVRDAAR....RVFAEPSKVHPIDHRGRYYDVPG.IHLCEP
Terribacillus_saccharophilus_WP_095261886.1:1-455 IADEFLEVSYKLWESSWEEDAVVEDLQN....QVLVKPEKVHEINHEGQYFQVEG.PHLSEP
Sediminibacillus_albus_WP_093212831.1:4-453 IAAEYLDVCFKLWEESWEEDAVLLDKER....GIYSDPAKVHDINHAGKYFKVPG.AHLSEP
Sciscionella_marina_WP_020496929.1:1-448 LAEEFLEVSYKLWEGSWEEDAVLHDRAE....RRYSDPAKVHEISHRGRYFTVDG.PHLSEP
Mycetocola_saprophilus_WP_043321267.1:1-452 VAEEFLDVSYKLWEGSWDRDAVIRDRER....AIYTNPRGVHEIGHVGKYFTVDG.PHLSEP
Lysinibacillus_macroides_WP_053993569.1:4-460 IADEYLEVCYKLWEGSWEEQAVIRDKET....GIFTDPTKIHEIQHKGKYFDVPG.IHLCEP
Pseudomonas_putida_WP_086978777.1:1-458 YADEYLEVLYKLFEGSWEDGAVVRDRET....GLFTDPRKVHEIRHQGKHFQVPG.IHLCEP
Sediminibacillus_halophilus_60_WP_074598704.1:4-4 IAAEYVDVCYKLWEGSWEDDAVRLDKEN....GIYTDPEKVHDIQHEGKYFKVPG.AHLSEP
Rhizobium_oryzae_WP_085420425.1:1-450 LADEYMEVMYRLWEGSWEDDATVFEREK....RIYADPSKVHKIRHHGRQYRIDT.VHLCEP
Pseudomonas_extremorientalis_WP_071491956.1:1-455 YAEEYLEVCYKLWEGSWEEGAVLRDRER....RIFSDPSKIHEIRHVGKHFQVPG.IHLCEP
Pseudomonas_fluorescens_WP_016977452.1:1-454 YAEEYLEVCYKLWEGSWEEGAVLRDRER....RIFSDPSKIHEIRHVGKHFQVPG.IHLCEP
Neorhizobium_sp._T25_27_WP_105419890.1:1-450 LADEYMEIMYRLWEGSWEDDAIVFDQAN....HVYADPAKVHKVHHRGKQYGIEA.IHLCEP
Pseudomonas_extremaustralis_WP_078833627.1:1-457 YAEEYLEVCYKLWEGSWEDGAVLRDRER....RIFSDPSKIHEIHHVGKHFQVPG.IHLCEP
Fictibacillus_aquaticus_WP_094253544.1:1-455 IADEFLEVSYKLWEESWEDGAIIEDVEN....GILVDSSKVHEINHTGEFFSVEG.PHLSEP
Pseudomonas_extremaustralis_KAA8559760.1:1-456 YAEEYLEVCYKLWEGSWEEGAVLRDRER....RIFSDPSKIHEIRHVGQHFQVPG.IHLCEP
Mycobacterium_sp._ACS1612_WP_067812332.1:1-454 IADEYTEVLYKLWEGSWEDDAVGVDPTR....AEYADPTKVHPIEHESKHFRVPG.IHLCEP
Bacillus_megaterium_WP_116075167.1:1-455 IADEFLEVCYKLWEGSWEEGAVVKDKEK....GIFTLPDRVHPINHKGKWFSVPG.VHLSEP
Pseudomonas_frederiksbergensis_70_WP_123409596.1: FAEEYLEVCYKLWEGSWEEGAILRDRER....RIFSDPSKIHEIRHVGKHFQVPG.IHLCEP
Pseudomonas_citronellolis_SFC61163.1:1-452 IADEYLDVLYKLWEGSWDDDAVIADRQL....RIYTDPDKVRYIDHVGEHFRVAG.PHLCQP
Herbaspirillum_chlorophenolicum_WP_050468599.1:4- RADEFMEVVYKLWEGSWEDGAVVRDRAN....RIYADPSKVHKIRHSGDYYRVEG.IHLSEP
Arthrobacter_crystallopoietes_WP_074700854.1:1-45 IAEEYLDVSYKLWEGSWDDGAVVRDRER....RVYTETDGVHEIGHAGRFFTVDG.PHLSEP
Terribacillus_goriensis_WP_038558686.1:1-438 IADEFLEVSYKLLEDSWEEDAVISDTET....GVLVDPEKVHRIKHKGTYFSVEG.PHVSEP
Bacillus_megaterium_WP_025749753.1:1-455 IADEFLEVCYKLWEGSWEEGAVVKDKEK....GIFTLPDRVHPINHKGKWFSVPG.VHLSEP
Neorhizobium_sp._T7_12_WP_105403044.1:1-451 LADEYMEIMYRLWEGSWEDDAIVFDQAN....HVYADPAKVHKVHHRGKQYGIEA.IHLCEP
Rummeliibacillus_sp._SL167_WP_146553619.1:4-459 IADEYLEVCYKLWENSWEDGAVVKDANK....GVFTEPAKVHDIAHHGKYFDVPG.IHLCEP
Burkholderia_sp._PAMC RADEYLDVLYKLWEGSWENDAVVRDRET....RVFADPAKVHKVDHHGRYYDVEG.YHLCEP
Pseudomonas_koreensis_WP_064585463.1:4-449 QADEYLEVLYKLWEGSWENGAVLNDREQ....RIYAQPEKVHKVEHKGEFYQVEG.YHLCEP
Pseudomonas_fluorescens_80_WP_064116961.1:6-451 QADEYLEVLYKLWEGSWENGAVLNDREQ....RIYANPEKVHKVEHKGEFYQVEG.YHLCEP
Pseudomonas_rhodesiae_WP_040265276.1:4-450 QADEYLQVLYKLWEGSWEDDAVLNDPQQ....RVYAQPGKVHKVEHRGEFYQVEG.YHLCEP
Collimonas_sp._OK412_WP_092393355.1:4-454 RAEEFMQVLYKLWEGSWEDGAVLRDRQR....RVYADPAKVHRIAHRGQHYQVDG.YHLSEP
Pseudomonas_marginalis_WP_058415544.1:1-456 YAEEYLQVCYKLWEGSWEEGAVLRDRER....RIFSDPSKIHEIRHVGKHFQVPG.IHLCEP
Pseudomonas_antarctica_WP_064452947.1:1-458 YAEEYLEVCYKLWEGSWEEGAVLRDRER....RIFSDPSKIHEIRHVGKHFQVPG.IHLCEP
Pseudomonas_protegens_WP_041115159.1:9-454 QADEYLEVLYKLWEGSWEDDAVLNDREQ....RIYAQPGKVHKVEHQGEFYQVSG.YHLCEP
Pseudomonas_synxantha_WP_057024776.1:4-449 QADEYLEVLYKLWEGSWEDDAVLNDPQA....RVYAQPGKVHKVEHHGEFYQVEG.YHLCEP
Rhizobium_leguminosarum_WP_128409501.1:2-451 AAEEYLEILYKLWEGSWDDDAVVRDKAG....GTYADPSKVRTVRHDGKYYRMEG.IHLSEP
Pseudomonas_chlororaphis_WP_038359779.1:6-451 QADEYLEVLYKLWEGSWENGAVLNDREQ....RIYAQPEKVHKVEHKGEFYQVEG.YHLCEP
Pseudomonas_syringae_90_WP_024642725.1:1-456 YAEEYLEVCYKLWEGSWEDGAILRDRER....RVFSDPSKIHEIRHVGKHFQVPG.IHLCEP
Collimonas_fungivorans_WP_061540754.1:4-453 RAEEFMQVLYKLWEGSWEDGAVLRDRQR....RVYADPAKVHRISHRGEYYQVDG.YHLSEP
Rhizobium_leguminosarum_WP_116409027.1:2-451 AAEDYLEILYKLWEGSWDDDAVLRDKAG....GIYADPSKVRAVHHDGKYYRMEG.IHLCEP
Microvirga_ossetica_WP_099511173.1:1-454 IADDYLDVLYKLWEGSWDDDAVIADGAK....GIYARPDKVRAIKHDGPYYQIDA.IHLSEP
Pseudomonas_caspiana_WP_087268376.1:4-451 QADEYLQVLYKLWEGSWEDDAVINDREQ....RVYAQPDKVHKVSHTGEFYQVEG.YHLCEP
Pseudomonas_lundensis_WP_048375063.1:4-451 QADEYLEVLYKLWEGSWEDGAMINDPAQ....RIYARPEKVHKVKHQGEFYQVEG.YHLCEP
Caballeronia_mineralivorans_WP_047894899.1:4-456 RADEYLDVTYKLWEGSWEDDAVVRDRAA....RVFADPQKIHKVNHHGRYYNVEG.YHLSEP
Pseudomonas_paralactis_WP_057702387.1:4-450 QADEYLQVLYKLWEGSWEDDAVLNDPQT....RVYAQPGKVHKVEHHGEFYQVEG.YHLCEP
Pseudomonas_taetrolens_WP_048381143.1:4-453 QADEYLEVLYKLWEGSWENDAVINDPAQ....RIYARPEKVHKVKHAGEFYQVEG.YHLCEP
Pseudomonas_psychrophila_WP_123750761.1:4-450 QADEYLEVLYKLWEGSWENDAVLNDPAQ....RIYAQPDKVHKVKHAGEFYQVEG.YHLCEP
Collimonas_arenae_100_WP_061532973.1:4-454 RAEEFMQVLYKLWEGSWEDGAVLRDRQR....RVFADPAKVHPVKHDGRYYQVDG.YHLSEP
Pseudomonas_viridiflava_WP_122210720.1:4-451 QADEYLQVLYKLWEGSWEDDAVINDRER....RVYAQPGKVHKIQHEGEFYQVEG.YHLCEP
Kocuria_varians_WP_068470402.1:1-454 IAEEFLDVCYKLWEGSWEDDAVVKDRES....GVYADPTKVHPVRHAGEHFTVPG.AGLTEP
Pseudomonas_wadenswilerensis_WP_115084816.1:4-455 QADEYLQVLYKLWEGSWEDGAVVNDRQQ....RVYARPDKVHKVNHQGEFYRVEG.YHLCEP
Pseudomonas_syringae_WP_011266344.1:4-452 QADEYLEVLYKLWEGSWEDDAVINDREQ....RVYAQPGKVHKVRHQGEFYQVEG.YHLCEP
Pseudomonas_sp._286_105WP_122415793.1:4-453 QADEYLEVLYKLWEGSWEDDAVINDRER....RVYAQPGKVHKIRHAGEFYQVEG.YHLCEP
Geobacillus_vulcani_WP_031406843.1:1-461 IADECLEVTYKLWEGSWEEGAFLEDRET....GVLIDPAKIHPINHHGAFFSVEG.PHLCEP
Geobacillus_icigianus_WP_033018365.1:1-461 IADECLEVTYKLWEGSWEEGAFLEDRET....GVLIDPGKIHPINHHGAFFSVEG.PHLCEP
Pseudomonas_floridensis_WP_083182568.1:4-453 QADEYLEVLYKLWEGSWEDDAVLNDREQ....RVYAQPGKVHKIRHEGEFYQVAG.YHLCEP
Pseudomonas_asturiensis_WP_073167523.1:4-451 QADEYLEVLYKLWEGSWEDGAVINDREQ....RVYAQPGKVHKVRHAGEFYQVEG.YHLCEP
Geobacillus_vulcani_110_WP_031406843.1:1-461 IADECLEVTYKLWEGSWEEGAFLEDRET....GVLIDPAKIHPINHHGAFFSVEG.PHLCEP
```

```
Pseudomonas_syringae_WP_024682626.1:4-452             QADEYLEVLYKLWEGSWEDDAVINDREQ....RVYAQPGKVHKVRHQGEFYQVEG.YHLCEP
LadAalpha_Geobacillus_thermoleovorans_BAM76377.1      IADECLEVTYKLWEGSWEEGAFLEDRET....GVLIDPAKIHPINHHGAFFSVEG.PHLCEP
Tsukamurella_pseudospumae_WP_068746786.1:1-457        HADEYLEVLYKLWEGSWEDDAVIRDRES....GVFTDPSKVHPIGHHGKYFDVPG.IHVGEP
Kocuria_sp._Marseille-P3598_WP_085529073.1:5-456      IAEEFLDVCYKLWEGSWEDDAVVADREA....GVYADPAKVHPIQHRGEYFSVPG.ASLTAP
Microvirga_aerophila_WP_114188789.1:1-456             VADEFMEVVYKLWEGSWEGDAVVRDRER....RLYARPDKVHRIQHSGRHYNVDA.IHLSEP
Burkholderia_ubonensis_WP_071760483.1:5-455           RADDYMDVVYKLWEQSWDDDAVIRDAHA....RVFAQPGKVRRVKHDGPFYSIDA.IHLCEP
Achromobacter_marplatensis_WP_006226823.1:4-450       RADEFLEVAYKLWEESWDRDAVKADKRA....RVYADPAAVRRVDHEGKYYKVQG.FHLSEP
Acinetobacter_baumannii_WP_070165746.1:1-443          IADEYLEVLYKLWEGSWEEGSVLRDREN....GIFADHKKVHPIQHEGKYFTVPG.IHICEP
Labedella_phragmitis_WP_128496094.1:1-453             VADEYLEVLYKLWEGSWEDDAVVRDRES....GIFTDPDKVHEIGHRGTHFTVPG.IHLSEP
Nocardia_cyriacigeorgica_120_WP_130918184.1:1-453     QADEYLEVLYKLWEGSWEDDAVVRDRER....GIYVDPAKVHHIGHQGKHFTVPG.VHLSEP
Nocardia_cyriacigeorgica_WP_130918184.1:1-453         QADEYLEVLYKLWEGSWEDDAVVRDRER....GIYVDPAKVHHIGHQGKHFTVPG.VHLSEP
Mycobacterium_sp._1164985.4_WP_067298521.1:1-453      HADEYLEVLYKLWEGSWEDDAVVRDAAR....GVFTEPDKVHHIGHQGTHFSVPG.VHLSEP
Rhodococcus_sp._1R11_WP_135042739.1:1-449             HADEYLEVLYKLWEASWDDDAMVRDRET....GVFTDPTKVHRIGHRGKHFTVPG.IHLAEP
Erwinia_amylovora_WP_004157587.1:5-459                YADEYLQVVYKLLEGSWEEGAVLRDRQR....RLFSDPSKIHPINHQGTFFQVPG.IHLCEP
Ralstonia_mannitolilytica_WP_102079134.1:4-451        RADEYLEVLYKLWEGSWEDDAVRRDKAA....RVFADPARVRKVQHSGRYYKVDG.YHLAEP
Leifsonia_flava_WP_135120808.1:15-465                 HADEYLEVLYKLWEGSWEDDAVVRDRET....GVFTDPSKVHEIGHRGKHFTVPG.IHISEP
Cryobacterium_roopkundense_WP_084141147.1:1-453       VADEYLEVLYKLWEGSWEDDAVVRDREA....GIFTDPSKVHEIGHHGKHFTVPG.IHLAEP
Cupriavidus_pauculus_WP_101681197.1:1-455             LADEYMALVYKLWEGSWDDDAVVADRAT....GVYANPEKVRVIHHHGPQYKVDA.MHLCAP
Rhizobium_sp._BK333_130_WP_133704837.1:2-454          AAEEYLEVVYKLWEGSWDDDAVRRDKMG....GLFADPSKVRAVRHEGSHYRMDG.IHLSEP
Caballeronia_mineralivorans_WP_047846789.1:1-454      VAAEYIEVLYKLFEGSWEEDAVVRDRHN....RVFTHPEKVHEIGHHGKYFDVPG.YHLCEP
Nocardia_altamirensis_WP_069163049.1:1-454            QADEYLEVLYKLWEGSWEDDAVVRDAAQ....GIYVDPAKVHHIGHRGKHFTVPG.IHLSEP
Cupriavidus_sp._P-10_WP_116322758.1:1-452             IAEEYMELMYKLWEGSWEDGAVCRDRAR....GIYADPSKVHAVNHDGEYFRLNG.IHLSEP
Serratia_sp._S1B_WP_116726654.1:1-452                 IADEYLEVLYKLWEGSWEQDAVLRDKQR....GIFADHRKVHPIQHQGKYFIVPG.IHICEP
Pseudomonas_putida_WP_043864435.1:1-452               LAEEYLQVCYKLWEASWEDDAVRADRAS....GVYADPAKVHGIGHQGEFFQVPG.FHLCEP
Pseudomonas_aeruginosa_WP_023084248.1:4-453           RADEYLEVLYKLWEGSWEDDAVLANRQR....RVYAQPEKIHKVRHHGDFYQVEG.YHLAEP
Pseudomonas_mucidolens_WP_084381795.1:9-455           QADEYLQVLYKLWEGSWEDDAVLNDPQA....RVYAQPNKVHKIEHQGEFYQVEG.YHLCEP
Pseudomonas_poae_WP_060549803.1:4-450                 QADEYLQVLYKLWEGSWEDDAVLNDPAQ....RIYAQPGKVHKVEHHGEFYQVEG.YHLCEP
Pseudomonas_chlororaphis_WP_123573412.1:9-454         QADEYLQVLYKLWEGSWENDAVINDREQ....RIYARPEKVHKVEHKGEFYQVEG.YHLCEP
Janthinobacterium_lividum_140_WP_072454679.1:1-44     LADEYLDVVYKLWEKSWDDDAVVNDKAR....GIYADPARVHPINHAGRHYQVPG.IHLCEP
Haladaptatus_cibarius_WP_049970513.1:4-446            RAEEFMRVCYRLWEDSWEDDAVIRDVGA....GKYTDPEKVHAIDYEGEHFSVPG.PHSCEP
Arthrobacter_luteolus_WP_066298473.1:1-443            LADEFMEVTYKLWEGSWEEGAVLKDRER....GIYTDPAKVHPINHSGAYYQVPG.IHLAEP
Geobacillus_sp._ZGt-1_WP_047758532.1:1-436            IADECLEVTYKLWEGSWEEGAFLEDRET....GVLIDPAKIHPINHHGAFFSVEG.PHLCEP
Lysinibacillus_composti_WP_124766485.1:4-458          IAEEYLQVCYKLWEASWEDDAVKVDKLN....RIYADPEKVHDIRHEGKYYKVPG.AHLSEP
Microcella_putealis_WP_130486201.1:20-474             HADEYLEVLYKLWEGSWEDDAVVRDRER....GVYTDPEKVHEIGHHGTHFTVPG.IHISEP
Pseudomonas_nitroreducens_WP_037016976.1:1-453        LAAEYLEVCYKLWEASWDDDAVVADRKS....GVYADPAKVHPIEHKGEHFEVPG.FHLSQP
Acinetobacter_wuhouensis_WP_130168784.1:1-453         IADEYLEVLYKLWEGSWENNAVLRDKQA....GIFADHSKVHPIEHEGKYFKVPG.IHICEP
Bradyrhizobium_sp._YR681_WP_008144663.1:1-454         IADEYMEVVYKLWEGSWEDDAVLRDRKR....GIFTDPSKVHRINHESANYRINNTIHLSEP
Schumannella_sp._10F1D-1_WP_141164655.1:10-460        HADEYLEVLYKLWEGSWQDDAVVKDRER....GIFTDPAKVHEIGHQGKHFTVPG.IHISEP
Pseudomonas_coronafaciens_150_WP_122355187.1:4-45     QADEYLEVLYKLWEGSWEDDAVINDTQQ....RVYAQPGKVHKVRHQGEFYQVEG.YHLCEP
Pseudomonas_syringae_WP_003430238.1:4-452             QADEYLEVLYKLWEGSWEDDAVINDREQ....RVYAQPGKVHKVRHQGEFYQVEG.YHLCEP
unclassified_Rhodococcus_WP_094682261.1:1-449         HADEYLEVLYKLWEASWDDDAVVRDRET....GVFTDPTKVHRIEHRGKHFTVPG.IHLAEP
Hafnia_alvei_WP_046449330.1:6-449                     MADEYLDVLYQLWEGSWQEDALLADRQN....RIYADSTKIHSIQHGEFFDVEG.YHLTSP
AS9A_3890_Hoyosella_subflava_WP_013808677.1:1-453     HADEYLEVLYKLWEGSWEDDAVVRDRER....GVYVDADKVHHVGHRGKHFSVPG.IHLSEP
Acinetobacter_baumannii_WP_000995600.1:1-443          IADEFLEVLYKLWEGSWEEGSVLRDRES....GIFADHKKVHPIQHEGKYFTVPG.IHICEP
Agrobacterium_sp._B133/95_WP_065696417.1:2-454        AAEEYLEVVYKLWEGSWDDDAVKRDKAG....GLFADPSKVRAVRHEGSHYRMEG.IHLSEP
Microbacterium_sp._KROCY2_WP_024289149.1:1-453        IAEEFLDVCYKLWEGSWEDDAVVKDVER....GIYADPAKVHPIQHQGKHFTVPG.AALTEP
Variovorax_paradoxus_WP_012747040.1:6-452             RADEYLEVLYKLWEGSWEDAAVRRDKAA....RVFADPARVHKVRHEGRYHQVEG.YHLAEP
Methylobacterium_radiotolerans_WP_076730229.1:5-4     YADEYLEVLYRLWEGSWDDAAVRRDRAA....RVFSDPAHIRKVTHRGAYFAVEG.YHLSEP
Streptomyces_sp._BK215_WP_132915265.1:7-452           WADEYMDVVYKLWEGSWEDGAVLGDRAG....GVYADPSKVHRIDHVGDRYRVAG.PHMCEP
Geobacillus_thermodenitrificans_160_WP_008880416.     IADEFLEVTYKLWEGSWEEGARVEDREA....GVLIDPNKVHSIDHQGDFFTVEG.PHLCEP
Pseudomonas_aeruginosa_WP_132556951.1:356-785         RADEYLEVLYKLWEGSWEDDAVLADRQR....RVYARPEKIHKIRHHGEFYQVEG.YHLAEP
Penicillium_solitum_OQE02104.1:14-471                 LAEEYIKVTYKLWESSWRQDAVTLDRER....GIYTDPAGVRQINHAGKYFNVPG.PHLCQP
Penicillium_vulpinum_OQE05502.1:12-469                LAEEYIKVTYKLWESSWRQDAVTLDRER....GIYTDPAGVREINHVGKYFNVPG.PHLCQP
Clavibacter_michiganensis_WP_045526194.1:1-454        IGDEFLDVASKLWEGSWEDDAVVVDREC....GVYTDPAKVHPIAHEGEHFRVPG.VHLSEP
Glutamicibacter_creatinolyticus_WP_054821484.1:6-     HADEYLEVVYKLLEGSWEDDAVKADKVR....GIYTNPEQVHPINHEGKYFKVPG.IAITEP
Campylobacter_sp._P0078_WP_086237522.1:4-454          QADEYMEVVYKLLEGSWEDNAVILDKNT....GAFADPNKIHPINHKGKYYEVPG.IHICEP
Pseudomonas_furukawaii_WP_003454569.1:1-447           QADEYLEVLYKLWEGSWEDDAVLADRGR....RIYARPDKVHPIRHHGEFYRVEG.YHLSQP
Leucobacter_triazinivorans_WP_130111548.1:1-442       HADEYLEVVYKLLEGSWEDDAVVRDRER....GVFAEPDKVHHIGHHGTHFTVPG.IHLSEP
Aspergillus_brasiliensis_CBS_101740_OJJ68286.1:12     QAEEYIKVTYKLWESSWRSDAVILDRTR....GIYTDPTLVREINHTGKYFSVPG.PHICQP
Aspergillus_udagawae_170_GAO89440.1:15-472            VAEEYVRVTYKLWESSWRDDAVVLDRER....GIYTDPSRVREINHEGKYFNVPG.PHLCQP
Aspergillus_flavus_RMZ37351.1:15-472                  IAEEYIKVTYKLWESSWRSDAVVLDRER....GVYTDPSRVREINHVGKYFSVPG.PHICQP
Aspergillus_flavus_RAQ54081.1:15-472                  IAEEYIKVTYKLWESSWRSDAVVLDRER....GIYTDPSRVREINHVGKYFSVPG.PHICQP
Penicillium_arizonense_XP_022490779.1:12-469          VAEEYLKVTYKLWESSWRQDAVIRDRER....GIYTDPSGVRQINHEGKYFNVPG.PHLTQP
Salinibacterium_sp._CGMCC                             HADEYLEVLYKLWEGSWEDDAVVVDRES....GVFTDPSKVHDINHEGKYFTVPG.IHISEP
Aspergillus_fumigatus_OXN06785.1:14-471               IAEEYVRVTYKLWESSWRDDAVVLDRER....GIYTDPSRVREINHEGKYFNVPG.PHLCQP
Aspergillus_lentulus_GAQ10210.1:7-464                 VAEEYVRVTYKLWESSWRDDAVVLDRER....GIYTDPSRVREINHNGKYFTVPG.PHLCQP
Aspergillus_flavus_RAQ66103.1:15-472                  IAEEYIKVTYKLWESSWRSDAVVLDRER....GVYTDPSRVREINHVGKYFSVPG.PHICQP
Aspergillus_fumigatus_OXN22894.1:14-471               IAEEYVRVTYKLWESSWRDDAVVLDRER....GIYTDPSRVREINHEGKYFNVPG.PHLCQP
Aspergillus_nidulans                                  IAEEYIKVAYKLWESSWRSDAVILDKQR....RVYTEPSRVREINHVGKYFNVPG.PHLCQP
Penicillium_nalgiovense_OQE79848.1:14-471             LAEEYIKVTYKLWESSWRQDAVTLDRER....GIYTDPAGVRQINHVGKYFNIPG.PHLCQP
Aspergillus_wentii                                    IAHEYMEVLYKLWEGSFRDDAVVEDRDA....GVYIASDGVREINHKGKYFDVPG.PHFCEP
Gordonia_iterans_WP_105943624.1:1-453                 HADEYLTVLYKLWEGSWEDDAVLRDRAA....GIFADPAKVHHIGHGEHFRVPG.IHLSEP
Azotobacter_beijerinckii_WP_090735436.1:4-454         QADEYLEVLYKLFEGSWEDGAVLEDRTG....RRYADPAKVHKVEHRGEFYRVEG.YHLCSP
Staphylococcus_sp._NAM3COL9_WP_057513692.1:4-453      RADEFLQVVYKLWEDSWEDGAVVNDKDT....DTYADPDKVHEINHEGEFFSVPG.PHIVEP
Acidovorax_sp._56_WP_099656603.1:11-450               QAEEFLDVFYQLLEGSWRDDAVKADKGA....RIYTDPAAVRKIAHHGHHYQVEG.VNLTAP
Paenibacillus_sp._BIHB4019_WP_099518466.1:1-456       MADEYMDVSYKYWEGSWQDDALLVDREK....RIFADPAKVHKVKHEGKYFKSEG.YHASTP
Penicillium_antarcticum_OQD90033.1:5-465              IADEYLDVTYKLWEASWRDDAVTFARDG.FKNQPYADPKAVRQINHKGKYFQVPG.PHLCEP
Aspergillus_oryzae_RIB40_190_XP_001823703.1:9-463     IAHEYMEVLYKLWEGSFRDDAVVDDREQ....GVYIASDAVRQIHHKGKYFEVPG.PHFCEP
Fusarium_oxysporum_f._sp._pisi                        IAEEYVDVTYKLWQSSWRDDAVKLDREK....GIYTDPSLVRLINHSGKYYTVPG.PHICQP
Colletotrichum_fructicola_Nara_gc5_ELA35345.1:18-     IAHEYVEVLYKLWEGSFRDDAVLADRES....GTYIASDAVSQIHHKGKYFEVPG.PHFVEP
Micrococcus_luteus_WP_041104460.1:19-468              HADEYLDVVYKLLEGSWEDDAVVYDKES....GVFADPAKVHDIAHQGRYFTVPG.HAVTEP
Aspergillus_awamori_GCB21923.1:12-469                 QAEEYIKVTYKLWESSWRSDAVVLDRTR....GIYTDPTRVREINHSGKYFTVPG.PHICQP
Aspergillus_niger_CBS                                 QAEEYIKVTYKLWESSWRSDAVVLDRTR....GIYTDPTRVREINHSGKYFTVPG.PHICQP
Aspergillus_lacticoffeatus_CBS_101883_XP_02545389     QAEEYITVTYKLWESSWRSDAVVLDRTR....GIYTDPTRVREINHSGKYFTVPG.PHICQP
Aspergillus_wentii_DTO_134E9_OJJ40237.1:14-471        VAEEYIKVAYKLWESSWRSDAVVLDKER....GIYTDPSRIREINHAGKYFNVPG.PHICQP
Aspergillus_indologenus_CBS_114.80_PYI31207.1:11-     TAEEYLRVTYKLWESSWRDDAVQLARST....GIYTSPDLVRPINHTGRTFTVPG.PHLCQP
Aspergillus_fischeri_NRRL_181_XP_001264002.1:14-4     IAEEYVRVTYKLWESSWRDDAVVLDRER....GIYTDPSRVREINHEGKYFNVPG.PHLCQP
Penicillium_coprophilum_200_OQE46388.1:11-466         IADEFLEVLYKLWEGSFRDDAVLADRQL....GTYIASDGVREINHKGKYFDVPG.PHFCEP
Cupriavidus_taiwanensis_WP_116379876.1:8-454          RADEFLDVLYKLWEGSWQNDAVLRDKAA....RVFADPAKVRKVSHSGRYYKVDG.YHLSEP
Penicillium_digitatum                                 IADEYLDVTYKLWEASWRDGAVSWKQGSESKTQAYADPKAVREINHKGKYFQVPG.PHLCEP
Aspergillus_niger_GAQ46262.1:12-469                   QAEEYIKVTYKLWESSWRSDAVVLDRTR....GIYTDPTRVREINHTGKYFSVPG.PHICQP
Aspergillus_nomius_NRRL_13137_XP_015412385.1:15-4     IAEEYIKVTYKLWESSWRSDAVVLDRER....GIYTDPSRVREINHVGKYFSVPG.PHICQP
Aspergillus_aculeatus_ATCC_16872_XP_020055010.1:1     TAEEYLRVTYKLWESSWRDDAVQLSRTT....GIYTSPELVRPINHTGPNFTVPG.PHLCQP
Penicillium_brasilianum_OOQ84969.1:19-474             IAHEYMEVLYKLWEGSFRDDAVLADREL....GTYIASDAVLPINHKGKYFEVPG.PHFCEP
Penicillium_subrubescens_OKP15100.1:19-474            IAHEYMEVVYKLWEGSFRDDAVLADRQL....GTYIASDAVLPINHKGKYFEVPG.PHFCEP
Trichoderma_arundinaceum_RFU80530.1:19-474            IAHEYMEVLYKLWEGSFRDDAVLRDREH....GNYIANDGVVQIHHKGKYFEVPG.PHFCEP
Aspergillus_thermomutatus_210_XP_026614594.1:15-4     VAEEYVRVTYKLWESSWRDDAVVLDRAR....GIYTDPSRVREINHEGKYFNVPG.PHLCQP
Serratia_liquefaciens_WP_044551273.1:4-449            QADEFLDVAYQLWEGSWEDGAVLADRQR....RIYADAQKIHKVIHRGEFYQVEG.YHLSSP
Micrococcus_luteus_WP_065572847.1:19-468              HADEYLDVVYKLLEGSWEDDAVVYDKES....GVFADPAKVHDIAHEGTWFKVPG.HAVTEP
Halolamina_rubra_WP_049979911.1:1-449                 RAAEFLDVCYALWEDSWEDDAVLRDAES....GRYTDPEKVHSIDHEGEFFDVPG.PHGSEP
Acinetobacter_pittii_WP_075382847.1:7-452             QAEEFLELCYKYWEGSWENDAVKKDKAR....RVFTDPSKVHTIHHHGKYYQSEG.VFQVSP
Corynebacterium_variabile_WP_052302534.1:2-464        HADEYLEVLYKLWEGSWDQDAVLADPVS....GVYTDPAKVRPINHAGTHYTVPG.IHVSEP
Aspergillus_luchuensis_GAT21254.1:12-471              QAEEYIKVTYKLWESSWRSDAVVLDRTR....GIYTDPTRVREINHTGKYFTVPG.PHICQP
```

```
Aspergillus_luchuensis                              QAEEYIKVTYKLWESSWRSDAVVLDRTR....GIYTDPTRVREINHTGKYFTVPG.PHICQP
Aspergillus_bombycis_XP_022386026.1:15-472          IAEEYIKVTYKLWESSWRSDAVVLDRER....GVYTDPSRVREINHVGKYFSVPG.PHICQP
Agrobacterium_vitis_WP_070163524.1:1-454            IANEYLEVIYKLLEGSWEEGAIIRDPKG....RVFTDPAKVHEIGHKGKHFDVPG.YGLTEP
Pseudomonas_psychrotolerans_220_WP_074584491.1:5-   QADEYLEVLYKLLEGSWADDAVVRDRVQ....RIYARPERVRKVAHHGEFFDVEG.YHLCEP
Micrococcus_yunnanensis_WP_135040276.1:19-468       HADEYLDVVYKLLEGSWEDDAVVYDKES....GVFADPAKVHDIAHEGTWFKVPG.HAVTEP
Acinetobacter_calcoaceticus_WP_004642549.1:7-452    QAEEFLELCYKYWEGSWENDAVKKDKVQ....RVFTDPSKVHTIHHHGKYYQSEG.VFQVSP
Penicillium_brasilianum_CEO59775.1:15-472           LAEEYVKVTYKLWESSWRQDAVVLDRER....GIYTDPARVRQINHVGKYYNVPG.PHICQP
Aspergillus_cristatus_ODM22551.1:12-469             VAEEYIKVAYKLWESSWRSDAVILDRQR....GIYAEPSRIREINHVGKYFEVPG.PHLCQP
Pseudomonas_taiwanensis_WP_027907363.1:1-446        LAEEYLEVLYKLWEKSWDEDAVVLERET....GRYVEPSKVHAIGHLGEHFQVPG.MHLSQP
Acidovorax_avenae                                   RADEFMDVAYQLWEGSWDDDAVLRDRAR....RIYADPARVRPVRHRGTHYQVEG.YHLCEP
Staphylococcus_pseudintermedius_WP_101431388.1:4-   RADEFLQVSYQLWEDSWEDDAVVKTRET....DTYADPSKVHAIHHKGTFFDIPG.PHLVEP
Rhodococcus_triatomae_WP_081607437.1:2-451          IADEFMEVVYKLWEGSWEDDAVVRDAER....GVYTDPTKVHPAAHKGTYFSVPD.PFLCEP
Alcaligenes_sp._RS4_230_WP_128393375.1:4-450        RADDFLQLCYKLWEGSWEDGAIIRDRNG....RIYTDPNKVHAIAHNGPYYRAHG.YHSSEP
Gibbsiella_quercinecans_WP_121552947.1:7-454        QADEFLDVAYQLWEGSWQDDAVLADKQR....RIYAAADKIHQIRHRGEFYQVEG.YHLSSP
Acinetobacter_ursingii_WP_044435211.1:5-452         QAEEFLQLCYQYWEGSWEDDAIQKDKIR....RIFTDPSRVHEIQHQGRFYQSQG.VFQVAP
Erwinia_typographi_WP_034887712.1:4-447             QADEFLDVVYQLWEGSWQDDALVVDRQQ....RLYADAQKIHQVNHQGEFYQVQG.YHLSSP
Trichoderma_harzianum_KKP01913.1:14-470             IADEYVDAVYKLWQSSWRDDAVKLDSEK....GIYTDPSLVRPINHQGKYYTVPG.PHVCQP
Fusarium_graminearum_235_CEF79766.1:12-466          IAEEYLEVMYKLFQSSWRDDAVRLDREA....GIYTDPSRVREIGHKGKYFNVAG.PHIVNP
Gordonia_alkanivorans_WP_006357654.1:10-461         HADEYLTVLYKLWEGSWERDAVRRDREA....GIFADPAKVHHIGHEGKHFKVPG.IHLSEP
Acinetobacter_radioresistens_WP_111281495.1:5-455   QAEEFLELCYKFWEGSWENDAVVKDRQN....RIFTEPAKVHSIRHQGQFYQSEG.VFQVSP
Mesorhizobium_plurifarium_WP_073985566.1:5-458      IADEFLEVVYKLWEGSWEDDAVIMDRTA....GVFADPAKVHPIRHKGRYFSVPD.AFVAEP
Oceanobacillus_halophilus_WP_121203049.1:1-454      RADEFLEVVYKLWEHSWEEDAVVYNREK....DIFANPEKVHAIDHEGEFFRVPG.VHLTEP
Acinetobacter_tandoii_240_WP_100241535.1:7-457      QAEEFLELCYKFWEGSWENDAILKDKAQ....RIFTNPAKVHAIQHQGQFYQSQG.VFQVSP
Burkholderia_plantarii_WP_042624083.1:1-457         RADEFMEVCYRLWEGSWEDGARLRDRER....GIYADPARIHAIRHEGAHFRSEG.VFQCEP
Alcaligenes_faecalis_WP_094195208.1:6-457           RAEDFLQLAYKLWEGSWEDGALIADKQR....RIHALPEKVHTVHHEGPFYRSNA.IHMSAP
Bradyrhizobium_arachidis_WP_092214516.1:1-453       LADEFLDAVYALWEGSWDDEAVVVDPIR....GVIADPSRIRKVHHHGAHFKIDA.IHLVHP
Cupriavidus_plantarum_WP_109583068.1:1-458          RADEFLDVCYALWEGGWEDGAVVRDREA....GVYGDPARIHRVRHDGEHLRTDG.VFQCEP
Variovorax_sp._770b2_245_WP_093443018.1:5-461       RADEFMDLVYQLWETSWDDDAVVADRAS....GIYTEPARVRRIRHEGPAYRLDA.MHLCEP
RZJ22128.1:4-447                                    RADEFIEVTQKLWD.SWEDDAFIYNKES....GQFFHADKIHEPQHTGKYFSVQGALNVSRP
LadB_Geobacillus_thermoleovorans_BAM76371.1         RTREYIHLLK......................QLFTLPS....VTYEGRFYRLEN.ASLF.P
LadAbeta_Geobacillus_thermoleovorans_BAM76372.1     RAREFVDVVNGLWE.SWDEEALIRDQSS....GIYFHAEKWRPLNHHGTHFSVRG.PLNSSR
Af1_AFLA_024140_XP_002373925.1                      IAEEYIKVTYKLWESSWRSDAVVLDRER....GIYTDPSRVREINHVGKYFSVPG.PHICQP
Af3_AFLA_58870_XP_002378631.1                       IADEYLDVTYKLWEGSWRDDAVNVKDGV....AGYADPKAVRQINHEGKYFNVPG.PHLCEP
Af4_AFLA_049780_XP_002383105.1                      QADEYLRVVYKLWEGSWASDALSPDPET....DTYVDPAKVRQINHKGKYFSLNT.RHIVDP
Af5_AFLA_126060_XP_002381799.1                      RADEYMELTYKLWEGTWRDGAVVKDPKT....GVYSDPNQVRAIEHNGKYFKSTA.ASQLPA
Af2_QMW32876.1:9-463                                IAHEYMEVLYKLWEGSFRDDAVVDDREQ....GVYIASDAVRQIHHKGKYFEVPG.PHFCEP
Yarrowia_lipolytica_CLIB122_YALI0C07546p_XP_50156   KADEYMDVVYKLWESSWAEDAVIKGLKT....DAF.DTSKINYIDHEGKHFKVKG.PAVALP
Rhizobium_sp._CF122_WP007794079.1                   RAEEFADVVRGLWD.TWEEDAFLRDKES....GIYFDPDKQHVLNHKGKFFKVRG.PLNVAR
Acinetobacter_sp._NIPH_2100_LLM                     RAQEYVDLIKKLWD.SWEDDAFIHDKAS....GVFYNPEKVHNPNHQGKYYAVKG.ALNVPR
Nostoc_sp._PCC_7107_WP_015115963.1                  RAEEFVAVVKGLWD.SWDDDAFIRDRES....GVYFDPNKLHILNHKGKHFSVRG.PLNVGR
Aspergillus_flavus_NRRL3357_uncharacterized         RAREFYDVVTGLWD.SFADDAFIRDQET....GIYFDPEKLHVLDHKGDDLKVRG.PLNIAR
Paenibacillus_curdlanolyticus_WP006037998.1         RAEEFVDVVTKLWD.SWDDDALLYDKDS....ARFADKDKVRAIDHKGKFFSVRG.PLNVAR
LadAbeta_Geobacillus_thermoleovorans_FMN-dependen   RAREFVDVVNGLWE.SWDEEALIRDQSS....GIYFHAEKWRPLNHHGTHFSVRG.PLNSSR
SsuD_1M41                                           ASAEFTQVWRRLLQ...........RET...............VDFNGKHIHVRG.AKLLFP
Paenibacillus_curdlanolyticus_WP006037998.1         RAEEFVDVVTKLWD.SWDDDALLYDKDS....ARFADKDKVRAIDHKGKFFSVRG.PLNVAR
Bacillus                                            AADEFLDV....WKGLLAGDT......................VNYEGKHIHVEN.SELLYP
Pseudomonas                                         VTDEFLRVWRRVLQG...........EA...............VDFHGKHIHVENAKALYPP
LadB_Geobacillus_thermoleovorans_FMN-dependent      RTREYIHLLK......................QLFTLPS....VTYEGRFYRLEN.ASLF.P
Pseudomonas_aeruginosa_PAO1_NP_251290.1             RTDEYLQALRAIWTAT.........................GPVDFDGEFYRFQGASPAVRP
Alkanesulfonate                                     RLEEYIKIVRRAWESA..........DP...............FDWDGKYYQFKQYSNKVRP
Alkanesulfonate                                     RLEEYIRILRRAWESA..........DP...............FDWDGEYYQFKQFSNKVRP
Aspergillus_niger_ATCC13496_RDH19503.1              RLEDYIRILRRAWASE..........DP...............FDWESEYYTFKQFSNRVRP
Penicillium_digitatum_Pd1_XP014534980.1             RLEDYIKILRRAWKSD..........EP...............FDWDSKYYTFKQFSNQVRL
Acinetobacter_rudis_WP_016657872.1                  AADEFLQI....WRGVLQGETVNFE......................GKHLNVKNAKALYPP
```

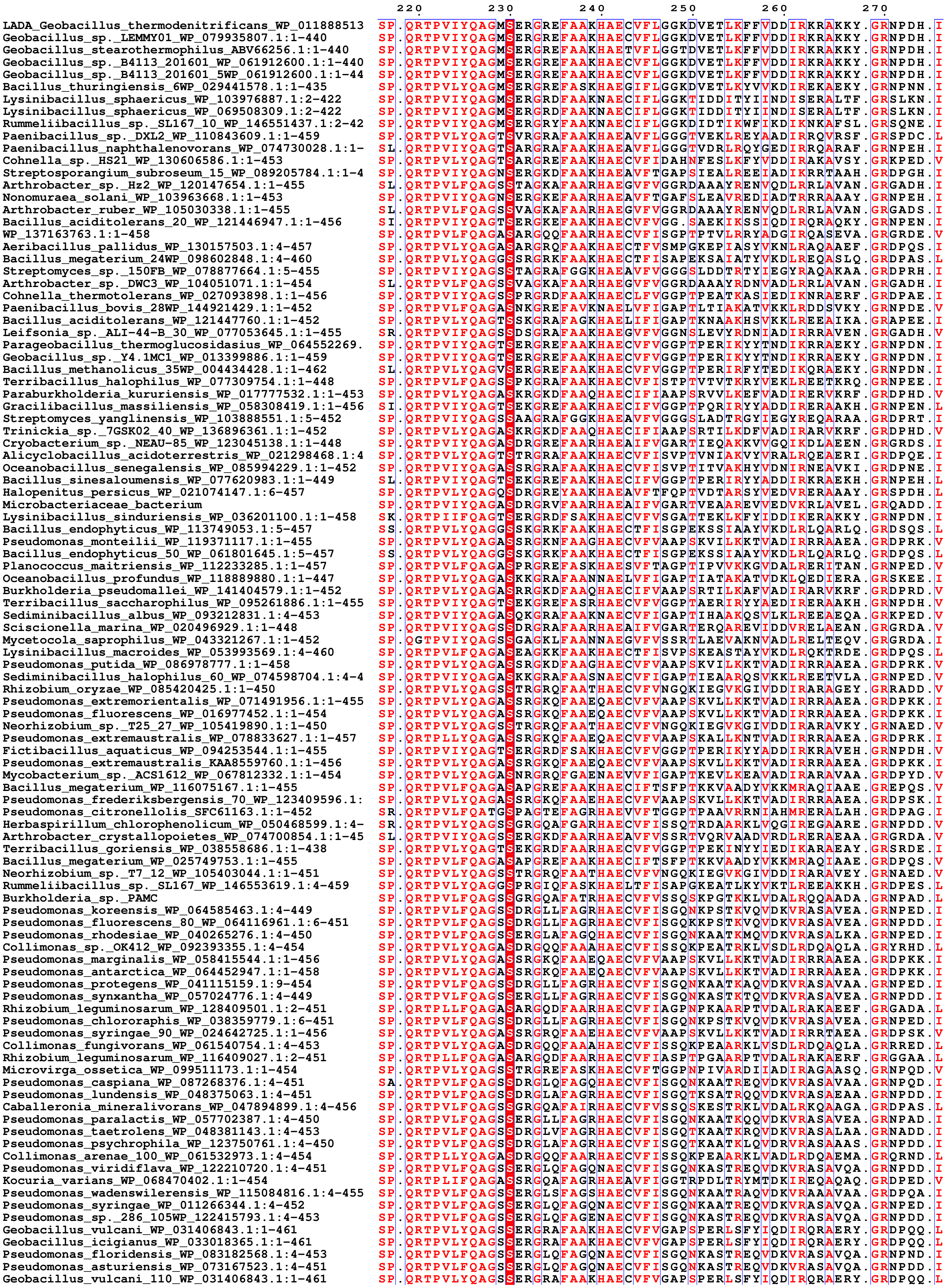
220
230
240
250
260
270
LADA_Geobacillus_thermodenitrificans_WP_011888513
Geobacillus_sp._LEMMY01_WP_079935807.1:1-440
Geobacillus_stearothermophilus_ABV66256.1:1-440
Geobacillus_sp._B4113_201601_WP_061912600.1:1-440
Geobacillus_sp._B4113_201601_5WP_061912600.1:1-44
Bacillus_thuringiensis_6WP_029441578.1:1-435
Lysinibacillus_sphaericus_WP_103976887.1:2-422
Lysinibacillus_sphaericus_WP_069508309.1:2-422
Rummeliibacillus_sp._SL167_10_WP_146551437.1:2-42
Paenibacillus_sp._DXL2_WP_110843609.1:1-459
Paenibacillus_naphthalenovorans_WP_074730028.1:1-
Cohnella_sp._HS21_WP_130606586.1:1-453
Streptosporangium_subroseum_15_WP_089205784.1:1-4
Arthrobacter_sp._Hz2_WP_120147654.1:1-455
Nonomuraea_solani_WP_103963668.1:1-453
Arthrobacter_ruber_WP_105030338.1:1-455
Bacillus_aciditolerans_20_WP_121446947.1:1-456
WP_137163763.1:1-458
Aeribacillus_pallidus_WP_130157503.1:4-457
Bacillus_megaterium_24WP_098602848.1:4-460
Streptomyces_sp._150FB_WP_078877664.1:5-455
Arthrobacter_sp._DWC3_WP_104051071.1:1-454
Cohnella_thermotolerans_WP_027093898.1:1-456
Paenibacillus_bovis_28WP_144921429.1:1-452
Bacillus_aciditolerans_WP_121447760.1:1-452
Leifsonia_sp._ALI-44-B_30_WP_077053645.1:1-455
Parageobacillus_thermoglucosidasius_WP_064552269.
Geobacillus_sp._Y4.1MC1_WP_013399886.1:1-459
Bacillus_methanolicus_35WP_004434428.1:1-462
Terribacillus_halophilus_WP_077309754.1:1-448
Paraburkholderia_kururiensis_WP_017777532.1:1-453
Gracilibacillus_massiliensis_WP_058308419.1:1-456
Streptomyces_yanglinensis_WP_103888551.1:5-452
Trinickia_sp._7GSK02_40_WP_136896361.1:1-452
Cryobacterium_sp._NEAU-85_WP_123045138.1:1-448
Alicyclobacillus_acidoterrestris_WP_021298468.1:4
Oceanobacillus_senegalensis_WP_085994229.1:1-452
Bacillus_sinesaloumensis_WP_077620983.1:1-449
Halopenitus_persicus_WP_021074147.1:6-457
Microbacteriaceae_bacterium
Lysinibacillus_sinduriensis_WP_036201100.1:1-458
Bacillus_endophyticus_WP_113749053.1:5-457
Pseudomonas_monteilii_WP_119371117.1:1-455
Bacillus_endophyticus_50_WP_061801645.1:5-457
Planococcus_maitriensis_WP_112233285.1:1-457
Oceanobacillus_profundus_WP_118889880.1:1-447
Burkholderia_pseudomallei_WP_141404579.1:1-452
Terribacillus_saccharophilus_WP_095261886.1:1-455
Sediminibacillus_albus_WP_093212831.1:4-453
Sciscionella_marina_WP_020496929.1:1-448
Mycetocola_saprophilus_WP_043321267.1:1-452
Lysinibacillus_macroides_WP_053993569.1:4-460
Pseudomonas_putida_WP_086978777.1:1-458
Sediminibacillus_halophilus_60_WP_074598704.1:4-4
Rhizobium_oryzae_WP_085420425.1:1-450
Pseudomonas_extremorientalis_WP_071491956.1:1-455
Pseudomonas_fluorescens_WP_016977452.1:1-454
Neorhizobium_sp._T25_27_WP_105419890.1:1-450
Pseudomonas_extremaustralis_WP_078833627.1:1-457
Fictibacillus_aquaticus_WP_094253544.1:1-455
Pseudomonas_extremaustralis_KAA8559760.1:1-456
Mycobacterium_sp._ACS1612_WP_067812332.1:1-454
Bacillus_megaterium_WP_116075167.1:1-455
Pseudomonas_frederiksbergensis_70_WP_123409596.1:
Pseudomonas_citronellolis_SFC61163.1:1-452
Herbaspirillum_chlorophenolicum_WP_050468599.1:4-
Arthrobacter_crystallopoietes_WP_074700854.1:1-45
Terribacillus_goriensis_WP_038558686.1:1-438
Bacillus_megaterium_WP_025749753.1:1-455
Neorhizobium_sp._T7_12_WP_105403044.1:1-451
Rummeliibacillus_sp._SL167_WP_146553619.1:4-459
Burkholderia_sp._PAMC
Pseudomonas_koreensis_WP_064585463.1:4-449
Pseudomonas_fluorescens_80_WP_064116961.1:6-451
Pseudomonas_rhodesiae_WP_040265276.1:4-450
Collimonas_sp._OK412_WP_092393355.1:4-454
Pseudomonas_marginalis_WP_058415544.1:1-456
Pseudomonas_antarctica_WP_064452947.1:1-458
Pseudomonas_protegens_WP_041115159.1:9-454
Pseudomonas_synxantha_WP_057024776.1:4-449
Rhizobium_leguminosarum_WP_128409501.1:2-451
Pseudomonas_chlororaphis_WP_038359779.1:6-451
Pseudomonas_syringae_90_WP_024642725.1:1-456
Collimonas_fungivorans_WP_061540754.1:4-453
Rhizobium_leguminosarum_WP_116409027.1:2-451
Microvirga_ossetica_WP_099511173.1:1-454
Pseudomonas_caspiana_WP_087268376.1:4-451
Pseudomonas_lundensis_WP_048375063.1:4-451
Caballeronia_mineralivorans_WP_047894899.1:4-456
Pseudomonas_paralactis_WP_057702387.1:4-450
Pseudomonas_taetrolens_WP_048381143.1:4-453
Pseudomonas_psychrophila_WP_123750761.1:4-450
Collimonas_arenae_100_WP_061532973.1:4-454
Pseudomonas_viridiflava_WP_122210720.1:4-451
Kocuria_varians_WP_068470402.1:1-454
Pseudomonas_wadenswilerensis_WP_115084816.1:4-455
Pseudomonas_syringae_WP_011266344.1:4-452
Pseudomonas_sp._286_105WP_122415793.1:4-453
Geobacillus_vulcani_WP_031406843.1:1-461
Geobacillus_icigianus_WP_033018365.1:1-461
Pseudomonas_floridensis_WP_083182568.1:4-453
Pseudomonas_asturiensis_WP_073167523.1:4-451
Geobacillus_vulcani_110_WP_031406843.1:1-461

```
Pseudomonas_syringae_WP_024682626.1:4-452                 SP.QRTPVLFQAGSSERGLQFAGQNAECVFISGQNKAATREQVDKVRASAVQA.GRNPED.I
LadAalpha_Geobacillus_thermoleovorans_BAM76377.1          SP.QRTPVLYQAGSSERGRAFAAKHAECVFVGAPSPERLSFYIQDIRQRAERY.GRDPQQ.L
Tsukamurella_pseudospumae_WP_068746786.1:1-457            SP.QRSPVIYQAGASPRGLKFATENAEAIFVAAPTKAILKDVVRTVREGLVAN.GRDPYS.A
Kocuria_sp._Marseille-P3598_WP_085529073.1:5-456          SP.QRTPVIFQAGASSRGQAFAGRHAEAVFVGGLRPDLTRYMTDKIRDEAEAA.GRNRDD.V
Microvirga_aerophila_WP_114188789.1:1-456                 SP.QRTPVLYQAGASARGKDFAASHAECVFLYGPNKEQTRKAVEDVRQRAAKL.GRDPNG.V
Burkholderia_ubonensis_WP_071760483.1:5-455               SP.QRTPVLYQAGASARGVEFAGRHAECVFVNGQSKAAARAAVLDIRAAAARQ.GRDPAS.I
Achromobacter_marplatensis_WP_006226823.1:4-450           SP.QRTPVLYQAGSSGRGQAFAARHAECVFVSSQTKEGLRKLVAEVRESVARE.GRSPRD.I
Acinetobacter_baumannii_WP_070165746.1:1-443              SP.QRTPVLYQAGASSRGQKFASQNAECVFIAAPSKIATKKVVQGIRQKLVQE.GRDPYS.V
Labedella_phragmitis_WP_128496094.1:1-453                 SI.QRSPVIYQAGASPRGIRFAASNAEAIFVASSTKAGLAATVGRIRDALEAA.GRDRYA.A
Nocardia_cyriacigeorgica_120_WP_130918184.1:1-453         SP.QRTPVIYQAGASPRGVRFAAENAEAIFIAGPSKRVLAQTVSRVRDALEAA.GRDRYA.A
Nocardia_cyriacigeorgica_WP_130918184.1:1-453             SP.QRTPVIYQAGASPRGVRFAAENAEAIFIAGPSKRVLAQTVSRVRDALEAA.GRDRYA.A
Mycobacterium_sp._1164985.4_WP_067298521.1:1-453          SP.QRTPVIYQAGSSPRGVRFAAENAEAIFTASPTKEILRQTVSTIRQELEIA.GRDPYS.V
Rhodococcus_sp._1R11_WP_135042739.1:1-449                 SP.QRTPVIFQAGASPRGVRFAAQNAEAIFVGAPTKEILAQTVSNIRRALQDA.GRDPHS.A
Erwinia_amylovora_WP_004157587.1:5-459                    SP.QRTPVLYQAGASSRGKRFAAEHAECVFVAAPSKVLLKKTVADIRRRAAET.GRDPRS.I
Ralstonia_mannitolilytica_WP_102079134.1:4-451            SP.QRTPVLFQAGSSGRGQRFAARHAECVFISPPNKEAARQTVAALREQLLAA.GRRPDD.I
Leifsonia_flava_WP_135120808.1:15-465                     SP.QRSPVIYQAGASPRGIGFAAANAEAIFVAAPTKEVLKSTVERIRDALEAE.GRDRYS.A
Cryobacterium_roopkundense_WP_084141147.1:1-453           ST.QRSPVIYQAGASPRGITFAAENAEAIFVAASTKPQLTATVKKIRDALEAE.GRDRYA.A
Cupriavidus_pauculus_WP_101681197.1:1-455                 SP.QRTPVLYQAGSSTRGRCFAATHAECVFVNGQKKEGVKEIVDDIRAQAVQL.GRQADD.V
Rhizobium_sp._BK333_130_WP_133704837.1:2-454              SP.QRTPLLFQAGASARGQDFAARHAECVFIASPTPQGAKPMTDALRQRAVDF.GRRADD.L
Caballeronia_mineralivorans_WP_047846789.1:1-454          SP.QRTPVLYQAGASGAGKAFAAQHAECVFVAAPTKEVLKSYVADVRHQAAEA.GRNAAG.L
Nocardia_altamirensis_WP_069163049.1:1-454                SP.QRTPVIYQAGASTRGVRFAAENAEAIFIAAPSKRLLAQTVSRIRDALVAA.GRDRYS.A
Cupriavidus_sp._P-10_WP_116322758.1:1-452                 SP.QRTPVLYQAGSSPKGQAFAGRHAECVFVSSPTKHVLTNTVKTMRAAAVQQ.GRCPED.L
Serratia_sp._S1B_WP_116726564.1:1-452                     SP.QRTPVLYQAGASSRGQQFASQNAECVFIAPPSKAAAKKVVQGIRAKLVEQ.GRDPYS.V
Pseudomonas_putida_WP_043864435.1:1-452                   SP.QRTPVLYQAGASTRGRAFAAANAECVFVAAPSKTVLKNQVAQVRAAAVAA.GRAPGD.I
Pseudomonas_aeruginosa_WP_023084248.1:4-453               SP.QRTPVLFQAGSSERGLAFAARHAECVFVSGQTREATRQLVQRIRAAVVGA.GRRAED.I
Pseudomonas_mucidolens_WP_084381795.1:9-455               SP.QRTPVLFQAGSSERGLLFAGRHAECVFISGQNKAATKLQVDKVRASAVQD.GRNADD.I
Pseudomonas_poae_WP_060549803.1:4-450                     SP.QRTPVLFQAGSSDRGLLFAGRHAECVFISGQTRAATKAQVDKVRASAVQA.GRNAQD.I
Pseudomonas_chlororaphis_WP_123573412.1:9-454             SP.QRTPVLFQAGSSDRGLLFAGRHAECVFISGQNKASTRAQVDKVRASAVAA.GRNPED.I
Janthinobacterium_lividum_140_WP_072454679.1:1-44         SP.QRTPLLYQAGTSPRGTRFAARHAEATFVSGPSKTVVKRYADDLRAAVRQS.GRDGDA.L
Haladaptatus_cibarius_WP_049970513.1:4-446                SP.QRTPVLYQAGSSERGRNFAANNAEALFVSQPTTEAVKSYVEDVRERATEV.GRNGDE.L
Arthrobacter_luteolus_WP_066298473.1:1-443                SP.QRTPAIFQAGASARGRRFGARHAEGIFLNSIRPDLTRTTDATRDEFEAA.GRPRDA.A
Geobacillus_sp._ZGt-1_WP_047758532.1:1-436                SP.QRTPVLYQAGSSERGRAFAAKHAECVFVGAPSPERLSFYIQDIRQRAERY.GRDPQQ.L
Lysinibacillus_composti_WP_124766485.1:4-458              SP.QRTPVLFQAGASTKGRAFAARNAELIFIGAPNIAAAKETVRKIREDIQKT.GRSQDE.V
Microcella_putealis_WP_130486201.1:20-474                 SP.QRTPVIYQAGASPRGVAFAAGNAEAIFVAAPTKEVLRGTVTRIRDALEAA.GRDRYA.A
Pseudomonas_nitroreducens_WP_037016976.1:1-453            SP.QRTPVLYQAGASSRGKAFAAGNAECVFVAAPTKNILRDQVADLRLAVEA.GRQPDD.V
Acinetobacter_wuhouensis_WP_130168784.1:1-453             SP.QRTPVLYQAGASSRGQQFASQNAECIFIAPPSKIAAKKVVQSIRQKLREE.GRDPYS.V
Bradyrhizobium_sp._YR681_WP_008144663.1:1-454             SP.QRTPVLYQAGTSPRGRQFAAKHAECVFMSGPSAKIIAPRVAAIREEAAKF.GRNPAE.I
Schumannella_sp._10F1D-1_WP_141164655.1:10-460            SP.QRTPVIYQAGASSRGIGFAAGNAEAIFVAAPSKEVLRSTVSKIRDALEAA.GRDRYS.A
Pseudomonas_coronafaciens_150_WP_122355187.1:4-45         SP.QRTPVLFQAGSSERGLQFAGQNAECVFISGQNKTATREQVNKVRASAVQA.GRNPED.I
Pseudomonas_syringae_WP_003430238.1:4-452                 SP.QRTPVLFQAGSSERGLQFAGQNAECVFISGQNKAATREQVDKVRASAVQA.GRNPED.I
unclassified_Rhodococcus_WP_094682261.1:1-449             SP.QRTPVIFQAGASPRGVRFAAQNAEAIFVGAPTKEILAQTVSNVRRALRDA.GRDPYS.A
Hafnia_alvei_WP_046449330.1:6-449                         SP.QRTPLLYQAGSSKRGIQFAARHAECTFVNGATPQAMKAQVNTLRQAAVAA.GRRADS.L
AS9A_3890_Hoyosella_subflava_WP_013808677.1:1-453         SP.QRTPVIYQAGASPRGVRFASEHAEAIFVAAPSKRVLKETVRRIRSGLVAA.GRAPYS.A
Acinetobacter_baumannii_WP_000995600.1:1-443              SP.QRTPVLYQAGASSRGQKFASQNAECVFIAAPSKIATKKVVQGIRQKLVQE.GRDPYS.V
Agrobacterium_sp._B133/95_WP_065696417.1:2-454            SP.QRTPLLFQAGASARGQDFAARHAECVFIASPVPQGAKPMTDALRQRAVDF.GRRADD.L
Microbacterium_sp._KROCY2_WP_024289149.1:1-453            SP.QRTPLIFQAGASSRGQAFAGKHAEAVFIGALRPDLTRYMTDRIRDKVEAE.GRDRND.V
Variovorax_paradoxus_WP_012747040.1:6-452                 SP.QRTPVLFQAGSSGRGQRFAARHAECVFISPPSKEAARQTVQALREQLVQA.GRRPDD.V
Methylobacterium_radiotolerans_WP_076730229.1:5-4         SP.QRTPVLYQAGASGRGRAFAGRHAECVFISARDPATARESVRAIRAEAVAA.GRNPDD.V
Streptomyces_sp._BK215_WP_132915265.1:7-452               SP.LRTPVLFQAGSSEAGRSFAARHAEGTFILAPNPLAARTNIDDVRARATAA.GRRPED.I
Geobacillus_thermodenitrificans_160_WP_008880416.         SP.QRTPLLYQTGSSDRGRAFAAKHAECVFVGAPSPERIRSYIQDIRQQAERY.GRDPQQ.L
Pseudomonas_aeruginosa_WP_132556951.1:356-785             SP.QRTPVLFQAGSSERGLAFAARHAECVFVSGQTREATRQLVQRIRAAVVGA.GRRAED.I
Penicillium_solitum_OQE02104.1:14-471                     SP.QRTPVILQAGTSKAGKTFAAQHAEAIFVAGHSPAVVAKNVKEIRELAQSEYGRDPQS.I
Penicillium_vulpinum_OQE05502.1:12-469                    SP.QRTPVILQAGTSKAGKTFAAQHAEAIFVAGHSPAVVAKNVREIRELAQSQYGRDPQS.I
Clavibacter_michiganensis_WP_045526194.1:1-454            SP.QRTPVVFQAGASPRGREFAAKHGEAIFINGLTPELTRPVTDDIRDRAERI.GRPRDS.V
Glutamicibacter_creatinolyticus_WP_054821484.1:6-         ST.QRTPVIYQAGASSRGVKFAAENAEAVFVTSPTREMLKATVTKIRDAAEAA.GRDRYD.I
Campylobacter_sp._P0078_WP_086237522.1:4-454              SI.QRTPVLFQAGNSPRGLEFAAKHAEAIFIAPIAKAYTKDAVKQVRQALIKA.GRNPYS.A
Pseudomonas_furukawaii_WP_003454569.1:1-447               SP.QRTPLLFQAGSSARGLKFAGNHAECVFIGGQNKEAVRAQVDKVRAAAVAA.GRPADA.I
Leucobacter_triazinivorans_WP_130111548.1:1-442           SP.QRTPLVFQAGASPRGIRFAARHAEAIFIASPTNAVARKSVDAIRSGLVEA.GRSPDA.A
Aspergillus_brasiliensis_CBS_101740_OJJ68286.1:12         SP.QRTPVILQAGTSKAGKAFAAQHAEAIFVAGHSPSVVAKNIAEIRETAKTQFGRDPTS.I
Aspergillus_udagawae_170_GAO89440.1:15-472                SP.QRTPVILQAGTSKAGKTFAAQHAEAIFVAGHSPAVVAKNIAEIRAMAQADFGRDPKS.I
Aspergillus_flavus_RMZ37351.1:15-472                      SP.QRTPVILQAGTSKSGKAFAAQHAEAIFVAGHSPVVVAKNVAEIRELAKTQFGRDPQS.I
Aspergillus_flavus_RAQ54081.1:15-472                      SP.QRTPVILQAGTSKSGKAFAAQHAEAIFVAGHSPVVVAKNVAEIRELAKTQFGRDPQS.I
Penicillium_arizonense_XP_022490779.1:12-469              SP.QRTPVILQAGTSKAGKTFAAQHAEAIFVAGHSPSVVAKNVKEIRELAATEFGRDPRS.I
Salinibacterium_sp._CGMCC                                 SP.QRTPVIFQAGASPRGIDFAANNAEAIFVASPTKEILKATVGKIRDALEAA.GRDRYS.A
Aspergillus_fumigatus_OXN06785.1:14-471                   SP.QRTPVILQAGTSKAGKTFAAQHAEAIFVAGHSPVVVAKNIAEIRATAQAQFGRDPKS.I
Aspergillus_lentulus_GAQ10210.1:7-464                     SP.QRTPVILQAGTSKAGKTFAAQHAEAIFVAGHSPAVVARNIAEIRAMAQAQFGRDPKS.I
Aspergillus_flavus_RAQ66103.1:15-472                      SP.QRTPVILQAGTSKSGKAFAAQHAEAIFVAGHSPVVVAKNVAEIRELAKTQFGRDPQS.I
Aspergillus_fumigatus_OXN22894.1:14-471                   SP.QRTPVILQAGTSKAGKTFAAQHAEAIFVAGHSPVVVAKNIAEIRATAQAQFGRDPKS.I
Aspergillus_nidulans                                      SP.QRTPVILQAGTSKAGKTFAAQHAEAIFVAGHSPSVVAKNVAEIRELAKSQYGRDPSS.I
Penicillium_nalgiovense_OQE79848.1:14-471                 SP.QRTPVILQAGTSKAGKTFAAQHAEAIFVAGHSPAVVAKNVKEIRELAQSEYRRDPQS.I
Aspergillus_wentii                                        SL.QRTPFLFQAGVSEAGNGFGGKHGEAIFVGGQTPEIVRATVDNIRRVAEGE.GRDPNH.I
Gordonia_iterans_WP_105943624.1:1-453                     SP.QRTPVIYQAGASPRGRRFAAQNAEAVFVAAPTKTILRELVGKIREELVAA.GRGPYD.A
Azotobacter_beijerinckii_WP_090735436.1:4-454             SP.QRTPLLFQAGTSERGRRFAGRHAEGVFITGQNPGVAEQVRQVRAEAVAA.GRPADA.V
Staphylococcus_sp._NAM3COL9_WP_057513692.1:4-453          SP.QRTPVLFQAGASEKGKTFAAKHAEAVFTKHTSVESLKRYVDDIHTRAEQH.GRSKED.I
Acidovorax_sp._56_WP_099656603.1:11-450                   SL.QRTPVIYQAGTSDRGKAFAARHAEAVFISGNNKALVRESVVALRQAAADA.GRAPED.L
Paenibacillus_sp._BIHB4019_WP_099518466.1:1-456           SP.QRTPVIFQAGASGRGRAFAAKHAEGIFLKAPTTEVLRDQIADIRRAAEY.GRDPAA.V
Penicillium_antarcticum_OQD90033.1:5-465                  SP.QRTPFILQAGTSTSGKAFAAKHAEAVFLHAQKPELVRPSVDSIRQQAAEL.GRNPQD.I
Aspergillus_oryzae_RIB40_190_XP_001823703.1:9-463         SP.QRTPFLFQAGVSEAGNGFGGKHGEAIFVGGQTPEGVRVTVDNIRKVAAEE.GRDANH.I
Fusarium_oxysporum_f._sp._pisi                            SP.QRTPLILQAGTSKAGKNFAAQHAEAIFVAGHSPVTVAKNIAEIRTLAKEQFGRDPKA.I
Colletotrichum_fructicola_Nara_gc5_ELA35345.1:18-         SP.QRTPFLFQAGVSEAGNGFGGKHGEAIFIGGQTIEGVRKTVDNLRKIAAEE.GRDPNH.I
Micrococcus_luteus_WP_041104460.1:19-468                  SV.QRTPVIYQAGASTRGRAFAGKHAEAVFINSPTKELAAATVKKIRQSLVDA.GRDPYD.V
Aspergillus_awamori_GCB21923.1:12-469                     SP.QRTPVILQAGTSKAGKAFAAQHAEAIFVAGHSPSVVAKNIAEIRETAKTQFGRDPAG.I
Aspergillus_niger_CBS                                     SP.QRTPVILQAGTSKAGKAFAAQHAEAIFVAGHSPSVVAKNIAEIRETAKTQFGRDPAG.I
Aspergillus_lacticoffeatus_CBS_101883_XP_02545389         SP.QRTPVILQAGTSKAGKAFAAQHAEAIFVAGHSPSVVAKNIAEIRETAKTQFGRDPAG.I
Aspergillus_wentii_DTO_134E9_OJJ40237.1:14-471            SR.QRTPVILQAGTSKAGKTFAAQHAEAIFVGGHSPSVVAKNVAEIRQLAQTEFGRDGTS.I
Aspergillus_indologenus_CBS_114.80_PYI31207.1:11-         SP.QRTPVILQAGTSKAGKLFAAQHAEAIFVAGHSPSVVKNNIAEIRELAATRFGRDGTS.I
Aspergillus_fischeri_NRRL_181_XP_001264002.1:14-4         SP.QRTPVILQAGTSKAGKTFAAQHAEAIFVAGHSPAVVAKNIAEIRAMAQAQFGRDPKS.I
Penicillium_coprophilum_200_OQE46388.1:11-466             SP.QRTPFLFQAGVSEAGGRFGGKHGEAIFIGGQTPEGTRLTVDNIRNIAKQQ.GRDPNH.I
Cupriavidus_taiwanensis_WP_116379876.1:8-454              SP.QRTPVLFQAGSSGRGQRFAARHAECVFISPPSKEAARKTVAALREQLVAA.GRRPDD.V
Penicillium_digitatum                                     SP.QRTPFILQAGTSTAGKAFAAKHAEAVFVHAQKPELVRPSVDSIRQQAAEI.GRNPQH.I
Aspergillus_niger_GAQ46262.1:12-469                       SP.QRTPVILQAGTSKAGKAFAAQHAEAIFVAGHSPSVVAKNIAEIRETAKTQFGRDPAG.I
Aspergillus_nomius_NRRL_13137_XP_015412385.1:15-4         SP.QRTPVILQAGTSKSGKAFAAQHAEAIFVAGHSPVVVAKNVAEIRELAKTQFGRDPQS.I
Aspergillus_aculeatus_ATCC_16872_XP_020055010.1:1         SP.QRTPVILQAGTSKSGKLFAAQHAEAIFVAGHSPSVVKNNIAEIRELAASQFGRDGTS.I
Penicillium_brasilianum_OQQ84969.1:19-474                 SP.QRTPFLFQAGVSEAGNGFGGKHGEAIFIGGQTPEATRATVENIRKIAQQE.GRDPNH.L
Penicillium_subrubescens_OKP15100.1:19-474                SP.QRTPFLFQAGVSEAGNGFGGKHGEAIFIGGQTPETTRATVENIRKIAQQE.GRNPNH.L
Trichoderma_arundinaceum_RFU80530.1:19-474                SP.QRTPFLFQAGVSEAGNGFGGKHGEAIFVGGQTPEGVRKTVDNIRDVARKE.GRDPNH.I
Aspergillus_thermomutatus_210_XP_026614594.1:15-4         SP.QRTPVILQAGTSKAGKTFAAQHAEAIFVAGHSPAVVAKNIAEIRTTAQGQFGRDPQS.I
Serratia_liquefaciens_WP_044551273.1:4-449                SP.QRTPLLFQAGSSSRGIQFAARHAECTFVNHATPAVMKAQVDKLRNAAVAA.GRQRQD.L
Micrococcus_luteus_WP_065572847.1:19-468                  SV.QRTPVIYQAGASTRGRAFAGKHAEAVFINSPTKELAAATVKKIRQALVDA.GRDPYD.V
Halolamina_rubra_WP_049979911.1:1-449                     SP.QRTPVIYQAGSSEYGRDFAAANAEAVFCSQPTEEGVREYVEDVQRAADA.GRDEDS.I
Acinetobacter_pittii_WP_075382847.1:7-452                 SI.QRTPTLFQAGASPKGMQFATRHAECVFIGGDKPEKIREQVEKKIRAAIEQQ.GRSADD.I
Corynebacterium_variabile_WP_052302534.1:2-464            SP.QRTPVIFQAGASRGSTFAADHAEAVFVAAPTKEALASTVRTLTAKLADA.GRSRED.V
Aspergillus_luchuensis_GAT21254.1:12-471                  SP.QRTPVILQAGTSKAGKAFAAQHAEAIFVAGHSPSVVAKNIAEIRETAKTQFGRDPAG.I
```

```
Aspergillus_luchuensis                                  SP.QRTPVILQAGTSKAGKAFAAQHAEAIFVAGHSPSVVAKNIAEIRETAKTQFGRDPAG.I
Aspergillus_bombycis_XP_022386026.1:15-472              SP.QRTPVILQAGTSKSGKAFAAQHAEAIFVAGHSPVVVAKNVAEIRELAKTQFGRDPQS.I
Agrobacterium_vitis_WP_070163524.1:1-454                SP.QRTPVLYQAGASGPGKKFAAEHAECVFVAAPTKSVLKAYVAEIRQQAAAA.GRDPNA.L
Pseudomonas_psychrotolerans_220_WP_074584491.1:5-       SR.QRTPVLFQAGSSDRGLAFAGRHAECVFISGQDQAATRAQVERVRGAAAAA.GRRADD.I
Micrococcus_yunnanensis_WP_135040276.1:19-468           SV.QRTPVIYQAGASTRGRAFAGKHAEAVFINSPTKELAAATVKKIRQALVDA.GRDPYD.V
Acinetobacter_calcoaceticus_WP_004642549.1:7-452        SV.QRTPTLFQAGASPKGMQFATRHAECVFIGGDKPEKIREQVKKIRALAEQQ.GRAGDD.I
Penicillium_brasilianum_CEO59775.1:15-472               SP.QRTPVILQAGTSKAGKTFAAQHAEAIFVAGHSPSVVAKNVAEIRQLAKTEFGRDPQS.I
Aspergillus_cristatus_ODM22551.1:12-469                 SP.QRTPLILQAGTSKAGKTFAAQHAEAIFVGGHSPSVVKKNIAEIREMAKTQFGRDPAS.I
Pseudomonas_taiwanensis_WP_027907363.1:1-446            SP.QRTPVLFQAGASARGQQFAARHAECVFISGPTPTVLRRYAEGIRQASDAQ.GRGRDE.V
Acidovorax_avenae                                       SP.QRTPVLFQAGSSGRGLRFAARHAECVFMSTQDKDSTRELVRALRAEVVRA.GRHPDD.V
Staphylococcus_pseudintermedius_WP_101431388.1:4-       SP.QRTPVLFQAGASEKGKTFAAKHAEAVFTKHTSIESLRAYVTDIRTRAQQF.GRNGED.I
Rhodococcus_triatomae_WP_081607437.1:2-451              SR.QRTPALFQAGASSAGREFAAKHAEAVFLVDTSVNSLRRSVDATTELARTL.GRSRSD.L
Alcaligenes_sp._RS4_230_WP_128393375.1:4-450            SP.QRTPVLYQAGSSGRGRQFASRHAECVFIAASDPATAKAASTKLRQDFVNA.GRQADD.V
Gibbsiella_quercinecans_WP_121552947.1:7-454            SP.QRTPLLFQAGASARGIRFAARHAECTFVNGATPSAMKAQVDKLRAAVVEA.GRAAED.L
Acinetobacter_ursingii_WP_044435211.1:5-452             SI.QRTPVLFQAGASPRGLAFATAHAECIFIGGDQPKKIRQQVDKIRELAVQQ.GRQADD.I
Erwinia_typographi_WP_034887712.1:4-447                 SP.QRTPLLFQAGSSARGIQFAARHAECSFVNASSPAAMRQQTQRLRQAALDA.GRRADD.L
Trichoderma_harzianum_KKP01913.1:14-470                 SP.QRTPVIMQAGTSKAGKAFSAKNAEAIFVAGHSPSVIAKNIADIRAAARDEYNRDPST.I
Fusarium_graminearum_235_CEF79766.1:12-466              SP.QRTPLLLQAGASKAGKLFAAQHAEAIFTSAHAPAVAKKNIAEIRQTAKDTFNRNPNN.I
Gordonia_alkanivorans_WP_006357654.1:10-461             SP.QGSPVIYQAGASPRGRRFAADNAEAIFVAAPTKAILRDVVSKIREELVLA.GRDPYD.A
Acinetobacter_radioresistens_WP_111281495.1:5-455       SP.QRTPVLFQAGASPRGLTFATQHAEAIFIGGDKPEKIKQQVEKIRSLATAQ.GREAEA.I
Mesorhizobium_plurifarium_WP_073985566.1:5-458          SP.QRTPVLFQAGVSGVGREFAARHAEGVFIAVHRPDLARKIVDDVRARAERL.GRDPQS.L
Oceanobacillus_halophilus_WP_121203049.1:1-454          SP.QRTPVLFQAGASPRGRDFAAKHAEAVFTKGHSLEALKSYTKDLRNRTIQQ.GRKGED.I
Acinetobacter_tandoii_240_WP_100241535.1:7-457          SP.QRTPVLYQAGASPRGLTFATQHAEGIFIGGDQPEKIKQQVDKIRALAVSQ.GRDANS.V
Burkholderia_plantarii_WP_042624083.1:1-457             SP.QRTPLLFQAGGSARGTRFAARHAECIFLGAPTRNGLRSSARAIRQALVDA.GRAPDS.A
Alcaligenes_faecalis_WP_094195208.1:6-457               SA.QRTPLLFQAGTSARGLQFAGQHAEGVFIGAGTPEAARESSRKLRQAAVDA.GRRAQD.L
Bradyrhizobium_arachidis_WP_092214516.1:1-453           SL.QRTPFLFQAGASNRGKDFAARHAEAVFLGEHSKAPTAANVAETRARARAF.GRDGRD.I
Cupriavidus_plantarum_WP_109583068.1:1-458              SP.QRTPLLFQAGGSDRGTRFAARHAECIFVGAPTPAGLRRTVDRLRDALREA.GRDPAS.V
Variovorax_sp._770b2_245_WP_093443018.1:5-461           SP.QRTPLLFQAGASPRGTAFAARHAEVVFVPNQGLKATAQVIRKLEAALTAV.GRTRDS.V
RZJ22128.1:4-447                                        V..QGYPVIVQAGQSEDGRELAGKYAEVIFTAQQNLADAQEFYRDVKSRLLKY.GRHADD.L
LadB_Geobacillus_thermoleovorans_BAM76371.1             KP.AQRPPIYFGGASDIAKEVAAKEADVYMMWGETFERMKERIQEMKQKAAHY.GRT....L
LadAbeta_Geobacillus_thermoleovorans_BAM76372.1         PP.QGKPVLIQAGSSQDGIRFAAHVAEVVFTAQSTLTDAQRFYQTVKQEAARA.GRNPDH.I
Af1_AFLA_024140_XP_002373925.1                          SP.QRTPVILQAGTSKSGKAFAAQHAEAIFVAGHSPVVVAKNVAEIRELAKTQFGRDPQS.I
Af3_AFLA_58870_XP_002378631.1                           SP.QRTPFLLQAGTSTAGKAFAAKHAEAIFLHGQKPELVRPSVDNVRQQAQAQ.GRDPAS.I
Af4_AFLA_049780_XP_002383105.1                          SP.QRTPFLFQAGTSAAGSAFAATHAEAIFVSSHSPAVLRPKIENIRKLAAEQ.GRDPRS.I
Af5_AFLA_126060_XP_002381799.1                          SK.QRTPLLFQAGASSAGKRFAAKHSEVMFLPGLEPEKTKAVVDDMRKHLAEI.GRPTDS.I
Af2_QMW32876.1:9-463                                    SP.QRTPFLFQAGVSEAGNGFGGKHGEAIFVGGQTPEGVRVTVDNIRKVAAEE.GRDANH.I
Yarrowia_lipolytica_CLIB122_YALI0C07546p_XP_50156       SV.QRTPVLFQAGMSSAGRAFAGKHAEAVFIAGPSPQLIRQTVNALRESAGDR.....K.L
Rhizobium_sp._CF122_WP007794079.1                       AP.QGQPVLVQAGSSEPGKELAARTAEVVFTAHQTIEDARGFYSDLKGRLAKY.GRRPEE.L
Acinetobacter_sp._NIPH_2100_LLM                         TP.QGYPVIVQAGQSGPGRDLAARYAEVIFTANQKLEDAQEFYRDVKSRLAKY.GRSADE.L
Nostoc_sp._PCC_7107_WP_015115963.1                      PP.QGYPVIVQAGASEVGRDLAARTAEVIFTANQTLADAQEFYADVKGRLAKY.GRSPDD.L
Aspergillus_flavus_NRRL3357_uncharacterized             PV.QGWPVIVQAGQSEPGKQLAAETAEAVFCSPRDLESAKALYADIKGRAVAA.GRDRNH.L
Paenibacillus_curdlanolyticus_WP006037998.1             PI.QGHPVVIQAGASEPGKELAARTAEVIFTAWQTLEEAQRFYSDVKGRLARY.GRSPDD.L
LadAbeta_Geobacillus_thermoleovorans_FMN-dependen       PP.QGKPVLIQAGSSQDGIRFAAHVAEVVFTAQSTLTDAQRFYQTVKQEAARA.GRNPDH.I
SsuD_1M41                                               AIQQPYPPLYFGGSSDVAQELAAEQVDLYLTWGEPPELVKEKIEQVRAKAAAH.GRK....I
Paenibacillus_curdlanolyticus_WP006037998.1             PI.QGHPVVIQAGASEPGKELAARTAEVIFTAWQTLEEAQRFYSDVKGRLARY.GRSPDD.L
Bacillus                                                PVQKPSPPIYFGGSSPAGQKVAAKHSDVYLTWGEPPEQVREKIAAVRKQAEAE.GRT....V
Pseudomonas                                             LQ.RPYPPLYFGGSSEAAHELAGEQVDVYLTWGEPLPAVAAKIADVRQRAARH.GRT....V
LadB_Geobacillus_thermoleovorans_FMN-dependent          KP.AQRPPIYFGGASDIAKEVAAKEADVYMMWGETFERMKERIQEMKQKAAHY.GRT....L
Pseudomonas_aeruginosa_PAO1_NP_251290.1                 RQ.SPHIPIYFSGSSDAAIEVAARHADTYMLWGEPLAAVDGHIRRVRAAAKAQ.GRD....L
Alkanesulfonate                                         L..NGTIPVSVGGSSDEAYRIGGSLADIFGLWGEPLKETKEQIDRIYAEAAKA.GRAENDRP
Alkanesulfonate                                         TK.ETIPVSV.GGSSDDAYRIGGALADIFGLWGEPLKETKEQIEKIYAAAEKA.GRTDRP..
Aspergillus_niger_ATCC13496_RDH19503.1                  ANPNGTIPVSVGGSSDEAYRVGGSLADIFGLWGEPLKETKEQIDRIYAEAERA.GRAPNDRP
Penicillium_digitatum_Pd1_XP014534980.1                 TG.DSIPVSV.GGSSPEAYRIGGSLADIFGLWGEPLKETKEQIERIYAEAAKA.GRPESDRP
Acinetobacter_rudis_WP_016657872.1                      VQ.KPYPPLYFGGSSTVAQDLAAKQVDVYLTWGEPPAAVAEKIAQIRAKAAQQ.GRE....V
```

```
                                                      280       290       300       310       320
LADA_Geobacillus_thermodenitrificans_WP_011888513  KMFAGICVIVGKTHDEAMEKLNSFQKYWSLEGHLAHYGGGT.GYDLSKYS...SNDY.I...
Geobacillus_sp._LEMMY01_WP_079935807.1:1-440       KMFAGICVIVGKTHDEAMEKLNSFQKYWSLEGHLAHYGGGT.GYDLSKYS...SNDY.I...
Geobacillus_stearothermophilus_ABV66256.1:1-440    KMFAGICVIVGKTHDEAMEKLNSFQKYWSLEGHLAHYGGGT.GYDLSKYS...SNDY.I...
Geobacillus_sp._B4113_201601_WP_061912600.1:1-440  KMFAGICVIVGKTHEEAMEKLNSFQKYWSLEGHLAHYGGGT.GYDLSKYS...SNDY.I...
Geobacillus_sp._B4113_201601_5WP_061912600.1:1-44  KMFAGICVIVGKTHEEAMEKLNSFQKYWSLEGHLAHYGGGT.GYDLSKYS...SNDY.I...
Bacillus_thuringiensis_6WP_029441578.1:1-435       KLFAGICVIVGKTYEEAIEKFDSFQKYWSLEGNIAHYAGGS.GYDLSQYN...INDY.I...
Lysinibacillus_sphaericus_WP_103976887.1:2-422     KMFMGLCIVTGLTDEEINSKVDLYKSFWSLEGNMAHYCGGQ.GIDLSKYS...QDDN.I...
Lysinibacillus_sphaericus_WP_069508309.1:2-422     KMFMGLCIVTGLTDEEINSKVDLYKSFWSLEGNMAHYCGGQ.GIDLSKYS...QDDN.I...
Rummeliibacillus_sp._SL167_10_WP_146551437.1:2-42  KFFMGLCIITGLTDDAIREKIELYKAFWSVEGNLAHYCGGQ.NLDLSKYE...KEDI.L...
Paenibacillus_sp._DXL2_WP_110843609.1:1-459        KLFAGVSVIVGRTKEEAERKYEDYKRLYSVEGVLAHYGGGS.GYDLSAYD...PDDY.L...
Paenibacillus_naphthalenovorans_WP_074730028.1:1-  KLFAGVATIVGRTKEEAQRKYEEYARLYSVEGVLAHYGGGS.GYDLSAYD...PEDD.L...
Cohnella_sp._HS21_WP_130606586.1:1-453             KVFMAINSIVGRTRAEAEEKLREYTAIRSDEAPQVLYGGFS.GIDLSKYD...RSDY.L...
Streptosporangium_subroseum_15_WP_089205784.1:1-4  KVFPGASVIVAETQREADAKVADLQRHIRAAGFLAQRGS...GIDLAAYD...PADS.IDDI
Arthrobacter_sp._Hz2_WP_120147654.1:1-455          KAFASAVVIVGRTHKEAQRKADEYRRLSSAEGYLAHAGGG..GIDLAAYR...PDEV.IDDI
Nonomuraea_solani_WP_103963668.1:1-453             TFFPSASVIVARTQFEVDAKLADLQRHIRPEGFLAQRGS...GIDLAAYD...PGEL.I...
Arthrobacter_ruber_WP_105030338.1:1-455            KAFASAVVIVGRTSKGAQRKADEYRRLSSAEGYLAHAGGG..GIDLAAYR...PDEV.IDDI
Bacillus_aciditolerans_20_WP_121446947.1:1-456     KAFTFLNVIVGETSEQAEEKYAELARYWSAEAAKAQYGGSS.GYDLSQYT..DLDSP.F...
WP_137163763.1:1-458                               LIYAQALLIVAPTREEAEARFAEYRRYVDLDAALALLSGWT.GIDFAGLD...PDAP.I...
Aeribacillus_pallidus_WP_130157503.1:4-457         KVFALFTPIVGRTEEEAWAKYEELSQYISYEGALSLLGGWS.GVDFSKYD...PDQE.I...
Bacillus_megaterium_24WP_098602848.1:4-460        KVFALFTPIVGRTEEEAWKKYEELSNYISYEGALSLLSGWS.GIDFSKYS...PDQE.I...
Streptomyces_sp._150FB_WP_078877664.1:5-455        KFMAGASVIVGKSDEDVARKVEEFTALRSVDGYLAHSGA...GLDWTRYS...PETR.IGDI
Arthrobacter_sp._DWC3_WP_104051071.1:1-454         KAFASAVVIVGRTAKDAQRKADEYRRLSSAEGYLAHAGGG..GIDLAAYR...PDEV.I...
Cohnella_thermotolerans_WP_027093898.1:1-456       KAFTFLNVVVAETTEEAERKFAEYSRLWSGEAAKAQYGGSS.GYDLSQYK...DLDA.IFEY
Paenibacillus_bovis_28WP_144921429.1:1-452         KILTMVTPVVGRTEEEAQEKYNEYKKHISYEGALALFGGWT.GVDLSGYD...PSTI.V...
Bacillus_aciditolerans_WP_121447760.1:1-452        KILACITPIVGQTEEEAQDKYNEYQKHVSYEGALALLGGWT.GIDFSEYD...PDEN.L...
Leifsonia_sp._ALI-44-B_30_WP_077053645.1:1-455     KTFASAVVIVDRDRDAALAKAEEYKRYSSDEGYLTHSGGG..GIDLAAYP...KDAL.ISDI
Parageobacillus_thermoglucosidasius_WP_064552269.  KVFAFLTVIVGKTTEEAEQKFAELNHLWSPDASKAQFSGAS.GYDLAEYENKDLNAP.F...
Geobacillus_sp._Y4.1MC1_WP_013399886.1:1-459       KVFAFLTVIVGKTTEEAEQKFAELNHLWSPDASKAQFSGAS.GYDLAEYETKDLNAP.F...
Bacillus_methanolicus_35WP_004434428.1:1-462       KVFSFLTVIVGETTEEAERKYRQELNRLWSPDAAKAQFGGAS.GYDLSQYEKSDLDQP.F...
Terribacillus_halophilus_WP_077309754.1:1-448      KVMSLFTPIVAETEEAAQLKLNEYRSYASYEGGLVLLGGWT.GIDFSEYD...PEEE.L...
Paraburkholderia_kururiensis_WP_017777532.1:1-453  LIFNLHTVITGRTEAEAHAKHADYRRYASDEGALALMSGWT.GIDLSKYD...LDEP.L...
Gracilibacillus_massiliensis_WP_058308419.1:1-456  KIISFLSVVVAETTEEAEAKFEEYNKVWSADAAKAQY.GAS.GYDIAEYEDADPDEP.F...
Streptomyces_yanglinensis_WP_103888551.1:5-452     KFMAGAAVIVGSTERDVARKVEEFTALRSVDGHLAHSGA...GLDWTRYS...PDTR.VGDI
Trinickia_sp._7GSK02_40_WP_136896361.1:1-452       LIFNLHTVITGRTEAQAHAKHADYRRYASDEGSLALMSGWT.GIDLSKYG...LDEP.L...
Cryobacterium_sp._NEAU-85_WP_123045138.1:1-448     RFLIGLDVIVGRTEAEAKAKVADLLQYRDLIGYLVHLGGGS.GIDLAAMG...AEEY.I...
Alicyclobacillus_acidoterrestris_WP_021298468.1:4  RVLALFTPIVGRTQAEAEEKYAEYKAHASYDGALSLFGGWT.GIDLSNYS...PNEN.L...
Oceanobacillus_senegalensis_WP_085994229.1:1-452   KIFCLFTPIVGKTQTEAEEKYLDYQKHGSYDGALALFGGWT.GIDFSGYD...PDEK.L...
Bacillus_sinesaloumensis_WP_077620983.1:1-449      KIISFLSVIVAETTEEAEQKFQEYNKMWSPDAAKAQY.GAS.GYDIAEYEDVDPDEP.F...
Halopenitus_persicus_WP_021074147.1:6-457          HVFAAIAPIVAPTEEEAQRKYAELEEYVSHEGALALVGGWT.DIDFAEYD...PDER.V...
Microbacteriaceae_bacterium                        RFLIGLNVVVGRTEEEARAKLEELLPYRDVVGYLVHFGGGS.GIDLASAG...AEEY.L...
Lysinibacillus_sinduriensis_WP_036201100.1:1-458   KTFTFLNVVAETTEEAENKFEQYRHFWSSEAAKAQFGGSS.GYDLAKYT...DKDAPF...
Bacillus_endophyticus_WP_113749053.1:5-457         KVFALFTVIVGRTEEEAWKKYEELSNYISYEGALSLLSGWC.GVDFSKWD...PNQE.I...
Pseudomonas_monteilii_WP_119371117.1:1-455         LIFNLQTVIVDETDAKAQAKWQELKSYASYEGALALISGWT.GIDFSKYQ...PDQV.L...
Bacillus_endophyticus_50_WP_061801645.1:5-457      KVFALFTVIVGRTEEEAWKKYEELSNYISYEGALSLLSGWC.GVDFSKWD...PKQE.I...
Planococcus_maitriensis_WP_112233285.1:1-457       LVYTTFTAIVGKTEQEAQEKYETLKSHVSYEGALALLSGWT.GIDFSGYE...PDDT.L...
Oceanobacillus_profundus_WP_118889880.1:1-447      KILTMFVPIVGKTEAEAKEKYKDYQQYISAEGALALFGGWT.GVDLSQYA...KDQE.I...
Burkholderia_pseudomallei_WP_141404579.1:1-452     LIFNLHTVITGRTSADAHAKHADYRRYASDEGALALMSGWT.GIDLSKYA...LDEP.L...
Terribacillus_saccharophilus_WP_095261886.1:1-455  KIFSFLSVIVAKTTEEAQQKYQAYTSHWSSDAAKAQF.GAS.GYDISEYEELDPNEP.F...
Sediminibacillus_albus_WP_093212831.1:4-453        KILTMFTPIVGRTQEAYQKLEDYKQHISHEGALALFGGWT.GIDLSEYG...PEDS.L...
Sciscionella_marina_WP_020496929.1:1-448           RFLVGLNVIVGETENEARRKLDEILRYRDTIGYLVHFGGSS.GIDLAALG...AEEY.L...
Mycetocola_saprophilus_WP_043321267.1:1-452        KALASINIVLGETEAEAKRKTEEVLALRDVHGYLVHLGGGT.GIDFSAVA...NNEK.L...
Lysinibacillus_macroides_WP_053993569.1:4-460      KVFALFTPIVGETEQEAWAKYDELASYVSLEGALTLLSGWS.GVDFSQYD...LDEE.L...
Pseudomonas_putida_WP_086978777.1:1-458            LIFNMQTVIVDETDAKAQAKWQELKSYASYEGALALISGWT.GIDFSKYQ...PDQV.L...
Sediminibacillus_halophilus_60_WP_074598704.1:4-4  KILTMFTPIVGRTEQEAREKFNDYKNHISHEGALALFGGWT.GIDLSQYG...PDES.L...
Rhizobium_oryzae_WP_085420425.1:1-450              KVFVGATIVTGRTEKEAREKFEDYRAHASAEGALVHAAASL.GIDFAKYD...LDEP.L...
Pseudomonas_extremorientalis_WP_071491956.1:1-455  LIFNLQTVIVGETDAKAKAKFEEYKSYVSYEGAMALISGWT.GIDFSQFK...PDEP.L...
Pseudomonas_fluorescens_WP_016977452.1:1-454       LIFNLQTVIVGETDAKAKAKFDEYKSYVSYEGAMALISGWT.GIDFSQFK...PDEP.L...
Neorhizobium_sp._T25_27_WP_105419890.1:1-450       KVFVGATIVTGRTEKEAREKFEDYRAYASSEGALVHAAGSL.GIDFAKYD...LDEP.L...
Pseudomonas_extremaustralis_WP_078833627.1:1-457   LIFNLQTVIVGETDAKAKAKFDEYKSYVSYEGAMALISGWT.GIDFSQFK...PDEP.L...
Fictibacillus_aquaticus_WP_094253544.1:1-455       KIICFLTVIVAETTEQAEAKYEEYNKLWSPDAAKSQFGGAS.GYDLSEYEKQDQDQP.F...
Pseudomonas_extremaustralis_KAA8559760.1:1-456     LIFNLQTVIVGETDAKAKAKFEEYKSYVSYEGAMALISGWT.GIDFSQFK...PDEP.L...
Mycobacterium_sp._ACS1612_WP_067812332.1:1-454     KIVNGHVVVTGATQQAAQARHDDFRRYIDSEGALALWSGWL.GTDLSRFN...LDDP.V...
Bacillus_megaterium_WP_116075167.1:1-455           LIFNLFTPIVGRTEKEAEEKLAQFESYVNFEGAATLFSGWT.GIDVSAYE...PEQP.I...
Pseudomonas_frederiksbergensis_70_WP_123409596.1:  LIFNLQTVILGETDAKAKAKFEEYKTWVSYEGAMALISGWT.GIDFSQFK...PDEP.L...
Pseudomonas_citronellolis_SFC61163.1:1-452         KFIVQAAVIVGRNDLEVQAKLEDYRRLQSLDGALAHMQS...GVDLAAFP...AHTP.L...
Herbaspirillum_chlorophenolicum_WP_050468599.1:4-  KVFMGLTVVTAATEQAARDKYREYASHASPEAGLAHFSAGT.GIDFSQYE...PDEP.I...
Arthrobacter_crystallopoietes_WP_074700854.1:1-45  KALASLNIVLGETEAEVARKVEEVVRLRDVEGHLVHFGGSS.GIDVASLE...TDEY.L...
Terribacillus_goriensis_WP_038558686.1:1-438       KAFTFLHVVVAETEEKALAKYKEVKQYWSADAAKAQYGGST.GYDLASYD...NEEE.LFTY
Bacillus_megaterium_WP_025749753.1:1-455           LIFNLFTPIVGRTEKEAEEKLAQFESYVNFEGAATLFSGWT.GIDVSAYE...PEQL.I...
Neorhizobium_sp._T7_12_WP_105403044.1:1-451        KVFVGATIVTARTEKEAREKFADYRAYASSEGALVHAAGSL.GIDFAKYD...LDEP.L...
Rummeliibacillus_sp._SL167_WP_146553619.1:4-459    KVLALATVIVAETEEKAQEKYDDLAQYISYEGALTLLSGWS.GIDFSQFD...PDEE.L...
Burkholderia_sp._PAMC                              KIFMGITVVVGRTHAEAEAKFAEYAHYASPEAGLAHFAAGT.GTDYSIFD...PDDE.I...
Pseudomonas_koreensis_WP_064585463.1:4-449         KVFMGLNVIVGETEEAWAWKHAEYLSYASAEAGVAHFSAST.GIDFSQYD...IDEP.I...
Pseudomonas_fluorescens_80_WP_064116961.1:6-451    KVFMGLNVIVGATEEAAWAKHAEYLSYASAEAGVAHFSAST.GIDFSQYE...IDEP.I...
Pseudomonas_rhodesiae_WP_040265276.1:4-450         KVFMGINVIVGATEALAWEKHAEYLSYASAEAGVAHFSAST.GIDFSQYE...LDEP.I...
Collimonas_sp._OK412_WP_092393355.1:4-454          KIFMGISVIVAPTAAEAREKYAEYCRYANPEAGLAHFSAGT.GFDFSKYG...IDEA.I...
Pseudomonas_marginalis_WP_058415544.1:1-456        LIFNLQTVIVGETDAKAKAKFDEYKAYVSYEGAMALISGWT.GIDFSQFK...PDEP.L...
Pseudomonas_antarctica_WP_064452947.1:1-458        LIFNLQTVIVGETDAKAKAKFHEEYKSYVSYEGAMALISGWT.GIDFSQFK...PDEP.L...
Pseudomonas_protegens_WP_041115159.1:9-454         KVFMGLNVIVGQTEELAWKKHAEYRSHASAEAGVAHFSAST.GIDFSQYE...IDEP.I...
Pseudomonas_synxantha_WP_057024776.1:4-449         KVFMGLNVIVGATEELAWRKHAEYLSYASAEAGVAHFSAST.AIDFSQYE...LDEP.I...
Rhizobium_leguminosarum_WP_128409501.1:2-451       KILSLITVVVGRTEKEARDKLEEYRRHASVEASLAHYSAST.GIDFSKYG...LDDV.L...
Pseudomonas_chlororaphis_WP_038359779.1:6-451      KVFMGLNVIVGATEEAAWAKHAEYLSYASAEAGVAHFSAST.GIDFSQYE...IDEP.I...
Pseudomonas_syringae_90_WP_024642725.1:1-456       LIFNLQTVILGETDAKAKAKFEEYKSWVSYEGAMALISGWT.GIDFSQFK...PDEP.L...
Collimonas_fungivorans_WP_061540754.1:4-453        KIFMGISVIVAPTAAEAREKYAEYCRHANPEAGLAHFASGT.GFDFSAYG...IDEA.I...
Rhizobium_leguminosarum_WP_116409027.1:2-451       KILNLISVVVGRTERQAQDRLEEYRRHASVEASLAHYSASI.GIDLAKYR...LDEV.L...
Microvirga_ossetica_WP_099511173.1:1-454           KVFVGMSLVVGRTEREANDKLEDYRRHASAEGALAHFSSSS.GVDFSKYD...LDEP.L...
Pseudomonas_caspiana_WP_087268376.1:4-451          KVFMGLNVIVGKTEADAQAKLAEYREFASAEAGVAHFAAST.GIDFSQYE...LDEP.I...
Pseudomonas_lundensis_WP_048375063.1:4-451         KVFMGLNVIVAPTEQQAHAKREEYLSYASAEAGVAHFSSST.GIDFSDYE...LDEP.I...
Caballeronia_mineralivorans_WP_047894899.1:4-456   KVFMGLTVVVGRTTTEAEEKFAEYARYASAEAGLAHFAAGS.GIDYSTFD...LDDE.L...
Pseudomonas_paralactis_WP_057702387.1:4-450        KVFMGLNVIVGATEALAWAKHAEYLSYASAEAGVAHFSAST.AIDFSAYA...LDEP.I...
Pseudomonas_taetrolens_WP_048381143.1:4-453        KLFMGLNVIVAPTEAQALAKRDDYLSHASAEAGVAHFSSST.GIDFADYE...LDEP.I...
Pseudomonas_psychrophila_WP_123750761.1:4-450      KVFMGLNVIVAPTEAQALAKREEYLAYASAEAGVAHFSSST.GIDFADYE...LDEP.I...
Collimonas_arenae_100_WP_061532973.1:4-454         KIFMGISVIVAPTEAEAREKYAEYCRYANPEAGLAHFSAGT.GFDFSTYG...IDQP.I...
Pseudomonas_viridiflava_WP_122210720.1:4-451       KVFMGLNVIVAATEALAQEKHAEYRQHASAEAGVAHFAAST.GIDFAQYE...LDEP.I...
Kocuria_varians_WP_068470402.1:1-454               KIYAMLSVVVADTDEEAGEKYRDLQYVDLEASQAIIGGWS.GVDLSQFP...EDEA.L...
Pseudomonas_wadenswilerensis_WP_115084816.1:4-455  KVFMGLNVIVARTEEQAKAKHAEYLRYASAEAGVAHFASST.GIDFSEYE...LDEP.I...
Pseudomonas_syringae_WP_011266344.1:4-452          KVFMGLNVIVAATEALAREKHAEYRRHASAEAGVAHFAAST.GIDFSQYE...LDEP.I...
Pseudomonas_sp._286_105WP_122415793.1:4-453        KVFMGLNVIVAATEALAQEKHAEYRRHASAEAGVAHFAAST.GIDFAQYE...LDEP.I...
Geobacillus_vulcani_WP_031406843.1:1-461           KFFTFLTVIVAKTTEQAEAKYEQLRRLWSMDAAKAQFSGAS.GYDLAEYEAKPADEP.F...
Geobacillus_icigianus_WP_033018365.1:1-461         KFFTFLTVIVAKTTEQAEAKYEQLRRLWSTDAAKAQFSGAS.GYDLAEYEAKPADEP.F...
Pseudomonas_floridensis_WP_083182568.1:4-453       KVFMGLNVIVAATEAQAWEKHAEYRRHASAEAGVAHFAAST.GIDFAQYG...LDEP.I...
Pseudomonas_asturiensis_WP_073167523.1:4-451       KVFMGLNVIVAATQALAQEKQAEYRRHASAEAGVAHFAAST.GIDFAQYE...LDEP.I...
Geobacillus_vulcani_110_WP_031406843.1:1-461       KFFTFLTVIVAKTTEQAEAKYEQLRRLWSMDAAKAQFSGAS.GYDLAEYEAKPADEP.F...
```

```
Pseudomonas_syringae_WP_024682626.1:4-452                 KVFMGLNVIVAATEALAREKHAEYRRHASAEAGVAHFAAST.GIDFSQYE...LDEP.I...
LadAalpha_Geobacillus_thermoleovorans_BAM76377.1          KFFTFLTVIVAKTTEQAEAKYEQLRRLWSMDAAKAQFSGAS.GYDLAEYEAKPADEP.F...
Tsukamurella_pseudospumae_WP_068746786.1:1-457            KVYTLLTIVTDETEAKAQAKLEEYHRYASVEGSLVFMSGWM.GVDLGAYD...PSDP.V...
Kocuria_sp._Marseille-P3598_WP_085529073.1:5-456          KIFAMLSVIVDESDEKAQAKYEDYLQYVSTESSQAIIGGWS.GVDLSEFG...EDEV.L...
Microvirga_aerophila_WP_114188789.1:1-456                 QLFLGRAIVVGRTRKEAEEKYEEYLSYASAEGALAHFSSTV.GIDFSQYD...LDEP.L...
Burkholderia_ubonensis_WP_071760483.1:5-455               RIFAGVTVVTAENERLAREKFDEYRRYASPEAGLAHFASST.GIDFAKYG...LDEP.I...
Achromobacter_marplatensis_WP_006226823.1:4-450           KFFMGVTAVVGRTEAEAREKHAEYLRYANPEAGLAHYASST.GIDFSRYG...ANEP.I...
Acinetobacter_baumannii_WP_070165746.1:1-443              KIYALLSIVTDETDAKAQAKFKEYQSYGSYDGALTLLSGWS.GVDFSQYQ...PTDK.V...
Labedella_phragmitis_WP_128496094.1:1-453                 KIYTLLTIITDETSEKAHAKHADYLRYASEEGALVFMSGWM.GIDLSRYD...PDEP.I...
Nocardia_cyriacigeorgica_120_WP_130918184.1:1-453         RIYALATIVTDASDEAAHRKHEEYLSYASVEGGLVFMSGWM.GIDLSNYD...LDDP.I...
Nocardia_cyriacigeorgica_WP_130918184.1:1-453             RIYALATIVTDASDEAAHRKHEEYLSYASVEGGLVFMSGWM.GIDLSNYD...LDDP.I...
Mycobacterium_sp._1164985.4_WP_067298521.1:1-453          KIFNLSTIVTASTDEEAHVRHAEYLSYGDPEGALTFMSGWM.GVDLSRYG...LDEP.V...
Rhodococcus_sp._1R11_WP_135042739.1:1-449                 RIYQLLTIVTDETSELAHAKHDEYRSYASAEGALVFMSGWM.GVDLSRYA...LDEP.L...
Erwinia_amylovora_WP_004157587.1:5-459                    LIFNLQTAIVGAMDQEAQAKWLDYKSYVSYEGALALISGWT.GIDFGQYQ...PDQV.L...
Ralstonia_mannitolilytica_WP_102079134.1:4-451            KVFMGAAVVTGRTEAEAHAKHADYRAYASREAGLAHFAAST.GVDFSRHG...LDDP.I...
Leifsonia_flava_WP_135120808.1:15-465                     RIYTLLTIITDATPEAAQAKYEDYLSYASEEGALVFMSGWM.GIDLSQYD...LDEP.V...
Cryobacterium_roopkundense_WP_084141147.1:1-453           KIYTLLTIITDASSEKAQAKYQDYLSYASEEGALVFMSGWM.GIDLSTYD...LDEP.I...
Cupriavidus_pauculus_WP_101681197.1:1-455                 KVFLGATLVVGRTDAEAREKFEEYRRYVSSEAALVHAAASL.GIDFARYD...LDEP.I...
Rhizobium_sp._BK333_130_WP_133704837.1:2-454              SILNLITVVVGRTEMEAEDRLEDYRRHASVEASLAHYSSSI.GIDLSKFG...MDEP.I...
Caballeronia_mineralivorans_WP_047846789.1:1-454          LIYNLVTVIVDETDAKAQAKFEEYRRYTSYDGSLVFMSGWT.GVDFGQYA...PTDL.V...
Nocardia_altamirensis_WP_069163049.1:1-454                RIYALATIITDATDEAARHKHEEYLSYASVEGGLVFMSGWM.GIDLSTYD...LDDP.I...
Cupriavidus_sp._P-10_WP_116322758.1:1-452                 VVFAMMNVIVGRTDEEAQEKLREYKQYASYEGALTLMSGWT.GIDFSSCD...PQDE.V...
Serratia_sp._S1B_WP_116726564.1:1-452                     KIYTLLSIVTDSSDAKAQQKFKEYQSYGSYDGALTLLSGWS.GVDFSQYQ...STDK.V...
Pseudomonas_putida_WP_043864435.1:1-452                   LVLEQLTVIVAPTDEQALARLREYREYASASGALTLMSGWT.GIDFAQYQ...SDQV.L...
Pseudomonas_aeruginosa_WP_023084248.1:4-453               RIFMGINVIVAATAAEARDKHAEYRRYASPEAGLAHFASSS.GIDLSRYG...LDEP.I...
Pseudomonas_mucidolens_WP_084381795.1:9-455               KVFMGLNVIVGATEELAWAKHAEYLSYASAEAGVAHFSAST.GIDFFQYE...LDEP.I...
Pseudomonas_poae_WP_060549803.1:4-450                     KVFMGLNVIVGATEELAWAKHAEYLSYASAEAGVAHFAAST.AIDFAEYE...LDEP.I...
Pseudomonas_chlororaphis_WP_123573412.1:9-454             KVFMGLNVIVAATEELAWKKHAEYRSYASAEAGVAHFSAST.AIDFSQYE...IDEP.I...
Janthinobacterium_lividum_140_WP_072454679.1:1-44         LIYAQALVITGATEEEARAKHAGYLRHVNIEAALVLLSGWT.GVDFSRYP...LDAT.I...
Haladaptatus_cibarius_WP_049970513.1:4-446                AFFAGIAPIVGETEEIAQAKYETYRENVDTEATLTLLGGFM.DLDFSELA...PDQP.V...
Arthrobacter_luteolus_WP_066298473.1:1-443                RIYALVTAVVADSDAAAEKLYREYLSYSSREGALTFYGGWS.GLDLSRYA...PEEE.L...
Geobacillus_sp._ZGt-1_WP_047758532.1:1-436                KFFTFLTVIVAKTTEQAEAKYEQLRRLWSMDAAKAQFSGAS.GYDLAEYEAKPADEP.F...
Lysinibacillus_composti_WP_124766485.1:4-458              KILTMFVPIVGRTEEEAKQKYEDYRQYISKEGALALFGGWT.GVDVSGYA...PDQV.I...
Microcella_putealis_WP_130486201.1:20-474                 RIYTLLTIITDATPEAAQAKYRDYLSYANPEGALVFMSGWM.GIDLSQFP...LDEP.V...
Pseudomonas_nitroreducens_WP_037016976.1:1-453            LVLNQLTVIVAPTDAEALAKLEDYRQYSDREGALALVSGWT.GIDFGQYA...PDQV.L...
Acinetobacter_wuhouensis_WP_130168784.1:1-453             KIYTLLSIVTDDTDQKAQEKFKEYQSYGSYDGALTLVSGWS.GVDFSQYQ...PHDQ.V...
Bradyrhizobium_sp._YR681_WP_008144663.1:1-454             LMFNMMTIILGNTEAEAAAKYADYRSHISPEGALALMSGWT.GIDFSGYE...LDQQ.V...
Schumannella_sp._10F1D-1_WP_141164655.1:10-460            KIYTLLTVITDATPEKAQAKLEDYLQYTSAEGALTFMSGWM.GVDLSQYE...LDEP.V...
Pseudomonas_coronafaciens_150_WP_122355187.1:4-45         KVFMGLNVIVAATEALAREKHAEYRRYASAEAGVAHFAAST.GIDFAEYE...LDEP.I...
Pseudomonas_syringae_WP_003430238.1:4-452                 KVFMGLNVIVAATEALAREKHAEYRRHASAEAGVAHFAAST.GIDFSRYE...LDEP.I...
unclassified_Rhodococcus_WP_094682261.1:1-449             RIYQLLTIVTDETSELAHAKHDEYRSYASAEGALVFMSGWM.GVDLSRYA...LDEP.L...
Hafnia_alvei_WP_046449330.1:6-449                         KVFMGVGVIVAKTEAEAKEKHHEYLRYASPEAGLAHFSSST.GIDLASFD...LDEP.I...
AS9A_3890_Hoyosella_subflava_WP_013808677.1:1-453         RIYALATIITDRSDAAAAAKHEEFKRYASLEGGLVFTSGWM.GIDLSRYD...LDEP.I...
Acinetobacter_baumannii_WP_000995600.1:1-443              KIYALLSIVTDETDAKAQAKFKEYQSYGSYDGALTLLSGWS.GVDFSQYQ...PTDK.V...
Agrobacterium_sp._B133/95_WP_065696417.1:2-454            RILNLITVVVGRTEKEAEDKLEDYRRHASVEASLAHYSSSI.GIDLSKFS...LDEP.I...
Microbacterium_sp._KROCY2_WP_024289149.1:1-453            KIYAMLSVVVDETDEKAQAKYREYQSYANLEASQAIIGGWS.GLDLSKFQ...EDEV.L...
Variovorax_paradoxus_WP_012747040.1:6-452                 KVFVGAAVVPGASTKEAQEKYADYRRYASREAGLAHFSAST.GIDYARYG...LDEP.I...
Methylobacterium_radiotolerans_WP_076730229.1:5-4         KVFVGLAIIPGRTRAEAEAKRDEYLSYASPEAGLAHFSAST.GIDFARYG...LDEP.I...
Streptomyces_sp._BK215_WP_132915265.1:7-452               SFFQGLSFVVGSTEEEARRKNAEIDEMLSVEGLLTHMSGNI.GVDLSAVD...LDKP.L...
Geobacillus_thermodenitrificans_160_WP_008880416.         KFFTFLTVIVAKTTEQAKAKYEQLRRLWSADAAKAQFSGAS.GYDLAEYETKPPHEP.F...
Pseudomonas_aeruginosa_WP_132556951.1:356-785             RIFMGINVIVAATAAEARDKHAEYRRYASPEAGLAHFASSS.GIDLSRYG...LDEP.I...
Penicillium_solitum_OQE02104.1:14-471                     KFLALLCPILGKTEEEAVEKFEYFRSLGSIDGALALFGGWT.GIDLDKYG...DDEE.L...
Penicillium_vulpinum_OQE05502.1:12-469                    KFLALLCPILGKTEEEAVEKFKYFRSLGSIDGALALFGGWT.GIDLDTYG...DDEE.L...
Clavibacter_michiganensis_WP_045526194.1:1-454            RILTLATVIVAETDEAAAALLEEYRGYVSLDGALALYGGWS.GLDLSGYD...PDAP.L...
Glutamicibacter_creatinolyticus_WP_054821484.1:6-         KIFAMQTIITGQDSATAQAKYEDYKSYADVEGALVLISGWM.GVDLSAYD...PDDV.I...
Campylobacter_sp._P0078_WP_086237522.1:4-454              KIYVLATIITDKTQKLAQAKYNDLLSYVSEEGSLVLNSGWL.GENLGKYA...LDDL.L...
Pseudomonas_furukawaii_WP_003454569.1:1-447               KVFMGINAIVAPTEAEALDKLAEYRRHASPEAGIAHFSAST.GIDLAAFG...LDEP.I...
Leucobacter_triazinivorans_WP_130111548.1:1-442           KIYLLLTIIVDETDELAQKKHEEYLSYVSPEGALTFLSGWM.GVDLSKYD...LDEP.L...
Aspergillus_brasiliensis_CBS_101740_OJJ68286.1:12         KFLALLCPVLGRTEEEAQEKFRYFRSLGSIDGALALFGGWT.GIDLDRYG...EDEE.L...
Aspergillus_udagawae_170_GAO89440.1:15-472                KFLALLCPVLGRTEEEAREKFQYYRSLGSIDGALALFGGWT.GINLDTYG...DDEE.L...
Aspergillus_flavus_RMZ37351.1:15-472                      KFLALICPVLGRTEEEAQEKFKYYRSLGSIDGALALFGGWT.GINLDTYG...DDEE.L...
Aspergillus_flavus_RAQ54081.1:15-472                      KFLALICPVLGRTEQEAQEKFKYYRSLGSIDGALALFGGWT.GINLDTYG...DDEE.L...
Penicillium_arizonense_XP_022490779.1:12-469              KFLALLCPVLGKTEEEARTKFEYYRSLGSIDGALALFGGWT.GIDLDKYG...DDEE.L...
Salinibacterium_sp._CGMCC                                 RIYTLLTVITDATPEKARAKHAEYLSYASEEGALVFMSGWM.GIDLSQYD...LDEP.V...
Aspergillus_fumigatus_OXN06785.1:14-471                   KFLALLCPVLGRTEEEAREKFQYYRSLGSIDGALALFGGWT.GINLDTYG...DDEE.L...
Aspergillus_lentulus_GAQ10210.1:7-464                     KFLALLCPVLGRTEEEAREKFQYYRSLGSIDGALALFGGWT.GINLDTYG...DDEE.L...
Aspergillus_flavus_RAQ66103.1:15-472                      KFLALICPVLGRTEEEAQEKFKYYRSLGSIDGALALFGGWT.GINLDTYG...DDEE.L...
Aspergillus_fumigatus_OXN22894.1:14-471                   KFLALLCPVLGRTEEEAREKFQYYRSLGSIDGALALFGGWT.GINLDTYG...DDEE.L...
Aspergillus_nidulans                                      KFLALLCPVLGKTEEEAKEKFAYYRSLGSIDGALALFGGWT.GINLDVYG...DDEE.L...
Penicillium_nalgiovense_OQE79848.1:14-471                 KFLALLCPILGKTEEEAVEKFKYFRSLGSIDGALALFGGWT.GIDLDKYG...DDEE.L...
Aspergillus_wentii                                        KIIVGINVIVAATDEEAKAKREEYLSYADEEGALALFGGWT.GVDLSSHS...DDED.F...
Gordonia_iterans_WP_105943624.1:1-453                     KIYTLVTVITASTPEAAREKAAEYQRYASDEGALVFMSGWM.GIDLSHYD...LAAP.I...
Azotobacter_beijerinckii_WP_090735436.1:4-454             KLFMGLTVIVAPSEAEARDKHAEYLRYASPEAGLAHLSSSI.GIDLAAYG...LDEP.I...
Staphylococcus_sp._NAM3COL9_WP_057513692.1:4-453          LVFPMVLPIIGETEEEAYQKYETLTEHISYEGSASLLSGHT.GIDFSEYD...PDHY.I...
Acidovorax_sp._56_WP_099656603.1:11-450                   KIVLGVSVVPGRTEAEARDKDAEYRRYANPEAGLAHFAAGT.GIDFARYG...LDEP.I...
Paenibacillus_sp._BIHB4019_WP_099518466.1:1-456           KVFTGLSVVVAQTREEAQRKFDEYMRHQSAEATLSSYAGVT.GIDLTQLD...PNAY.F...
Penicillium_antarcticum_OQD90033.1:5-465                  KIVAGALVIVAETDEAAQAKFDELKTYGDREGALALFGGWT.GYDLSTYD...DDQD.F...
Aspergillus_oryzae_RIB40_190_XP_001823703.1:9-463         KIIVGINAIVAATDEEAKAKREEYLRYADEEGALALFGGWT.GVDLSGYT...DDED.F...
Fusarium_oxysporum_f._sp._pisi                            KFLAMFCPIIGKTQEEADAKYKEFVGYGSEDGALALFGGWT.GIDLAQYG...DDEE.L...
Colletotrichum_fructicola_Nara_gc5_ELA35345.1:18-         KIILGINVIVAATDEEAEAKRNDYLKYADTEGALALFGGWT.GVDLSGYP...DDED.F...
Micrococcus_luteus_WP_041104460.1:19-468                  KIFAMQTIVTGATDEDARAKYNDLAQYVDPLGGLVLMSGWM.GIDLSQYD...LDEP.I...
Aspergillus_awamori_GCB21923.1:12-469                     KFLALLCPVLGRTEEEAQEKFRYFRSLGSIDGALALFGGWT.GIDLDRYG...DDEE.L...
Aspergillus_niger_CBS                                     KFLALLCPVLGRTEEEAQEKFRYFRSLGSIDGALALFGGWT.GIDLDRYG...DDEE.L...
Aspergillus_lacticoffeatus_CBS_101883_XP_02545389         KFLALLCPVLGRTEEEAQEKFRYFRSLGSIDGALALFGGWT.GIDLDRYG...EDEE.L...
Aspergillus_wentii_DTO_134E9_OJJ40237.1:14-471            KFLALLCPVIGNTEEEAQEKFKYYRSLGSIDGALALFGGWT.GIDLDKYG...DDEE.L...
Aspergillus_indologenus_CBS_114.80_PYI31207.1:11-         KFLALLCPVLAATEEEAQAKFREYQSYGDVEGALALFGGWT.GIDLSTYG...EEEE.L...
Aspergillus_fischeri_NRRL_181_XP_001264002.1:14-4         KFLALLCPVLGRTEEEAREKFQYYRSLGSIDGALALFGGWT.GINLDTYG...DDEE.L...
Penicillium_coprophilum_200_OQE46388.1:11-466             KVIVGINVIVAATNEEAEAKREDYLQFADDEGALALFGGWT.GIDLSNHA...DDED.F...
Cupriavidus_taiwanensis_WP_116379876.1:8-454              KVFMGAAVVTGRTEAEARARHAEYRDYASREAGLAHFAAST.GVDFARYG...LDDP.V...
Penicillium_digitatum                                     KVIAGALVIVAETDEEATAKFEELKTYGDREGALALFGGWT.GYDLSTYS...DDQD.F...
Aspergillus_niger_GAQ46262.1:12-469                       KFLALLCPVLGRTEEEAWEKFRYFRSLGSIDGALALFGGWT.GIDLDRYG...EDEE.L...
Aspergillus_nomius_NRRL_13137_XP_015412385.1:15-4         KFLALLCPVLGRTEEEAQEKFKYYRSLGSIDGALALFGGWT.GINLDTYG...DDEE.L...
Aspergillus_aculeatus_ATCC_16872_XP_020055010.1:1         KFLALLCPVLGATEEEARNTFKEYQSYGDVEGALALFGGWT.GIDLSQYG...EEEE.L...
Penicillium_brasilianum_OOQ84969.1:19-474                 KVIVGINVIVAATDAEAEAKREEYLQYADAEGALALFGGWT.GIDLSSYS...DDED.F...
Penicillium_subrubescens_OKP15100.1:19-474                KIIVGINVIIAATDAEAEAKREEYLQYADSEGALALFGGWT.GIDLSGYS...DDED.F...
Trichoderma_arundinaceum_RFU80530.1:19-474                KIIVGINVIVAATDEEAQALRESYVQYADQEGALALFGGWT.GVDLSSYS...DDED.F...
Aspergillus_thermomutatus_210_XP_026614594.1:15-4         KFLALLCPVLGRTEEEAQEKFQYYRSLGSIDGALALFGGWT.GINLDTYG...DDEE.L...
Serratia_liquefaciens_WP_044551273.1:4-449                KVFMGVGVIVAPTAAEAQDKYREYLRYASPEAGLAHFSSST.GIDLSHFG...LDDP.I...
Micrococcus_luteus_WP_065572847.1:19-468                  KIFAMQTIVTGATDEDARATYDDLAQYVDPLGGLVLMSGWM.GIDLSQYD...LDEP.I...
Halolamina_rubra_WP_049979911.1:1-449                     SFFIGIVPVVAPTEAEAEAKYEALKDSIDVESTLALLSGFL.DMDLSELD...PDQK.V...
Acinetobacter_pittii_WP_075382847.1:7-452                 KVFVGITVVVAETHDLAVQKLNEYRQYASPEAGLAHYASST.GIDLSKFA...DDEA.I...
Corynebacterium_variabile_WP_052302534.1:2-464            VVYALLTVITDATEDAARAKAAEYRSYIDHEGALTLMSGWM.GVDFSSFD...LDDP.V...
Aspergillus_luchuensis_GAT21254.1:12-471                  KFLALLCPVLGRTEEEAWEKFRYFRSLGSIDGALALFGGWT.GIDLDRYG...EDEE.L...
```

```
Aspergillus_luchuensis                                   KFLALLCPVLGRTEEEAWEKFRYFRSLGSIDGALALFGGWT.GIDLDRYG...EDEE.L...
Aspergillus_bombycis_XP_022386026.1:15-472               KFLALLCPVLGQTEEEAQEKFKYYRSLGSIDGALALFGGWT.GINLDTYG...DDEE.L...
Agrobacterium_vitis_WP_070163524.1:1-454                 KIYTLITIITDETEGKAKAKFEDYKQYVSYDGSLVFMSGWS.GIDFGQYA...PTDV.I...
Pseudomonas_psychrotolerans_220_WP_074584491.1:5-        KLFMGLTVIVAPTAAEARDKHAEYLHYASPEAGVAHHSASV.GIDLAAYG...LDEP.I...
Micrococcus_yunnanensis_WP_135040276.1:19-468            RIFAMQTIVTGATDEDARAKYDDLAQYVDPLGGLVLMSGWM.GIDLSQYD...LDEP.I...
Acinetobacter_calcoaceticus_WP_004642549.1:7-452         KIFVGITVVVAETHDLAVQKLNEYRQYASPEAGLAHYASST.GIDLSKFD...DDEA.I...
Penicillium_brasilianum_CEO59775.1:15-472                KFLALLCPILGRTEEEAIEKFKYYRSLGSIDGALALFGGWT.GIDLDKYG...DDEE.L...
Aspergillus_cristatus_ODM22551.1:12-469                  KFLALLCPILGKTEEEAKEKFAYYRSLGSIDGALALFGGWT.GIDLDTYG...DDQE.L...
Pseudomonas_taiwanensis_WP_027907363.1:1-446             LIYAQALLIVAPTRQEAEARFAQYRRYVDLDAALALLSGWT.GIDFAGLD...PDAP.I...
Acidovorax_avenae                                        KVVVGLTAVPGATRREAEDKYQDYCRHASPEAALAHLSAGA.GIDFSTWR...HDTPLI...
Staphylococcus_pseudintermedius_WP_101431388.1:4-        LVFPMVLPIIGETEEEAYEKYEALQNEVSYEGSVALLSGHT.GIDFSQYD...PDEY.I...
Rhodococcus_triatomae_WP_081607437.1:2-451               SFIAGVSVVVAPTDSEARAKFEQQLPFISVEGTLARQSSLM.QLDFGEID...IDAP.L...
Alcaligenes_sp._RS4_230_WP_128393375.1:4-450             KIFVGIAVVTGQTQGQAWEKYEEYLRYASAQAGVAHFSSST.GIDFSRFG...LDEP.I...
Gibbsiella_quercinecans_WP_121552947.1:7-454             KIFMGISVIVAPTAREAQEKHQEYLRYASPEAGLAHFSSST.GINFSRFA...LDEP.V...
Acinetobacter_ursingii_WP_044435211.1:5-452              KIFLGITVVTGETEQIAQEKLAEYRQYASPEAGLAHYASSV.GMDLSKFA...DDEV.I...
Erwinia_typographi_WP_034887712.1:4-447                  RIFMGVSVIVAPSEREAQEKHRSYLEYASPEAGIAHFSSSI.GMDLAAFD...LDEP.I...
Trichoderma_harzianum_KKP01913.1:14-470                  KFLAMLCPIIGRTQEEADAKYADYIQYGSEDGALALFGGWT.GIDLAQYD...DDQE.L...
Fusarium_graminearum_235_CEF79766.1:12-466               KVLALVTPILGRTEEEAKAKYDDYKQYASLEGALSLFGGWT.GMDLNQYG...DDEE.L...
Gordonia_alkanivorans_WP_006357654.1:10-461              KIYTLLTIITDSTPEAARAKAAEYQSYASEEGALVFMSGWM.GIDLASYN...IDEP.I...
Acinetobacter_radioresistens_WP_111281495.1:5-455        KIFIGITVVSAETDALAQEKLAEYCHYASPEAGLAHYSSSV.GIDLSQFT...EQET.I...
Mesorhizobium_plurifarium_WP_073985566.1:5-458           KFFAMATVVTGADDAEALAMHEAYRKVISTEGHLARLSAIL.QLDLSKLD...PDQP.L...
Oceanobacillus_halophilus_WP_121203049.1:1-454           RIFPMVLPIIGSTEAEAYAKYEDLKNHVSYEGTAALLSGHT.GIDFSVYN...PDQY.L...
Acinetobacter_tandoii_240_WP_100241535.1:7-457           KIFVGITVVTAETDTLAQEKLAEYIRYASPEAGLAHYSSSV.GIDLSKFA...DDEP.I...
Burkholderia_plantarii_WP_042624083.1:1-457              RLFAMLTVIVDSTAERAQARYAACRERASHEGALALLSGWT.GIDLARYR...PDDT.L...
Alcaligenes_faecalis_WP_094195208.1:6-457                KIYAGVAVVTGATEAEAKEKYADYLAHASSEGGLAHFAAST.GVDFSQYG...LDEP.I...
Bradyrhizobium_arachidis_WP_092214516.1:1-453            RFYALLTVIVGETHAEAEAKYRDYQRYIDPKGSLALLSGWT.GIDLSRYS...LDDP.F...
Cupriavidus_plantarum_WP_109583068.1:1-458               RIFAMFTVIVDETEALAKARHDAYRARVSYDGALALLSGWT.GIDLSRYA...PDDT.L...
Variovorax_sp._770b2_245_WP_093443018.1:5-461            RFVTTLQVVTGRTDTEARERFDEYRGYAQPEAGLVQFSSAI.GVDLSRFG...LDDP.V...
RZJ22128.1:4-447                                         KIMPGVSIFVAKTEQEAQEKYQLLNSLIHPKVGLSLLSGLSGGINLDKYD...LDAP.F...
LadB_Geobacillus_thermoleovorans_BAM76371.1              RYSISFQVVLGETEKEAWEQADALVSHLSESAKRK.......KDELIQKG.....DS.V...
LadAbeta_Geobacillus_thermoleovorans_BAM76372.1          LIMPGVAPYIGRTEQEAREQYEQLQELIVPEVGLAFLSDYLGGIDFSGYS...LDDP.L...
Af1_AFLA_024140_XP_002373925.1                           KFLALICPVLGRTEEEAQEKFKYYRSLGSIDGALALFGGWT.GINLDTYG...DDEE.L...
Af3_AFLA_58870_XP_002378631.1                            KVVAGILAIVAETDEAAHAKFAELAQYGDPEGALALFGGWS.GYDLSKYE...DDQD.F...
Af4_AFLA_049780_XP_002383105.1                           KFFATFTPIIGRTDDEAQAKYEELQKYASVVGGLVLFSGWT.GIDISRIP...LDQD.ITAA
Af5_AFLA_126060_XP_002381799.1                           KFIAGILVIVDETDEKAQAKYEEYLAQSDLEGVATLFGGWT.NNDLSKFD...DDED.F...
Af2_QMW32876.1:9-463                                     KIIVGINAIVAATDEEAKAKREEYLRYADEEGALALFGGWT.GVDLSGYT...DDED.F...
Yarrowia_lipolytica_CLIB122_YALI0C07546p_XP_50156        LVVSTLMVIVKATNQEAQDYYKELSDASDKEGALIFCSQMF.GADLKSYP...PGQD.L...
Rhizobium_sp._CF122_WP007794079.1                        KIMPGIFPVVGRTQAEAEAKFQEIQDLIHPVVGLSILNGMAEGVDLSKYP...LDGP.I...
Acinetobacter_sp._NIPH_2100_LLM                          LILPGVSAFVGRTEEEARAKYQQLTDLIHPEAGLALLSGLSGGFDFSGYD...LDGP.F...
Nostoc_sp._PCC_7107_WP_015115963.1                       KIMPGAFPIIGSTEEKAQEKYEFIQSLIHPDVAWGILKNYYKGVDLSKYS...FDDL.A...
Aspergillus_flavus_NRRL3357_uncharacterized              KILPAAFIVVGDSVEEARAKRLKLDSLVHYDSAIASLSIAL.GTDASGFD...PDGP.L...
Paenibacillus_curdlanolyticus_WP006037998.1              KIMPGVFPIIGRTEEEALEKKALFEQLIPEQAGVALLSALI.SFDLSGYP...VDGP.L...
LadAbeta_Geobacillus_thermoleovorans_FMN-dependen        LIMPGVAPYIGRTEQEAREQYEQLQELIVPEVGLAFLSDYLGGIDFSGYS...LDDP.L...
SsuD_1M41                                                RFGIRLHVIVRETNDEAWQAAERLISHLDDETIAKAQAAFA............RTDS.V...
Paenibacillus_curdlanolyticus_WP006037998.1              KIMPGVFPIIGRTEEEALEKKALFEQLIPEQAGVALLSALI.SFDLSGYP...VDGP.LPEL
Bacillus                                                 KFGIRLHIIVRETEEEAW............................................
Pseudomonas                                              KFGIRLHVIVRETDEEAWRAADRLIEHISDETIAAAQQSFA.RVDSEGQR............
LadB_Geobacillus_thermoleovorans_FMN-dependent           RYSISFQVVLGETEKEAWEQADALVSHLSESAKRK.......KDELIQKG.....DS.V...
Pseudomonas_aeruginosa_PAO1_NP_251290.1                  RFSVSFRPIVADTEEAAWKRAAEVLEQVRENRARL.......GLPLRDHQ...PQNV.....
Alkanesulfonate                                          RIWVTFRPIIAETEELAWAK..........................................
Alkanesulfonate                                          RIWVTFRPIIAETEELAWEK.....AHRTLDALQANRPGGQ.GNA........PSRP.....
Aspergillus_niger_ATCC13496_RDH19503.1                   RIWVTFRPIIAETDELAWAK.....AYQTLEKLKEHRANGQ.GKAPANAP...PPQN.V...
Penicillium_digitatum_Pd1_XP014534980.1                  RIWVTFRPIVAETEDLAWAK.....AHRTLDALNSNRANGQ.....SRVP...VNAP.....
Acinetobacter_rudis_WP_016657872.1                       KFGIRLHVIVRRTNEEAWTAANQLIEHISDETIAKA......QQSFARFD...SVGQ.....
```

```
                                                         330               340       350
LADA_Geobacillus_thermodenitrificans_WP_011888513      .........GSI.........................SVGEIINNMSKLDGKWFKLSVGTP
Geobacillus_sp._LEMMY01_WP_079935807.1:1-440           .........GSI.........................SVGEIINNMSKLDGKWFKLSVGTP
Geobacillus_stearothermophilus_ABV66256.1:1-440        .........GSI.........................SVGEIINNMSKLDGKWFKLSVGTP
Geobacillus_sp._B4113_201601_WP_061912600.1:1-440      .........GSL.........................SVGEIINNMSKLDGKWFKLSVGTP
Geobacillus_sp._B4113_201601_5WP_061912600.1:1-44      .........GSL.........................SVGEIINNMSKLDGKWFKLSVGTP
Bacillus_thuringiensis_6WP_029441578.1:1-435           .........GTL.........................SVKEIIGNLSKIDGKWFKLLIGTP
Lysinibacillus_sphaericus_WP_103976887.1:2-422         NGF..................................AVGEIVNTLNRIDGKWFKVIIGKP
Lysinibacillus_sphaericus_WP_069508309.1:2-422         N..................................GF.AVGEIVNTLNRIDGKWFKVIIGKP
Rummeliibacillus_sp._SL167_10_WP_146551437.1:2-42      .........NGS.........................KVTDILDNLRKIDGKWFRVITGKP
Paenibacillus_sp._DXL2_WP_110843609.1:1-459            D.Y.V...ETD...HGRTSAAQFTKHSPT.RK.....KVREVIETLGTIGGRGL.FVAGTP
Paenibacillus_naphthalenovorans_WP_074730028.1:1-      E.Y.V...ETD...HGQTSAAQFTKHSPK....KL..KVREVVKTLGTIGGRGL.FVVGTA
Cohnella_sp._HS21_WP_130606586.1:1-453                 SYY.....ASD...HGQAAVARFTTMSEK.KM.....NVGEVVDEIVKIGGRSA.LLFGSG
Streptosporangium_subroseum_15_WP_089205784.1:1-4      VAR.....GGD...FTERAVRPLVGRGL.........RVGDVLDSVGRIGQGGPFFVAGTP
Arthrobacter_sp._Hz2_WP_120147654.1:1-455              LAR.....EDR...PGRDRQPSTRRHPAG.T......TVGEALE.RITRFDRGPFVAIGTA
Nonomuraea_solani_WP_103963668.1:1-453                 ..D.DIVARGGD...FTERAIRPLAGRGL........RVRDVLDSVGRVGQGGPFFVAGTP
Arthrobacter_ruber_WP_105030338.1:1-455                LAR.....ENR...PGRDRQPSNRRHPAG.T......TVGEALE.RITRFDRGPFVAIGTP
Bacillus_aciditolerans_20_WP_121446947.1:1-456         EYK.....HTE...HGQSRAASLTKDAPK....KL..TVREVLNRFETIDRNH..VLVGNP
WP_137163763.1:1-458                                   E.Y.V...END...AGRAALAAFTAADPN.RR...W.TVREAAE.FVGLGGRGP.VLVGTA
Aeribacillus_pallidus_WP_130157503.1:4-457             E.Y.I...DTN...AIKSVLESLTKASPN...KKW.TVRELAN.FAGIGGMGP.VVVGTP
Bacillus_megaterium_24WP_098602848.1:4-460             K.Y.I...ETN...AVKSMVELLTKSSPN.KK..W.TVRELAT.FAGIGGMGP.VTVGTP
Streptomyces_sp._150FB_WP_078877664.1:5-455            IAR.....EDP...GYQRLTRRYKPGQ..........TVGDIIG.QTGRFDRGPFYVAGTP
Arthrobacter_sp._DWC3_WP_104051071.1:1-454             ..D.DILARENR...PGRDRQPSNRRHPAG.T.....TVGEALE.RITRFDRGPFVAIGTP
Cohnella_thermotolerans_WP_027093898.1:1-456           K.......HTE...HGQSRAASLTKDAPT....KL..TVREVLSKFDNLSRKF..AIVGNP
Paenibacillus_bovis_28WP_144921429.1:1-452             E.Y.V...END...SIRSALENYTKVDPT..R.QW.TVGEIAE.QIGIGGMGI.NMVGTP
Bacillus_aciditolerans_WP_121447760.1:1-452            K.Y.V...KND...AIQSTVENFTKIDSD...KKW.TIDEIKK.YTGIGGLGP.VTVGSP
Leifsonia_sp._ALI-44-B_30_WP_077053645.1:1-455         LAA.....EAK...AGRDNAGANRRHYPP.GA.....TVADALE.AVTRFDRPPFFAVGTP
Parageobacillus_thermoglucosidasius_WP_064552269.      EFK.....NTE...HGHYKAASLTKDASK....RL..SIGEALRKLEQLDRES..IIVGNP
Geobacillus_sp._Y4.1MC1_WP_013399886.1:1-459           E.F.K...NTE...HGHYKAASLTKDASK....KL..SIGEALSKLEQLDRES..IIVGNP
Bacillus_methanolicus_35WP_004434428.1:1-462           EFK.....PTE...HGHYKAASLTKDASK....KL..KIGEALSRLENIDREQ..VIVGNP
Terribacillus_halophilus_WP_077309754.1:1-448          R.F.V...ENE...AIHSAVETFTKIDPN...KKW.TVREVAE.FVGIGGIGP.VVAGTP
Paraburkholderia_kururiensis_WP_017777532.1:1-453      R.Y.V...ESN...AVQSAVEALSSADPT.RV..W.TVREIAK.WGGIGGLGP.LTVGDP
Gracilibacillus_massiliensis_WP_058308419.1:1-456      E.YKK...KTE...GGHYKAATLTKDAPK....KL..TVGDVLNRFEAIDRNS..YIVGNP
Streptomyces_yanglinensis_WP_103888551.1:5-452         IAR.....KVP...GYQRLIRQYRPEQ..........TVGDVLT.RIGGFNRGQFFVAGTP
Trinickia_sp._7GSK02_40_WP_136896361.1:1-452           S.Y.V...ESN...AVQSAVEALSSADPA.RV..W.TVREIAK.WGGIGGLGP.LTVGSP
Cryobacterium_sp._NEAU-85_WP_123045138.1:1-448         EYK.....GRD...HIQSSEAAFANK...........QVRDIID.YYENPTENPFFVSGTP
Alicyclobacillus_acidoterrestris_WP_021298468.1:4      R.Y.V...KNR...AVESAVETFTKIDPD...KTW.NVDEIAD.FVGIGGIGP.VVVGTP
Oceanobacillus_senegalensis_WP_085994229.1:1-452       R.Y.I...RND...AIQSAIETFTKIDPN...KQW.TVDEIAK.FIGVGGIGP.VAVGTP
Bacillus_sinesaloumensis_WP_077620983.1:1-449          G.YKK...KTE...GGHYKAALLTKDAPK....QL..TVGEVLNRFESLDRNS..IIVGNP
Halopenitus_persicus_WP_021074147.1:6-457              E.H.I...ESD...AIQGIVDSFTKADPD...REW.TVGEVAT.FIGVGGIGV.PVVGTP
Microbacteriaceae_bacterium                            E.Y.K...ARD...HIQSSEKAFADK...........RVRDVVD.HYENPLADPFFVWGTP
Lysinibacillus_sinduriensis_WP_036201100.1:1-458       EYQ.....HTE...HGQTRAASLTKDAPK....KL..TVQEVLNKFETIDRDH..VLVGNP
Bacillus_endophyticus_WP_113749053.1:5-457             E.Y.I...ETK...AVQSMLESLTKYSSN...KTW.TVQELAK.FAGIGGMGP.VAVGTP
Pseudomonas_monteilii_WP_119371117.1:1-455             E.H.I...HTN...AIQSAVEAFSTADPN.KR..W.TVQELAD.WVGIGGFGP.LIVGSA
Bacillus_endophyticus_50_WP_061801645.1:5-457          E.Y.I...ETK...AVQSMLESLTKYSSN.KT..W.TVQELAK.FAGIGGMGP.VAVGTP
Planococcus_maitriensis_WP_112233285.1:1-457           E.Y.I...QSN...AMQSAVEVFTRADPD...RKW.TIGELAT.FVGIGGRGP.VAVGTP
Oceanobacillus_profundus_WP_118889880.1:1-447          S.Y.I...END...SIRSALENFTKIHPDRPS.....TVEDVIQ.TIGIGGMGA.TAIGAP
Burkholderia_pseudomallei_WP_141404579.1:1-452         E.H.V...ESN...AIQSAVEALSSADPT.RK..W.TVREIAR.WGGIGGLGP.VSVGDA
Terribacillus_saccharophilus_WP_095261886.1:1-455      T.YGK...TTE...GGHYKAASLTKDASK....QL..TVGEALKRFDTPDK.S..LIVGNP
Sediminibacillus_albus_WP_093212831.1:4-453            R.Y.V...END...AIQSAVENFTKIDPD..K.QW.TVKEIKE.FIGIGGLGA.YTAGSP
Sciscionella_marina_WP_020496929.1:1-448               GFK.....ARG...HVESAERSFADK...........RVREVVD.HFSDPFDDPFFVVGAP
Mycetocola_saprophilus_WP_043321267.1:1-452            EYK.....GRG...HIESAEAEHTRDAKA.N......GATSIIDKLNDPRGDEF.FVSGTV
Lysinibacillus_macroides_WP_053993569.1:4-460          N.Y.I...ETN...AIKSLLEVLTKFSPN...KKW.TVRELAT.FAGIGGMGP.VVVGTP
Pseudomonas_putida_WP_086978777.1:1-458                E.H.I...HTN...AIQSAVEAFSTADPN.KR..W.TVQELAD.WVGIGGFGP.LIVGSA
Sediminibacillus_halophilus_60_WP_074598704.1:4-4      R.Y.V...ENN...AIQSAVENFTRIDPN...KQW.TVKEIKE.FIGIGGLGA.YAVGSP
Rhizobium_oryzae_WP_085420425.1:1-450                  ETG.....RSA...AIVSNVEAMTRTAGP....QW.TKRKLLE.QMVLGSRQA.PMVGSA
Pseudomonas_extremorientalis_WP_071491956.1:1-455      R.H.V...HTN...AIQSAVEAFSTADPN.KV..W.TPNELAD.WVGIGGFGP.LFVGGP
Pseudomonas_fluorescens_WP_016977452.1:1-454           K.H.V...HTN...AIQSAVEAFSTADPN.KV..W.TPNELAD.WVGIGGFGP.LFVGGP
Neorhizobium_sp._T25_27_WP_105419890.1:1-450           ETG.....KSS...AIVSNVEAMTRTAGP....QW.TKRKLLE.QMVLGSRQA.PMVGSA
Pseudomonas_extremaustralis_WP_078833627.1:1-457       K.H.V...HTN...AIQSAVEAFSTADPS.KV..W.TPKELAD.WVGIGGFGP.LFVGSP
Fictibacillus_aquaticus_WP_094253544.1:1-455           EFK.....KTE...HGHYKAASLTKDASK....QL..SIKEALGRLESIDRNT..VFIGNP
Pseudomonas_extremaustralis_KAA8559760.1:1-456         K.H.V...HTN...AIQSAVEAFSTADPN.KV..W.TPNELAD.WVGIGGFGP.LFVGGP
Mycobacterium_sp._ACS1612_WP_067812332.1:1-454         A.V.T...DNE...YLKSSVTMFGQGQ.......W.TLRDFID.AHAIGVEGG.FTVGSP
Bacillus_megaterium_WP_116075167.1:1-455               E.F.I...ATN...SVRSALEKFTISDPD...RKW.TVEEVVK.FVSIGGMGP.VTVGSP
Pseudomonas_frederiksbergensis_70_WP_123409596.1:      K.H.V...HTN...AIQSAVEAFSTADPN.KV..W.TPNELAD.WVGIGGFGP.LFVGSP
Pseudomonas_citronellolis_SFC61163.1:1-452             ........AEL...PGGGPWAALLASDPQ.A......TVGQLVKRLGGFDEGRF.FVAGTP
Herbaspirillum_chlorophenolicum_WP_050468599.1:4-      R.A.A...KTN...SIESLAKTVTNPELN.....W.TKRKLLQ.QLELGGRYA.TVVGSP
Arthrobacter_crystallopoietes_WP_074700854.1:1-45      Q.Y.V...GRD...HAQSMEAEFATGAGR.K......RAHELIDALADPRNDEF.FIAGTP
Terribacillus_goriensis_WP_038558686.1:1-438           K.......HTE...QGQSRAAYLTKDAAK....PF.TVKEIQEKFSQPAEMD..IIIGSP
Bacillus_megaterium_WP_025749753.1:1-455               E.F.I...AKN...SVRSALEKFTISDPD...RKW.TIEEVVK.FVSIGGMGP.VTVGSP
Neorhizobium_sp._T7_12_WP_105403044.1:1-451            E.T.G...KSS...AIVSNVEAMTRTAGP....QW.TKRKLLE.QMVLGSRQA.PMVGSA
Rummeliibacillus_sp._SL167_WP_146553619.1:4-459        K.Y.I...ETN...AIQSMLESLTKASPN...KKW.TPRELAN.FAGIGGLGP.VFVGTP
Burkholderia_sp._PAMC                                  Q.P.P...GAP.KSRGIESSVQRVTDGQAG.....W.TVRRLLD.QLKLGGRYT.TIVGDP
Pseudomonas_koreensis_WP_064585463.1:4-449             Q.Y.V...KSN...AIQSATKNLQNND.......W.TRRKLLD.QHALGGRYI.TVVGSP
Pseudomonas_fluorescens_80_WP_064116961.1:6-451        Q.Y.V...KSN...AIQSATKNLQNND.......W.TRRKLLD.QHALGGRYI.TVVGSP
Pseudomonas_rhodesiae_WP_040265276.1:4-450             Q.Y.V...KSN...AIQSATKTLQNND.......W.TRRKLLD.QHALGGRYI.TLVGSP
Collimonas_sp._OK412_WP_092393355.1:4-454              K.P.A...SSN...AIQSSAKAVTAADAG.....W.TVRKLLD.QLALGGRYV.TIVGSP
Pseudomonas_marginalis_WP_058415544.1:1-456            K.H.V...HTN...AIQSAVEAFSTADPN.KV..W.TPNELAD.WVGIGGFGP.LFVGGP
Pseudomonas_antarctica_WP_064452947.1:1-458            K.H.V...HTN...AIQSAVEAFSTADPN.KV..W.TPNELAD.WVGIGGFGP.LFVGGP
Pseudomonas_protegens_WP_041115159.1:9-454             Q.Y.V...KSN...AIQSATKNLQNND.......W.TRRKLLD.QHALGGRYF.TVVGSP
Pseudomonas_synxantha_WP_057024776.1:4-449             Q.Y.V...KSN...AIQSATKNLQNND.......W.TRRKLLD.QHALGGRYI.TLVGSP
Rhizobium_leguminosarum_WP_128409501.1:2-451           DQS.....STN...ANQSALAAITKQAAK....PV.TLRDIID.QMVLGSRMK.PMVGSP
Pseudomonas_chlororaphis_WP_038359779.1:6-451          Q.Y.V...KSN...AIQSATKNLQNND.......W.TRRKLLD.QHALGGRYI.TVVGSP
Pseudomonas_syringae_90_WP_024642725.1:1-456           R.H.V...HTN...AIQSAVETFSTADPN.TI..W.TPQALAD.WVGIGGFGP.LFVGSP
Collimonas_fungivorans_WP_061540754.1:4-453            K.P.A...SSN...AIQSSAKAVTAADAG.....W.TVRKLLD.QLALGGRYV.TIVGSP
Rhizobium_leguminosarum_WP_116409027.1:2-451           D.Q.S...STN...ANQSALAAITKQAAK....PV.TKREIVD.LMVLGSRMK.PMVGSP
Microvirga_ossetica_WP_099511173.1:1-454               Q.Y.E...KNN...ANNSTLEAITKRSAG.TV..W.TVRKLID.QMILGSRQK.PFVGTP
Pseudomonas_caspiana_WP_087268376.1:4-451              Q.Y.V...KSN...AIQSATKNLKNND.......W.TRQKLLD.QHALGGRYI.TLVGSP
Pseudomonas_lundensis_WP_048375063.1:4-451             Q.Y.V...KSN...AIQSATQVLQNND.......W.TRRKLLE.QHALGGRYM.TVVGSP
Caballeronia_mineralivorans_WP_047894899.1:4-456       E.P.P...GGH.KSRGIESSVQRVTDGQSG.....W.TVRRLLD.QLRLGGRFT.TIVGDP
Pseudomonas_paralactis_WP_057702387.1:4-450            Q.Y.V...KSN...AIQSATKNLQNND.......W.TRRKLLE.QHALGGRYI.TLVGSP
Pseudomonas_taetrolens_WP_048381143.1:4-453            Q.Y.V...KSN...AIQSATNVLQNND.......W.TRRKLLE.QHALGGRYM.TLVGSP
Pseudomonas_psychrophila_WP_123750761.1:4-450          Q.Y.V...KSN...AIQSATKVLQNND.......W.TRRKLLE.QHALGGRYI.TVVGSP
Collimonas_arenae_100_WP_061532973.1:4-454             K.P.A...SSN...AMQSAVKALTAPDAG.....W.TVRKLLD.QLALGGRYV.TIVGSP
Pseudomonas_viridiflava_WP_122210720.1:4-451           Q.Y.V...KNN...AIQSATKNLKNND.......W.TRQKLLD.QHALGGRYI.TVIGSP
Kocuria_varians_WP_068470402.1:1-454                   N.Y.I...KTD...SIQSFLTPFTLQAKD..K.RW.TREEIAK.HCALGGMGA.VIVGSP
Pseudomonas_wadenswilerensis_WP_115084816.1:4-455      Q.Y.V...KSN...AIQSATKNLQNND.......W.TRRKLLE.QHALGGRYI.TLIGSP
Pseudomonas_syringae_WP_011266344.1:4-452              Q.Y.V...KNN...AIQSATKNLKNND.......W.TRQKLLD.QHALGGRYI.TLIGSP
Pseudomonas_sp._286_105WP_122415793.1:4-453            Q.Y.V...KSN...AIQSATKNLKNND.......W.TRQKLLD.QHALGGRYI.TVIGSP
Geobacillus_vulcani_WP_031406843.1:1-461               LFR.....ATD...HGHYKAASLTKDATK....PL.TIGEALQ.KAGTLTRES.VIIGNP
Geobacillus_icigianus_WP_033018365.1:1-461             LFR.....ATD...HGHYKAASLTKDATK....PL.TIGEALQ.KAGTLTRES.VIIGNP
Pseudomonas_floridensis_WP_083182568.1:4-453           Q.Y.V...KNN...AIQSATKNLKNND.......W.TRQKLLD.QHALGGRYI.TVIGSP
Pseudomonas_asturiensis_WP_073167523.1:4-451           Q.Y.V...KSN...AIQSATRNLKNND.......W.TRQKLLD.QHALGGRYI.TVIGSP
Geobacillus_vulcani_110_WP_031406843.1:1-461           LFR.....ATD...HGHYKAASLTKDATK....PL.TIGEALQ.KAGTLTRES.VIIGNP
```

```
Pseudomonas_syringae_WP_024682626.1:4-452            Q.Y.V....KNN....AIQSATKNLKNND.......W.TRQKLLD.QHALGGRYI.TLIGSP
LadAalpha_Geobacillus_thermoleovorans_BAM76377.1     LFR......ATD....HGHYKAASLTKDATK....PL.TIGEALQ.KAGTLTRES.VIIGNP
Tsukamurella_pseudospumae_WP_068746786.1:1-457       G.D.V....KSN....AIISAVSNFQQADPS.GK.DW.TVEDIGK.WGRIGGMGP.VLVGSA
Kocuria_sp._Marseille-P3598_WP_085529073.1:5-456     N.Y.V....KTD....SIQSFLTPFTLQSKD..K.EW.TREEIAK.HCAIGGMGD.VIVGGP
Microvirga_aerophila_WP_114188789.1:1-456            QYQ......KTD....AMASMLEAFTRDSSQ....VW.TLRKIFD..KGLGARNP.PLVGSA
Burkholderia_ubonensis_WP_071760483.1:5-455          S.Y.V....KTD....SIQSAVDAISKKSTS.GA..W.TVRRMLD.QMSLGGRYQ.PIVGSP
Achromobacter_marplatensis_WP_006226823.1:4-450      R.Y.V....KNN....AIESAVKNLTVSRTD.....R.TVTDLLA.DMALGGRYP.ALVGSA
Acinetobacter_baumannii_WP_070165746.1:1-443         E.Y.I....QTN....AIQSLLDSYVNADPE.RV..W.TIEEIAN.WNSLGGNGP.VLVGSA
Labedella_phragmitis_WP_128496094.1:1-453            G.N.V....ESN....AIQSAVANFQEANDD.GG.EF.RVRDIAR.MGAIGGLGP.RIVGSP
Nocardia_cyriacigeorgica_120_WP_130918184.1:1-453    G.D.V....ESN....AIQSAVAAFQEFDDD.GR.EW.TVRDIGR.WAGIGGMGP.VFVGSG
Nocardia_cyriacigeorgica_WP_130918184.1:1-453        G.D.V....ESN....AIQSAVAAFQEFDDD.GR.EW.TVRDIGR.WAGIGGMGP.VFVGSG
Mycobacterium_sp._1164985.4_WP_067298521.1:1-453     G.N.V....DSN....AILSAVAAFQSADPD.GR.EW.AIRDIAE.WGKIGGMGP.RFVGSG
Rhodococcus_sp._1R11_WP_135042739.1:1-449            G.N.V....ESN....AIQSFVASFQSIGAD.GK.EW.TVRDIAE.WGGIGGAGP.RIVGSP
Erwinia_amylovora_WP_004157587.1:5-459               K.H.L....HTN....AIQSAVETFSTADPD.RQ..W.TVQALAD.WVGIGGFGP.LLVGSA
Ralstonia_mannitolilytica_WP_102079134.1:4-451       D.Y.G....GGN....AIESATT..TAARHG.....W.TRRKLLE.LFELGGRYP.AIVGDP
Leifsonia_flava_WP_135120808.1:15-465                G.N.V....QSN....AIRSVIANYEESAKN.GE.VF.TVRDIAR.GGAIGGLGP.TIVGSG
Cryobacterium_roopkundense_WP_084141147.1:1-453      G.N.V....KSN....AIQSAVSNVQGAGGE.GK.EW.TVRDIAR.QGAIGGLGP.IVVGSG
Cupriavidus_pauculus_WP_101681197.1:1-455            QTG......KSQ....AIVSNVEAMTRSAGP....QW.TRRKLLE.QMVLGSRQP.PWVGSA
Rhizobium_sp._BK333_130_WP_133704837.1:2-454         E.Q.K....TTN....AQQAALAAVTGKSSG.KP.AM.TPRQIID.QMVLGSRQK.PMVGSP
Caballeronia_mineralivorans_WP_047846789.1:1-454     R.K.V....ETN....AIVSAIEHLAGGA...KS..W.TIDELAA.WGGIGGMGP.VFVGSA
Nocardia_altamirensis_WP_069163049.1:1-454           G.Q.V....ESN....AIQSAVAAFQEFDDDGGR.EW.TVRDIGQ.WAGIGGMGP.VFVGSG
Cupriavidus_sp._P-10_WP_116322758.1:1-452            K.H.I....RND....SMHTAIDRFTIADPN...RVW.TVGEVAE.HLAVGGSGP.VIVGSV
Serratia_sp._S1B_WP_116726564.1:1-452                E.Y.I....QTN....AIQSLLDSYVNADPN.RV..W.TIEEIAN.WNSVGGNGP.VLVGSP
Pseudomonas_putida_WP_043864435.1:1-452              R.E.V....QSD....AIQSAVEAFTRADPS.RT..W.TTAEIAD.YCAIGGDGP.VLVGSA
Pseudomonas_aeruginosa_WP_023084248.1:4-453          R.Y.E....KNN....AIESVVKNLTVARTD.....S.TVRKLLD.QLALGGRYV.TLVGDP
Pseudomonas_mucidolens_WP_084381795.1:9-455          Q.Y.V....KSN....AIQSATKTLQEND.......W.TRRKLLE.QHALGGRYI.TLVGSP
Pseudomonas_poae_WP_060549803.1:4-450                Q.Y.V....KSN....AIQSASKTLQNND.......W.TRRKLLE.QHALGGRYI.TLVGSP
Pseudomonas_chlororaphis_WP_123573412.1:9-454        Q.Y.V....KSN....AIQSATKNLQNND.......W.TRRKLLD.QHALGGRYI.TVVGSP
Janthinobacterium_lividum_140_WP_072454679.1:1-44    D.Y.I....DSP....AGRSALASFSQADPD.RT..W.TVREAAE.YIGLGGRGP.VLVGDA
Haladaptatus_cibarius_WP_049970513.1:4-446           E.H.I....ETE....AIQGAVNALTKHAPE...RDW.TVRDVAE.FCGLGSTSP.VVVGSP
Arthrobacter_luteolus_WP_066298473.1:1-443           K.Y.L....DTN....AARSALSIFTTADPG..R.SW.TPNAIAD.YLGIGGIGP.VLVGSA
Geobacillus_sp._ZGt-1_WP_047758532.1:1-436           LFR......ATD....HGHYKAASLTKDATK....PL.TIGEALQ.KAGTLTRES.VIIGNP
Lysinibacillus_composti_WP_124766485.1:4-458         S.Y.I....END....SIRSTLENFTKINLDRPS....TVQDVID.AVGIGGMGA.AAIGSP
Microcella_putealis_WP_130486201.1:20-474            G.N.V....PSN....AIQSVVSNFAAAAEH.GQ.EF.TVGDIGR.HSAIGGLGP.LIVGSG
Pseudomonas_nitroreducens_WP_037016976.1:1-453       R.H.V....QSD....AIQSAVDAFSAADPQ.RQ..W.TAAEIAD.YCALGGDGP.LLVGSP
Acinetobacter_wuhouensis_WP_130168784.1:1-453        E.Y.I....KTN....AIQSMLDAYVNADPN.RV..W.TIEEIAH.WNSVGGNGP.VLVGSA
Bradyrhizobium_sp._YR681_WP_008144663.1:1-454        R.H.V....QND....AGRSALDNVTRGDPD.RV..W.TVRDVIE.HVGIGGAGP.VVVGTP
Schumannella_sp._10F1D-1_WP_141164655.1:10-460       G.N.V....KSN....AIQSVLTNLQQEKDL.GR.EW.TVGDLAR.QGAIGGLGP.FIVGSG
Pseudomonas_coronafaciens_150_WP_122355187.1:4-45    Q.Y.V....KNN....AIQSATKNLQNND.......W.TRQKLLD.QHALGGRYI.TLIGSP
Pseudomonas_syringae_WP_003430238.1:4-452            Q.Y.V....KNN....AIQSATKNLKDND.......W.TRQKLLD.QHALGGRYI.TLIGSP
unclassified_Rhodococcus_WP_094682261.1:1-449        G.N.V....ESN....AIQSFVASFQSIGAD.GK.EW.TVRDIAE.WGGIGGAGP.RIVGSP
Hafnia_alvei_WP_046449330.1:6-449                    VAG......KVE....AIESVTKAFSG.........W.TKRRLLE.QHALGARYP.VIVGSP
AS9A_3890_Hoyosella_subflava_WP_013808677.1:1-453    G.N.V....ESN....AIRSAVAAFQESGDD.GR.EW.LVRDIAE.WASIGGLGP.RFVGSG
Acinetobacter_baumannii_WP_000995600.1:1-443         E.Y.I....QTN....AIQSLLDSYVNADPE.RV..W.TIEEIAN.WNSLGGNGP.VLVGSA
Agrobacterium_sp._B133/95_WP_065696417.1:2-454       E.Q.K....TTN....AQQAALAVVTGKASG.KP.AM.TPREIID.QMVLGSRQK.PMVGSP
Microbacterium_sp._KROCY2_WP_024289149.1:1-453       N.Y.V....KTE....AIQSFLTPFTMQDGA..K.QW.TREEIAR.HCAIGGMGD.VVVGSP
Variovorax_paradoxus_WP_012747040.1:6-452            D.Y.G....KTN....AIESATR..TAEQQG.....W.THRKLLD.LFELGGRYP.AIVGDA
Methylobacterium_radiotolerans_WP_076730229.1:5-4    P.Y.A....PGN....AIQSATAAAARRGL........TKRDLLA.ELQLGSRYA.VLTGDA
Streptomyces_sp._BK215_WP_132915265.1:7-452          ..HEL....RTE....VAHGAVRGLIESAPDKTM....TFADLARLMVTT......RAVGTP
Geobacillus_thermodenitrificans_160_WP_008880416.    LFH......TTD....HGHYKAASLTKDAAR....PM.TIGAALE.KAGTLTRDA.VIIGNP
Pseudomonas_aeruginosa_WP_132556951.1:356-785        R.Y.E....KNN....AIESVVKNLTVARTD.....S.TVRKLLD.QLALGGRYV.TLVGDP
Penicillium_solitum_OQE02104.1:14-471                R.H.V....ESN....AIRSAVEGWSKATPEVAK..W.TKATVGR.HITVGGLGA.TPVGTA
Penicillium_vulpinum_OQE05502.1:12-469               R.H.V....ESN....AIRSAVEGWSKATPDVAK..W.TKATVGQ.HITVGGLGA.TPVGTA
Clavibacter_michiganensis_WP_045526194.1:1-454       K.Y.I....DTD....AARSALAIFTRYDPD..R.DW.TPRDVAH.YVGIGGIGP.VISGSA
Glutamicibacter_creatinolyticus_WP_054821484.1:6-    GSN.V....KSN....AIQSSVETFQKASGD.EGSPW.TIRQLAE.WVGVGGFGP.ITVGSG
Campylobacter_sp._P0078_WP_086237522.1:4-454         S.N.I....KSN....AMLGKVEEYANSKTDNGK.QW.SLRELIK.VAGIGALGQ.KIVGGA
Pseudomonas_furukawaii_WP_003454569.1:1-447          VER......KTN....AIESVVKGFSG.........W.TKRRLLE.QHALGGRYP.QAVGSP
Leucobacter_triazinivorans_WP_130111548.1:1-442      G.N.V....ESN....AIQSTVAAFQADNPD.GR.AW.TVRDMVE.RNGIAGLGA.TVVGSA
Aspergillus_brasiliensis_CBS_101740_OJJ68286.1:12    R.H.V....ESN....AIRSAVEGWSKATPEVEK..W.TKATVGQ.HITVGGLGA.TPVGTP
Aspergillus_udagawae_170_GAO89440.1:15-472           R.H.V....ESN....AIRSAVEGWSKSTPEVAK..W.TKSTVGQ.HITVGGLGA.TPVGTP
Aspergillus_flavus_RMZ37351.1:15-472                 R.H.V....ESN....AIRSAVEGWSKATPGVDK..W.TKSTVGQ.HITVGGLGA.TPVGTP
Aspergillus_flavus_RAQ54081.1:15-472                 R.H.V....ESN....AIRSAVEGWSKATPGVDK..W.TKSTVGQ.HITVGGLGA.TPVGTP
Penicillium_arizonense_XP_022490779.1:12-469         R.H.V....ESN....AIRSAVEGWSKATPEVAK..W.TKATVGQ.HITVGGLGA.TPVGTA
Salinibacterium_sp._CGMCC                            G.N.V....KSN....AIQSAVSNFQQANAE.GG.EW.TVGDIAR.HSAIGGLGP.TIVGSG
Aspergillus_fumigatus_OXN06785.1:14-471              R.H.V....ESN....AIRSAVEGWSKSTPEVAK..W.TKSTVGQ.HITVGGLGA.TPVGTP
Aspergillus_lentulus_GAQ10210.1:7-464                R.H.V....ESN....AIRSAVEGWSKSTPEVAK..W.TKSTVGQ.HITVGGLGA.TPVGTP
Aspergillus_flavus_RAQ66103.1:15-472                 R.H.V....ESN....AIRSAVEGWSKATPGVDK..W.TKSTVGQ.HITVGGLGA.TPVGTP
Aspergillus_fumigatus_OXN22894.1:14-471              R.H.V....ESN....AIRSAVEGWSKSTPEVAK..W.TKSTVGQ.HITVGGLGA.TPVGTP
Aspergillus_nidulans                                 R.S.V....PSN....AIRSAVEGWSKATPEVP..KW.TKKTVGE.HITVGGLGS.TPVGTP
Penicillium_nalgiovense_OQE79848.1:14-471            R.H.V....ESN....AIRSAVEGWSKATPEVAK..W.TKATVGQ.HITVGGLGA.TPVGTA
Aspergillus_wentii                                   RFS......ESP....RVQSIVRRWSATVPGTENLQW.TKRRIVE.FLSVGGLGA.KIVGSP
Gordonia_iterans_WP_105943624.1:1-453                G.D.V....ESN....AIRSAVSAFAQASES.GK.PW.SVGDLAE.WGGIGGMGP.VFIGSG
Azotobacter_beijerinckii_WP_090735436.1:4-454        R.Y.Q....KTN....AIESVARTFTAAGSG.....W.TRRKLLE.QHALGGRYP.TIVGSP
Staphylococcus_sp._NAM3COL9_WP_057513692.1:4-453     EDI......ETE....AMQGNLNMYTKDPHH....KW.TLREAVK.NHGLGNGTA.KFIGTK
Acidovorax_sp._56_WP_099656603.1:11-450              A.Y.G....QTN....SIQSAVA..LAKRQG.....W.TRRGMLE.QYALGGRYP.AIVGTG
Paenibacillus_sp._BIHB4019_WP_099518466.1:1-456      ..ENI....HTE....MGQTHTDRFTKHSKT.KK....TVQEVMDDFVQKGFRGL.TVVGTP
Penicillium_antarcticum_OQD90033.1:5-465             R.F.V....ELP....AVRSMVNHWASTVPGTEGQKW.DKKTIAE.YLTLGGNGV.KIIGSV
Aspergillus_oryzae_RIB40_190_XP_001823703.1:9-463    RFS......ESP....RVQSIVRRWSATVPGTENLPW.TKRRIVE.YLSVGGLGA.KVVGSP
Fusarium_oxysporum_f._sp._pisi                       R.Y.V....ESN....AIRSAVESWSKSIPGVPK..W.TKHTVAN.HIKVGGLGA.TVAGTP
Colletotrichum_fructicola_Nara_gc5_ELA35345.1:18-    LMT......DSP....RIRSIVRRWSDTVPGTDNLPW.TKRRIVE.YLSIGGMGA.KIVGSP
Micrococcus_luteus_WP_041104460.1:19-468             G.D.V....KSN....AIQSTVETFQKASGTD.E.EW.TVRKLAE.WVGIGGFGP.VIVGGG
Aspergillus_awamori_GCB21923.1:12-469                R.H.V....ESN....AIRSAVEGWSKATPEVEK..W.TKATVGQ.HITVGGLGA.TPVGTP
Aspergillus_niger_CBS                                R.H.V....ESN....AIRSAVEGWSKATPEVEK..W.TKATVGQ.HITVGGLGA.TPVGTP
Aspergillus_lacticoffeatus_CBS_101883_XP_02545389    R.H.V....ESN....AIRSAVEGWSKATPEVEK..W.TKATVGQ.HITVGGLGA.TPVGTP
Aspergillus_wentii_DTO_134E9_OJJ40237.1:14-471       R.H.V....ESN....AIRSAVEGWSKATPEVAK..W.TKSTVGQ.HITVGGLGA.TPVGTP
Aspergillus_indologenus_CBS_114.80_PYI31207.1:11-    R.G.V....QSN....AIRSAVEGWSKATPEVA..KW.TKSTVGR.HITVGGLGA.TPVGTP
Aspergillus_fischeri_NRRL_181_XP_001264002.1:14-4    R.H.V....ESN....AIRSAVEGWSKSTPEVAK..W.TKSTVGQ.HITVGGLGA.TPVGTP
Penicillium_coprophilum_200_OQE46388.1:11-466        RFS......DSP....RVQSIVRRWSATVPGTDSLPW.TKRRIVE.YISVGGLQA.KVVGSP
Cupriavidus_taiwanensis_WP_116379876.1:8-454         N.Y.E....GGN....AIESATK..TAAQHG.....W.TRRKLLD.LFELGGRYP.AIVGDP
Penicillium_digitatum                                R.F.V....ELP....AVRSMVNHWASTVPGTEGQKW.DKKTIAE.YLVLGGNGV.KIIGSV
Aspergillus_niger_GAQ46262.1:12-469                  R.H.V....ESN....AIRSAVEGWSKATPEVEK..W.TKATVGQ.HITVGGLGA.TPVGTP
Aspergillus_nomius_NRRL_13137_XP_015412385.1:15-4    R.H.V....ESN....AIRSAVEGWSKATPGVDK..W.TKSTVGQ.HITVGGLGA.TPVGTP
Aspergillus_aculeatus_ATCC_16872_XP_020055010.1:1    R.G.V....QSN....AIRSAVEGWSKATPEVAK..W.TKATVGR.HITVGGLGA.TPVGTP
Penicillium_brasilianum_OOQ84969.1:19-474            R.F.V....ESP....RVQSIVRRFSATVPGTDNLPW.TKKRIVE.YISVGGLQA.KVIGSP
Penicillium_subrubescens_OKP15100.1:19-474           R.F.A....DSP....RVQSIVRRWSATVPGTDSLPW.TKKRIVE.YISVGGLQA.KVIGSP
Trichoderma_arundinaceum_RFU80530.1:19-474           RFT......DSP....RLQSIVRRWSDTVPGTDNLPW.TKRRIVE.YLSLGGLGA.KAVGSP
Aspergillus_thermomutatus_210_XP_026614594.1:15-4    R.Q.V....ESN....AIRSAVEGWSKSTPEVA..KW.TKSTVGQ.HITVGGLGA.TPVGTP
Serratia_liquefaciens_WP_044551273.1:4-449           Q.T.G....PTQ....AIQSVTQTY....RG.....W.TRRQLLE.QHAMGGRYP.LVVGDP
Micrococcus_luteus_WP_065572847.1:19-468             G.D.V....KSN....AIQSAVETFQKASGTDE..EW.TVRKLAE.WVGIGGFGP.VIVGGG
Halolamina_rubra_WP_049979911.1:1-449                E.H.I....ETQ....AIQGTMNAFTQADPD...REW.TVREVAE.FSGLGSTSP.IVVGTP
Acinetobacter_pittii_WP_075382847.1:7-452            P.Y.Q....KSN....SIVSITEKFKEQQ.......I.TKNDLKA.QHVLGGRYP.LIVGSG
Corynebacterium_variabile_WP_052302534.1:2-464       V.G.I....ESN....AIQSAVAAFQRSAGEEGR.EW.TVREVAE.FGGIGGLGP.VVVGSG
Aspergillus_luchuensis_GAT21254.1:12-471             R.H.V....ESN....AIRSAVEGWSKATPEVEK..W.TKATVGQ.HITVGGLGA.TPVGTP
```

```
Aspergillus_luchuensis                            R.H.V....ESN....AIRSAVEGWSKATPEVEK..W.TKATVGQ.HITVGGLGA.TPVGTP
Aspergillus_bombycis_XP_022386026.1:15-472        R.H.V....ESN....AIRSAVEGWSKATPGVDK..W.TKSTVGQ.HITVGGLGA.TPVGTP
Agrobacterium_vitis_WP_070163524.1:1-454          Q.K.V....ETN....AIHSFVEHIAGGD...KS..W.TIEELAK.FGGIGGLGP.VFVGAP
Pseudomonas_psychrotolerans_220_WP_074584491.1:5- RPQ......RTE....AIESALKRLVAKDAG.....W.TRRKLLE.QHALGGRYT.ALVGDP
Micrococcus_yunnanensis_WP_135040276.1:19-468     G.D.V....KSN....AIQSAVETFQKASGT.DE.EW.TVRKLAE.WVGIGGFGP.VIVGGG
Acinetobacter_calcoaceticus_WP_004642549.1:7-452  P.Y.Q....KSN....SIVSITEKFKEQQ.......I.TKNDLKA.QHVLGGRYP.LIVGSG
Penicillium_brasilianum_CEO59775.1:15-472         R.H.V....ESN....AIRSAVEGWSKATPEVAK..W.TKTTVGQ.HITVGGLGA.TPVGTA
Aspergillus_cristatus_ODM22551.1:12-469           R.T.V....ESN....AIRSAVEGWSKATPEVP..KW.TKKTVGE.HITVGGLGA.TAVGTP
Pseudomonas_taiwanensis_WP_027907363.1:1-446      E.Y.V....END....AGRAALAAFTAADPN.RR..W.TVREAAE.FIGLGGRGP.VLIGSP
Acidovorax_avenae                                 G.Q.H....RSN....GIENSLRRLANGRAH.....Y.TLGDLLE.ELALGGRYA.TVVGSP
Staphylococcus_pseudintermedius_WP_101431388.1:4- E.N.I....ETE....AMQGNLNLYTKDPHR....KW.TLREAVK.NHGLGNGTV.KFIGTP
Rhodococcus_triatomae_WP_081607437.1:2-451        E.Y.V....ETD....GIRSFLERFTIADPD.RT..W.TPRQVAE..QMARSLGAITVVGSP
Alcaligenes_sp._RS4_230_WP_128393375.1:4-450      S.Y.G....QSN....AMQSASQTA..SQQG.....W.TRRDLLN.QHKLGSRYP.TLVGDA
Gibbsiella_quercinecans_WP_121552947.1:7-454      A.G.G....PTR....AIESVSNTYS.........GW.TKRRLLE.QHALGGRYP.LIVGDP
Acinetobacter_ursingii_WP_044435211.1:5-452       P.Y.Q....KSN....SIASVNEKFKTQQ.......I.TKNDLKN.QHVLGGRYP.LIVGSG
Erwinia_typographi_WP_034887712.1:4-457           V.S.G....DTR....AIESVSKAF....SS.....W.TRRRLLE.QHAMGSRYP.LLVGNP
Trichoderma_harzianum_KKP01913.1:14-470           R.H.V....ESN....AIRSAVEAWSKASPSVAK..W.TKRAVAK.HIQVGGLGA.TVIGTP
Fusarium_graminearum_235_CEF79766.1:12-466        R.Q.V....ESN....AVKSTVEGYARFSPVNS..KW.TKHTVAE.HVSLGGNGP.LLVGTP
Gordonia_alkanivorans_WP_006357654.1:10-461       G.N.V....ESN....AIQSAVAAFQQASES.GE.EW.RVRDIAA.WGGIGGMGP.VLIGSG
Acinetobacter_radioresistens_WP_111281495.1:5-455 P.Y.Q....QTN....SIASVNNKFKEQK.......I.TVADLKD.QHVLGGRYP.LIVGSG
Mesorhizobium_plurifarium_WP_073985566.1:5-458    E.Y.V....ETQ....GIRSVLDIYTRLDPN...RRW.TPRAIGE.FLGIAGGGA.EIVGGP
Oceanobacillus_halophilus_WP_121203049.1:1-454    EDM......ETE....AVQGNLDMYTKDPNK....KW.TLREAVQ.NHGLGNGTV.KFIGTP
Acinetobacter_tandoii_240_WP_100241535.1:7-457    P.Y.Q....KTN....SIASVNQKFKEQS.......I.TRNDLKA.QHVLGGRYP.LIVGSG
Burkholderia_plantarii_WP_042624083.1:1-457       A.Y.V....DTD....AGQSALASFSKLDPD.KV..W.TVRDATE.FAAIGGRGA.VVVGDA
Alcaligenes_faecalis_WP_094195208.1:6-457         S.F.G....PSN....AIQSAAAAA..QKQG.....WTTRRKLLE.QFTLGSRYK.TIVGDP
Bradyrhizobium_arachidis_WP_092214516.1:1-453     P.T.VK...KDN....AVTSMIDSFSRSE...RP..W.TIREIIE.HNGIGGRGP.VIVGSP
Cupriavidus_plantarum_WP_109583068.1:1-458        A.Y.V....DTN....AGQSALASFSKLDPS...RTW.TVRDAVE.FVGIGGRGG.VAVGDP
Variovorax_sp._770b2_245_WP_093443018.1:5-461     S.G.G....SAD....AIQSSVSSL.AGQGG.KP.SP.TVRQLLA.QMPLGGRHT.PVVGSP
RZJ22128.1:4-447                                  P.K.LE...DADINFSSRQQMMIDIARKHNF.......SIRQLYEYIASARGHW..TLIGTA
LadB_Geobacillus_thermoleovorans_BAM76371.1       .........GAK....RLHELMKTSAERRFQIGPNLWAGLTQVLS......GNSI.ALVGTP
LadAbeta_Geobacillus_thermoleovorans_BAM76372.1   PDD.IPETNGNQ....SRRQLIIDLARRERL.......TIRQLYQ..RIAGARGHFTIIGTP
Af1_AFLA_024140_XP_002373925.1                    R.H.V....ESN....AIRSAVEGWSKATPGVDK..W.TKSTVGQ.HITVGGLGA.TPVGTS
Af3_AFLA_58870_XP_002378631.1                     R.F.V....EQP....AIRSMVNHWASTVPGTEGKKW.DKKTISE.YLIMGGNGA.KVIGSA
Af4_AFLA_049780_XP_002383105.1                    DSL......EAH....KVTSMLDAFTTTSEDVPQ..W.TPRVVAQ.KAAIGGLGP.VGIGSP
Af5_AFLA_126060_XP_002381799.1                    SFT......AVG....GIQSLISSWSKTVPNSNGLKW.TKRRVLQ.ELALGGAHP.RAIGSP
Af2_QMW32876.1:9-463                              R.F.S....ESP....RVQSIVRRWSATVPGTENLPW.TKRRIVE.YLSVGGLGA.KVVGSP
Yarrowia_lipolytica_CLIB122_YALI0C07546p_XP_50156 R.E.T....DNP....NLVKAVSMWASQTDSKDE.VW.DVDKVAN.EYKLAGRGG.VAVGDA
Rhizobium_sp._CF122_WP007794079.1                 P.D.VP...ETN.ASKSRQKLLLDLARRENL.......TIRELYLRVAGARGHW..QIVGTP
Acinetobacter_sp._NIPH_2100_LLM                   P........ELG....EGQGMVSRPALIADIAKKNNF.SIRQLYE.WVA.GARGHWQLIGTP
Nostoc_sp._PCC_7107_WP_015115963.1                P.E.LP..SDTN.NNKSRLKLVKDLATRGTL.......TLRQLYLALATARGHR..TIIGTP
Aspergillus_flavus_NRRL3357_uncharacterized       PTD.IPETNASK....TGRAGVLKLAEDEKL.......TVRQLAQRHGGYSGL...AFVGTP
Paenibacillus_curdlanolyticus_WP006037998.1       P.E.LPELSQIN.GGKSRFQLLKDLADREQL.......TIRQLYQ..RIAGARGHREIVGTP
LadAbeta_Geobacillus_thermoleovorans_FMN-dependen PDD.IPETNGNQ....SRRQLIIDLARRERL.......TIRQLYQ..RIAGARGHFTIIGTP
SsuD_1M41                                         GQQ......RMA....ALHNGKRDNLEISPN....LW.AGVGLVR.....GGAGT.ALVGDG
Paenibacillus_curdlanolyticus_WP006037998.1       P........ELS....QINGGKSRFQLLKDLADREQL.TIRQLYQ..RIAGARGHREIVGTP
Bacillus                                          ..............................................................
Pseudomonas                                       .........RMA....ALHGGRRDRLEIQPN....LW.AGVGLVR.....GGAGT.ALVGDP
LadB_Geobacillus_thermoleovorans_FMN-dependent    .........GAK....RLHELMKTSAERRFQIGPNLWAGLTQVLS......GNSI.ALVGTP
Pseudomonas_aeruginosa_PAO1_NP_251290.1           .........GSQ....RLLAAAEQGEVLDSR....LWTGVARLTG.....ARWNSTALVGTP
Alkanesulfonate                                   ..............................................................
Alkanesulfonate                                   .........QNV....GSQRLLDIAARGEVQ.DRALW.YPTVVAT...NARGAST.ALVGSY
Aspergillus_niger_ATCC13496_RDH19503.1            ................GSQRLLDIAARGEIQDRALWYPTVT.....ATNARGAST.ALVGSP
Penicillium_digitatum_Pd1_XP014534980.1           P........PQN....VGSRRLLDIATRGEVQDRALWYPTVTATN....AQGAST.ALVGSP
Acinetobacter_rudis_WP_016657872.1                K........RMA....ALHGGKRDHLEVSPN....LW.AGVGLVR.....GGAGT.ALVGDP
```

```
                                                      360       370        380       390       400       410
LADA_Geobacillus_thermodenitrificans_WP_011888513   KKVADEMQYLVEEAGIDGFNL..VQYVSPGTFVDFIELVVPELQKRGLYR..VDYEEG..TY
Geobacillus_sp._LEMMY01_WP_079935807.1:1-440        KKVADEMQYLVEEAGIDGFNL..VQYVSPGTFVDFIELVVPELQKRGLYR..VDYEEG..TY
Geobacillus_stearothermophilus_ABV66256.1:1-440     KKVADEMQYLVEEAGIDGFNL..VQYVSPGTFVDFIELVVPELQKRGLYR..VDYEEG..TY
Geobacillus_sp._B4113_201601_WP_061912600.1:1-440   KKVADEMQYLVEEAGIDGFNL..VQYISPGTFVDFIELVVPELQKRGLYR..VDYDEG..TY
Geobacillus_sp._B4113_201601_5WP_061912600.1:1-44   KKVADEMQYLVEEAGIDGFNL..VQYISPGTFVDFIELVVPELQKRGLYR..VDYDEG..TY
Bacillus_thuringiensis_6WP_029441578.1:1-435        KEIANEMQYLVEETDIDGFNL..VQYISPGTFQDFIDLVVPELQKRGLYK..TQYTEG..TY
Lysinibacillus_sphaericus_WP_103976887.1:2-422      KEVADQIQHIIEQTNIDGFNL..VQYHSPDTFEDFIKYIVPELQNRGLYQ..KDYSPG..TY
Lysinibacillus_sphaericus_WP_069508309.1:2-422      KEVADQIQHIIEQTNIDGFNL..VQYHSPDSFEDFIKYIVPELQNRGLYQ..KEYSPG..TY
Rummeliibacillus_sp._SL167_10_WP_146551437.1:2-42   VEVVNQIQSIIEETDIDGFNL..VQFHSPHTFEDFINYIVPELQNRELYQ..KDYSPG..TY
Paenibacillus_sp._DXL2_WP_110843609.1:1-459         EEVADKLQYWVEEAGIDGFNV..TQFVSPGTLRDFVELVVPVLQRRGLFR..EEYEET..TL
Paenibacillus_naphthalenovorans_WP_074730028.1:1-   EEVADRLQYWVEEAGIDGFNV..SQFLSPGTLQDFVELVVPELQKRKLFR..ENYEET..TL
Cohnella_sp._HS21_WP_130606586.1:1-453              EEVADQMQQWVERTGIDGFNL..AHLITPGSLEDIIDLVVPILQERGLYK..TEYAKG..TM
Streptosporangium_subroseum_15_WP_089205784.1:1-4   ERVADEIERWADETGAAGFNL..MQYLSPGTAEDFIELVVPELQKRGRYR..TSYDDT..TL
Arthrobacter_sp._Hz2_WP_120147654.1:1-455           TEVADEIERWVEGTDLDGFNL..RQFLTPGTAEDFIELVVPELQKRGLYR..RSYEES..TL
Nonomuraea_solani_WP_103963668.1:1-453              ERVADEIERWADETGATGFNL..MQYLSPGTAEDFIELVVPELQRRGRYR..TSYEDS..TL
Arthrobacter_ruber_WP_105030338.1:1-455             TEVADEIERWVEATGLDGFNL..RQFLTPGTAEDFIELVVPELQKRGRYR..RSYEES..TL
Bacillus_aciditolerans_20_WP_121446947.1:1-456      SEVADAIQFQFEESGVDGFNL..AHLVTPESLEEFIDLVIPELQKRGIYK..TEYEQG..SF
WP_137163763.1:1-458                                SEVADQLESWLDQTGIDGFNL..TYAVQPDDLANVVELLVPELQRRGRYP..LSHAEG..TL
Aeribacillus_pallidus_WP_130157503.1:4-457          KQIADTFEEWIEDTDIDGFNL..AYAITPGTFKDFVDLVIPELQQRGVVK..KEYEEG..TL
Bacillus_megaterium_24WP_098602848.1:4-460         EKIADTLEEWIDETDIDGFNL..AYAITPGTFKDFVELVSPELQKRGLVK..KEYEEG..TF
Streptomyces_sp._150FB_WP_078877664.1:5-455         GVVADAIENWLDEGGIDGINL..RQFLTPGTARDFIELVVPELRRRGRFR..ESYEEGE.TL
Arthrobacter_sp._DWC3_WP_104051071.1:1-454          MEVADEIERWVEGTDLDGFNL..RQFLTPGTAEDFVELVVPELQRRGLYR..RSYEES..TL
Cohnella_thermotolerans_WP_027093898.1:1-456        QEVADGIQSYFEATGVDGFNL..AHFVTPGSLVDFVDLVVPELQRRGVYK..TEYAEG..TY
Paenibacillus_bovis_28WP_144921429.1:1-452          EQIADLMEEWVNEAGVDGFNI..AYAVSPGTFKDFAELVVPVLQARGRVP..IAYEET..TL
Bacillus_aciditolerans_WP_121447760.1:1-452         EQVANDLESWVEETGVDGFNI..AYAITPGTFKDFIELVVPLLQERGLVR..KEYEGD..TL
Leifsonia_sp._ALI-44-B_30_WP_077053645.1:1-455      VEVADAIERWVDETGLDGFNL..RQFLTPGTARDFIEYVVPELQKRGRYR..TSYEAP..TL
Parageobacillus_thermoglucosidasius_WP_064552269.   EEVADAIQYRFEASGVDGFNL..NHLVTPSSLEDFIELVIPILQKRGLYK..TEYKQG..TL
Geobacillus_sp._Y4.1MC1_WP_013399886.1:1-459        EEVADAIQHRFEESGVDGFNL..NHLITPSSLEDFIELVIPILQKRGLYK..TEYKPG..TL
Bacillus_methanolicus_35WP_004434428.1:1-462        IEVADAIQYQFEASGVDGFNL..NHLITPGSLEDFIDLVVPILQERGLYK..TEYKEG..TL
Terribacillus_halophilus_WP_077309754.1:1-448       EQVADAMEDWVEQTGVDGFNI..AYAVTPGTFKDLADYVVPILQKRGRIR..EKYEGK..TL
Paraburkholderia_kururiensis_WP_017777532.1:1-453   AQVADELQSWVEETDVDGFNL..AYALTHETFSDFVELVVPELQRRGAYK..TEYARG..TL
Gracilibacillus_massiliensis_WP_058308419.1:1-456   KEVADAIQFQFEESGVDGFNL..NHLVTPGDLESFVDLVIPELQARGIYK..KDYREG..TL
Streptomyces_yanglinensis_WP_103888551.1:5-452      KVVADTIENWLDEGGIDGINL..RQFLTPGTAQDFIELVVPELRRRGRYR..ESYEDGE.TL
Trinickia_sp._7GSK02_40_WP_136896361.1:1-452        EQVADELQSWVEETGVDGFNL..AYALTHETFSDFVDLVVPELQRRGVYK..TEYAPG..TL
Cryobacterium_sp._NEAU-85_WP_123045138.1:1-448      EQVADRIEEIAEAADLDGFNL..VQYLSPGTFEDFIDLVVPELQKRGRYR..TAYRDGE.TL
Alicyclobacillus_acidoterrestris_WP_021298468.1:4   DTIADAMEYWMDEAGVDGFNI..AYTVSPGTFVDFVERVVPVLQRRGLVR..HAYGGT..TL
Oceanobacillus_senegalensis_WP_085994229.1:1-452    ERIADTMEQWVNEAGVDGFNI..TYAVTPGTFEEFVDQVVPVLQERGLVQ..KEYEGN..TM
Bacillus_sinesaloumensis_WP_077620983.1:1-449       IEVADAIQYQFEESGVDGFNL..NHLVTPGDLEDFVDLVIPILQERGLYK..TEYKQG..TL
Halopenitus_persicus_WP_021074147.1:6-457           AQIADEMERWVDEAGIDGFNL..TEVLRSGTLSDFVEHVVPELQDRGLHR..EEYTGE..TL
Microbacteriaceae_bacterium                         EQVADRIEELADGIGLDGFNL..VQYLSPGTFEDFIELVVPELQRRGRYR..TEYRPGE.TL
Lysinibacillus_sinduriensis_WP_036201100.1:1-458    SEVADAIQYQFEATGIDGFNL..AHFVTPANIEEFINLVVPELQKRGIYK..ENYTEG..TL
Bacillus_endophyticus_WP_113749053.1:5-457          QQIADIFEEWIDETDIDGFNL..AYTVTPGSFADFVELVIPELQERGLVK..TEYEEG..TL
Pseudomonas_monteilii_WP_119371117.1:1-455          QTVADELQAWVEETDVDGFNL..AYALAHETFRDVVELLVPELQQRGAYK..TEYRPG..TL
Bacillus_endophyticus_50_WP_061801645.1:5-457       QQIADIFEEWIDETDIDGFNL..AYTVTPGSFADFVELVIPELQERGLVK..TEYEEG..TL
Planococcus_maitriensis_WP_112233285.1:1-457        EQVADEVERWRDETGVDGFNL..AYTLAPGSFEDFVELVVPVLQERGLVQ..KEYTEG..TY
Oceanobacillus_profundus_WP_118889880.1:1-447       EQIADEMERWVNEAGIDGFNI..AYAITPGSFKDFVELVIPVLQERGLVQ..TEYEGS..TL
Burkholderia_pseudomallei_WP_141404579.1:1-452      REIADDLQSWVDETDVDGFNL..AYAVTPETFVDVVELVVPELQRRGAYQ..TDYAPG..TL
Terribacillus_saccharophilus_WP_095261886.1:1-455   EEVADAIQHQFEVSGVDGFNL..NHLVTPGDLESFVDLVIPILQERGLYK..QSYKTG..TL
Sediminibacillus_albus_WP_093212831.1:4-453         EQVADTMEQWVEEAGIDGFNI..AYAITPGTFKDFIDLVVPILQERGLVR..KEYRGD..SL
Sciscionella_marina_WP_020496929.1:1-448            DQVADRIEEIAERTGVDGFNL..VQYLSPGTFTDFIELVVPELQRRGRYR..RSYTEGT.TL
Mycetocola_saprophilus_WP_043321267.1:1-452         EQVADRIEEIAEVTGVDGFNL..VQYLSPGTFTDVVELLVPELQKRGRYR..TEYTEH..TL
Lysinibacillus_macroides_WP_053993569.1:4-460       KQIVDKFEEWVDETDIDGFNI..AYAITPGTFIDFADLVTPELQRRGLLK..TAYEEG..VY
Pseudomonas_putida_WP_086978777.1:1-458             QTVADELQAWVEETDVDGFNL..TYALAHETFRDVVELLVPELQQRGAYK..TEYRPG..TL
Sediminibacillus_halophilus_60_WP_074598704.1:4-4   EQIADTMEKWVKEAGVDGFNI..AYAITPGTFQDFVDLVVPILQERGLIR..SSYKGE..TL
Rhizobium_oryzae_WP_085420425.1:1-450               EQIADRLVTWMDEAGVDGFNL..SRTVVPECFDDIIELVIPILQERGLYK..TDYREG..TF
Pseudomonas_extremorientalis_WP_071491956.1:1-455   ETVADLLQEWVEETDVDGFNL..AYALTHETFIDAVDLLVPELQKRGVYK..TEYAQG..TL
Pseudomonas_fluorescens_WP_016977452.1:1-454        ETVADLLQEWVEETDVDGFNL..AYALTHETFIDAVDLLVPELQKRGVYK..TEYAQG..TL
Neorhizobium_sp._T25_27_WP_105419890.1:1-450        EQIADNLVAWMDEAGVDGFNL..SRTIVPECFDDIIELVIPILQERGLYK..TSYREG..PF
Pseudomonas_extremaustralis_WP_078833627.1:1-457    ETVADLLQEWVEDTDVDGFNL..AYALTHDTFIDAVELLVPELQKRGVYK..TEYAQG..TL
Fictibacillus_aquaticus_WP_094253544.1:1-455        VEVADQIQHHFETSGVDGYNL..NHLVTPSCLEDFIDLVIPILQERGIYK..KEYKDG..SL
Pseudomonas_extremaustralis_KAA8559760.1:1-456      ETVADLLQEWVEETDVDGFNL..AYALTHETFIDAVDLLVPELQKRGVYK..TEYAQG..TL
Mycobacterium_sp._ACS1612_WP_067812332.1:1-454      SQVADELQEWVEETGVDGFNL..TNVITPGSFADFVTYVVPELQRRGAYK..TAYSEG..TL
Bacillus_megaterium_WP_116075167.1:1-455            EQVADFMEEWIDDTGMDGFNM..AYVTTPGTFADFIDLVIPVLQKRGLFK..TDYSEG..TY
Pseudomonas_frederiksbergensis_70_WP_123409596.1:   ETVADLLQEWVEETDVDGFNL..AYALTHETFIDAVELLVPELQKRGVYK..TEYAPG..TL
Pseudomonas_citronellolis_SFC61163.1:1-452          KVVADEIEKWLDEDGIDGINL..RQFLTPHTARDFIELVVPELRRRGRFR..EAYNDGE.TL
Herbaspirillum_chlorophenolicum_WP_050468599.1:4-   TQVADGLEAWMADTGIDGFNL..TRTVTPESFEDFVDLVIPVLQERGSYK..RDYADG..TL
Arthrobacter_crystallopoietes_WP_074700854.1:1-45   EQVADRIEEIVDATDVDGFNI..VQFLSPGTFTDVVELLVPELQKRGRYR..TAYSESERTL
Terribacillus_goriensis_WP_038558686.1:1-438        EQVADKIEDFFVRSGVDGFNL..THYITPKDLNDFVELVVPILQERGLYK..RDYAEG..TF
Bacillus_megaterium_WP_025749753.1:1-455            EQVADFMEEWINDTGMDGFNM..AYVTTPGTFADFIDLVIPVLQERDLFK..MDYSEG..TY
Neorhizobium_sp._T7_12_WP_105403044.1:1-451         EQIADNLVAWMDEAGVDGFNL..SRTVVPECFDDIIELVIPILQERGLYK..TGYREG..PF
Rummeliibacillus_sp._SL167_WP_146553619.1:4-459     EKIADEFEEWIDDTDIDGFNL..AYAITPGTFEDIVEFVIPELQKRGIFK..KEYDEG..TL
Burkholderia_sp._PAMC                               VEVADALESWVNEAGVDGFNL..TRTVTPESYEDFIDLVIPELQARGIYK..TTYADG..TF
Pseudomonas_koreensis_WP_064585463.1:4-449          EQVADELESWIAETGLDGFNL..TRIVTPESYVDFIELVIPELQRRGSYK..TAYDSG..SL
Pseudomonas_fluorescens_80_WP_064116961.1:6-451     EQVADELESWIAETGLDGFNL..TRIVTPESYVDFIELVIPELQRRGSYK..TAYDTG..SL
Pseudomonas_rhodesiae_WP_040265276.1:4-450          QQVADELESWISETGLDGFNL..TRIVTPESYVDFIDLVIPELQRRGSYK..TEYEQG..TL
Collimonas_sp._OK412_WP_092393355.1:4-454           QQVADQLEAWIRDTDIDGFNL..TRTVAPESFEDFVNLVIPELQARGSYK..LDYEPG..TL
Pseudomonas_marginalis_WP_058415544.1:1-456         QTVADLLQEWVEETDVDGFNL..AYALTHETFIDAVDLLVPELQKRGVYK..TEYAQG..TL
Pseudomonas_antarctica_WP_064452947.1:1-458         ETVADLLQEWVEETDVDGFNL..AYALTHETFIDAVDLLVPELQKRGVYK..TEYAQG..TL
Pseudomonas_protegens_WP_041115159.1:4-454          QQVADDLESWIAETGLDGFNL..TRIVTPESYVDFIDLVIPELQRRGSYK..TAYDDG..TL
Pseudomonas_synxantha_WP_057024776.1:4-449          EQVADELESWISETGLDGFNL..TRIVTPESYVDFIDLVIPELQRRGSYK..TAYEQG..TL
Rhizobium_leguminosarum_WP_128409501.1:2-451        EQIADHLTTWIEEGGIDGFNL..ARTVAPESLRDFVDLVVPVLQERRVFK..ADYAAG..PL
Pseudomonas_chlororaphis_WP_038359779.1:6-451       EQVADELESWIAETGLDGFNL..TRIVTPESYVDFIDLVIPELQRRGSYK..TAYDTG..SL
Pseudomonas_syringae_90_WP_024642725.1:1-456        ETVADLLQEWVEETDVDGFNL..AYALTHETFIDAVELLVPELQKRGVYK..TEYAKG..TL
Collimonas_fungivorans_WP_061540754.1:4-453         QQVADQLEAWVRDTDIDGFNL..TRTVAPESFEDFVNLVIPELQARGSYK..LEYEDG..TL
Rhizobium_leguminosarum_WP_116409027.1:2-451        EQIADHLATWIEEGGIDGFNL..ARTVAPESLRDFVELVVPVLQERGLFK..VDYADG..PL
Microvirga_ossetica_WP_099511173.1:1-454            EQIADHIASWVEDADIDGINL..PRTVAPESLADFVDLVVPVLQERGLYK..ADYADG..TL
Pseudomonas_caspiana_WP_087268376.1:4-451           EQVADELESWIAETGLDGFNL..TRIVTPESYVDFIDLVIPELQRRGSYK..TAYDSG..SL
Pseudomonas_lundensis_WP_048375063.1:4-451          EQVADELEAWIAETGLDGFNL..TRIVTPESYQDFIELVIPELQRRGSYK..TAYDPG..TL
Caballeronia_mineralivorans_WP_047894899.1:4-456    VQVADALESWMDETGIDGFNL..TRTVTPESYEDFVDLVIPELQARGVYK..TSYADG..TL
Pseudomonas_paralactis_WP_057702387.1:4-450         EQVADELESWISETGLDGFNL..TRVVTPESYVDFIDLVIPELQRRGSYK..TEYEQG..TL
Pseudomonas_taetrolens_WP_048381143.1:4-453         AQVADELESWIEETGLDGFNL..TRIVTPESYVDFIDLVIPELQRRGSYK..TAYDNG..SL
Pseudomonas_psychrophila_WP_123750761.1:4-450       EQVADELESWIEQTGLDGFNL..TRIVTPESYADFIDLVIPELQRRGSYK..TAYDSG..SL
Collimonas_arenae_100_WP_061532973.1:4-454          QQVADQMEAWIRDADIDGFNL..TRTVAPESFEDFVNLVIPELQARGSYK..LDYEDG..TL
Pseudomonas_viridiflava_WP_122210720.1:4-451        EQVADELESWIAETGLDGFNL..TRIVTPESYEDFIDLVIPELQRRGSYK..TAYEEG..SL
Kocuria_varians_WP_068470402.1:1-454                STVADELERWVDEGGLDGINL..AYHVSPGSFEDFVEYVVPELQARGRYR..TEYEGS..TL
Pseudomonas_wadenswilerensis_WP_115084816.1:4-455   TQVADELESWIAETGLDGFNL..ARTVTPESYVDFIDLVIPELQRRGSYK..TAYEQG..SL
Pseudomonas_syringae_WP_011266344.1:4-452           RQVADELESWIEETGLDGFNL..TRIVTPESYEDFIDLVIPELQRRGSYK..TAYQEG..SL
Pseudomonas_sp._286_105WP_122415793.1:4-453         EQVADELESWIAETGLDGFNL..TRIVTPESYAEDFIDLVIPELQRRGSYK..TAYEDG..SL
Geobacillus_vulcani_WP_031406843.1:1-461            IQVADAIEERARRSGVDGFNL..THLITPKDLDDFIEYVVPILQRRGLYK..TAYTPG..TM
Geobacillus_icigianus_WP_033018365.1:1-461          IQVADAIEERARRSGVDGFNL..THLITPKDLDDFIEYVVPILQRRGLYK..TAYTPG..TM
Pseudomonas_floridensis_WP_083182568.1:4-453        EQIADELESWIEETGLDGFNL..TRIVTPESYEDFIDLVIPELQRRGSYK..TAYEDG..SL
Pseudomonas_asturiensis_WP_073167523.1:4-451        EQVADELESWIAETGLDGFNL..TRIVTPESYEDFIDLVIPELQRRGSYK..TAYEDG..SL
Geobacillus_vulcani_110_WP_031406843.1:1-461        IQVADAIEERARRSGVDGFNL..THLITPKDLDDFIEYVVPILQRRGLYK..TAYTPG..TM
```

```
Pseudomonas_syringae_WP_024682626.1:4-452                EQVADELESWIEETGLDGFNL..TRIVTPESYEDFIDLVIPELQRRGSYK..TAYQDG..SL
LadAalpha_Geobacillus_thermoleovorans_BAM76377.1         IQVADAIEERARRSGVDGFNL..THLITPKDLDDFIEYVVPILQRRGLYK..TAYTPG..TM
Tsukamurella_pseudospumae_WP_068746786.1:1-457           QRVADELQEWGEETDVDGFNL..AYAITPGTFEDIVEFIVPELRRRGVYP..SEYTPG..TL
Kocuria_sp._Marseille-P3598_WP_085529073.1:5-456         EKVADELERWIDEGGLDGINL..AYHVTPGSFEDFVEFVVPELQKRGRYR..TEYEGS..TF
Microvirga_aerophila_WP_114188789.1:1-456                EEIADELQSWVEETGVDGFNI..SRVVTPETLEDFVDLVVPILQERGVYK..RDYAEG..TL
Burkholderia_ubonensis_WP_071760483.1:5-455              SQVADELQAWIDETGIDGFNL..TRTVMPESFEDFVDWVVPELQNRGVYK..EDYDPAP.TL
Achromobacter_marplatensis_WP_006226823.1:4-450          AQVADELESWVAETDIDGFNL..ARTVTPECYGDFIDLVIPELQSRGSYK..TAYADG..PL
Acinetobacter_baumannii_WP_070165746.1:1-443             ETVSDALQQWVEDTDIDGFNL..AYILAHQTFADVVEFIVPELQKRGVYQ..TSYAQG..TL
Labedella_phragmitis_WP_128496094.1:1-453                TEIADVLQEWVEETDVDGFNL..AYAITPGTFEDVVEHIVPELRRRGVYP..EEYTPG..SL
Nocardia_cyriacigeorgica_120_WP_130918184.1:1-453        ETVADRLQEWVDDTDLDGFNL..AYAVTPGSFEDIVRHVVPVLTERGVYA..TEYQPG..TL
Nocardia_cyriacigeorgica_WP_130918184.1:1-453            ETVADRLQEWVDDTDLDGFNL..AYAVTPGSFEDIVRHVVPVLTERGVYA..TEYQPG..TL
Mycobacterium_sp._1164985.4_WP_067298521.1:1-453         VRVSDTLQEWVEETDVDGFNL..AYAITPGSFAEFVDHVVPVLTERGAYQ..TSYTPG..TL
Rhodococcus_sp._1R11_WP_135042739.1:1-449                GEVADQLQEWVDKTDVDGFNL..AYAITPGTFEDVVQYVVPELQRRGVYR..TEYTPG..TL
Erwinia_amylovora_WP_004157587.1:5-459                   QTVADELQSWVEETDVDGFNL..AYALTHETFSDVVEHLIPELQKRGVYK..QAYQPG..TL
Ralstonia_mannitolilytica_WP_102079134.1:4-451           GQVADTLIEWIDETGIDGFNL..SRTVVPESYEDFVDLVVPVLQERGRYK..TAYGEG..TL
Leifsonia_flava_WP_135120808.1:15-465                    EEIADTLQEWVDYTDVDGFNL..AYAITPGTFEDVIEFVVPVLRERGAYP..TEYVEG..TL
Cryobacterium_roopkundense_WP_084141147.1:1-453          AEVADHLQDWVADTDVDGFNL..AYAITPGTFEDVIEFVIPELRKRGAYP..EAYAEG..SL
Cupriavidus_pauculus_WP_101681197.1:1-455                EHVADLLMAWSEETGVDGFNL..SRTVVPECFEDVIDLVVPLLQERGAYK..TAYQPG..TY
Rhizobium_sp._BK333_130_WP_133704837.1:2-454             EQIADRLQVWVEKGGIDGFNL..ARTVAPESLRDFIDLVVPVLQERGLFK..ADYAEG..TL
Caballeronia_mineralivorans_WP_047846789.1:1-454         ATVADILQTWVEETGVDGFNL..AYAVAHETFEDIVNYLVPELQRRGVYR..KEYTPG..TL
Nocardia_altamirensis_WP_069163049.1:1-454               ATVADLLQEWVDDTDLDGFNL..AYAVTPGSFEDVVRHVVPVLAARGAYA..TEYVPG..TL
Cupriavidus_sp._P-10_WP_116322758.1:1-452                KKVADELQQWMEETDIDGFNL..AYTVAPESINDFVELVVPELQARGVYK..TEYTQG..TL
Serratia_sp._S1B_WP_116726564.1:1-452                    ETVADALQEWVAETDVDGFNL..AYILAHQTFSDVVEFIVPELQKRGVYQ..TEYAQG..TL
Pseudomonas_putida_WP_043864435.1:1-452                  QTVADQLQEWVQETDVDGFNL..ASVVAPETFEAVVDLLVPELQARGLFK..RDYAQG..TL
Pseudomonas_aeruginosa_WP_023084248.1:4-453              RQVADDLEGWIADTDLDGFNL..TRTVEPESYADFIDLVVPELQARGSYK..TAYAPG..TL
Pseudomonas_mucidolens_WP_084381795.1:9-455              EQVADELESWINETGLDGFNL..TRMVTPESYVDFIDWVIPELQRRGSYK..TAYEGG..TL
Pseudomonas_poae_WP_060549803.1:4-450                    EQVADELELWISETGLDGFNL..TRIVTPESYVDFIDLVVPELQKRGSYK..TEYETG..TL
Pseudomonas_chlororaphis_WP_123573412.1:9-454            QQVADDLESWIAETGLDGFNL..TRIVTPESYADFIDLVIPELQRRGSYK..TAYDNG..TL
Janthinobacterium_lividum_140_WP_072454679.1:1-44        RQVADQLESWLDETGIDGFNL..AFAVAHDSMADVVEHIVPELQRRGRYH..THYEEG..SL
Haladaptatus_cibarius_WP_049970513.1:4-446               EQVADELEAWRDETGVTGFNL..KELVRPGTLWDFVDLVVPELEKRGLVR..EEYEGE..TL
Arthrobacter_luteolus_WP_066298473.1:1-443               ETVADEVERWVDEAGLDGINL..AYAVTPGSFEQFVDLVVPELRKRGRVW..DGYDGG..TL
Geobacillus_sp._ZGt-1_WP_047758532.1:1-436               IQVADAIEERARRSGVDGFNL..THLITPKDLDDFIEYVVPILQRRGLYK..TAYTPG..TM
Lysinibacillus_composti_WP_124766485.1:4-458             EQIADTMEQWVNEAGVDGFNI..AYAITPGTFKDFGELVVPILRERGLVP..EEYEGN..TF
Microcella_putealis_WP_130486201.1:20-474                VEIADQLEQWVDETDVDGFNL..AYAVTPGTFADVVEHVVPVLRERGLYP..DAYDEG..TL
Pseudomonas_nitroreducens_WP_037016976.1:1-453           QTVADQLQAWVEETDVDGFNL..SSLVAPETFVDIVDLLVPELQARGLFK..REYEQG..TL
Acinetobacter_wuhouensis_WP_130168784.1:1-453            STVADELQHWVDDTDVDGFNL..AYILAHQSFIDVVEHIVPELQRRGVYQ..TAYREG..TL
Bradyrhizobium_sp._YR681_WP_008144663.1:1-454            ESVADKIEDWFEKTDVDGLNV..AFAISPGDFEDIADMLVPELTKRGRYK..PEYAKG..TL
Schumannella_sp._10F1D-1_WP_141164655.1:10-460           EQIADELQSWVDETDVDGFNL..AYAITPGTFEDIVEFVIPILRERGVYP..TEYQPG..TL
Pseudomonas_coronafaciens_150_WP_122355187.1:4-45        EQVADELESWIEETGLDGFNL..TRIVTPESYEDFIDLVIPELQRRGSYK..TAYQDG..TL
Pseudomonas_syringae_WP_003430238.1:4-452                EQVADELESWIEETGLDGFNL..TRIVTPESYEDFIDLVIPELQRRGSYK..TAYQDG..SL
unclassified_Rhodococcus_WP_094682261.1:1-449            GEVADQLQEWVDETDVDGFNL..AYAITPGTFEDVVRFVVPELQRRGVYR..TEYTPG..TL
Hafnia_alvei_WP_046449330.1:6-449                        SQVADALQVWIDEADVDGFNL..TRIVNPGSYRDFIDLVVPELQSRGVYK..TEYAEG..AL
AS9A_3890_Hoyosella_subflava_WP_013808677.1:1-453        NTVADHIQNWVTETDVDGFNL..AYAITPGSFEDVITHVVPVLQERGAYP..ATYSQG..SL
Acinetobacter_baumannii_WP_000995600.1:1-443             ETVSDALQQWVEDTDVDGFNL..AYILAHQTFADVVEFIVPELQKRGVYQ..TSYAQG..TL
Agrobacterium_sp._B133/95_WP_065696417.1:2-454           EQIADRLQLWMEEGGIDGFNL..ARTVAPESLHDFVDLVIPVLQERGLFK..ADYAEG..TL
Microbacterium_sp._KROCY2_WP_024289149.1:1-453           QTVADQLETWIDEGGLDGINL..AYHVSPGSFEDFVEHVVPELQKRGRYR..TDYDGP..TL
Variovorax_paradoxus_WP_012747040.1:6-452                SQVADELQSWIDETGADGFNL..SRTVVPESYEDFVDLVVPELQNRGIYK..TAYAEG..SL
Methylobacterium_radiotolerans_WP_076730229.1:5-4        VTIADELQAWITEGEVDGFNL..SRIVVPESFSDFIDLVIPELQDRGLYK..TAYAEG..SL
Streptomyces_sp._BK215_WP_132915265.1:7-452              EQIADELERW.GKAGVDGFNV..TYVTTPGTFEDFIQHVVPVLQERGLMR..REYTPG..TL
Geobacillus_thermodenitrificans_160_WP_008880416.        IEVADAIEERARRSGVDGFNL..VHLITPKDLDDFIEYVVPILQRRGLYK..TAYPPG..TM
Pseudomonas_aeruginosa_WP_132556951.1:356-785            RQVADDLEGWIADTGLDGFNL..TRTVEPESYADFIDLVVPELQARGSYK..TAYAPG..TL
Penicillium_solitum_OQE02104.1:14-471                    AQVADNMERWVAEADVDGFNL..AYAIKPGSFKDIIDLLIPELRKRGLFW..DDYAVPKGTY
Penicillium_vulpinum_OQE05502.1:12-469                   AQVADSMERWVEEADVDGFNL..AYAIKPGSFKDIIDLLIPELRKRGLFW..DDYAVPKGTY
Clavibacter_michiganensis_WP_045526194.1:1-454           TTVADELERWIEVGGTDGFNL..AYVVTPGTFEAVVDLLVPELRRRGRVW..DEYPEG..TL
Glutamicibacter_creatinolyticus_WP_054821484.1:6-        AEVAEKLIDWVDYTDVDGFNL..AYAITPGTFEDIVEYVVPELQARGAYK..TEYAEG..SL
Campylobacter_sp._P0078_WP_086237522.1:4-454             KEVCDELEKLVEHSDADGFNL..AYATTPGTFEDVVEFIVPELQKRGSYQ..LEYQSG..SL
Pseudomonas_furukawaii_WP_003454569.1:1-447              SQVADQLLAWIDETGLDGFNL..TRTVTPESYIDFIDLVIPELQQRGRYK..TAYADG..AL
Leucobacter_triazinivorans_WP_130111548.1:1-442          ATVADEMERIIAETGVDGFNL..AYAVTPGTFEDVVEHVVPELQRRGVYP..TEYAPG..TL
Aspergillus_brasiliensis_CBS_101740_OJJ68286.1:12        QQVADEMERWVNEADVDGFNL..AYAIKPGSFKDIIDLLIPELRKRGLFW..DDYAVKKGTY
Aspergillus_udagawae_170_GAO89440.1:15-472               AQVADEMERWVREADVDGFNL..AYAIKPGSFKDIIDLLIPELRRRGLFW..EDYAVERGTY
Aspergillus_flavus_RMZ37351.1:15-472                     EQVADEMERWVREADVDGFNL..AYAVKPGSFKDMIELLIPELRRRGLFW..DDYAVNKGTY
Aspergillus_flavus_RAQ54081.1:15-472                     EQVADEMERWVREADVDGFNL..AYAVKPGSFKDIIELLIPELRRRGLFW..DDYAVDKGTY
Penicillium_arizonense_XP_022490779.1:12-469             AQVADNMERWVEEADVDGFNL..AYVVKPGSFKDIIDHLIPELRRRGLFW..DDYAVPKGTY
Salinibacterium_sp._CGMCC                                EEIADQLQEWVDYTDVDGFNL..AYAVTPGTFEDIVEHVIPVLQARGVYP..TEYAKG..TL
Aspergillus_fumigatus_OXN06785.1:14-471                  AQVADEMERWVREADVDGFNL..AYAIKPGSFKDIIDLLIPELRRRGLFW..EDYAVERGTY
Aspergillus_lentulus_GAQ10210.1:7-464                    AQVADEMERWVREADVDGFNL..AYAIKPGSFKDIIDLLIPELRRRGLFW..EDYAVERGTY
Aspergillus_flavus_RAQ66103.1:15-472                     EQVADEMERWVREADVDGFNL..AYAVKPGSFKDMIELLIPELRRRGLFW..DDYAVNKGTY
Aspergillus_fumigatus_OXN22894.1:14-471                  AQVADEMERWVREADVDGFNL..AYAIKPGSFKDIIDLLIPELRRRGLFW..EDYAVERGTY
Aspergillus_nidulans                                     EQVADEMERWVREADVDGFNL..AYAIKPGSFKDIIDLLIPELRRRGLFH..EDYAAPRATY
Penicillium_nalgiovense_OQE79848.1:14-471                AQVADSMERWVQEADVDGFNL..AYAIKPGSFKDIIDLLIPELRKRGLFW..DDYAVPKGTY
Aspergillus_wentii                                       TTVADELERWVEVSGVDGFNL..AHITNPGTFEDIIEYLLPELRRRGIFR..SEVEKEGVTA
Gordonia_iterans_WP_105943624.1:1-453                    DEIAQTFADWVDETDVDGFNI..AYAVTPGSFEDIVDHVLPSLVRRGAYP..SEYRPG..TL
Azotobacter_beijerinckii_WP_090735436.1:4-454            QQVADALANWIEQTDLDGFNL..TRTVTPESYADFIDLVVPELQSRGLYK..TAYAAG..SL
Staphylococcus_sp._NAM3COL9_WP_057513692.1:4-453         EQVADKFEAWALEGGADGFNI..AQSYSPGTFEEFVDHVIPELQKRGLYR..TEYEGT..TL
Acidovorax_sp._56_WP_099656603.1:11-450                  DQVADALQSWVDDTGVDGFNL..TRTVVPESFEDFIDIVVPELQNRGLHK..SSYTQG..SL
Paenibacillus_sp._BIHB4019_WP_099518466.1:1-456          EDVADQIERWTEETDVDGFNL..EPYVLPGSYVDFVDLVIPELQKRGLFK..TDYEAG..TL
Penicillium_antarcticum_OQD90033.1:5-465                 KTVADELERWVSVGDVDGFNF..SYASLPDTFDDVIQLLLPELQRRGLFW..SDYAVKGGTL
Aspergillus_oryzae_RIB40_190_XP_001823703.1:9-463        TTVADELERWVEVAGVDGFNL..AHITNPGTFEDIIEYLLPELRRRGRFRSVVGKEGA..TA
Fusarium_oxysporum_f._sp._pisi                           ERIADEMERWVNEADVDGFNL..AYALMPASFEDVISDLLPVLRERGLFW..EDYAVDGGTY
Colletotrichum_fructicola_Nara_gc5_ELA35345.1:18-        TTAADELERWVQGADVDGFNL..AHVTNPGTFEDIIEHLLPELRKRGIFR..SEVEKEGATA
Micrococcus_luteus_WP_041104460.1:19-468                 ESAARQLVEWADETDVDGFNL..AYHITPGTFEDVVEFVVPELQKLGRYK..TAYTDG..TL
Aspergillus_awamori_GCB21923.1:12-469                    EQVADEMERWVNEADVDGFNL..AYAIKPGSFKDIIDLLIPELRKRGLFW..DDYAVKKGTY
Aspergillus_niger_CBS                                    EQVADEMERWVNEADVDGFNL..AYAIKPGSFKDIIDLLIPELRKRGLFW..DDYAVKKGTY
Aspergillus_lacticoffeatus_CBS_101883_XP_02545389        EQVADEMERWVNEADVDGFNL..AYAIKPGSFKDIIDLLIPELRKRGLFW..DDYTVKKGTY
Aspergillus_wentii_DTO_134E9_OJJ40237.1:14-471           AQVADYMERWVQEADVDGFNL..AYAVKPGSFKDIINLLLPELRRRGLFH..DDYAVNKGTY
Aspergillus_indologenus_CBS_114.80_PYI31207.1:11-        AQVADEMERWVAEADVDGFNL..AYAIKPGSFKDIIDLLLPELRRRGLFW..EDYAVPRGTY
Aspergillus_fischeri_NRRL_181_XP_001264002.1:14-4        AQVADEMERWVREADVDGFNL..AYAIKPGSFKDIIDLLIPELRRRGLFW..EDYAVERGTY
Penicillium_coprophilum_200_OQE46388.1:11-466            KTVADELERWVEISDVDGFNL..AHIVNPGTFEDIIEFLLPELRRRGVFR..EGIEKEGATA
Cupriavidus_taiwanensis_WP_116379876.1:8-454             SQVADTLAGWVDETGIDGFNL..SRTVVPESYEDFVDLVVPALQERGRYK..TAYADG..TL
Penicillium_digitatum                                    KTVADELERWVTVGDVDGFNL..SYASLPDTFDDVIRLLLPELQRRGLFW..SDYAVKGGTF
Aspergillus_niger_GAQ46262.1:12-469                      EQVADQMERWVNEADVDGFNL..AYAIKPGSFKDIIDLLIPELRKRGLFW..DDYTVKEGTY
Aspergillus_nomius_NRRL_13137_XP_015412385.1:15-4        QQVADEMERWVEEADVDGFNL..AYAVKPGSFKDIIELLIPELRRRGLFW..DEYAVNKGTY
Aspergillus_aculeatus_ATCC_16872_XP_020055010.1:1        AQVADEMERWVAEADVDGFNL..AYAIKPGSFKDIIDLLLPELRKRGLFW..EDYAVPRGTY
Penicillium_brasilianum_OOQ84969.1:19-474                TTVADELERWVEVSGVDGFNL..AHIANPGTFEDIIEFLLPELRRRGRFRSKVEHEGA..TA
Penicillium_subrubescens_OKP15100.1:19-474               TTVADELERWVEVSGVDGFNL..AHIANPGTFEDIIEFLLPELRRRGRFRSEVEHEGA..TA
Trichoderma_arundinaceum_RFU80530.1:19-474               TTVADELERWIEVSGVDGFNL..AHVTNPGSFEDIIEHLLPELRRRGVFR..NGIEKEGATA
Aspergillus_thermomutatus_210_XP_026614594.1:15-4        AQVADEMERWVREADVDGFNL..AYAVKPGSFKDIIDLLLPELRRRGLFW..EDYAVERGTY
Serratia_liquefaciens_WP_044551273.1:4-449               QQVADALLQWIDQADIDGFNL..TRIVNPGSYRDFIDLVVPELQSRGRYK..TAYRAG..SL
Micrococcus_luteus_WP_065572847.1:19-468                 ESAARQLVEWADETDVDGFNL..AYHITPGTFEDIVEFVVPELQKLGRYK..TAYTDG..TL
Halolamina_rubra_WP_049979911.1:1-449                    EQVADELVYWHESVGVDGFNV..KEAVRTGSLDEFVDQVVPELQDRGLFR..TEYEGD..TL
Acinetobacter_pittii_WP_075382847.1:7-452                EEVAEYLIHLLDETDIDGFNL..TRTVAPESHHDFIRLVIPELQQRGRYK..TAYKTG..SL
Corynebacterium_variabile_WP_052302534.1:2-464           ESVADQLENWVEATGVDGFNL..AYAVTPGTFRDVIDHVVPVLQARGEYP..TTYAPG..TL
Aspergillus_luchuensis_GAT21254.1:12-471                 QQVADQMERWVNEADVDGFNLIQAYAIKPGSFKDIIDLLIPELRKRGLFW..DDYTVKKGTY
```

```
Aspergillus_luchuensis                                QQVADQMERWVNEADVDGFNL..AYAIKPGSFKDIIDLLIPELRKRGLFW..DDYTVKKGTY
Aspergillus_bombycis_XP_022386026.1:15-472            QQVADEMERWVEEADVDGFNL..AYAIKPGSFKDIIELLIPELRRRGLFW..DEYAVNKGTY
Agrobacterium_vitis_WP_070163524.1:1-454              DQVADILQEWVTDTDVDGFNI..AYAVTPGSFEDVVTYIVPELQKRGAYP..TAYKPG..TL
Pseudomonas_psychrotolerans_220_WP_074584491.1:5-     GQVADALLGWIDETGLDGFNL..ARTVTPECYGDFIDLVIPELQSRGRYK..TAYAEG..SF
Micrococcus_yunnanensis_WP_135040276.1:19-468         ESAARQLVEWADETDVDGFNL..AYHITPGTFEDIVEFVVPELQKLGRYK..TAYTDG..TL
Acinetobacter_calcoaceticus_WP_004642549.1:7-452      EEVAEYLIHLLDETDIDGFNL..TRTVAPESHHDFIRLVIPELQQRGRYK..TAYKTG..SL
Penicillium_brasilianum_CEO59775.1:15-472             AQVADSLERWVQEADVDGFNL..AYAIKPGSFKDIIELLIPELRRRGLFW..DDYAVPKGTY
Aspergillus_cristatus_ODM22551.1:12-469               QQVADQMEKWVDEADVDGFNI..AYAVKPGTFKDVIDLLIPELRRRGLFH..EDYAVPKGTY
Pseudomonas_taiwanensis_WP_027907363.1:1-446          GEVADQLETWLDQTGIDGFNL..TYAVQPDDLTNVVELLVPELQHRGRYP..TAYTEG..TL
Acidovorax_avenae                                     QDIADEMQSWLDEAGVDGFNL..ARTVVPESYTDFIDLVVPELQNRGLHK..TAYAEG..TL
Staphylococcus_pseudintermedius_WP_101431388.1:4-     TQIANQLELWANEGGADGFNI..AQAYSPGTFEEFVDFIIPELQKRGIYR..TEYEGE..TL
Rhodococcus_triatomae_WP_081607437.1:2-451            TTVADRLEELADEAGLDGFNV..YDNLPLRTLPDFVDLVVPELQRRGRVP..TAYEAD..TL
Alcaligenes_sp._RS4_230_WP_128393375.1:4-450          REVADQLERWIDEGDIDGFNL..SRIVVPETYEDFVDLVVPELQARGRYK..TAYEPG..TL
Gibbsiella_quercinecans_WP_121552947.1:7-454          QQVADNLLQWVDQAGIDGFNL..TRIVNPQSYTDFIDLVIPELQARGRYK..TAYAAG..SF
Acinetobacter_ursingii_WP_044435211.1:5-452           SHVAEQLIRLLDDTGIDGFNL..TRTVAPESHQDFIRFVVPELQHRGRYK..TEYQAG..SL
Erwinia_typographi_WP_034887712.1:4-447               SQVADALINWIDEGDIDGFNL..TRILNPQSYRDFIDLVVPELQQRGRYK..TAYQPG..SL
Trichoderma_harzianum_KKP01913.1:14-470               EKVADEMERWVREADVDGFNI..AYALMPQTFQDVIDLLLPVLKERGLFW..DGYAVDGGTY
Fusarium_graminearum_235_CEF79766.1:12-466            TQVADSLEEWVTEAGVDGFNF..GYVLFPQSFKDISDLLIPELRSRGLFW..DDYAVPGGTY
Gordonia_alkanivorans_WP_006357654.1:10-461           DEVAEQLQDWVAETDVDGFNV..AYAVTPGSFEDIVTHVIPALERRGVYD..RGYVPG..TL
Acinetobacter_radioresistens_WP_111281495.1:5-455     ATVAEQLIHLIDETGIDGFNL..TRTVAPESHQDFIRWVIPELQQRGRYK..TAYSEG..TL
Mesorhizobium_plurifarium_WP_073985566.1:5-458        VKAADQLEAWFDEAGIDGFNI..TDPMPLKSFPDFNRHVLPELRRRGRVR..ERYEGA..TY
Oceanobacillus_halophilus_WP_121203049.1:1-454        EQIADKLETWAIEGDADGFNI..AQSYSLETFREFVDHVVPELQKRGIYR..TEYKGK..TL
Acinetobacter_tandoii_240_WP_100241535.1:7-457        AAVAEQLIQWIDETGIDGFNL..TRTVAPESHQDFIRYVIPELQQRGRYK..TSYSAG..SL
Burkholderia_plantarii_WP_042624083.1:1-457           AQVADELQAIVDDTGIDGFNL..AWVESPRTFEDVVRYLVPELQRRGAYK..TRYETG..TL
Alcaligenes_faecalis_WP_094195208.1:6-457             QQVADALTTWIDEGEIDGFNL..TRIVVPETWEDFANLVVPELQNRGRYR..TAYEGG..TL
Bradyrhizobium_arachidis_WP_092214516.1:1-453         TEVADQLQAWVAETDIDGFNL..SYAVTPGGYQDFAELVVPELQRRGVYK..TGYAKG..TL
Cupriavidus_plantarum_WP_109583068.1:1-458            RQVADTLEAWMDETGVDGFNL..ASVEMPRTFEDIVAHLVPELQRRGVYK..TEYADG..TL
Variovorax_sp._770b2_245_WP_093443018.1:5-461         AQVADEIEAWVDEAGVDGFNL..VRTVSPEGLEHFVDLVVPELQRRGLHK..TAYANG..VW
RZJ22128.1:4-447                                      EQVVDQLQNWFENEAADGFNI..LPPTTPAGLNDFVDFIVPELQRRGLFR..TEYEGK..TL
LadB_Geobacillus_thermoleovorans_BAM76371.1           EQVADRLIEFIS.LGFDYVLL..RGFPHLETIEQVGTSVIPLVRERLRQT............
LadAbeta_Geobacillus_thermoleovorans_BAM76372.1       EQVADQLEYWFLQGAADGFNL..MFPYYPNGLQSFVEHVIPILQKRGLFR..RNYEGT..TL
Af1_AFLA_024140_XP_002373925.1                        EQVADEMERWVREADVDGFNL..AYAVKPGSFKDIIELLIPELRRRGLFW..DDYAVNHGTY
Af3_AFLA_58870_XP_002378631.1                         KTVADELERWVEVGDVDGFNL..SYASIPETFDDIIKYLIPELQKRGIFH..TDYAVKGGTF
Af4_AFLA_049780_XP_002383105.1                        QRVADEMERWIREADLDGFNL..GYVTTPGTFEEVVDLLIPELRRRGLYP..ASAEADGLTA
Af5_AFLA_126060_XP_002381799.1                        STVADILQRWVDVADVDGFNF..SYAVSPGTFEDMIEYLFPELRRRGVIW..DDYEVKGGSA
Af2_QMW32876.1:9-463                                  TTVADELERWVEVAGVDGFNL..AHITNPGTFEDIIEYLLPELRRRGRFRSVVGKEGA..TA
Yarrowia_lipolytica_CLIB122_YALI0C07546p_XP_50156     ETVADIIEDWIEIGDLDGFNL..SHATFPGTYDDIVGYLIPELQKRGRFH..EDYPENEHTF
Rhizobium_sp._CF122_WP007794079.1                     SRIADEMEHWFTTGAADGFNV..MPPHLPVGLRDFTEFVLPELRRRGLFR..TEYQGR..TL
Acinetobacter_sp._NIPH_2100_LLM                       EQIVDQLQQWFENEAADGFNI..LPPSTPAGLNDFVELIVPELQRRGLFR..TEYEGT..TL
Nostoc_sp._PCC_7107_WP_015115963.1                    ESIADQLEEWFNNGAADGFNI..MPPILPTALDDFVNLVVPILQKRGLFR..TEYEGS..TL
Aspergillus_flavus_NRRL3357_uncharacterized           ESIAEEMSVWLDEEGADGFTV..VFPFLPQGLDDVVQRLVPELQRRGIFR..QDYEGT..TL
Paenibacillus_curdlanolyticus_WP006037998.1           TQIADQLQEWFENGAADGFNI..MPPYLPGGLDDFVDLVVPELQRRGLFR..TEYEGA..TL
LadAbeta_Geobacillus_thermoleovorans_FMN-dependen     EQVADQLEYWFLQGAADGFNL..MFPYYPNGLQSFVEHVIPILQKRGLFR..RNYEGT..TL
SsuD_1M41                                             PTVAARINEYAA.LGIDSFVL..SGYPHLEEAYRVGELLFPLLD........VAIPEI..PQ
Paenibacillus_curdlanolyticus_WP006037998.1           TQIADQLQEWFENGAADGFNI..MPPYLPGGLDDFVDLVVPELQRRGLFR..TEYEGA..TL
Bacillus                                              .QAADRL.......................................................
Pseudomonas                                           RQVAERIGEYA.ELGIDSFIF..SGYPHLEEAYRFAELVFPLLPE.................
LadB_Geobacillus_thermoleovorans_FMN-dependent        EQVADRL.............L.........................................
Pseudomonas_aeruginosa_PAO1_NP_251290.1               EQVAAALGEYYR.LGVSTFLI..RGFDPLGDAVRYGQGLIPAIHDH..............IA
Alkanesulfonate                                       ..............................................................
Alkanesulfonate                                       QTIVDSILDYVD.LGAELISI..RGYDNLNDAIDYGRYILPPVRQ...............AL
Aspergillus_niger_ATCC13496_RDH19503.1                QTISDSILDYVD.LGADLISI..RGYDNLNDAIDYGRYVLPKV..RGELK..........A
Penicillium_digitatum_Pd1_XP014534980.1               QTIVDSLIDYVD.LGADLISI..RGYDNLNDAIDYGRYILPKVRE...............TL
Acinetobacter_rudis_WP_016657872.1                    ETVVARIKEYAD.LGIESFIF..SGYPHLEEAYRFAELVFPLLPEPYASY..AQFGQT..NL
```

```
                                                      420       430       440
LADA_Geobacillus_thermodenitrificans_WP_011888513 REKLF....GKGNYRLPDDHIAARYRNISSNV..........
Geobacillus_sp._LEMMY01_WP_079935807.1:1-440 REKLF....GKGNYRLPDDHIAARYRNISSNV..........
Geobacillus_stearothermophilus_ABV66256.1:1-440 REKLF....GKGNYRLPDDHIAARYRNISSNV..........
Geobacillus_sp._B4113_201601_WP_061912600.1:1-440 REKLF....GKGNYRLPDDHIAARYRNISSNV..........
Geobacillus_sp._B4113_201601_5WP_061912600.1:1-44 REKLF....GKGNYRLPDDHIAARYRNISSNV..........
Bacillus_thuringiensis_6WP_029441578.1:1-435 REKLF....GKGNSKLPNNHFINEYRN...............
Lysinibacillus_sphaericus_WP_103976887.1:2-422 REKLF..............NLKHKYR................
Lysinibacillus_sphaericus_WP_069508309.1:2-422 REKLF..............NLKHKYR................
Rummeliibacillus_sp._SL167_10_WP_146551437.1:2-42 REKLF..............YGKHEYKF...............
Paenibacillus_sp._DXL2_WP_110843609.1:1-459 RERLF....GGGVVRLPDDHPGARYRRSASPI..........
Paenibacillus_naphthalenovorans_WP_074730028.1:1- RERLF....GKGTVQLPEGHPGSVYRRTTTT...........
Cohnella_sp._HS21_WP_130606586.1:1-453 REKLF....GPGESRLPDRHPGAAFR................
Streptosporangium_subroseum_15_WP_089205784.1:1-4 RERLL....GPGIHRLPETHPGAAFRR...............
Arthrobacter_sp._Hz2_WP_120147654.1:1-455 RERLF....GPGNTRLFDQHPGARYRN...............
Nonomuraea_solani_WP_103963668.1:1-453 RERLL....GPGVHRLPATHPGAAHRR...............
Arthrobacter_ruber_WP_105030338.1:1-455 RERLF....GPGSARLPDRHPGARYRN...............
Bacillus_aciditolerans_20_WP_121446947.1:1-456 REKLF....NQGSSLLPNDHPGSKYRKTA.............
WP_137163763.1:1-458 RHKLF....GRG.DRLPEGHAGRQVRIQPANT..........
Aeribacillus_pallidus_WP_130157503.1:4-457 REKLF....GKGQSRLPEDHIGAKYRNLS.............
Bacillus_megaterium_24WP_098602848.1:4-460 REKLF....GKGQPRLSKEHIGSTYQKISYQI..........
Streptomyces_sp._150FB_WP_078877664.1:5-455 RERLF....GPGSARLLDDHPGKRFRDL..............
Arthrobacter_sp._DWC3_WP_104051071.1:1-454 RERLF....GAGNTRLFDQHPGARYR................
Cohnella_thermotolerans_WP_027093898.1:1-456 REKLF....GPGKRLLPDHHPGAKVRTF..............
Paenibacillus_bovis_28WP_144921429.1:1-452 RGNLF....GAG.PQLPDHHPGKSY.................
Bacillus_aciditolerans_WP_121447760.1:1-452 RESLF....GRG.SQLPGHHPGKQY.................
Leifsonia_sp._ALI-44-B_30_WP_077053645.1:1-455 RERLF....GAGNARLFPEHPGARYR................
Parageobacillus_thermoglucosidasius_WP_064552269. REKLF....NHGSSLLPEDHPGSAYRSKS.............
Geobacillus_sp._Y4.1MC1_WP_013399886.1:1-459 REKLF....NYGSSLLPEDHPGSAYRSKS.............
Bacillus_methanolicus_35WP_004434428.1:1-462 RQKLF....GHGSNLLPEDHPGSKYRRIYSSV..........
Terribacillus_halophilus_WP_077309754.1:1-448 RENL......SGNSKLPDRHPA....................
Paraburkholderia_kururiensis_WP_017777532.1:1-453 REKLH....GGG.ARLAEPHPGARYRS...............
Gracilibacillus_massiliensis_WP_058308419.1:1-456 REKLF....PDGNSRLPEDHPGSKFR................
Streptomyces_yanglinensis_WP_103888551.1:5-452 RERLF....GAGRTRLPEDHPGTRY.................
Trinickia_sp._7GSK02_40_WP_136896361.1:1-452 REKLH....GRG.ARLAEPHPGAAYR................
Cryobacterium_sp._NEAU-85_WP_123045138.1:1-448 RERLF....DKG.PLAPDAHPAGQAR................
Alicyclobacillus_acidoterrestris_WP_021298468.1:4 RDNLS....GRG.SFLLDDHPARKYRS...............
Oceanobacillus_senegalensis_WP_085994229.1:1-452 RANLT......GQDQLPDHHPGKSYR................
Bacillus_sinesaloumensis_WP_077620983.1:1-449 REKLF....PQGKSILPEDHPGSKYRRVPA............
Halopenitus_persicus_WP_021074147.1:6-457 RETMF....ETGDGFLHEDHTGSAYADDS.............
Microbacteriaceae_bacterium RERLF...EGRG.PLLPERHPAGALREAARS...........
Lysinibacillus_sinduriensis_WP_036201100.1:1-458 REKLF....GTGRNLLPNDHIGAKYRNAYS............
Bacillus_endophyticus_WP_113749053.1:5-457 REKLF....GKGRARLSEEHVGSQYRNMN.............
Pseudomonas_monteilii_WP_119371117.1:1-455 REKLF....GDG.PRLPATHPAAGYRDLS.............
Bacillus_endophyticus_50_WP_061801645.1:5-457 REKLF....GKGRARLSEEHVGSQYRNMN.............
Planococcus_maitriensis_WP_112233285.1:1-457 REKIY....GKGQAQLKDNHPGRTFKHL..............
Oceanobacillus_profundus_WP_118889880.1:1-447 RDNLF....GKG.DKLLDTH......................
Burkholderia_pseudomallei_WP_141404579.1:1-452 REKLY....RAG.PRLAAPHPAARYR................
Terribacillus_saccharophilus_WP_095261886.1:1-455 REKLF....DYESSRLPSGHPASNYR................
Sediminibacillus_albus_WP_093212831.1:4-453 RDNLF....GNG.ARLPENHPGRQDR................
Sciscionella_marina_WP_020496929.1:1-448 RERLH....GEG.PRLPASHPGAAHR................
Mycetocola_saprophilus_WP_043321267.1:1-452 RERLF...PGQG.SQLPDTHPAAAYR................
Lysinibacillus_macroides_WP_053993569.1:4-460 REKLF....GKGKAYLAQEHIGANYRKDSNKA..........
Pseudomonas_putida_WP_086978777.1:1-458 REKLF....GDG.PRLPASHPAAGYRDLSRQL..........
Sediminibacillus_halophilus_60_WP_074598704.1:4-4 RDNLF....GAG.PRLSENHPGKKY.................
Rhizobium_oryzae_WP_085420425.1:1-450 REKLF......GSPRLPDRHVAAGYR................
Pseudomonas_extremorientalis_WP_071491956.1:1-455 REKLF....GDG.ARLASNHPGAGYRNLS.............
Pseudomonas_fluorescens_WP_016977452.1:1-454 REKLF....GEG.ARLPEVHPGSGYRNL..............
Neorhizobium_sp._T25_27_WP_105419890.1:1-450 REKLF......GAPRLPDRHIAARYR................
Pseudomonas_extremaustralis_WP_078833627.1:1-457 REKLF....GEG.PRLPSNHPGAGYRDLSGS...........
Fictibacillus_aquaticus_WP_094253544.1:1-455 REKLF....SHGRSVLPNDHPGRKF.................
Pseudomonas_extremaustralis_KAA8559760.1:1-456 REKLF....GDG.ARLSANHPGAGYRNLSA............
Mycobacterium_sp._ACS1612_WP_067812332.1:1-454 RDKLF....GRG.PRLPETHRAAAYRRVKEGV..........
Bacillus_megaterium_WP_116075167.1:1-455 REKLF.....KKSHFLPDEHYGAQYRKTS.............
Pseudomonas_frederiksbergensis_70_WP_123409596.1: REKLF....GEG.PRLPDIHPGAGYRDLA.............
Pseudomonas_citronellolis_SFC61163.1:1-452 RERLF....GAGHARLPADHHGARYRDPAA............
Herbaspirillum_chlorophenolicum_WP_050468599.1:4- REKLF.....GGASVLPQNHAAAAFR................
Arthrobacter_crystallopoietes_WP_074700854.1:1-45 RERLF...PGNG.PLLPATHPGAAYR................
Terribacillus_goriensis_WP_038558686.1:1-438 REKLY......G..............................
Bacillus_megaterium_WP_025749753.1:1-455 REKLF.....KKSHFLPDEHYGVRYRKTS.............
Neorhizobium_sp._T7_12_WP_105403044.1:1-451 REKLF......GTQRLPSRHIAARYRT...............
Rummeliibacillus_sp._SL167_WP_146553619.1:4-459 REKLF....GES.SYLRPDHIGHKYKRIPESI..........
Burkholderia_sp._PAMC REKLF....GGA.AHLPQEHAGASFRNLS.............
Pseudomonas_koreensis_WP_064585463.1:4-449 REKLF.....HGEAQLPEQHTGASFR................
Pseudomonas_fluorescens_80_WP_064116961.1:6-451 REKLF.....HGEAQLPEQHTGSSYR................
Pseudomonas_rhodesiae_WP_040265276.1:4-450 REKVF.....HASARLPDQHTGSSYRH...............
Collimonas_sp._OK412_WP_092393355.1:4-454 REKLF....EAGQPTLPGRHPAAGYRQV..............
Pseudomonas_marginalis_WP_058415544.1:1-456 REKLF....GDG.ARLAPNHPGASYRDLAA............
Pseudomonas_antarctica_WP_064452947.1:1-458 REKLF....GEG.ARLASNHPGAGYRDLKATT..........
Pseudomonas_protegens_WP_041115159.1:9-454 REKLF....RQG.RKLPEQHTGAAYR................
Pseudomonas_synxantha_WP_057024776.1:4-449 REKVF.....HGAARLPEQHTGATYR................
Rhizobium_leguminosarum_WP_128409501.1:2-451 RQKLF....GGGNGRLPAAHPAAQFR................
Pseudomonas_chlororaphis_WP_038359779.1:6-451 REKLF.....HGEAQLPEQHTGAAFR................
Pseudomonas_syringae_90_WP_024642725.1:1-456 REKLF....GDG.PRLEAGHPGAGFRDLAA............
Collimonas_fungivorans_WP_061540754.1:4-453 REKLF....EAGQPTLPARHPAASYRQ...............
Rhizobium_leguminosarum_WP_116409027.1:2-451 RQKLF....GGGNGRLPAAHPAARFR................
Microvirga_ossetica_WP_099511173.1:1-454 REKLF....GEGRNHLPAEHWGARFRS...............
Pseudomonas_caspiana_WP_087268376.1:4-451 REKLF....PEGGARLPEQHVGASYRS...............
Pseudomonas_lundensis_WP_048375063.1:4-451 RQKLF....TEGDAHLPQRHTGASYRH...............
Caballeronia_mineralivorans_WP_047894899.1:4-456 REKLF....GRG.AKLPDEHTGASFRRL..............
Pseudomonas_paralactis_WP_057702387.1:4-450 REKVF.....HGTARLPEQHTGSSYRH...............
Pseudomonas_taetrolens_WP_048381143.1:4-453 RQKLF....TDGSAHLPDRHTGAAFRHTT.............
Pseudomonas_psychrophila_WP_123750761.1:4-450 RQKLF....VEGDAHLPERHTGASFR................
Collimonas_arenae_100_WP_061532973.1:4-454 REKLF....DTGQATLPARHPAAGFRQL..............
Pseudomonas_viridiflava_WP_122210720.1:4-451 RKKLF....PQGTDRLPPQHAGAGYRQ...............
Kocuria_varians_WP_068470402.1:1-454 RESLY....GAGQDRVKETHPAAAYRG...............
Pseudomonas_wadenswilerensis_WP_115084816.1:4-455 REKLF....ASDQPHLPADHPGASYRHTPTT...........
Pseudomonas_syringae_WP_011266344.1:4-452 RKKLF....PQGTDRLPERHAGAAHRHI..............
Pseudomonas_sp._286_105WP_122415793.1:4-453 RRKLF....PQGTDRLPPQHAGAGYRQGS.............
Geobacillus_vulcani_WP_031406843.1:1-461 REKLF....GNG.PRLSHDHPAAAFRPIGSRT..........
Geobacillus_icigianus_WP_033018365.1:1-461 REKLF....GNG.PRLAHDHPAAAFRPIGSRT..........
Pseudomonas_floridensis_WP_083182568.1:4-453 RKKLF....PQGTDRLPPHHAGAGYRHRS.............
Pseudomonas_asturiensis_WP_073167523.1:4-451 RKKLF....PQGTDRLPPQHAGAGYRQ...............
Geobacillus_vulcani_110_WP_031406843.1:1-461 REKLF....GNG.PRLSHDHPAAAFRPIGSRT..........
```

```
Pseudomonas_syringae_WP_024682626.1:4-452  RKKLF....PQGTDRLPQRHAGAAHRHI................
LadAalpha_Geobacillus_thermoleovorans_BAM76377.1  REKLF....GNG.PRLSHDHPAAAFRPIGSRT............
Tsukamurella_pseudospumae_WP_068746786.1:1-457  RHKLF....GQG.DRLPADHPARRHRYVGA..............
Kocuria_sp._Marseille-P3598_WP_085529073.1:5-456  RENLY....GAGRTSISGDHPAATYRG.................
Microvirga_aerophila_WP_114188789.1:1-456  REKLF....GKGQALLPDTHPGASYRTLGQS.............
Burkholderia_ubonensis_WP_071760483.1:5-455  REKLF....GAG.ARLPAWHAGARHR..................
Achromobacter_marplatensis_WP_006226823.1:4-450  REKLF....GAGRAELPVEHVG...RR.................
Acinetobacter_baumannii_WP_070165746.1:1-443  REKLF....GAG.PYLPENHRGAKYRNL................
Labedella_phragmitis_WP_128496094.1:1-453  RHKLS....GRG.DRLPEEHPGSRHR..................
Nocardia_cyriacigeorgica_120_WP_130918184.1:1-453  RNALF....GAG.DRLPEEHRGARYR..................
Nocardia_cyriacigeorgica_WP_130918184.1:1-453  RNALF....GAG.DRLPEEHRGARYR..................
Mycobacterium_sp._1164985.4_WP_067298521.1:1-453  RHKLL....GNG.DRLPEDHRGSRYR..................
Rhodococcus_sp._1R11_WP_135042739.1:1-449  RHKLF....GKG.DRLPGEHRA......................
Erwinia_amylovora_WP_004157587.1:5-459  REKLF....NQG.ARLAAPHPAAGYRRTTAD.............
Ralstonia_mannitolilytica_WP_102079134.1:4-451  RHKLF....AEG.DRLPARHAAAGFRHL................
Leifsonia_flava_WP_135120808.1:15-465  RQKLH....GRG.DRLPAEHRGAGYR..................
Cryobacterium_roopkundense_WP_084141147.1:1-453  RNKLH....GRG.DRLPTEHRGAGYR..................
Cupriavidus_pauculus_WP_101681197.1:1-455  RDKLF......GTARLPATHAAAQYRGTGAN.............
Rhizobium_sp._BK333_130_WP_133704837.1:2-454  REKLF....GRGDGRLPESHPAAAFRH.................
Caballeronia_mineralivorans_WP_047846789.1:1-454  REKLF....GRG.RLLPAEHPGARYRDIES..............
Nocardia_altamirensis_WP_069163049.1:1-454  RNALF....GAG.DRLPDEHRGARYR..................
Cupriavidus_sp._P-10_WP_116322758.1:1-452  REKLF.....KGSPRLVSPHPGAEYQ..................
Serratia_sp._S1B_WP_116726564.1:1-452  REKLF....GQG.PYLPEDHRGAGYR..................
Pseudomonas_putida_WP_043864435.1:1-452  RNKLF....GAG.DRLGAPHPAAALR..................
Pseudomonas_aeruginosa_WP_023084248.1:4-453  REKLF....GGGRARLQAEHAGAAFRQ.................
Pseudomonas_mucidolens_WP_084381795.1:9-455  REKIF.....TRDAHLPQQHTGATYRH.................
Pseudomonas_poae_WP_060549803.1:4-450  REKVF.....HASARLPKQHTGSTYRH.................
Pseudomonas_chlororaphis_WP_123573412.1:9-454  REKLF.....QREAQLPEQHSGSTYR..................
Janthinobacterium_lividum_140_WP_072454679.1:1-44  RHKVF....GQG.PRLPADH........................
Haladaptatus_cibarius_WP_049970513.1:4-446  RENLV......GRRYLADDH........................
Arthrobacter_luteolus_WP_066298473.1:1-443  REYVG....GAGKPRVADTHPAARYRG.................
Geobacillus_sp._ZGt-1_WP_047758532.1:1-436  REKLF....GNG.PRLSHDHPAAAFRPIGSRT............
Lysinibacillus_composti_WP_124766485.1:4-458  RDNLF....GKG.DRLPNHHPGKQTKVLQLN.............
Microcella_putealis_WP_130486201.1:20-474  REKLH....GRG.DRLPAEHRGAGYRLVPA..............
Pseudomonas_nitroreducens_WP_037016976.1:1-453  RNKLF....GQG.DRLGASHRAAKLR..................
Acinetobacter_wuhouensis_WP_130168784.1:1-453  REKLF....GAG.AHLPEYHRGAQFRH.................
Bradyrhizobium_sp._YR681_WP_008144663.1:1-454  REKLF....GDGRARLDAPHPAAGYR..................
Schumannella_sp._10F1D-1_WP_141164655.1:10-460  RQKLH....GRG.DRLPEEHKGASFR..................
Pseudomonas_coronafaciens_150_WP_122355187.1:4-45  RKKLF....PEGTDRLPERHAGAAHRHI................
Pseudomonas_syringae_WP_003430238.1:4-452  RKKLF....PQGTDHLPQRHAGAAHRHI................
unclassified_Rhodococcus_WP_094682261.1:1-449  RHKLF....GNG.DRLPEEHRA......................
Hafnia_alvei_WP_046449330.1:6-449  RHKLF....GQG.AQLTALHPAAKRR..................
AS9A_3890_Hoyosella_subflava_WP_013808677.1:1-453  REKLF....GNG.ARLPDDHRGARYR..................
Acinetobacter_baumannii_WP_000995600.1:1-443  REKLF....GAG.PYLPENHRGAKYRNL................
Agrobacterium_sp._B133/95_WP_065696417.1:2-454  RQKLF....GRGDGRLPSSHPAAAFRH.................
Microbacterium_sp._KROCY2_WP_024289149.1:1-453  RENVY....GAGQTKVLDTHPAAKYR..................
Variovorax_paradoxus_WP_012747040.1:6-452  RRKLF....DAG.DRLPARHAAAQFRH.................
Methylobacterium_radiotolerans_WP_076730229.1:5-4  RAKLF....GQG.DRLPARHPAEAYRG.................
Streptomyces_sp._BK215_WP_132915265.1:7-452  REKLL....PGGGPRLPERHPAAAHR..................
Geobacillus_thermodenitrificans_160_WP_008880416.  REKLF.......................................
Pseudomonas_aeruginosa_WP_132556951.1:356-785  REKLF....GGGRARLQAEHAGAAFRQ.................
Penicillium_solitum_OQE02104.1:14-471  RENLY...GQPGQSGPHADHPASKYR..................
Penicillium_vulpinum_OQE05502.1:12-469  RENLY...AQPGQSGPHADHPAYKYR..................
Clavibacter_michiganensis_WP_045526194.1:1-454  RGRLN....GTGSPVVPEWHPAHAYRG.................
Glutamicibacter_creatinolyticus_WP_054821484.1:6-  RNKLF....GAG.DRVKDTHRAAQYK..................
Campylobacter_sp._P0078_WP_086237522.1:4-454  RNKLF....DKG.DRLSSDHIGSKYR..................
Pseudomonas_furukawaii_WP_003454569.1:1-447  RHKLF....GQG.PRLAAPHPAAHWRQ.................
Leucobacter_triazinivorans_WP_130111548.1:1-442  RDKLF....GAG.DRLPDDHRGAGYR..................
Aspergillus_brasiliensis_CBS_101740_OJJ68286.1:12  RENLY...GKEGQSGPPADHPAAKYR..................
Aspergillus_udagawae_170_GAO89440.1:15-472  RENLY...GKPGQTGPPEDHPAAKYR..................
Aspergillus_flavus_RMZ37351.1:15-472  RENLY...GKPGQSGPPEDHPAAKYR..................
Aspergillus_flavus_RAQ54081.1:15-472  RENLY...GRPGQSGPPADHPAAKYR..................
Penicillium_arizonense_XP_022490779.1:12-469  RENLY...SEPGQSGPRADHPASKYR..................
Salinibacterium_sp._CGMCC  RNKLH....GRG.DRLPAEHKGASFK..................
Aspergillus_fumigatus_OXN06785.1:14-471  RENLY...GKPGQTGPPADHPASRYR..................
Aspergillus_lentulus_GAQ10210.1:7-464  RENLY...GKPGQTGPLEDHPASRYR..................
Aspergillus_flavus_RAQ66103.1:15-472  RENLY...GKPGQSGPPEDHPAAKYR..................
Aspergillus_fumigatus_OXN22894.1:14-471  RENLY...GKPGQTGPPADHPASRYR..................
Aspergillus_nidulans  RENLY...GKPGQSGPPADHPAAKYR..................
Penicillium_nalgiovense_OQE79848.1:14-471  RENLY...TQPGQSGPRADHPASKYR..................
Aspergillus_wentii  REAYI......GSRRLPEDHPGSKYK..................
Gordonia_iterans_WP_105943624.1:1-453  RHKLF....GNG.DRLPGAHRGAGFR..................
Azotobacter_beijerinckii_WP_090735436.1:4-454  REKLF.....AAGPRLPASHPGAAWRDLS...............
Staphylococcus_sp._NAM3COL9_WP_057513692.1:4-453  RENMF....GKDKKTIKENHPAKNKR..................
Acidovorax_sp._56_WP_099656603.1:11-450  RHKLF....GKG.NRLPDQH........................
Paenibacillus_sp._BIHB4019_WP_099518466.1:1-456  RERLF....GKGEARLPEHHPGASFRTPAALV............
Penicillium_antarcticum_OQD90033.1:5-465  RENMY...GKKGQARLPDAHPGAKY...................
Aspergillus_oryzae_RIB40_190_XP_001823703.1:9-463  REVFI......GSRRLPEDHPGSKY...................
Fusarium_oxysporum_f._sp._pisi  RENVY...GRKGVARPPADHPAAKY...................
Colletotrichum_fructicola_Nara_gc5_ELA35345.1:18-  REVFI......GSRRLPEDHPGSKYK..................
Micrococcus_luteus_WP_041104460.1:19-468  RHKLF....GRG.DHLPQNHHGASF...................
Aspergillus_awamori_GCB21923.1:12-469  RENLY...SKEGQSGPPADHPAAKYR..................
Aspergillus_niger_CBS  RENLY...SKEGQSGPPADHPAAKYR..................
Aspergillus_lacticoffeatus_CBS_101883_XP_02545389  RENLY...SKEGQSGPPADHPAAKYR..................
Aspergillus_wentii_DTO_134E9_OJJ40237.1:14-471  RENLY...GKAGQSGPPSDHPASRYR..................
Aspergillus_indologenus_CBS_114.80_PYI31207.1:11-  RENLY...GKKGQAGPPEDHPAAKYR..................
Aspergillus_fischeri_NRRL_181_XP_001264002.1:14-4  RENLY...GKPGQRGPPVDHPASKYR..................
Penicillium_coprophilum_200_OQE46388.1:11-466  REVFI......GSQRLPADHPGSKYK..................
Cupriavidus_taiwanensis_WP_116379876.1:8-454  RHKLF....AEG.DRLPARHAADAFRH.................
Penicillium_digitatum  RENMY...GKQGQSRLPDTHPGAKY...................
Aspergillus_niger_GAQ46262.1:12-469  RENIY...GKEGQSGPPADHPAAKYR..................
Aspergillus_nomius_NRRL_13137_XP_015412385.1:15-4  RENLY...GQPGQSGPPTDHPAAKYR..................
Aspergillus_aculeatus_ATCC_16872_XP_020055010.1:1  RENLY...GRRGQTGPPEGHPAAKYR..................
Penicillium_brasilianum_OOQ84969.1:19-474  RE.LF.....IGSKRLPEDHPGSKYK..................
Penicillium_subrubescens_OKP15100.1:19-474  REVFI......GSKRLPEDHPGSKYK..................
Trichoderma_arundinaceum_RFU80530.1:19-474  REVFI......GSKRLPEDHPGSLYK..................
Aspergillus_thermomutatus_210_XP_026614594.1:15-4  RENLY...GKPGQTGPPEDHPAARYR..................
Serratia_liquefaciens_WP_044551273.1:4-449  RQKLF.....QQQDRLPPSHPAARWR..................
Micrococcus_luteus_WP_065572847.1:19-468  RHKLF....GRG.DHLPQNHHGASF...................
Halolamina_rubra_WP_049979911.1:1-449  RERLY....GEGQRRLPDHHPA......................
Acinetobacter_pittii_WP_075382847.1:7-452  RNKIF....NRG.DQLPKQHPVQAFR..................
Corynebacterium_variabile_WP_052302534.1:2-464  REKLFADRTGAG.PLVPHNHSAARYRPAST..............
Aspergillus_luchuensis_GAT21254.1:12-471  RENIY...GKEGQSGPPADHPAAKYR..................
```

```
Aspergillus_luchuensis                             RENIY...GKEGQSGPPADHPAAKYR................
Aspergillus_bombycis_XP_022386026.1:15-472         RENLY...GTPGQSGPPTDHPAAKYR................
Agrobacterium_vitis_WP_070163524.1:1-454           REKLF....GQG.PYLPQNHPADQYRDIEA............
Pseudomonas_psychrotolerans_220_WP_074584491.1:5-  RHKLF....GRG.DHLDTRHFGSRFRQ...............
Micrococcus_yunnanensis_WP_135040276.1:19-468      RHKLF....GRG.DHLPQNHHGASF.................
Acinetobacter_calcoaceticus_WP_004642549.1:7-452   RNKIF....NRG.DQLPKQHPVQAFR................
Penicillium_brasilianum_CEO59775.1:15-472          RENLY...STPGQAGPRADHPASKYR................
Aspergillus_cristatus_ODM22551.1:12-469            RENVY...RKKGQSGPPADHPASKYR................
Pseudomonas_taiwanensis_WP_027907363.1:1-446       RHKLF....GQG.AHLPGAH......................
Acidovorax_avenae                                  RHKLA....GRG.DRLAPTHHGA...................
Staphylococcus_pseudintermedius_WP_101431388.1:4-  RENMF....GKGHTKVKPTHPA....................
Rhodococcus_triatomae_WP_081607437.1:2-451         RGNVT....GRG.ARLGDGHVGASFR................
Alcaligenes_sp._RS4_230_WP_128393375.1:4-450       RHKLF....ERG.ATLSSSHFASQWRH...............
Gibbsiella_quercinecans_WP_121552947.1:7-454       RKKLF.....QQQDCLPASHPAARWRTPAA............
Acinetobacter_ursingii_WP_044435211.1:5-452        RHKLH....QQG.DRLAASHPADAFR................
Erwinia_typographi_WP_034887712.1:4-447            RKKIF.....QQQDRLPERHPAARWR................
Trichoderma_harzianum_KKP01913.1:14-470            RENLY...AKKGLARPPADHPASKY.................
Fusarium_graminearum_235_CEF79766.1:12-466         RENFY...GIPGQKYPLEEHVASSH.................
Gordonia_alkanivorans_WP_006357654.1:10-461        RHKLF....GRG.DHLPENHRGSTYR................
Acinetobacter_radioresistens_WP_111281495.1:5-455  RHKLF....QQG.NLLSAQHPVQQFRCLT.............
Mesorhizobium_plurifarium_WP_073985566.1:5-458     RERFY....GEGHLRLPADHPASAYRRLA.............
Oceanobacillus_halophilus_WP_121203049.1:1-454     RENLF....ETGNPHLPDNHPARKRKSL..............
Acinetobacter_tandoii_240_WP_100241535.1:7-457     RHKLF....QQG.DHLAAQHPVQQFRCLSST...........
Burkholderia_plantarii_WP_042624083.1:1-457        REKLL....GGG.ARLSAPHVGARFRAAGAD...........
Alcaligenes_faecalis_WP_094195208.1:6-457          RQQLF....GQG.DRLSATHPAAQWRHPASG...........
Bradyrhizobium_arachidis_WP_092214516.1:1-453      REKIF....GPGQARLPSSHPAARFRH...............
Cupriavidus_plantarum_WP_109583068.1:1-458         REKLTGTEAQPASATLQAPHVGAAFRR...............
Variovorax_sp._770b2_245_WP_093443018.1:5-461      REKIF....GTGQARLPSTHAGAQWRDLSQQT.........
RZJ22128.1:4-447                                   RENLG........LKRPENRHVLAR.................
LadB_Geobacillus_thermoleovorans_BAM76371.1        .EVWI.....................................
LadAbeta_Geobacillus_thermoleovorans_BAM76372.1    RDHLG..........LPVPKSIRV..................
Af1_AFLA_024140_XP_002373925.1                     RENLY...GKPGQSGPPDDHPAAKYRWNAGVDAEEHKIPDN.
Af3_AFLA_58870_XP_002378631.1                      RENMY...GEKGQARLPQSHPGAKYVWHAGEETPKYALEKNT
Af4_AFLA_049780_XP_002383105.1                     REKVY....GKGQRELRADHPGSQYKYEVYQEEAAVAETEVS
Af5_AFLA_126060_XP_002381799.1                     RENYF..QDGLG.SRLREGHPGREYTWN..............
Af2_QMW32876.1:9-463                               REVFI......GSRRLPEDHPGSKY.................
Yarrowia_lipolytica_CLIB122_YALI0C07546p_XP_50156  RELLY...DTPGNSYLRSDH......................
Rhizobium_sp._CF122_WP007794079.1                  RENL......................................
Acinetobacter_sp._NIPH_2100_LLM                    RENL.......GLARPENQYVLAR..................
Nostoc_sp._PCC_7107_WP_015115963.1                 RENLG........LRRPDNQFVVQ..................
Aspergillus_flavus_NRRL3357_uncharacterized        REHL.......GLPRPNNRFFS....................
Paenibacillus_curdlanolyticus_WP006037998.1        RENLG........LRRPADYTA....................
LadAbeta_Geobacillus_thermoleovorans_FMN-dependen  RDHL......................................
SsuD_1M41                                          PQPLN....PQGEAVANDFIPRKVAQS...............
Paenibacillus_curdlanolyticus_WP006037998.1        RENLG........LRRPADYTA....................
Bacillus                                           ..........................................
Pseudomonas                                        ..........................................
LadB_Geobacillus_thermoleovorans_FMN-dependent     ..........................................
Pseudomonas_aeruginosa_PAO1_NP_251290.1            RLPLL.............RHAG....................
Alkanesulfonate                                    ..........................................
Alkanesulfonate                                    QER..............GAQPTA...................
Aspergillus_niger_ATCC13496_RDH19503.1             RENSN.....................................
Penicillium_digitatum_Pd1_XP014534980.1            KER...............ENDASK..................
Acinetobacter_rudis_WP_016657872.1                 TGPFG...EMIANDVVPQKLAGQS..................
```
