# Supplementary material for: Identification of long-chain alkane-degrading (LadA) monooxygenases in Aspergillus flavus via in silico analysis
Source: Front Microbiol. 2022 Aug 30;13:898456. doi: 10.3389/fmicb.2022.898456 (PMC9468676; doi:10.3389/fmicb.2022.898456)

Supplementary Figure 2. Maximum Likelihood phylogenetic tree with GenBank accession numbers correspond to all leaf labels in Figure 2

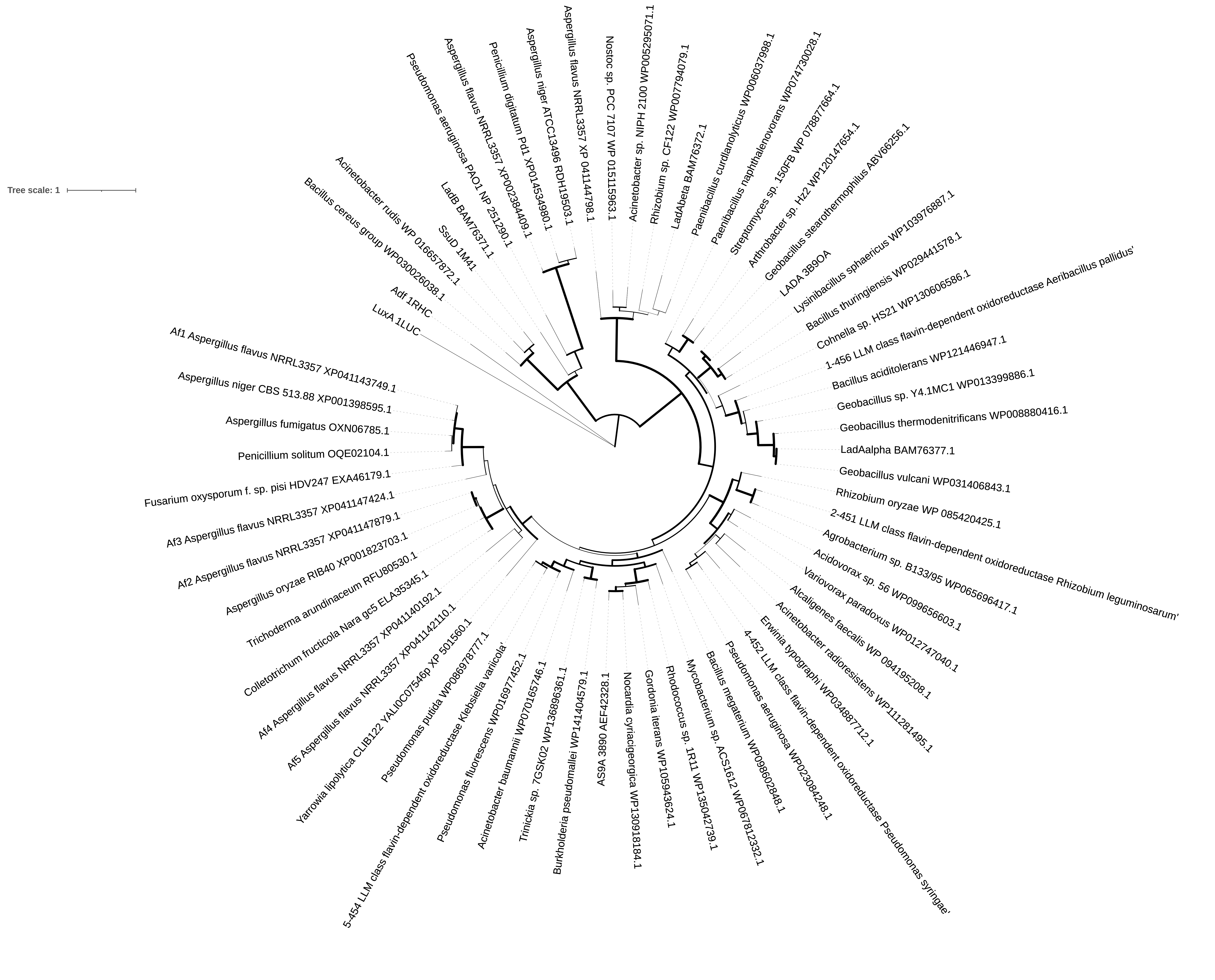

Supplementary Figure 2B. Similarity matrix of the A representative sample (n=63) of the 260 derived protein sequences of the identified LadA-like FMN-dependent monooxygenases in bacteria and fungi

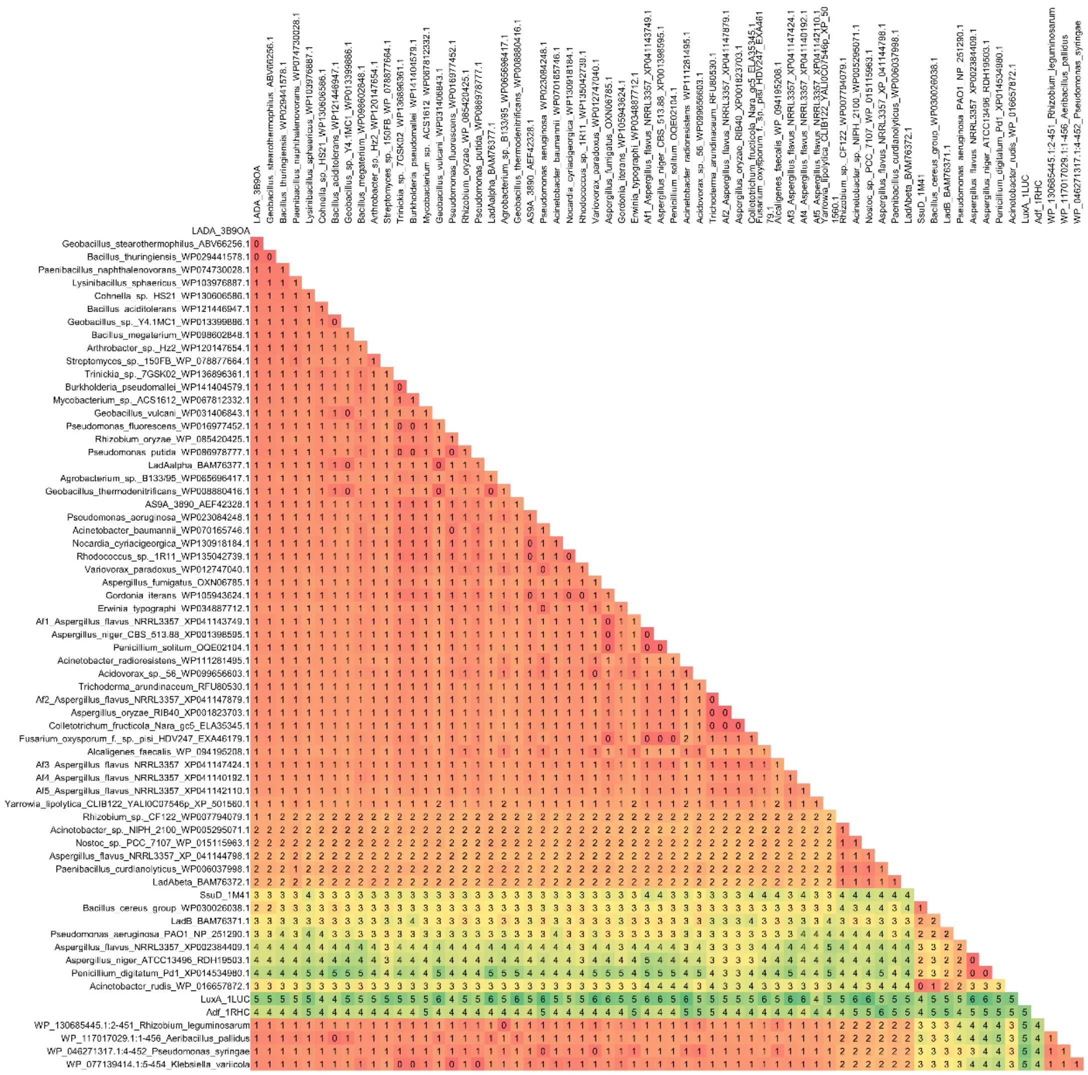

Supplement: Supplementary file 3 [file Image_2.pdf]
