# Supplementary material for: Identification of long-chain alkane-degrading (LadA) monooxygenases in Aspergillus flavus via in silico analysis
Source: Front Microbiol. 2022 Aug 30;13:898456. doi: 10.3389/fmicb.2022.898456 (PMC9468676; doi:10.3389/fmicb.2022.898456)

Supplementary Figure 3. Secondary structures of A. flavus LadAα homologs obtained by PSIPRED 4.0

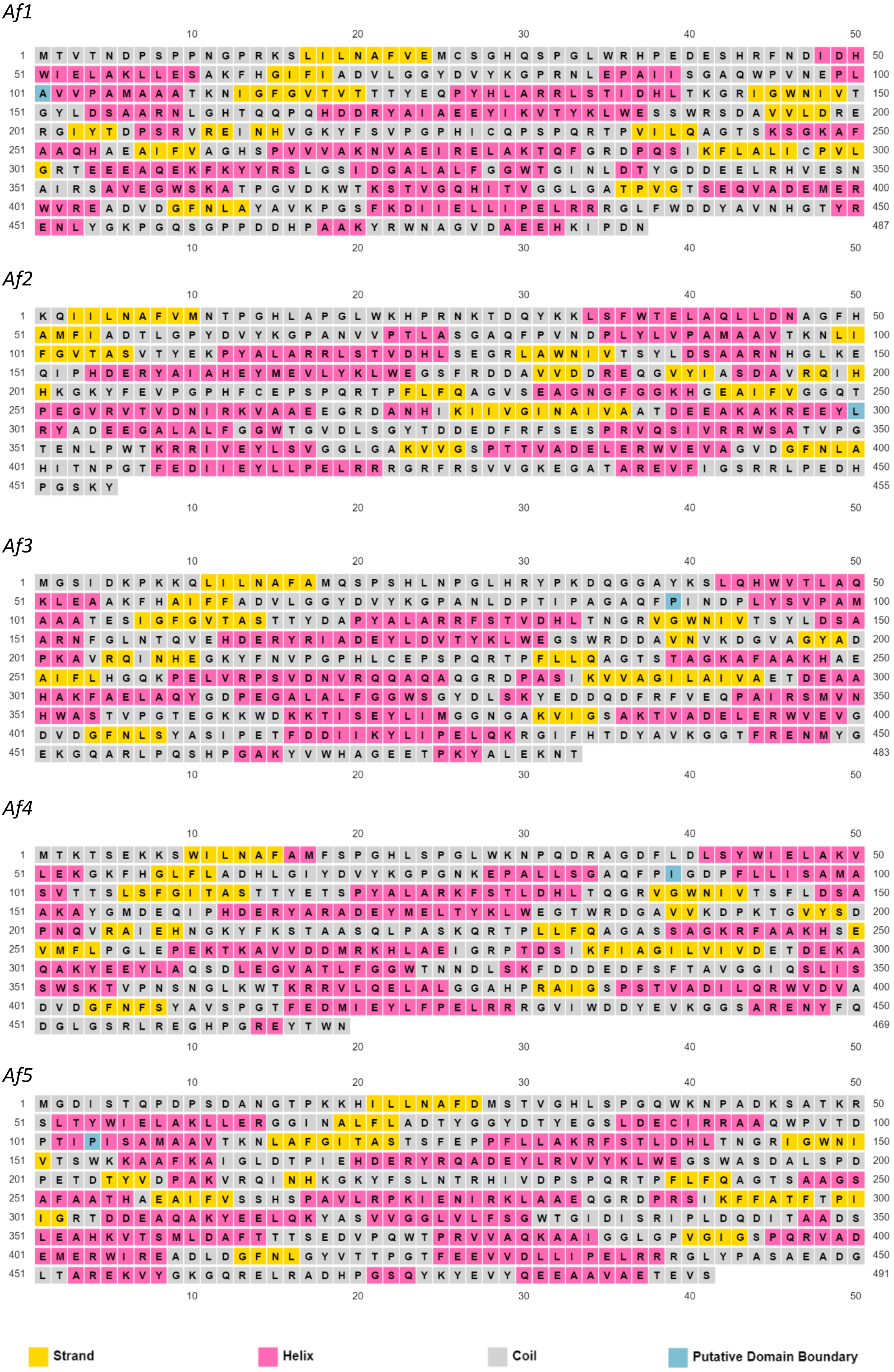

Supplement: Supplementary file 4 [file Image_3.pdf]
