# Supplementary material for: Identification of long-chain alkane-degrading (LadA) monooxygenases in Aspergillus flavus via in silico analysis
Source: Front Microbiol. 2022 Aug 30;13:898456. doi: 10.3389/fmicb.2022.898456 (PMC9468676; doi:10.3389/fmicb.2022.898456)

Supplementary Figure 4. Comparative modelling of LadAα homologs in *A. flavus.* **(A)** Three-dimensional models of LadAα homologs in *A. flavus*, predicted by SWISS-MODEL (Benkert, Biasini and Schwede, 2011; Bertoni *et al.*, 2017; Waterhouse *et al.*, 2018); two hair-pin loop at the top of each model is indicated in green and the large bulge is indicated in greenish-yellow. **(B)** Statistics of 3-dimensional model quality evaluated by PROCHECK (Laskowski *et al.*, 1993), VERIFY3D (Einsenberg, Luthy and Bowei, 1997), ProSA(Wiederstein and Sippl, 2007) and ERRAT(Colovos and Yeates, 1993) protein structure validation tools. **(C)**, Superpose of models *Af1* to *Af5* with *G. thermodenitrificans* LadA (3B9O chain A) crystal structure (Li *et al.*, 2008) (cyan)

A

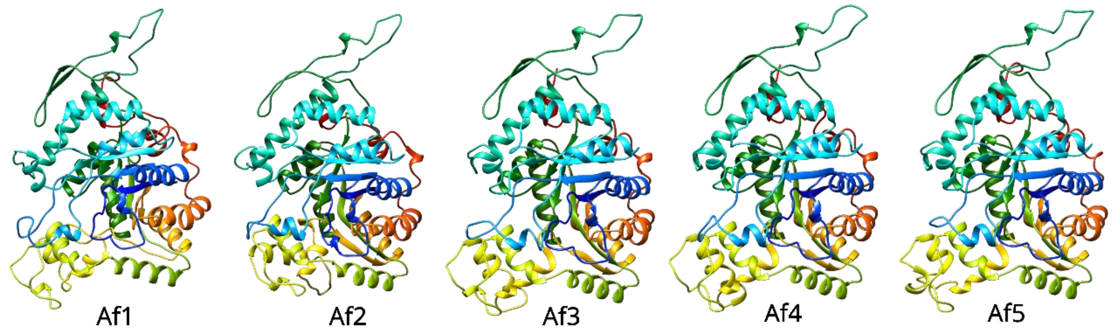

B

Aspergillus LadA homolog	PROCHECK				VERIFY3D	ProSA	ERRAT
	Ramachandran plot statistics (%)				Compatibility score (%)	z-score	
	Core	Allowed	General	Disallowed			
Af1	90.3%	8.4%	0.8%	0.5%	94.37%	-9.01	84.547
Af2	91.5%	7.8%	0.3%	0.5%	96.04%	-9.39	89.888
Af3	91.3%	7.2%	1.0%	0.5%	92.61%	-9.2	91.629
Af4	91.2%	7.6%	0.8%	0.5%	90.02%	-9.37	87.585
Af5	92.0%	6.3%	1.0%	0.8%	94.79%	-9.03	91.556

C

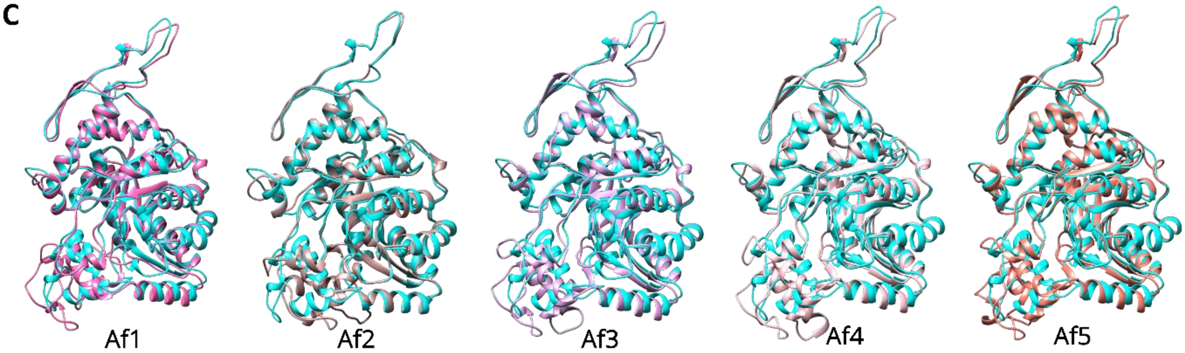

Supplement: Supplementary file 5 [file Image_4.pdf]
