# Supplementary material for: Identification of long-chain alkane-degrading (LadA) monooxygenases in Aspergillus flavus via in silico analysis
Source: Front Microbiol. 2022 Aug 30;13:898456. doi: 10.3389/fmicb.2022.898456 (PMC9468676; doi:10.3389/fmicb.2022.898456)

Supplementary Figure 5. Re-docking analysis of *G. thermodentrificans* LadA (3B9O) (Li *et al.*, 2008) with FMN and hexadecane for the validation of the docking method. **(A)** Comparison of confirmations of FMN; FMN in docked 3B9O_A: FMNopt complex (cyan) with FMN in crystal structure of LadA: FMN complex (gold). **(B)** Reaction scheme of the FMN and the alkane with atom labels **(C)** Active pocket residues of the re-docked pose of 3B9O_A with FMNopt and hexadecane (dark brown) as viewed by UCSF chimera and BIOVIA Discovery Studio Visualizer; residues within 5 Å around the bound ligands surfacing the active pocket are labelled in red lettering while, other residues inside the active pocket are in black, Tyr63 and Gln79 found outside the pocket (> 5 Å) are also labelled only to highlight their position for reference

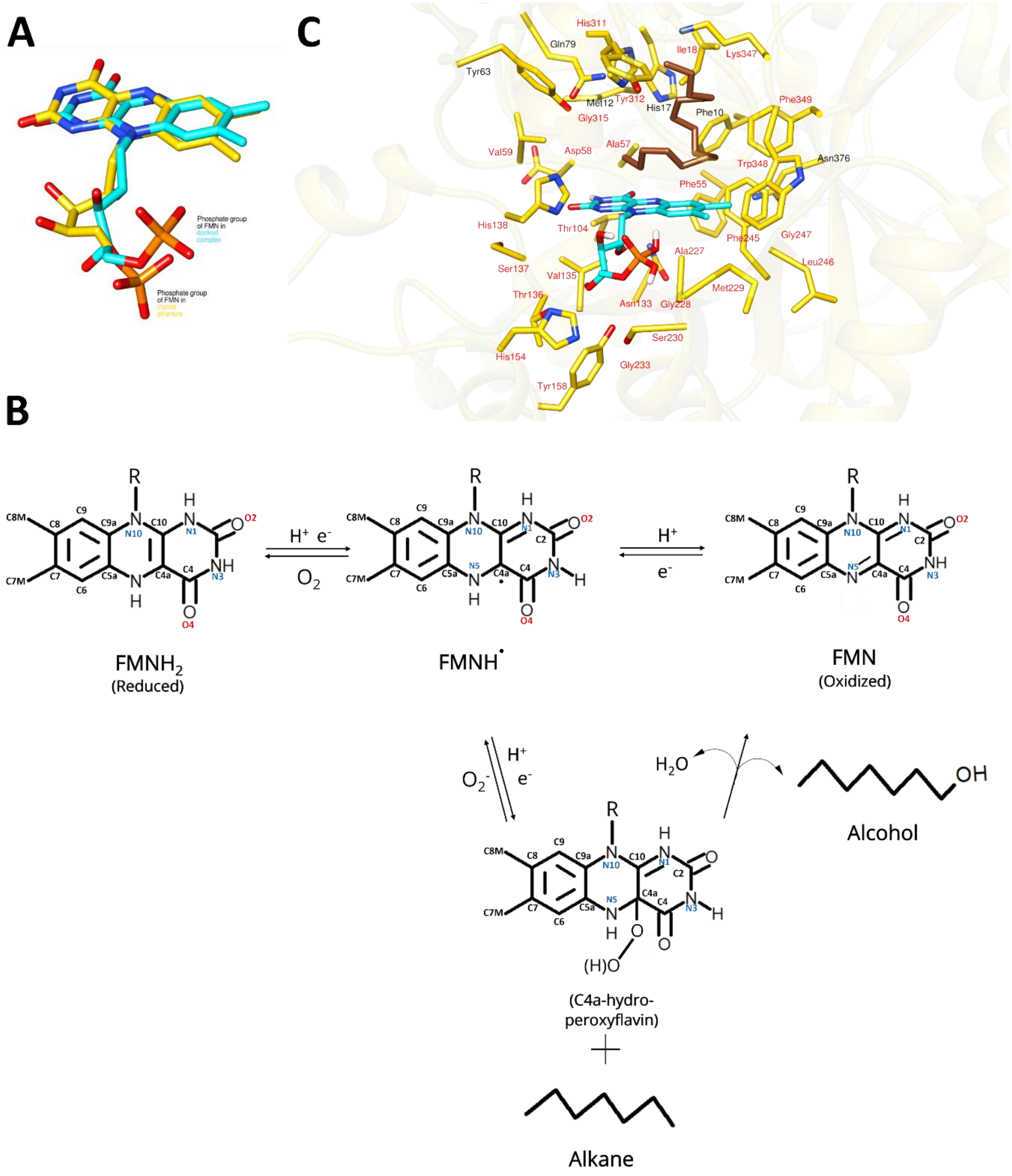

Supplement: Supplementary file 6 [file Image_5.pdf]
