# Supplementary material for: Identification of long-chain alkane-degrading (LadA) monooxygenases in Aspergillus flavus via in silico analysis
Source: Front Microbiol. 2022 Aug 30;13:898456. doi: 10.3389/fmicb.2022.898456 (PMC9468676; doi:10.3389/fmicb.2022.898456)

Supplementary Figure 6. The pocket residues of 3B90_A: FMNopt: alkane (C17-C30) complexes visualized by BIOVIADiscovery Studio Visualizer; amino acid residues are found within 5 Å around the bound ligands inside the active pocket except for Tyr63 and Gln79. The distance between the terminal / subterminal carbon of the alkane with the pi-electron cloud of the FMN is <= 5 Å. Alkane (dark brown), FMN (green) and amino acid residues (gray). (Note: since the crystal structure of 3B90 chain A (3B90_A) starts from Lys (3rd residue in the complete sequence), Biovia Discovery studio Visualizer displays the amino acid positions concurrently numbered considering the position of Lys as the 1st residue. As such a difference of two residue numbers are observed between 3B9O complete sequence and 3B9O_A; e.g: Tyr158 is identified as Tyr156 in 3B9O_And this figure; e.g: Tyr158 is ideintified as Tyr156 in 3B9O_A.

3B9O_A: FMN: C17

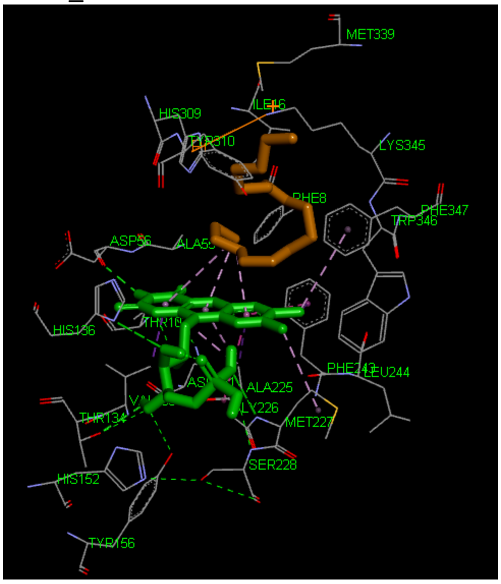

3B9O_A: FMN: C18

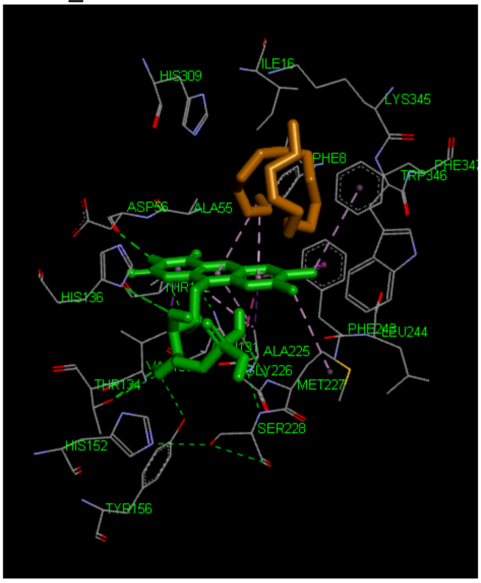

3B9O_A: FMN: C19

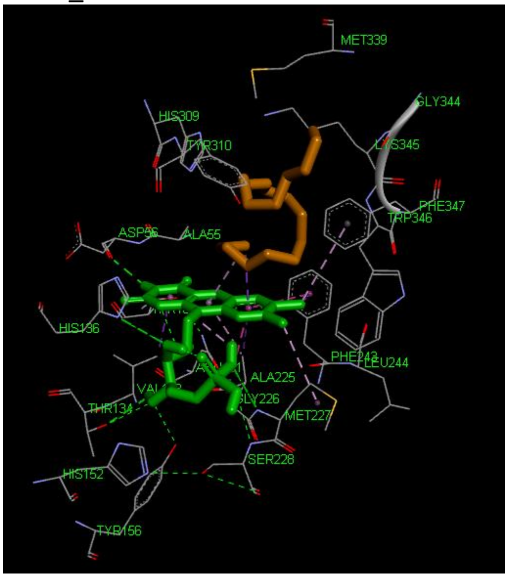

3B9O_A: FMN: C20

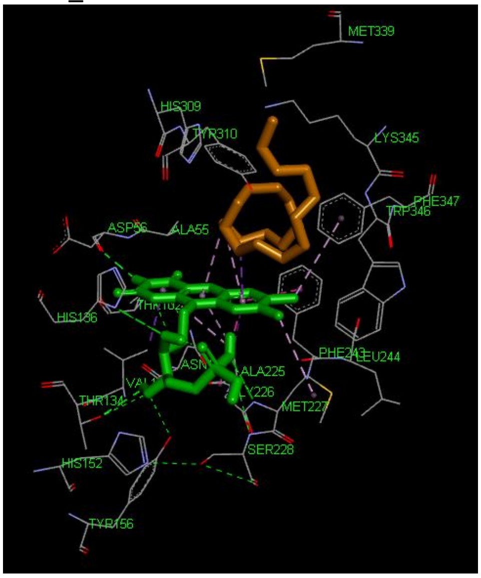

3B9O_A: FMN: C21

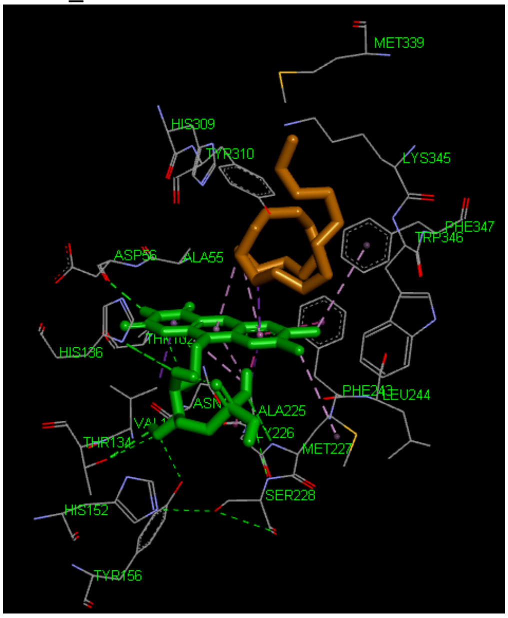

3B9O_A: FMN: C22

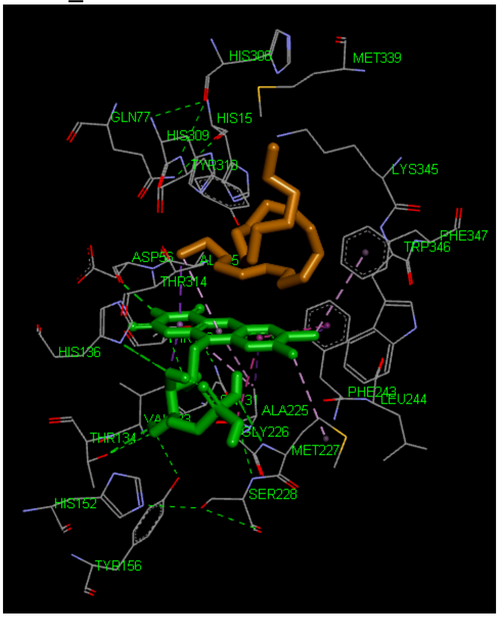

3B9O_A: FMN: C23

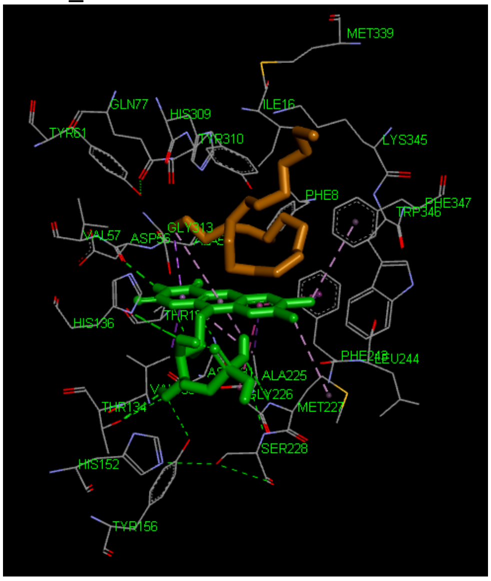

3B9O_A: FMN: C24

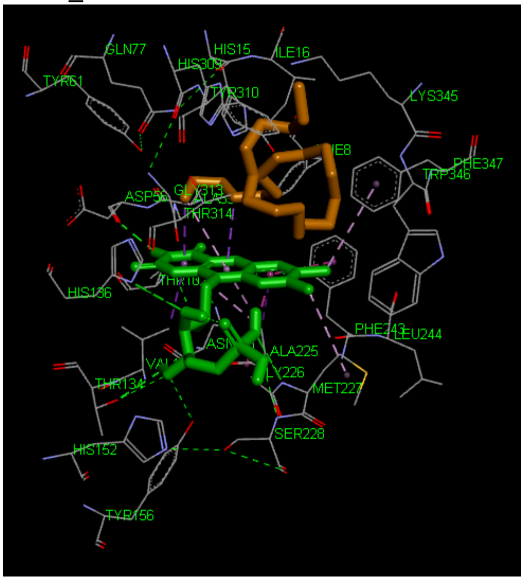

3B9O_A: FMN: C25

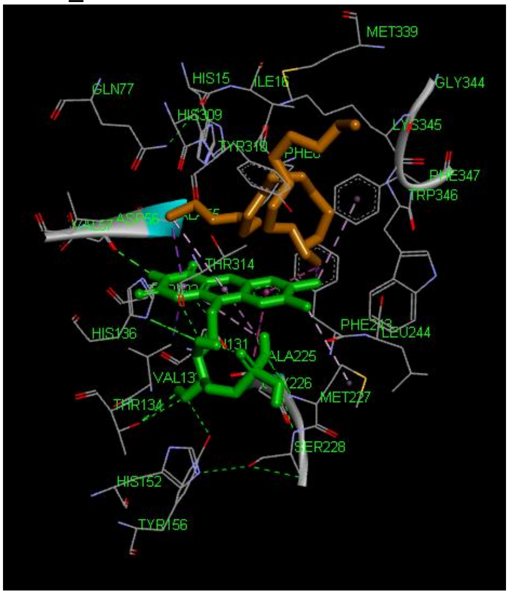

3B9O_A: FMN: C26

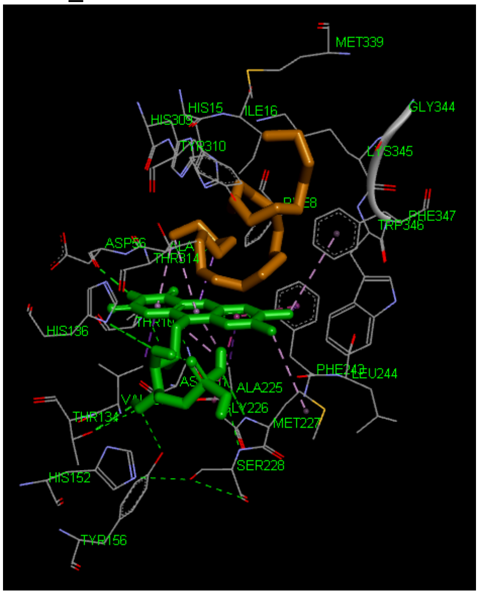

3B9O_A: FMN: C27

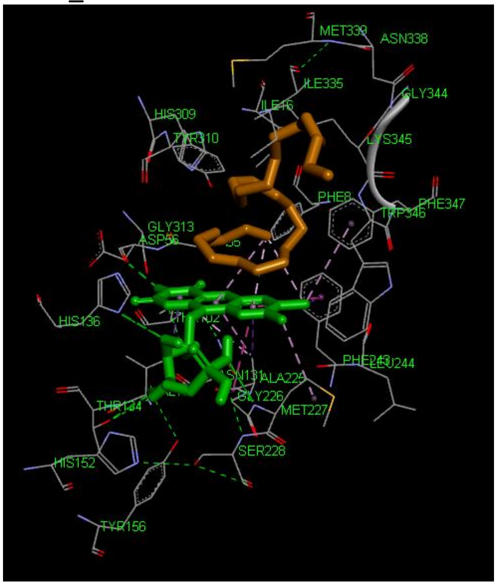

3B9O_A: FMN: C28

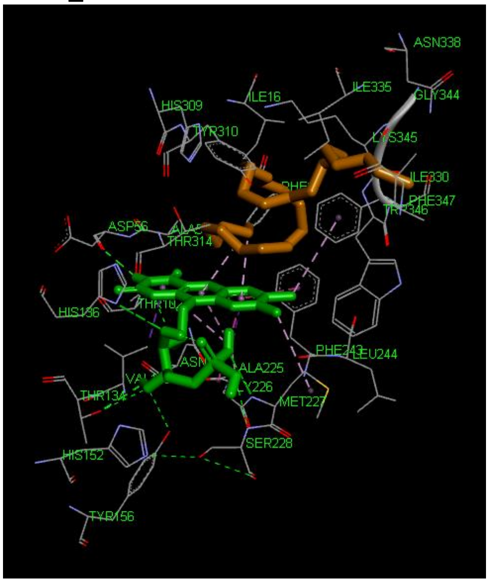

3B9O_A: FMN: C29

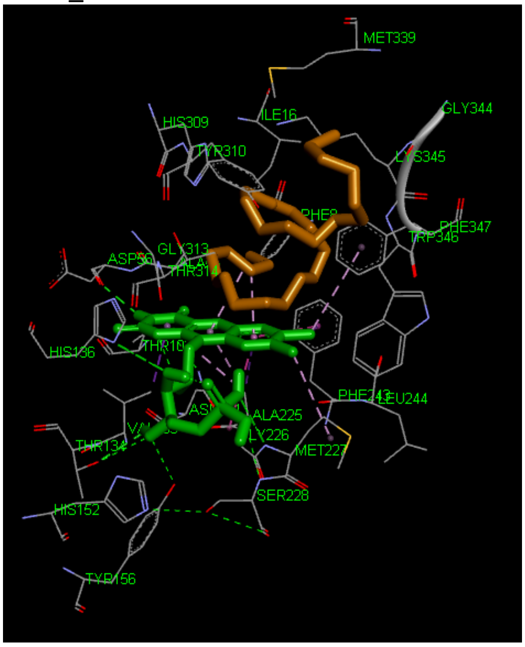

3B9O_A: FMN: C30

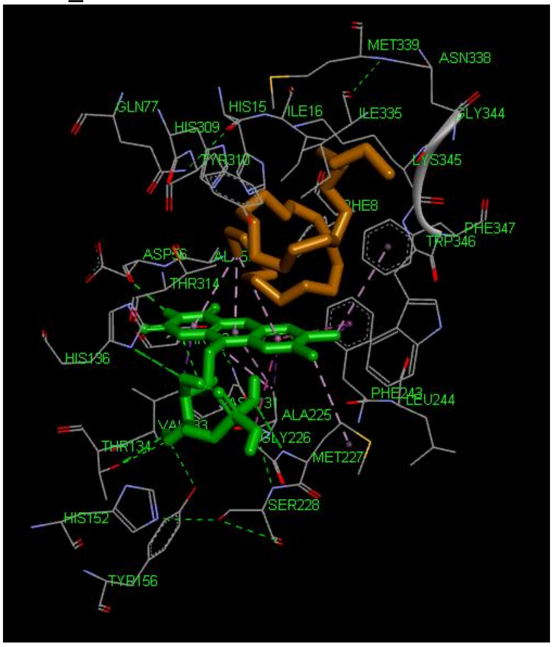

Supplement: Supplementary file 7 [file Image_6.pdf]
