# Supplementary material for: Identification of long-chain alkane-degrading (LadA) monooxygenases in Aspergillus flavus via in silico analysis
Source: Front Microbiol. 2022 Aug 30;13:898456. doi: 10.3389/fmicb.2022.898456 (PMC9468676; doi:10.3389/fmicb.2022.898456)

Supplementary Figure 8. The pocket residues of Af3: FMNopt: alkane (C17-C30) complexes visualized by BIOVIA Discovery Studio Visualizer; amino acid residues are found within 5 Å around the bound ligands inside the active pocket except for Tyr63 and Gln79. The distance between the terminal / subterminal carbon of the alkane with the pi-electron cloud of the FMN is <= 5 Å. Alkane (dark brown), FMN (green) and amino acid residues (gray).

Af3: FMN: C17

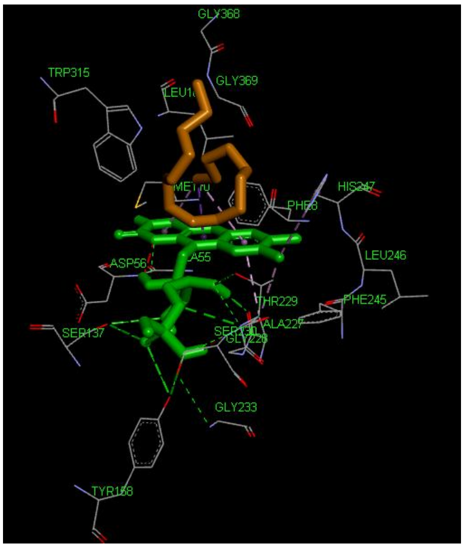


Af3: FMN: C18

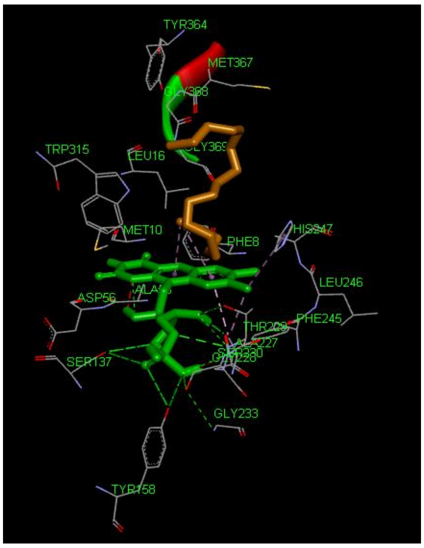


Af3: FMN: C19

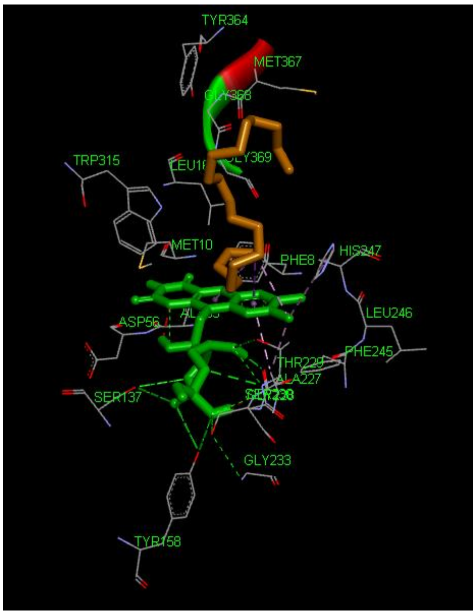


Af3: FMN: C20

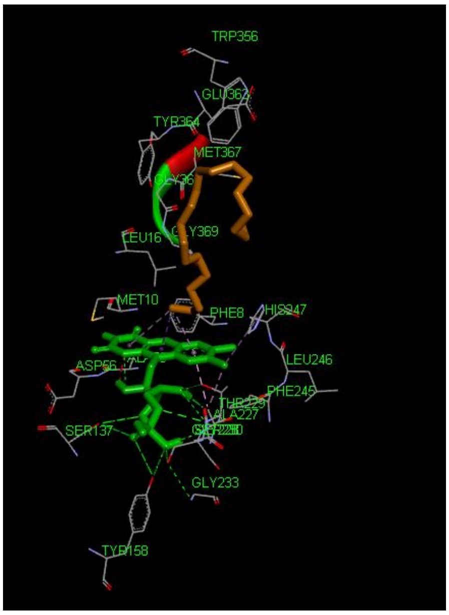


Af3: FMN: C21

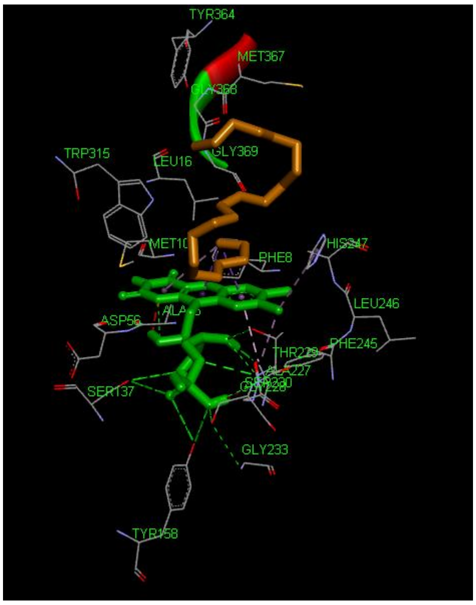


Af3: FMN: C22

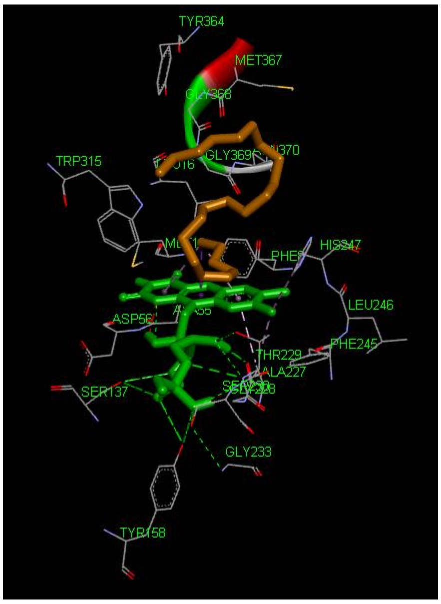

Af3: FMN: C23

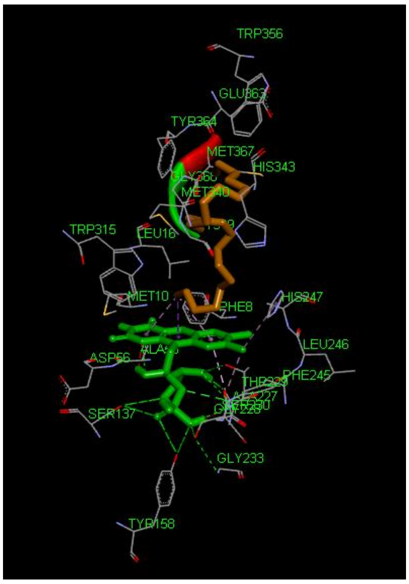


Af3: FMN: C24

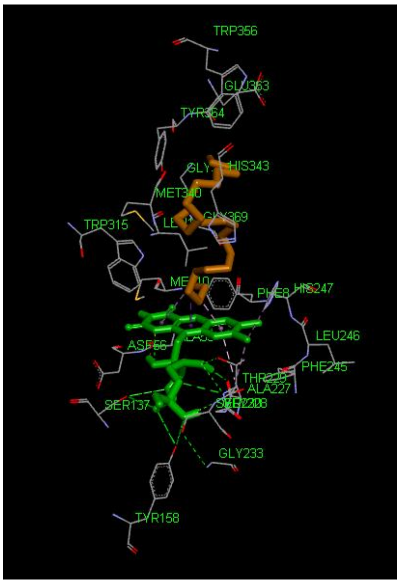


Af3: FMN: C25

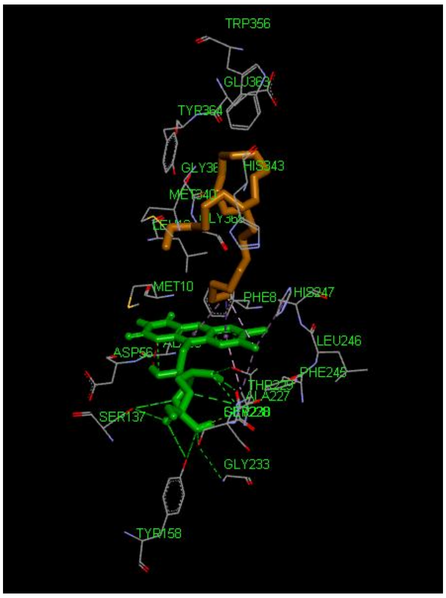


Af3: FMN: C26

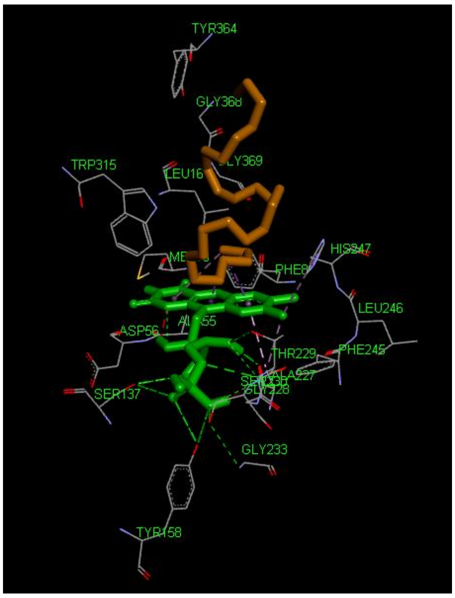


Af3: FMN: C27

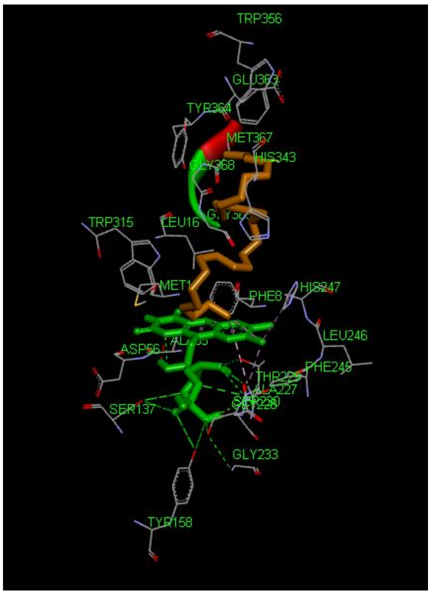


Af3: FMN: C28

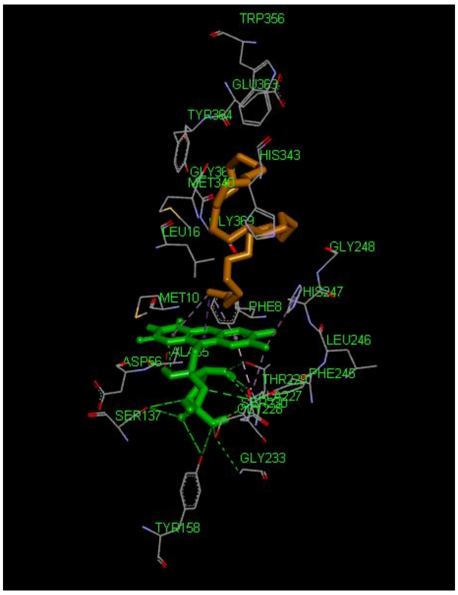

Af3: FMN: C29

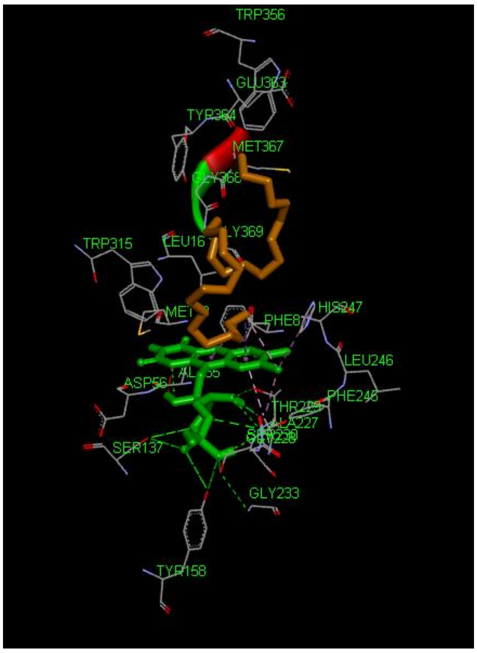


Af3: FMN: C30

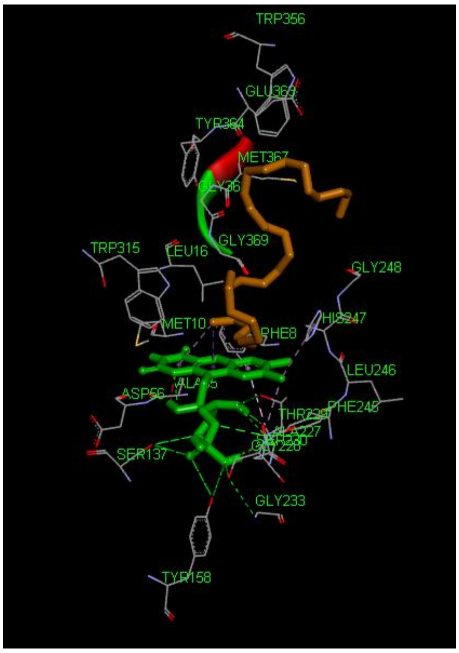

Supplement: Supplementary file 9 [file Image_8.pdf]
