# Supplementary material for: Identification of long-chain alkane-degrading (LadA) monooxygenases in Aspergillus flavus via in silico analysis
Source: Front Microbiol. 2022 Aug 30;13:898456. doi: 10.3389/fmicb.2022.898456 (PMC9468676; doi:10.3389/fmicb.2022.898456)

Supplementary Figure 9. The pocket residues of Af4: FMNopt: alkane (C17-C30) complexes visualized by BIOVIA Discovery Studio Visualizer; amino acid residues are found within 5 Å around the bound ligands inside the active pocket except for Tyr63 and Gln79 that are found outside the pocket (> 5 Å) and the distance between the terminal / subterminal carbon of the alkane with the pi-electron cloud of the FMN is <= 5 Å, alkane (drak brown), FMN (green), amino acid residues (gray) and labels (green) (Note: actual residue numbers differs from the reference amino acid positions due to insertions / deletions; e.g: Tyr158 in text is displayed as Tyr156 in this figure)

Af4: FMN: C17

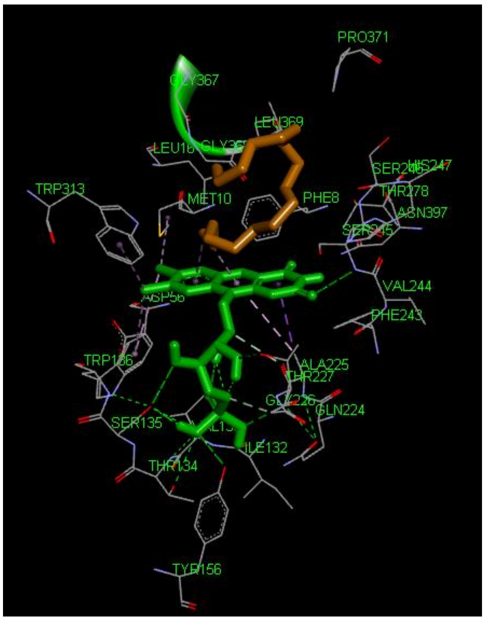

Af4: FMN: C18

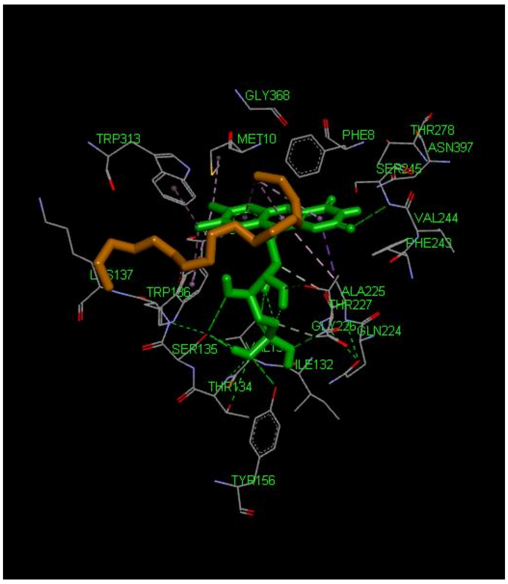

Af4: FMN: C19

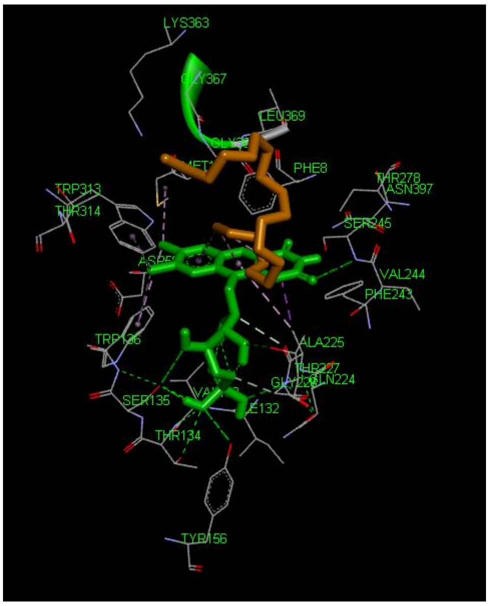

Af4: FMN: C20

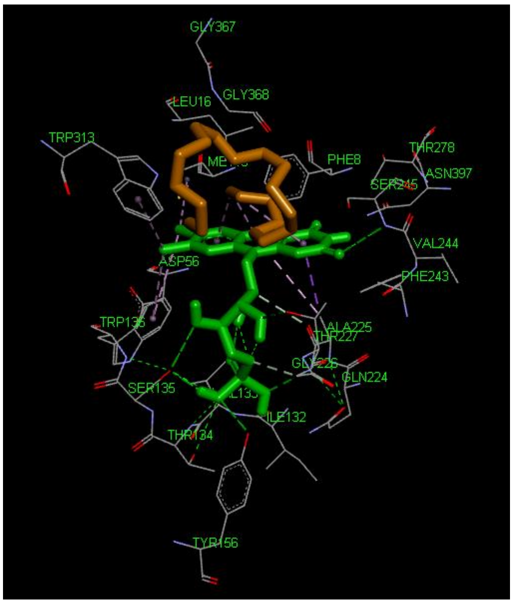

Af4: FMN: C21

Af4: FMN: C22

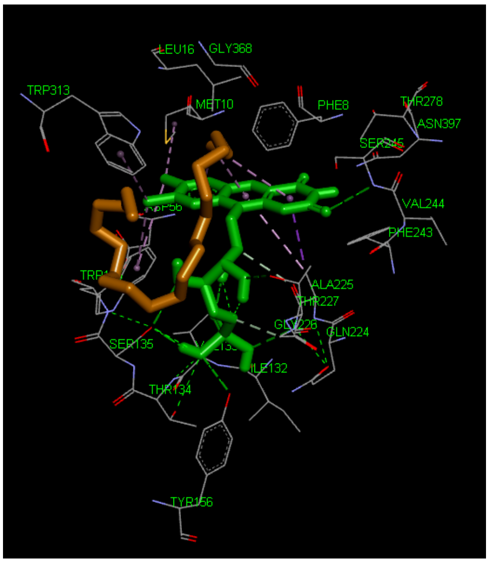

Af4: FMN: C23

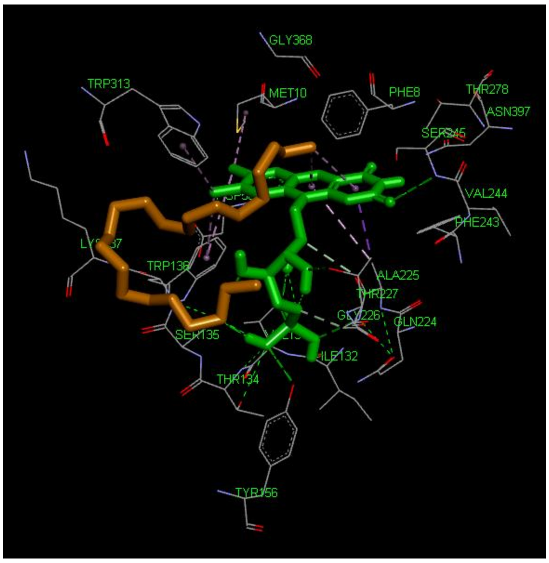

Af4: FMN: C24

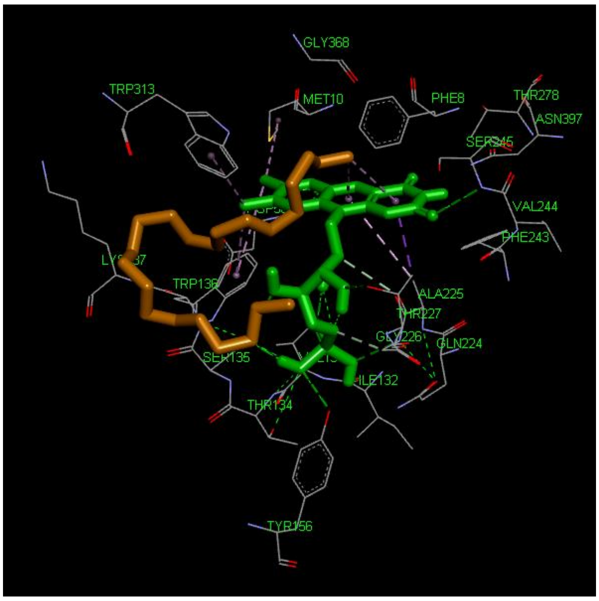

Af4: FMN: C25

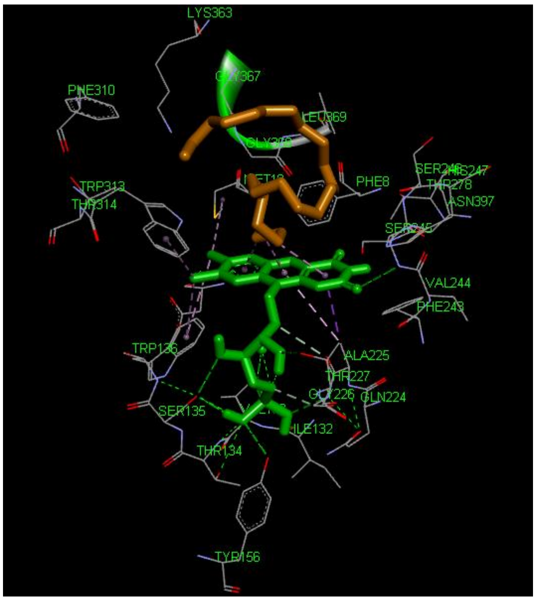

Af4: FMN: C26

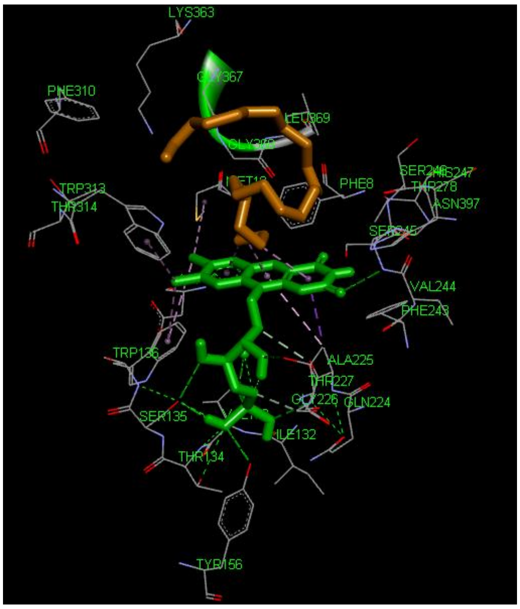

Af4: FMN: C27

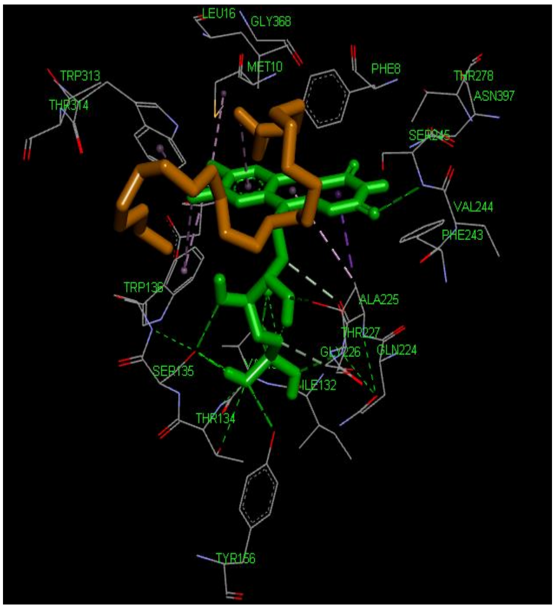

Af4: FMN: C28

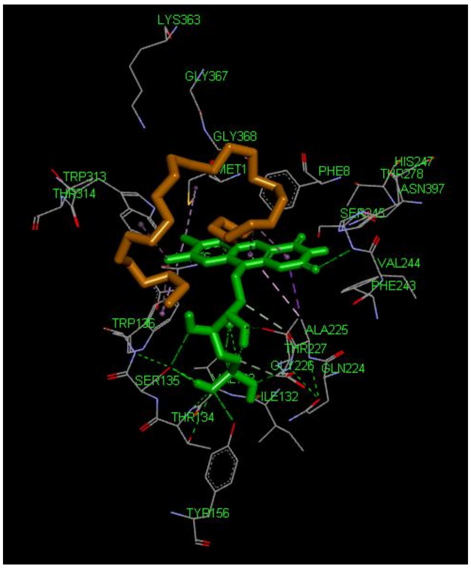

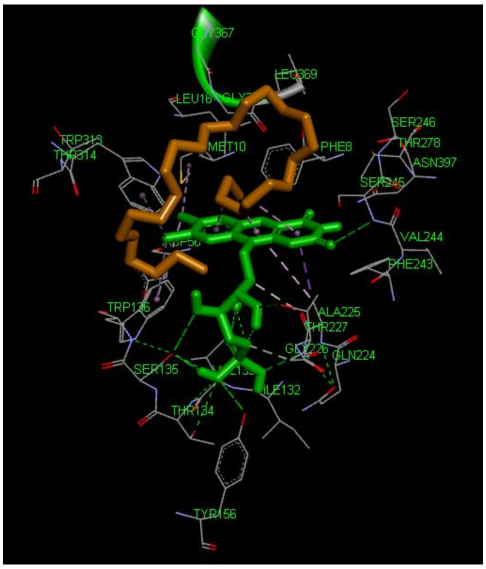

Af: FMN: C29

Af4: FMN: C30

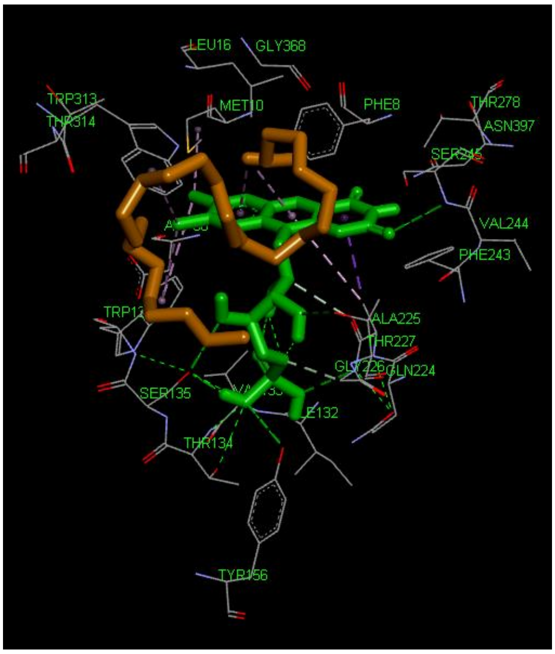

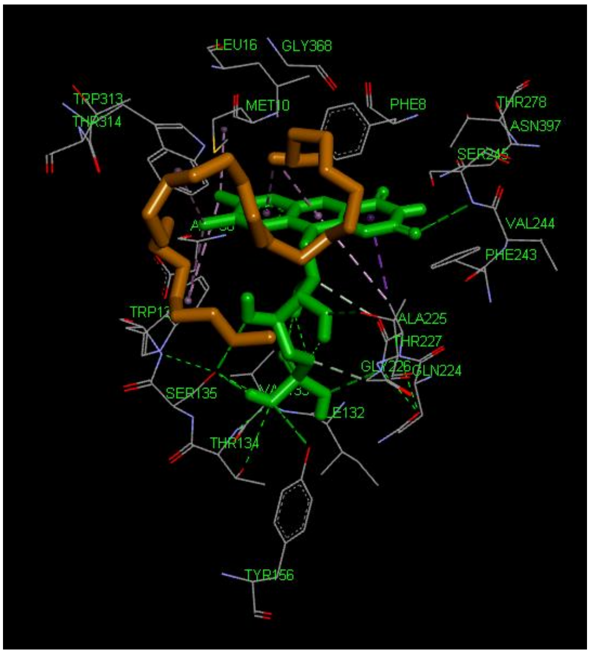

Supplement: Supplementary file 10 [file Image_9.pdf]
