# Supplementary material for: Identification of long-chain alkane-degrading (LadA) monooxygenases in Aspergillus flavus via in silico analysis
Source: Front Microbiol. 2022 Aug 30;13:898456. doi: 10.3389/fmicb.2022.898456 (PMC9468676; doi:10.3389/fmicb.2022.898456)

Supplementary Figure 10. The pocket residues of Af5: FMNopt: alkane (C17-C30) complexes visualized by BIOVIA Discovery Studio Visualizer; amino acid residues are found within 5 Å around the bound ligands inside the active pocket except for Tyr63 and Gln79. The distance between the terminal / subterminal carbon of the alkane with the pi-electron cloud of the FMN is <= 5 Å. Alkane (dark brown), FMN (green) and amino acid residues (gray). (Note: Figures depict actual amino acid residue numbers which differ from the reference amino acid positions mentioned in the manuscript, due to insertions / deletions; e.g: Tyr158 is identified as Tyr159 in Af5)

Af5: FMN: C17

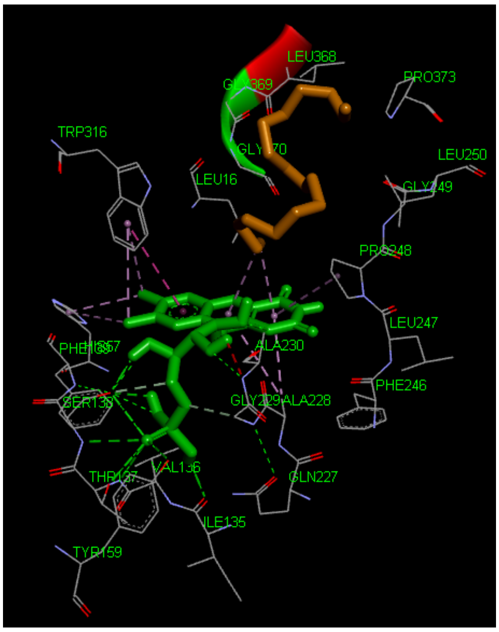

Af5: FMN: C18

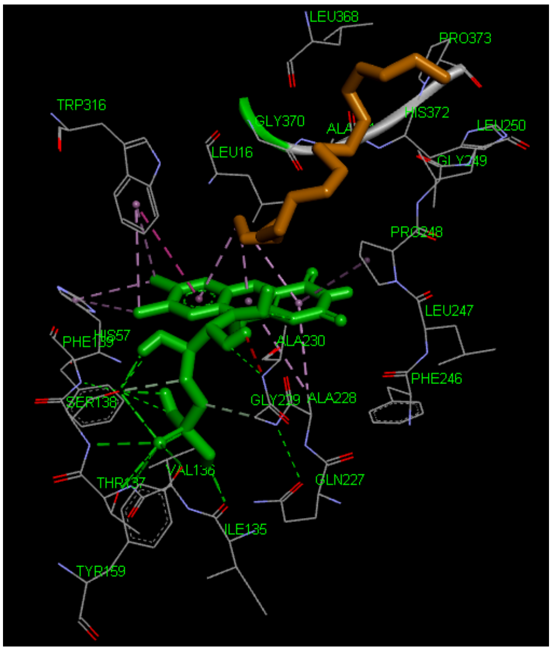

Af5: FMN: C19

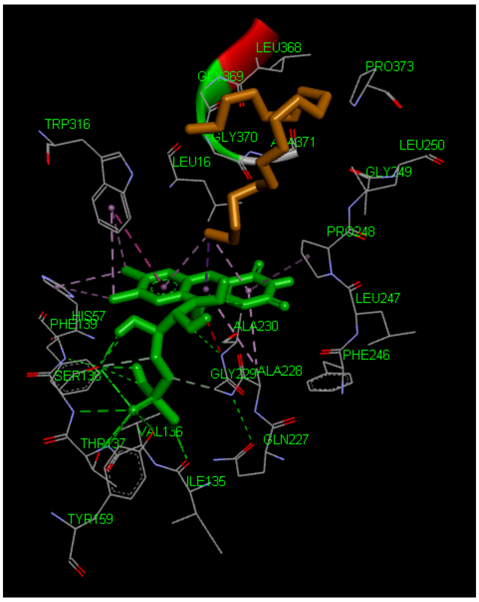

Af5: FMN: C20

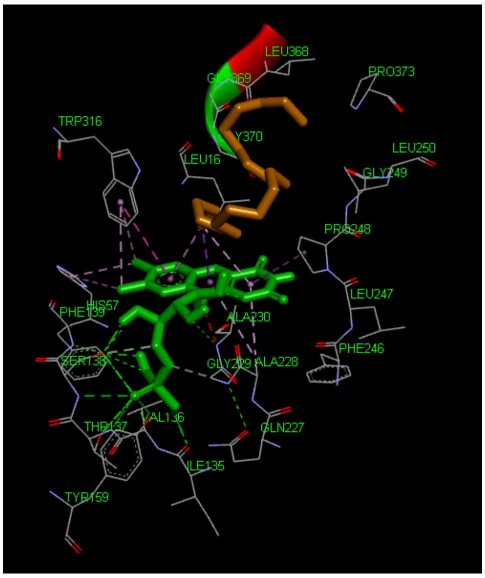

Af5: FMN: C21

Af5: FMN: C22

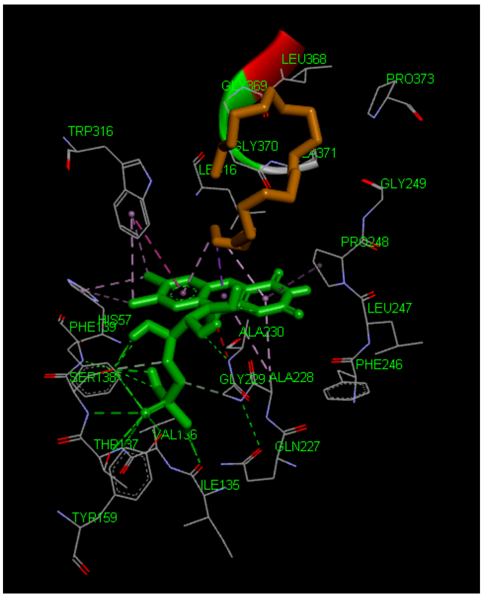

Af5: FMN: C23

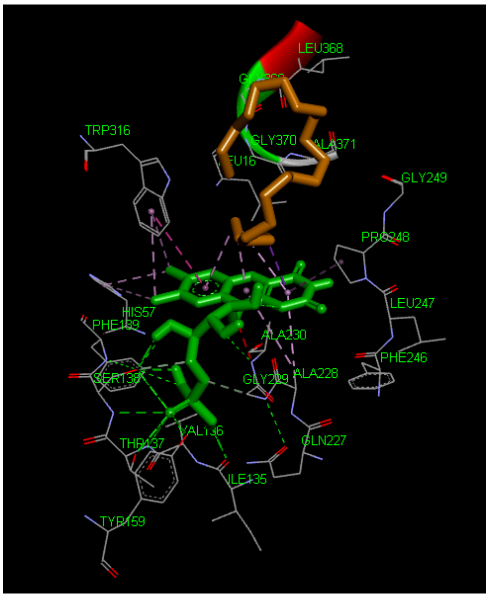

Af5: FMN: C24

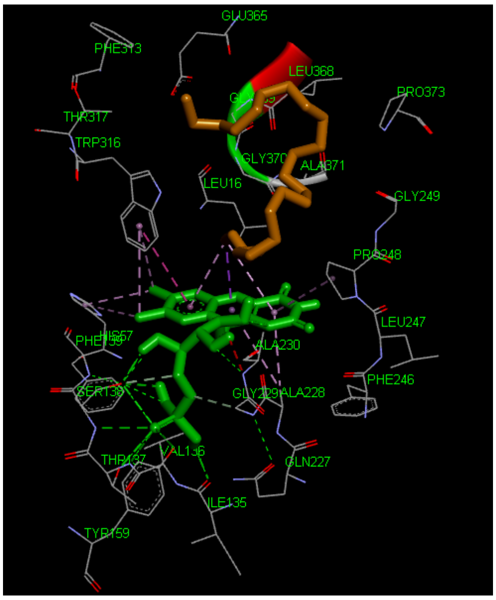

Af5: FMN: C25

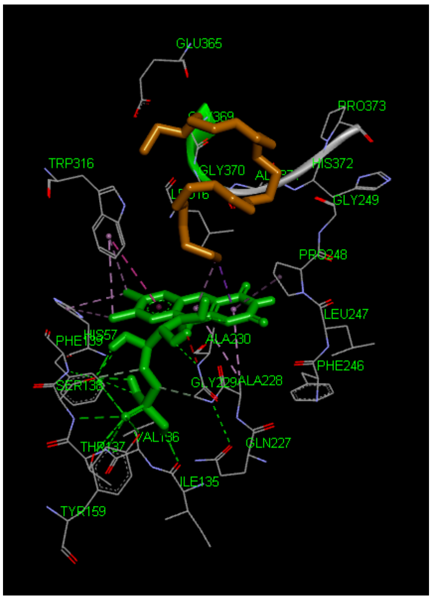

Af5: FMN: C26

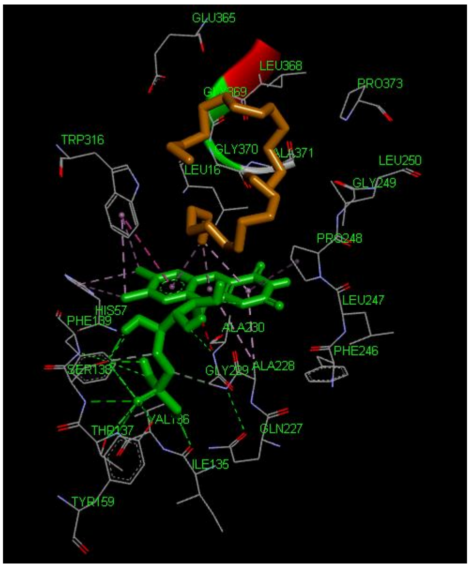

Af5: FMN: C27

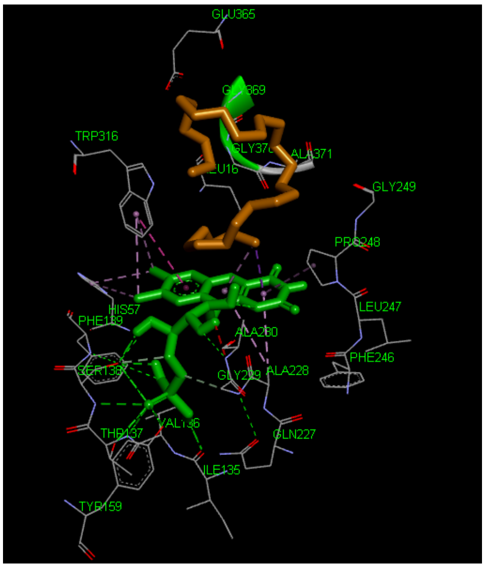

Af5: FMN: C28

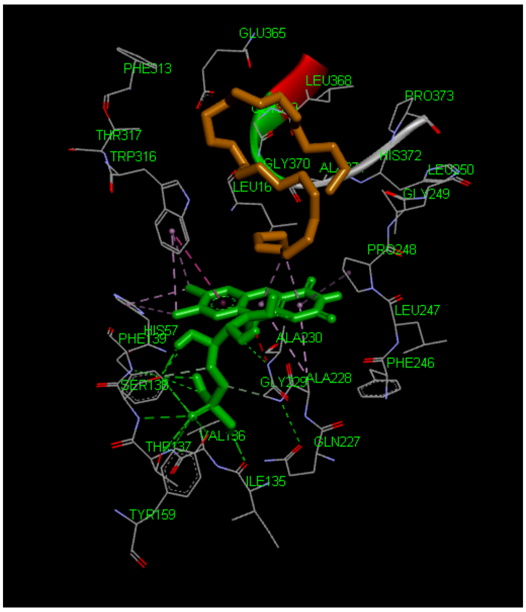

Af5: FMN: C29

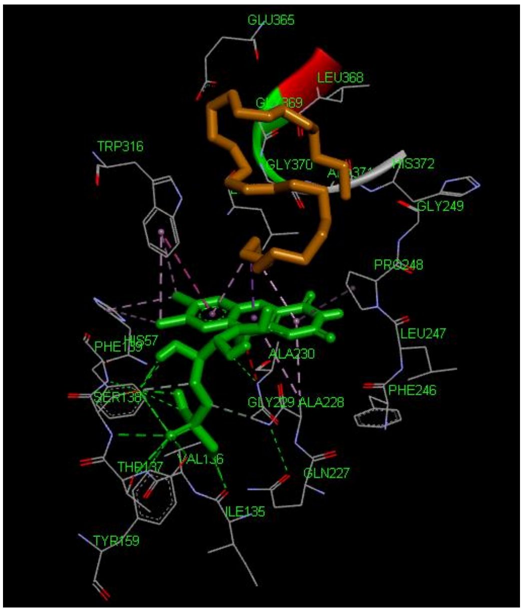

Af5: FMN: C30

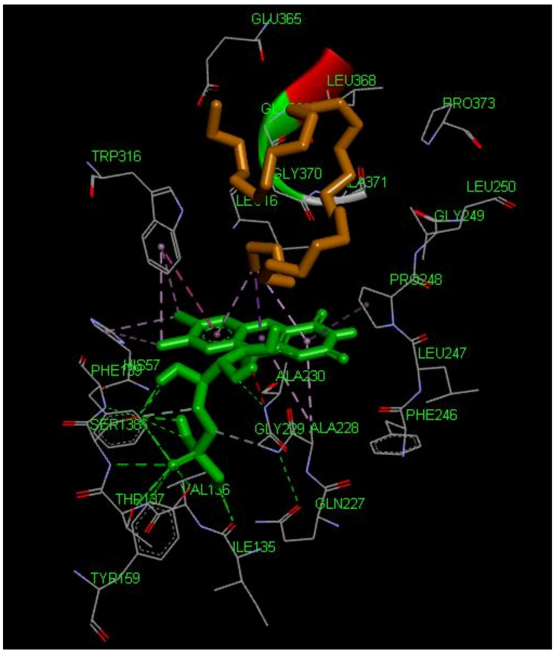

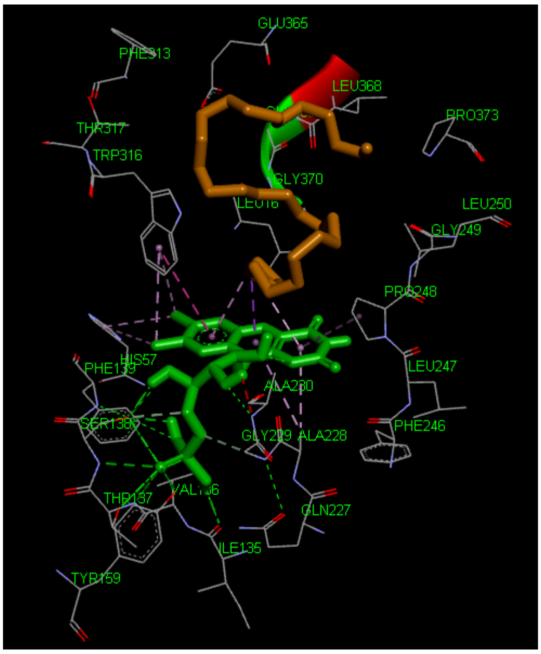

Supplement: Supplementary file 11 [file Image_10.pdf]
