# Supplementary material for: Identification of long-chain alkane-degrading (LadA) monooxygenases in Aspergillus flavus via in silico analysis
Source: Front Microbiol. 2022 Aug 30;13:898456. doi: 10.3389/fmicb.2022.898456 (PMC9468676; doi:10.3389/fmicb.2022.898456)
Supplement: Supplementary file 12 [file Image_11.pdf]

Supplementary Figure 11: Comparison of active pocket residues of *A. flavus* LadAα homologs with FMNopt and hexadecane: *Af1*, *Af3*, *Af4*, and *Af5* and re-docking analysis of 3B9O_A; residues within 5 Å from the bound ligands inside the active pocket are labelled in red lettering, while others are in black. Active pocket residues of *G. thermodentrificans* LadA (Li *et al.*, 2008) that were conserved but observed outside the active pocket in the *A. flavus* homologs are depicted in brackets.

Numbers below the residue name indicate actual residue numbers of sequences / protein 3D structures. The crystal structure of 3B90 chain A (3B90_A) used in redocking analysis starts from Lys (3rd residue in the complete sequence. As such a difference of two residue numbers are observed between 3B9O complete sequence and 3B9O_A.

Pocket residues described in Li et al., 2008	Substrate binding	Hydrophobic cavity	Substrate activation	Substrate binding		Hydrophobic cavity	FMN binding	Hydrophobic cavity	Substrate activation	Substrate activation					FMN binding	FMN binding
Geobacillus LadA (3B9O)	**Phe 10**	**Met 12**	**His 17**	**Ile 18**		**Ala 57**	**Asp 58**	**Val 59**	**Tyr 63**	**Gln 79**					**Ser 137**	**His 138**
Re-docking Analysis (3B9O_A)	Phe 8	(Met) 10	(His) 15	Ile 16	Phe 53	Ala 55	Asp 56	Val 57	(Tyr) 61	(Gln) 77	Thr 102	Asn 131	Val 133	Thr 134	Ser 135	His 136
Af1	Phe 8	(Glu) 10	His 15	Gln 16	Phe 53	Ala 55	Asp 56	Val 57	(Tyr) 61	(Gln) 79	Thr 104	Asn 133	Val 135	Thr 136	Gly 137	Tyr 138
Af3	Phe 8	Met 10	His 15	Leu 16	Phe 53	Ala 55	Asp 56	Val 57	(Tyr) 61	(Gln) 79	(Thr) 104	Asn 133	Val 135	Thr 136	Ser 137	Tyr 138
Af4	Phe 8	Met 10	His 15	Leu 16	Phe 53	Ala 55	Asp 56	Thr 57	(Tyr) 61	(Gln) 77	(Thr) 102	Asn 131	Val 133	Thr 134	Ser 135	Trp 136
Af5	Phe 8	Met 10	(His) 15	Leu 16	(Phe) 53	Ala 55	Asp 56	His 57	(Tyr) 61	(Gln) 79	(Thr) 104	Asn 134	Val 136	Thr 137	Ser 138	Phe 139

Pocket residues described in Li et al., 2008		FMN binding	FMN binding		FMN binding	FMN binding		FMN binding			Substrate activation			Substrate binding	Substrate binding	Substrate binding	
Geobacillus LadA (3B9O)		**Tyr 158**	**Ala 227**		**Met 229**	**Ser 230**		**Phe 245**			**His 311**			**Lys 347**	**Trp 348**	**Phe 349**	
Re-docking Analysis (3B9O_A)	HIS 152	Tyr 156	Ala 225	Gly 226	Met 227	Ser 228	Gly 231	Phe 243	Leu 244	Gly 245	His 309	Tyr 314	Gly 315	Lys 345	Trp 346	Phe 347	Asn 376
Af1	(His) 154	Tyr 158	Ala 227	Gly 228	Thr 229	Ser 230	Gly 233	Phe 245	Val 246	Ala 247	.	(Phe) 313	Trp 316	Gly 368	Leu 369	Gly 370	Asn 397
Af3	(His) 154	Tyr 158	Ala 227	Gly 228	Thr 229	Ser 230	Gly 233	Phe 245	Leu 246	His 247	.	(Phe) 312	Trp 315	Gly 368	.	.	Asn 398
Af4	(His) 152	Tyr 156	Ala 225	Gly 226	Thr 227	Ser 228	Gly 231	Phe 243	Val 244	Ser 245	.	(Phe) 310	Trp 313	Gly 367	Leu 369	.	Asn 397
Af5	(His) 155	Tyr 159	Ala 228	Gly 229	Ala 230	Ser 231	Gly 234	Phe 246	Leu 247	Pro 248	.	(Phe) 313	Trp 316	Gly 369	Ala 371	.	Asn 399
